# Longitudinal serum biomarker screening identifies malate dehydrogenase 2 as candidate prognostic biomarker for Duchenne muscular dystrophy

**DOI:** 10.1002/jcsm.12517

**Published:** 2019-12-27

**Authors:** Mirko Signorelli, Burcu Ayoglu, Camilla Johansson, Hanns Lochmüller, Volker Straub, Francesco Muntoni, Erik Niks, Roula Tsonaka, Anja Persson, Annemieke Aartsma‐Rus, Peter Nilsson, Cristina Al‐Khalili Szigyarto, Pietro Spitali

**Affiliations:** ^1^ Department of Biomedical Data Sciences Leiden University Medical Center Leiden The Netherlands; ^2^ Department of Protein Sciences, SciLifeLab, School of Engineering Sciences in Chemistry, Biotechnology and Health KTH Royal Institute of Technology Stockholm Sweden; ^3^ Department of Protein Science, School of Chemistry, Biotechnology and Health KTH Royal Institute of Technology Stockholm Sweden; ^4^ Department of Neuropediatrics and Muscle Disorders, Faculty of Medicine, Medical Center University of Freiburg Freiburg Germany; ^5^ Centro Nacional de Análisis Genómico (CNAG‐CRG), Center for Genomic Regulation Barcelona Institute of Science and Technology (BIST) Barcelona Spain; ^6^ Children's Hospital of Eastern Ontario Research Institute University of Ottawa Ottawa Canada; ^7^ Division of Neurology, Department of Medicine The Ottawa Hospital Ottawa Canada; ^8^ MRC Centre for Neuromuscular Diseases, Institute of Genetic Medicine Newcastle University Newcastle upon Tyne UK; ^9^ The Dubowitz Neuromuscular Centre UCL Institute of Child Health London UK; ^10^ Department of Neurology Leiden University Medical Center Leiden The Netherlands; ^11^ Department of Human Genetics Leiden University Medical Center Leiden The Netherlands; ^12^ Division of Affinity Proteomics, SciLifeLab, Department of Protein Science KTH Royal Institute of Technology Stockholm Sweden

**Keywords:** Duchenne muscular dystrophy, Protein biomarkers, Prognostic biomarker, Rare diseases, Serum biomarkers

## Abstract

**Background:**

Duchenne muscular dystrophy (DMD) is a fatal disease for which no cure is available. Clinical trials have shown to be largely underpowered due to inter‐individual variability and noisy outcome measures. The availability of biomarkers able to anticipate clinical benefit is highly needed to improve clinical trial design and facilitate drug development.

**Methods:**

In this study, we aimed to appraise the value of protein biomarkers to predict prognosis and monitor disease progression or treatment outcome in patients affected by DMD. We collected clinical data and 303 blood samples from 157 DMD patients in three clinical centres; 78 patients contributed multiple blood samples over time, with a median follow‐up time of 2 years. We employed linear mixed models to identify biomarkers that are associated with disease progression, wheelchair dependency, and treatment with corticosteroids and performed survival analysis to find biomarkers whose levels are associated with time to loss of ambulation.

**Results:**

Our analysis led to the identification of 21 proteins whose levels significantly decrease with age and nine proteins whose levels significantly increase. Seven of these proteins are also differentially expressed in non‐ambulant patients, and three proteins are differentially expressed in patients treated with glucocorticosteroids. Treatment with corticosteroids was found to partly counteract the effect of disease progression on two biomarkers, namely, malate dehydrogenase 2 (MDH2, *P* = 0.0003) and ankyrin repeat domain 2 (*P* = 0.0005); however, patients treated with corticosteroids experienced a further reduction on collagen 1 serum levels (*P* = 0.0003), especially following administration of deflazacort. A time to event analysis allowed to further support the use of MDH2 as a prognostic biomarker as it was associated with an increased risk of wheelchair dependence (*P* = 0.0003). The obtained data support the prospective evaluation of the identified biomarkers in natural history and clinical trials as exploratory biomarkers.

**Conclusions:**

We identified a number of serum biomarkers associated with disease progression, loss of ambulation, and treatment with corticosteroids. The identified biomarkers are promising candidate prognostic and surrogate biomarkers, which may support drug developers if confirmed in prospective studies. The serum levels of MDH2 are of particular interest, as they correlate with disease stage and response to treatment with corticosteroids, and are also associated with the risk of wheelchair dependency and pulmonary function.

## Introduction

Duchenne muscular dystrophy (DMD) is caused by lack of dystrophin as a result of mutations in the *DMD* gene.^1^ DMD patients experience a severe disease progression with disease milestones such as loss of ambulation, scoliosis, inability to self‐feed, cardio‐respiratory complications, and premature death.^2^, ^3^ The development of functional outcome measures in view of clinical trials and natural history studies has provided more details about DMD, enabling to better understand and quantify disease progression.^4^, ^5^, ^6^, ^7^, ^8^ However, the intra‐individual and inter‐individual variabilities in outcome measures have so far not enabled to properly power interventional studies and in retrospect have also accounted for underpowered studies up to Phase 3.^9^ The combination of noisy outcome measures and low drug potency has so far limited the availability of medicinal products to DMD patients.^10^ There is a growing interest in biomarker research to improve medical care, accelerate the development of drugs, and improve the design of clinical trials. While multiple biomarkers have shown potential response to dystrophin restoration in animal models,^11^ there is an urgent need for monitoring biomarkers able to anticipate disease milestones and clinical benefit in response to treatment. This type of biomarkers would enable drug developers to reduce the costs of clinical trials, while reducing the unnecessary exposure of patients to biological drugs, which often come with complicated patient management and increase risk of safety issues compared with conventional drugs. Biomarker research is ranging from MRI/MRS^12^, ^13^ to blood/urine based biomarkers^14^, ^15^, ^16^, ^17^, ^18^ in order to maximize the information for the whole body while reducing the need of resorting to invasive procedures such as obtaining muscle biopsies.

In this study, we analyse a longitudinal cohort of DMD patients, which is to our knowledge the largest cohort ever described. We provide a comprehensive evaluation of serum protein profiles focusing on the biomarkers abundance changes with disease progression. Protein profiles were analysed using an antibody‐based suspension bead array platform followed by a thorough analysis of signals over time. We identify protein profiles whose levels change significantly with age, are significantly different between ambulant and non‐ambulant patients or across treatment groups, and are significantly associated with time to loss of ambulation. Among those proteins, malate dehydrogenase 2 (MDH2) can be seen as a pivotal example of a disease monitoring biomarker, because it is associated not only with disease progression but also with an increased risk of disease milestones such as loss of ambulation and with clinical benefit in treatment with glucocorticoids.

## Materials and methods

### Characteristics of the subjects involved in the study

**Figure 1:**
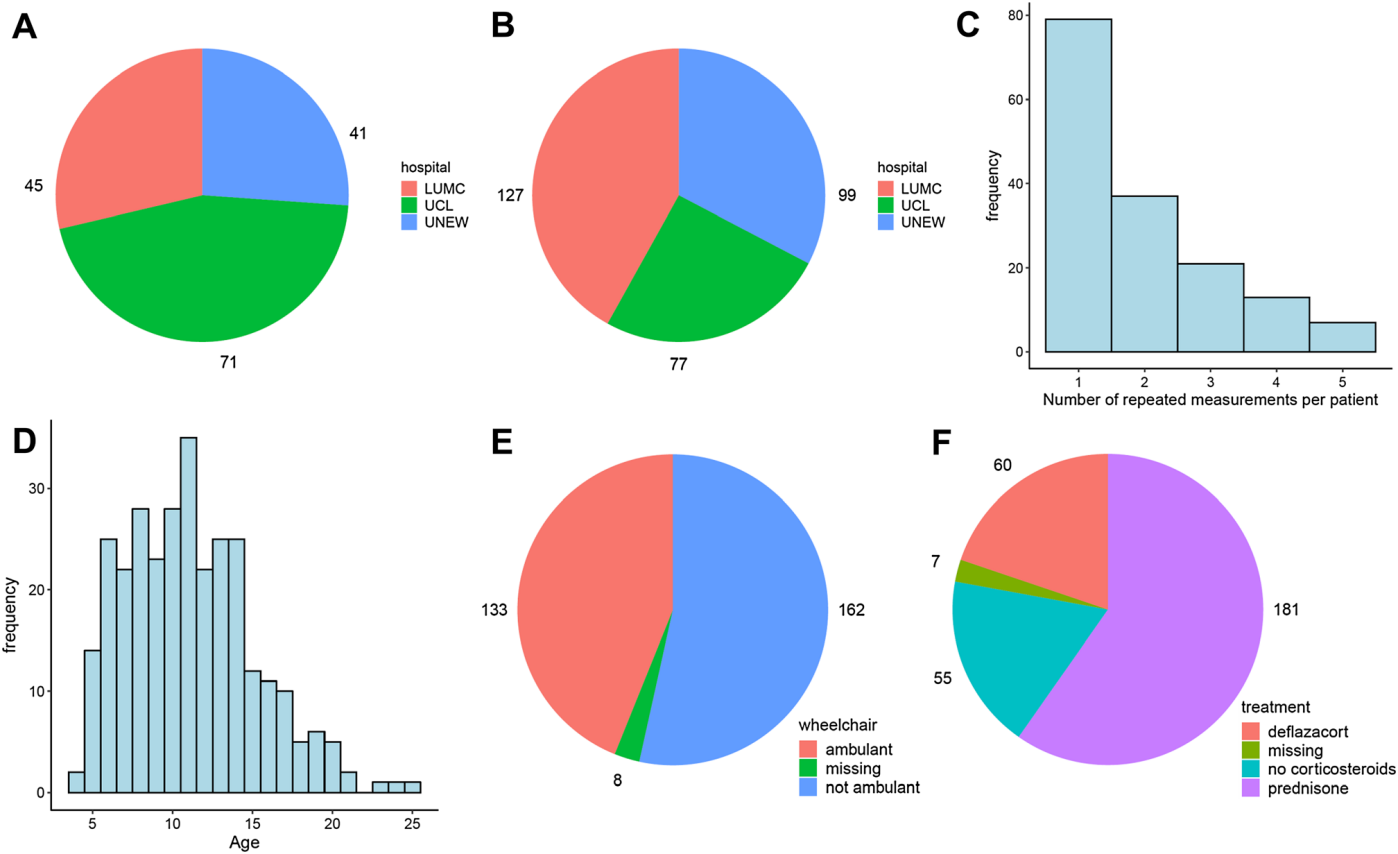
Description of the subjects and samples included in the study. (A) Distribution of patients across hospitals (LUMC, Leiden University Medical Center; UCL, University College London; UNEW, University of Newcastle). (B) Distribution of samples across hospitals. (C) Number of repeated measurements per patient. For 79 patients, only one measurement is available; for 78 patients, between two and five repeated measurements are available. (D) Distribution of age across samples. (E) Distribution of samples by ambulation status. (F) Distribution of samples by treatment group.

Patients involved in this study were followed up at three hospitals: the Leiden University Medical Center (hereafter referred to as LUMC), Leiden (NL); the Dubowitz Neuromuscular Centre, UCL Institute for Child Health (referred to as UCL), London (UK); and the John Walton Muscular Dystrophy Centre of the University of Newcastle (referred to as UNEW), Newcastle Upon Tyne (UK). A total of 303 serum samples were collected from 157 DMD patients; although more patients were followed at UCL (*Figure* 1A), longitudinal sampling was enriched at LUMC and UNEW (*Figure* 1B). All samples were shipped to the same location for analysis in order to centralize the analysis and reduce the variation introduced by different labs/operators. The study has been approved by the Institutional Review Board of the involved clinical centres. Informed consent forms were obtained for all participants. The investigation was conducted according to the Declaration of Helsinki.

For 79 patients only, one sample was collected; for 78 patients, between two and five repeated measurements were obtained (*Figure* 1C), with a median follow‐up time of 2 years (range: 0.3–7.4 years). The age of patients ranged between 3.6 and 25.3 years, with a median age of 10.8 years (*Figure* 1D). One hundred and thirty‐three samples were taken from ambulant and 162 from non‐ambulant patients; information on ambulation status was unknown for eight samples from eight different patients (*Figure* 1E). Two hundred and forty‐one samples were obtained from patients treated with glucocorticosteroids (181 were treated with prednisone/prednisolone and 60 with deflazacort) and 55 from patients who were not treated for at least 3 months prior to the date of sample collection; information on treatment with corticosteroids was not available for seven samples from seven patients (*Figure* 1F). Detailed information on the distribution of samples by age, ambulation status, and treatment group across hospitals can be found in *Table* 1.

**Table 1:** Overview of the characteristics of the subjects involved in the study

Hospital	Number of patients	Number of samples	Median age (range)	GC (%)	Prednisone among treated (%)	Ambulant (%)
LUMC	45	127	10.6 (4.7–19.4)	78.6	86.9	39.7
UCL	71	77	10.4 (3.6–15.8)	84.7	86.9	33.8
UNEW	41	99	11.3 (4–25.3)	82.7	51.9	60.2
Total	157	303	10.8 (3.6–25.3)	81.4	75.1	45.1

The 303 serum samples were collected from 157 patients at the Leiden University Medical Center (LUMC), University College London (UCL), and University of Newcastle (UNEW). Median age was 10.8, with a minimum age of 3.6 and a maximum age of 25.3; 81.4% of samples were collected from patients treated with glucocorticosteroids (%GC); among them, 75.1% received prednisone and 24.9% deflazacort; 45.1% of samples were collected from ambulant patients.

### Selection of candidate biomarkers

Numerous biomarkers associated with DMD have been identified, but information regarding individual variation over time is lacking due to the cross‐sectional nature of most studies. For this study, we selected protein targets by performing a review of the literature up to January 2015. Protein biomarkers were considered if abundance levels were differentially represented in serum and/or plasma samples obtained from DMD patients in comparison to healthy controls.^16^, ^18^, ^19^, ^20^, ^21^, ^22^, ^23^, ^24^, ^25^, ^26^ For each target, validated antibodies were selected from the Human Protein Atlas^27^ based on their specificity in immuno‐based applications (protein array, western blot, and immunohistochemical staining) and subsequently used to measure relative protein abundance with a suspension bead array platform as previously described.^16^ We considered 118 proteins, targeted by 240 antibodies listed in Table S1. To validate the protein profile measurements more than one antibody was used for each target if available. For 81 proteins, two or more antibodies were used; correlation between antibodies that measure the same protein ranged between −0.2 and 0.98, with a median value of 0.47; detailed comparisons between antibodies that target the same protein are presented in  File S1.

### Generation of antibody bead arrays and protein profiling in serum

Antibodies were diluted in buffer and immobillized on carboxylated magnetic beads with different IDs (Luminex Corp.) as described previously^16^; 3 μL of each serum sample was transferred to microtiter plates and labelled with biotin. The labelling reaction was stopped by adding 0.5 M Tris‐HCL, pH 8.0.^16^ The samples were subsequently heat treated, and 1 μL was diluted in 50 μL of assay buffer consisting of PBS‐T 0.05%, 10% v/v rabbit IgG, and 1:1000 ProClin™300 (Sigma Aldrich) and incubated overnight at room temperature with the generated antibody bead array. For detection of captured proteins, the beads were washed and incubated with R‐phycoerythrin conjugated streptavidin (Invitrogen). After washing the beads, raw median fluorescent intensity (MFI) and the total bead count was recorded for each target analysed in each sample^16^ in a Luminex FM3D instrument (Luminex Corp.). Raw values were normalized using probabilistic quotient normalization method^28^, ^29^ prior to further analysis.

### Analysis of longitudinal protein expression with linear mixed models

To study the dynamic evolution of each antibody, we considered linear mixed models where the normalized log‐MFI value of each protein depends on age, hospital, wheelchair dependence, and on the type of corticosteroids used (none, prednisone, or deflazacort); linear mixed models^30^ are an extension of the linear regression model that can be employed to analyse longitudinal data. Because MFI values of most proteins were positively skewed, MFI values were log‐transformed to reduce their asymmetry and improve their approximation to normality. Correlations between repeated measurements from the same individual were modelled through a flexible random effect structure that comprises a random intercept and a random slope for age; the random intercept was allowed to have a different variance in the three treatment groups. We employed the likelihood ratio test to simplify, when possible, the random effects part of the model. Then, we employed the Wald test to identify proteins whose MFI levels are associated with age and proteins that are differentially expressed in wheelchair‐dependent patients. Moreover, we used the *F* test to identify proteins that are differentially expressed across treatment groups and across hospitalsS2. We applied the Benjamini–Hochberg procedure^31^ to correct for multiple testing.

**Table 2:** List of proteins significantly associated with age

Protein	Antibody	*β*_*AGE*_	*P*‐value	FDR
MDH2	HPA019848	−0.093	1.7E‐27	4.0E‐25
ETFA	HPA018990	−0.056	2.9E‐24	3.4E‐22
MYL3	HPA016564	−0.061	2.8E‐22	2.2E‐20
NES	HPA026111	−0.067	2.6E‐21	1.6E‐19
CK	HPA001254	−0.065	1.2E‐17	5.6E‐16
CA3	HPA021775	−0.053	7.6E‐12	3.1E‐10
MYOM3	HPA029752	−0.026	2.6E‐09	9.1E‐08
LDHB	HPA019007	−0.032	1.5E‐08	4.4E‐07
COL1A1	HPA011795	−0.022	1.8E‐08	4.9E‐07
ENO3	HPA000793	−0.027	1.2E‐07	2.9E‐06
BASP1	HPA050333	−0.023	2.3E‐07	5.1E‐06
C4A	HPA048287	0.027	6.9E‐07	1.3E‐05
MGP	HPA014274	0.023	7.0E‐07	1.3E‐05
TNNT3	HPA037810	−0.023	7.4E‐07	1.3E‐05
MAP 4	HPA038150	−0.024	3.4E‐06	5.3E‐05
TTN	HPA007042	−0.033	3.6E‐06	5.3E‐05
C4A	HPA046356	0.022	4.2E‐06	5.9E‐05
DES	HPA018803	−0.026	6.7E‐06	9.0E‐05
NES	HPA006286	−0.019	1.7E‐05	0.0002
TNNT2	HPA015774	−0.031	3.5E‐05	0.0004
AKAP1	HPA008691	−0.021	4.5E‐05	0.0005
ANKRD2	HPA040884	−0.019	5.1E‐05	0.0006
MGP	HPA013949	0.015	0.0001	0.0011
CA3	HPA026700	−0.021	0.0002	0.0016
C4BPA	HPA000926	0.014	0.0002	0.0019
GSN	HPA070538	0.013	0.0002	0.0019
HDAC2	HPA011727	−0.03	0.0003	0.0029
LCP1	HPA019493	−0.013	0.0005	0.0044
C3	HPA003563	0.008	0.0008	0.0068
C4BPA	HPA001797	0.009	0.0010	0.0077
C4A	HPA050103	0.015	0.0017	0.0130
CFH	HPA049176	0.005	0.0018	0.0133
CFH	HPA053326	0.01	0.0024	0.0173
C4BPA	HPA001578	0.008	0.0031	0.0217
KRT10	HPA012014	−0.02	0.0042	0.0286
RELB	HPA011985	0.015	0.0053	0.0353
PDZK1	HPA005755	0.011	0.0056	0.0363
FH	HPA027341	0.015	0.0069	0.0431
AKAP1	HPA008620	−0.014	0.0070	0.0431

Results from the test on the effect of age on protein expression. The effect is significant (FDR < 0.05) for 30 proteins that are targeted by 39 antibodies. *β*_*AGE*_ denotes the effect of a unit increase in age on the log‐expression value of each antibody, *P*‐value is the *P*‐value of the Wald test on the significance of *β*_*AGE*_, and FDR is the false discovery rate from the Benjamini–Hochberg multiple testing correction.

**Table 3:** List of proteins differentially expressed in wheelchair‐dependent patients.

Protein	Antibody	*β*_*WHEELCHAIR*_	*P*‐value	FDR
MDH2	HPA019848	−0.253	7.5E‐08	1.8E‐05
ETFA	HPA018990	−0.15	2.2E‐05	0.0027
CFH	HPA049176	0.047	3.5E‐05	0.0028
C3	HPA003563	0.07	0.0001	0.0079
MYL3	HPA016564	−0.133	0.0003	0.0156
CK	HPA001254	−0.161	0.0007	0.0284
C4BPA	HPA001578	0.067	0.0012	0.0405

Results of the test on the effect of wheelchair dependence on protein expression. The effect is significant (FDR < 0.05) for seven proteins. *β*_*WHEELCHAIR*_ denotes the expected log‐MFI difference of each antibody between wheelchair dependent and ambulant patients. *P*‐value is the *P*‐value of the Wald test on the significance of *β*_*WHEELCHAIR*_, and FDR is the false discovery rate from the Benjamini–Hochberg multiple testing correction.

Estimation of the linear mixed models and hypothesis testing was performed with the R package nlme.^32^ We identified one outlier sample, which was excluded from the analysis alongside with nine samples for which information on ambulation status and/or treatment with glucocorticosteroids was missing. Therefore, each model was estimated based on 293 samples from 149 patients.

### Survival analysis

The relationship between biomarker abundance and time to loss of ambulation was investigated with a penalized Cox proportional‐hazards model for time‐dependent covariates.^33^ This is an extension of the Cox model that allows to study the effect of covariates that change over time on a survival outcome and can thus be employed to study the effect of longitudinal biomarkers on time to loss of ambulation. The analysis was performed on a sample of 52 patients, 15 of which lost ambulation in the course of the study, and it was carried out with the R package survival.^34^

We first identified 30 promising proteins that were found to be differentially expressed over time in the longitudinal analysis of gene expression. For some proteins, measurements on more than one antibody was available; in order to reduce the multiple testing burden, for each protein, we selected the antibody with stronger evidence (smaller *P*‐value) of dynamic change. Then, we tested whether each biomarker is significantly associated with loss of ambulation by comparing a model where age, the biomarker, and their interactions are included as covariates to a null model where only age is included. Finally, we computed the false discovery rate (FDR) using the Benjamini–Hochberg correction for multiple testing.^31^

## Results

### Identification of proteins associated with age, wheelchair dependency, and treatment with glucocorticoids

Biomarker candidates were selected for this study by performing a review of the literature up to January 2015. Numerous biomarkers associated with DMD have been identified, but information regarding individual variation over time is lacking due to the cross‐sectional nature of the studies. One of the main reasons is the scarce availability of samples from patients affected by rare disorders, in particular longitudinally collected patient material. The advantage of longitudinal studies over cross‐sectional designs is that longitudinal studies do not only allow assessment of differences between individuals but also to study individual changes over time. However, in longitudinal studies repeated measurements from the same subject are not independent but correlated; mixed models allow accounting for this correlation through the use of subject‐specific random effects. Therefore, we employed linear mixed models to analyse the dynamic evolution of protein profiles to identify proteins that are associated with age, that are altered in wheelchair‐dependent patients, or that are altered across treatment groups. We derived estimates of the effect of each covariate on protein profiles and identified significant effects after multiple testing correction. To ensure that analysis across clinical centres can be performed, we corrected the analysis per clinical centre. The importance of this correction is highlighted by the fact that we found significant differences across clinical centres for 99 proteins, targeted by 168 antibodies (FDR < 5%, *Table* S2).

#### A signature of 30 proteins is associated with disease progression

**Figure 2:**
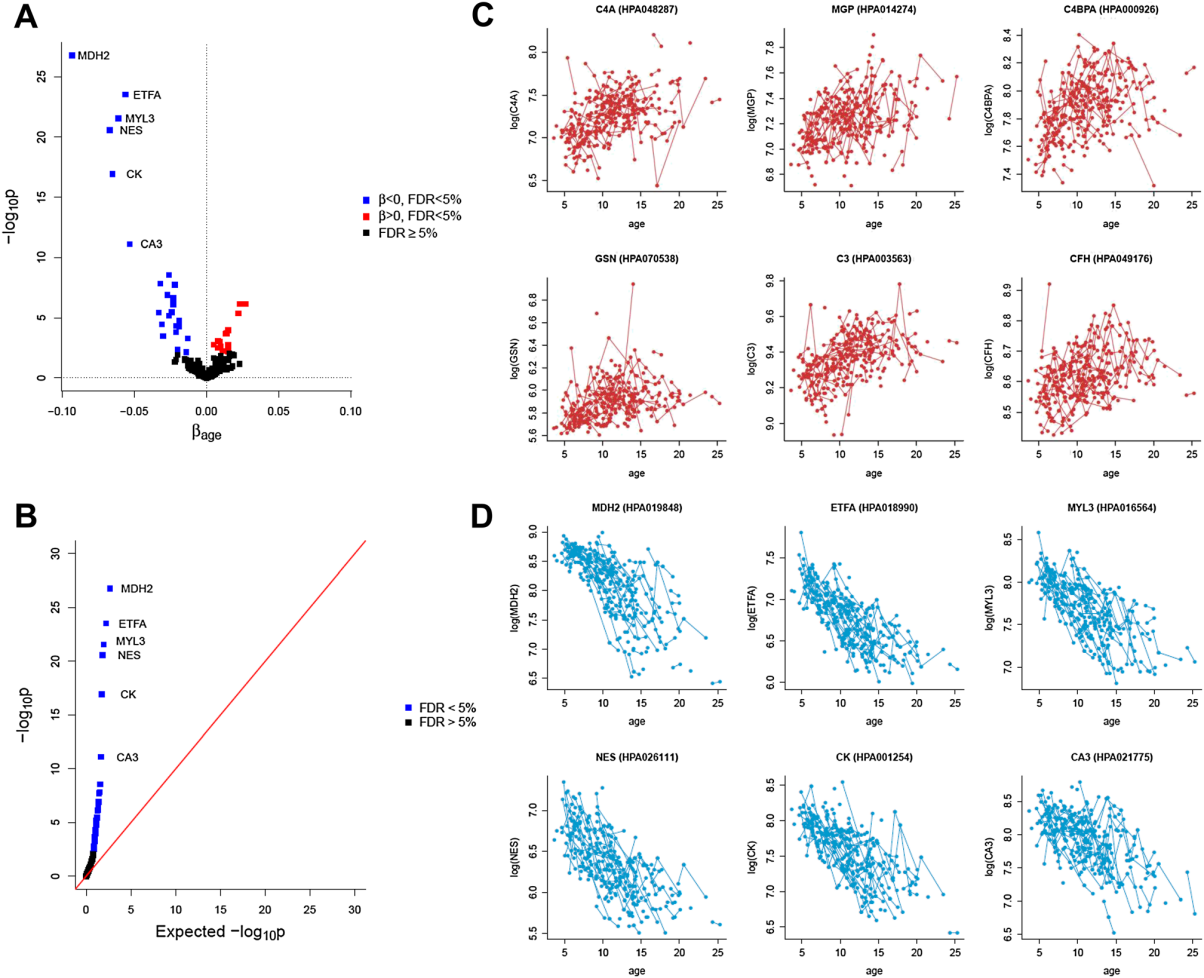
Serum protein biomarkers that are significantly associated with age. (A) Volcano plot with the results of the test on the significance of age. Thirty proteins targeted by 39 antibodies, listed in *Table* 2, are significant at 5% level after FDR correction for multiple testing. (B) P‐p plot for the test on age. The plot compares the expected −*log*_10_*p*‐values when the null hypothesis is true (*x*‐axis) to the −*log*_10_*p*‐values obtained from the test. (C) Biomarker abundance plot illustrating individual trajectories for the top 6 proteins whose levels significantly increase with age. (D) Biomarker abundance plot illustrating individual trajectories for the top 6 proteins whose levels significantly decrease with age.

Hypothesis testing on the effect of age led to the identification of 30 proteins significantly associated with age (FDR < 5%, *Table* 2 and *Figure* 2A and 2B). Notably, for some of those proteins, the association with age was confirmed with one or several different antibodies (when available), allowing a first layer of technical validation. Nine proteins showed increasing intensity with age (*Figure* 2C and File S2); these were C4A, MGP, C4BPA, GSN, C3, CFH, RELB, PDZK1, and FH. Twenty‐one proteins, instead, decreased significantly with age (*Figure* 2D and File S2). These were MDH2, ETFA, MYL3, NES, CK, CA3, MYOM3, LDHB, COL1A1, ENO3, BASP1, TNNT3, MAP 4, TTN, DES, TNNT2, AKAP1, ANKRD2, HDAC2, LCP1, and KRT10. All biomarkers, except GSN, LDHB, ENO3, DES, LCP1, and KRT10 were analysed using several antibodies recognizing different epitopes. C4A and C4BPA were detected and confirmed by three different antibodies whereas MGP, CFH, NES, CA3, and AKAP1 were confirmed by two different antibodies. All markers except GSN and RELB showed reliable detection levels with an average MFI above 400 units.

#### MDH2, ETFA, CFH, C3, MYL3, CK, and C4BPA discriminate between ambulant and non‐ambulant patients

**Figure 3:**
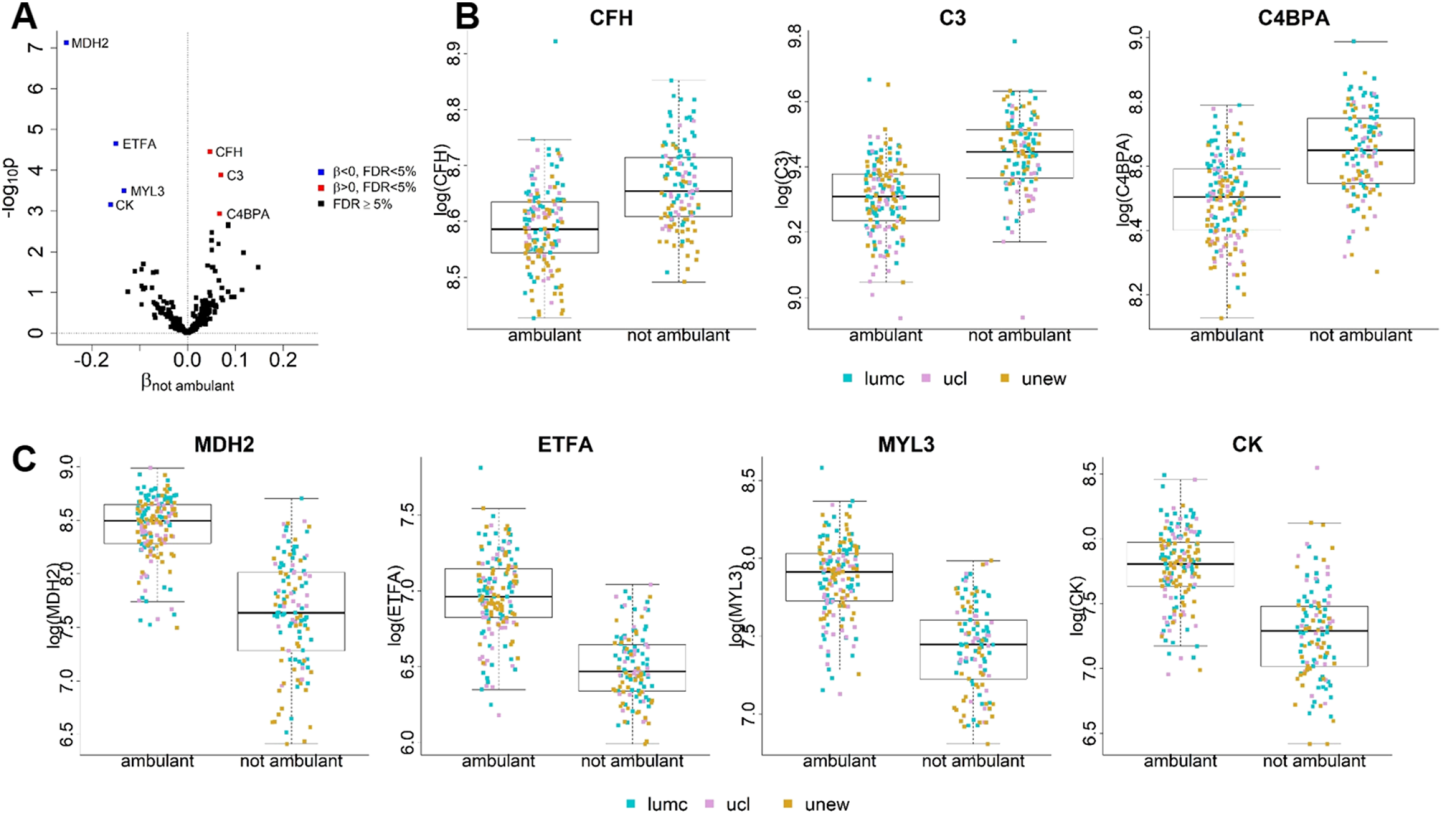
Differentially abundant protein biomarkers in non‐ambulant patients. (A) Volcano plot with the results of the test on the significance of ambulation status. Seven proteins, listed in *Table* 3, are significant at 5% level after FDR correction for multiple testing. (B–C) Boxplots comparing protein expression levels in ambulant and non‐ambulant patients for each of the significant proteins. Points denoting individual measurements are plotted using colours to distinguish hospital. (B) proteins that are elevated in non‐ambulant patients; (C) proteins that are reduced in non‐ambulant patients.

We further identified seven proteins that show a different relative abundance level between ambulant and non‐ambulant patients (FDR < 5%; *Table* 3 and *Figure* 3A). The serum levels of MDH2, ETFA, MYL3, and CK were significantly lower in wheelchair‐dependent patients (*Figure* 3C), whereas CFH, C3, and C4BPA were higher in these patients (*Figure* 3B). Because the mixed model allows us to estimate the conditional effect of loss of ambulation after accounting for the effect of age (as well as treatment and hospital) and the direction of changes in non‐ambulant patients is in the same direction of the age effect, this result points out that for these seven proteins there is an additional change on top of the age effect for non‐ambulant patients.

#### MDH2, ANKRD2, and COL1A1 are affected by treatment with glucocorticosteroids

**Figure 4:**
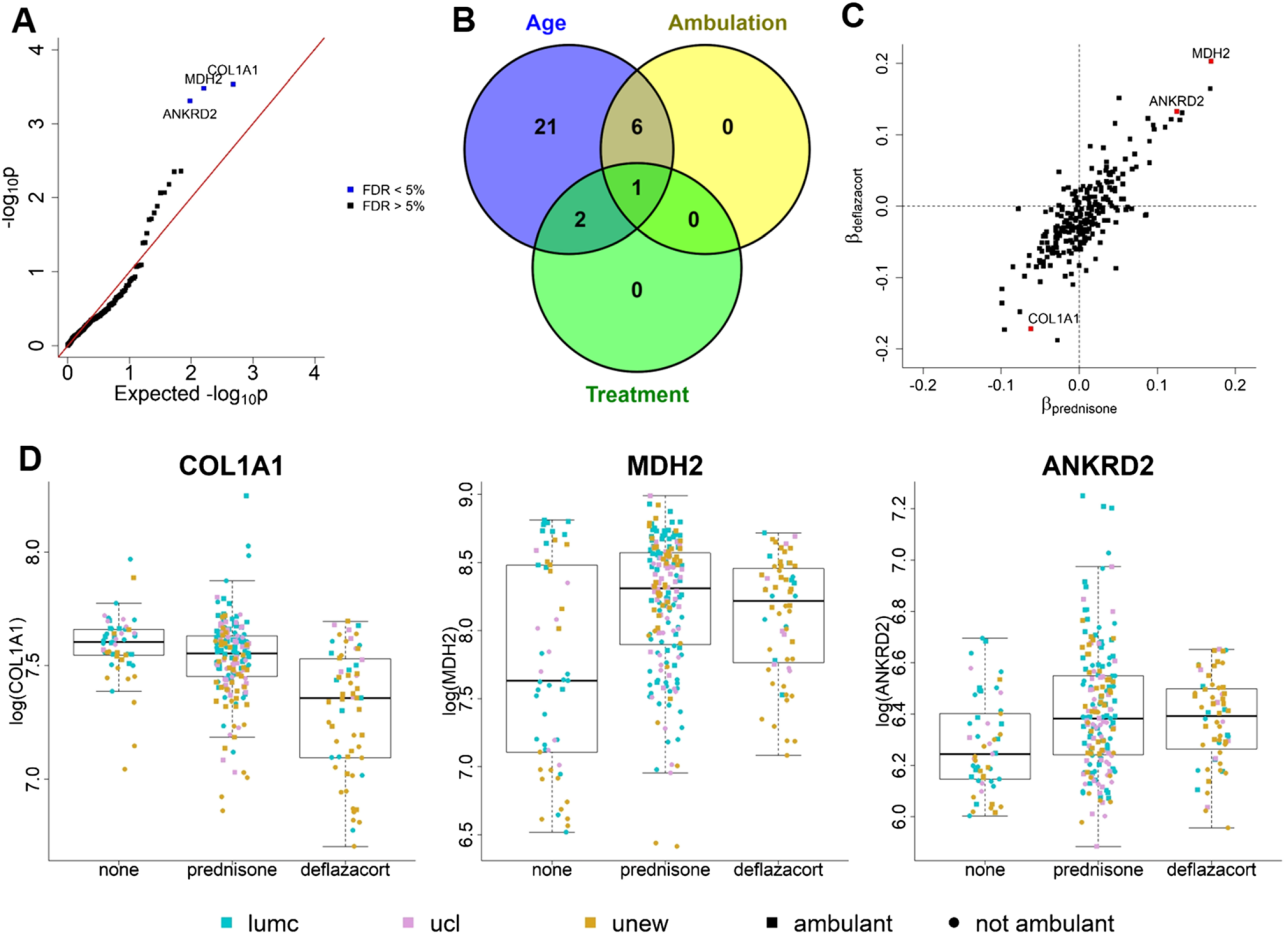
Effect of glucocorticosteroid treatment on COL1A1, MDH2 and ANKRD2. (A) Result of the test on differences between the three treatment groups. The plot compares the expected −*log*_10_*p*‐values when the null hypothesis is true (*x*‐axis) to the −*log*_10_*p*‐values obtained from the test. COL1A1, MDH2, and ANKRD2 are significant at 5% level after FDR correction for multiple testing. (B) Overlap between the lists of proteins significantly associated with age, loss of ambulation, and treatment. (C) Comparison of the effect sizes of prednisone and deflazacort across proteins. It can be observed that prednisone and deflazacort tend to have similar effects on several proteins (Pearson's correlation coefficient = 0.795). The points corresponding to COL1A1, MDH2, and ANKRD2 are highlighted in red. (D) Boxplots comparing protein expression levels across treatment groups for each of the significant proteins. Points denoting individual measurements are plotted using colours to distinguish hospital and shape to distinguish between samples from ambulant and from non‐ambulant patients.

To test whether treatment with glucocorticoids, a symptomatic treatment that is part of the standards of care for DMD patients, affected the protein profiles in blood, we compared patients treated with prednisone, patients treated with deflazacort, and untreated patients. Three proteins, namely, COL1A1, MDH2, and ANKRD2, showed significant differences across these three groups (*Table* 4A and *Figure* 4A). Each of these proteins was also found to be significantly associated with age, and MDH2 also with loss of ambulation (*Figure* 4B). Overall, we found the effect sizes of prednisone and deflazacort to be similar for most proteins (*Figure* 4C). For the three proteins with significant differences across treatment groups, paired comparisons showed that MDH2 and ANKRD2 were elevated in patients treated with either prednisone or deflazacort compared with patients who did not receive corticosteroids; no difference was observed between prednisone and deflazacort‐treated patients. Instead, the level of COL1A1 was significantly lower in treated patients compared with untreated ones; however, COL1A1 levels were further reduced in deflazacort‐treated patients compared with prednisone treated ones (*Table* 4B and *Figure* 4D).

**Table 4A:** List of proteins that are differentially expressed in patients treated with corticosteroids

Protein	Antibody	*β*_*PREDNISONE*_	*β*_*DEFLAZACORT*_	*P*‐value	FDR
COL1A1	HPA011795	−0.062	−0.172	0.0003	0.0394
MDH2	HPA019848	0.169	0.203	0.0003	0.0394
ANKRD2	HPA040884	0.125	0.133	0.0005	0.0394

Test on differences in protein levels between treatment groups. The effect is significant (FDR < 0.05) for three proteins. *β*_*PREDNISONE*_ and *β*_*DEFLAZACORT*_ respectively denote the effects of prednisone and deflazacort use on the log‐MFI value of the antibody, in comparison to patients who were not treated with corticosteroids. *P*‐value is the *P*‐value of the *F* test on the significance of treatment with corticosteroids (*H*_0_ : *β*_*PREDNISONE*_ = *β*_*DEFLAZACORT*_ = 0), and FDR is the *P*‐value after application of the Benjamini–Hochberg multiple testing correction.

**Table 4B:** Tests on differences between pairs of treatment groups

Protein	Antibody	FDR P vs. U	FDR D vs. U	FDR P vs. D
COL1A1	HPA011795	0.0179	0.0002	0.0047
MDH2	HPA019848	0.0006	0.001	0.4593
ANKRD2	HPA040884	0.0014	0.0014	0.8087

We tested differences between pairs of treatment groups for the three proteins that showed an overall significant difference between groups. The table reports the false discovery rates for the comparison between patients treated with prednisone (P) and those who did not receive corticosteroids (U), between patients treated with deflazacort (D) and those who did not receive corticosteroids (U), and between patients treated with prednisone (P) or with deflazacort (D).

Of note, treatment with steroids appears to counterbalance the age‐dependent decrease of MDH2 and ANKRD2; on the other hand, it further reduces COL1A1 serum levels on top of age. To clarify the interplay between age and steroids, we included a post hoc comparison with the interaction between age and treatment groups (*Table* S3). The interaction term was significant for MDH2 (*P* = 0.009) and COL1A1 (*P* = 0.006) but not for ANKRD2 (*P* = 0.088). We found the mean yearly decrease in MDH2 to be attenuated in patients treated with both prednisone and deflazacort in comparison to untreated patients. On the other hand, the reduction of COL1A1 with age is worsened by treatment with deflazacort, while treatment with prednisone does not result in any significant change.

### MDH2, KRT10, and DES are associated with an increased risk of wheelchair dependency

**Figure 5:**
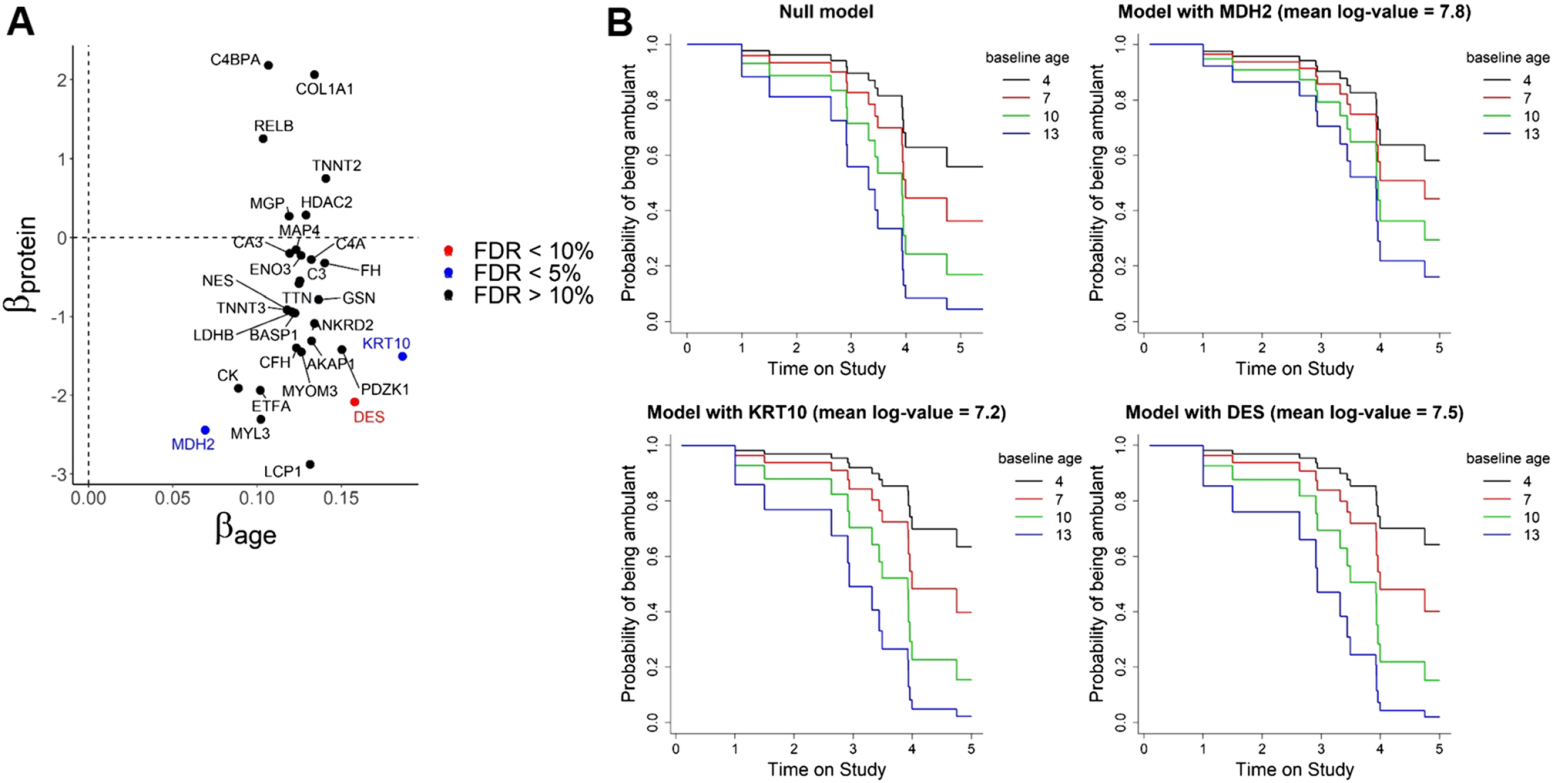
Proteins with significant improvement in the prediction of loss of ambulation. (A) Scatter plot with the effect sizes of the main effects of age (*x*‐axis) and protein. Proteins significant at 5% (MDH2 and KRT10) and 10% (DES) levels are highlighted. (B) Survival curves for different baseline ages from the null model with baseline age as only covariate, and from the three significant models where besides baseline age, also the expression level of a protein and its interaction with age are included as covariates.

To understand whether protein profiles showing changes with disease progression may be used to predict disease milestones, we investigated whether any of the 30 proteins associated with age were associated with time to loss of ambulation after accounting for baseline age. Survival analysis was performed including patients who were still ambulant when their first sample was taken. A total of 52 patients were included in this analysis. Loss of ambulation was observed for 15 patients during the study, while 37 patients were right censored as they were still able to walk when the last samples were obtained. We found KRT10 and MDH2 to be significantly associated with time to loss of ambulation (FDR < 5%, *Table* 5 and *Figure* 5). Weaker evidence of association with time to ambulation loss was also found for DES (FDR < 10%).

**Table 5:** Significance of the association between protein levels and time to loss of ambulation, after correction for age

Protein	Antibody	*P*‐value	FDR
KRT10	HPA012014	0.0018	0.0388
MDH2	HPA019848	0.0026	0.0388
DES	HPA018803	0.0076	0.0758
MYL3	HPA016564	0.0139	0.1044
CK	HPA001254	0.0221	0.1204
COL1A1	HPA011795	0.0261	0.1204
ETFA	HPA018990	0.0281	0.1204
C4BPA	HPA000926	0.0375	0.132
LCP1	HPA019493	0.0396	0.132
TNNT2	HPA015774	0.099	0.2736
PDZK1	HPA005755	0.1088	0.2736
MYOM3	HPA029752	0.1198	0.2736
RELB	HPA011985	0.1276	0.2736
AKAP1	HPA008691	0.1277	0.2736
NES	HPA026111	0.1594	0.3188
ANKRD2	HPA040884	0.1975	0.3703
LDHB	HPA019007	0.2373	0.4162
TNNT3	HPA037810	0.259	0.4162
TTN	HPA007042	0.2636	0.4162
HDAC2	HPA011727	0.3182	0.4651
GSN	HPA070538	0.3424	0.4651
BASP1	HPA050333	0.3428	0.4651
C3	HPA003563	0.3599	0.4651
CFH	HPA049176	0.3721	0.4651
C4A	HPA048287	0.4092	0.4765
MGP	HPA014274	0.4267	0.4765
MAP 4	HPA038150	0.4289	0.4765
FH	HPA027341	0.4797	0.4926
CA3	HPA021775	0.4864	0.4926
ENO3	HPA000793	0.4926	0.4926

Results of the test on the significance of each antibody in a Cox model where we controlled for baseline age. *P*‐value is the *P*‐value of the Wald test on the significance of the antibody (main effect + interaction with baseline age), and FDR is the *P*‐value after application of the Benjamini–Hochberg multiple testing correction.

## Discussion

The work that we present in this manuscript aimed to identify blood‐based, relatively non‐invasive biomarkers associated with disease progression, disease milestones, and clinical benefit following treatment with corticosteroids in DMD. Towards this aim, we studied the abundance profile of a number of serum proteins known to be linked to DMD pathophysiology. The list of targets included proteins already shown to distinguish between DMD patients and healthy controls, as well as gene products of known genetic modifiers.^16^, ^35^, ^36^ A total of 118 proteins were detected using 240 antibodies. The selected proteins were analysed in 303 samples obtained from 157 DMD patients, which to our knowledge represents the largest longitudinal cohort of DMD patients described so far. Patients were followed up in three clinical centres (two in the United Kingdom and one in the Netherlands). Analysis of the data allowed the identification of MDH2 as a candidate prognostic and surrogate biomarker for DMD. MDH2 was negatively associated with age and was further decreased in non‐ambulant patients compared with ambulant ones, showing an association with disease progression. A time to event analysis clarified that a reduction of MDH2 in serum was further associated with an increased risk of wheelchair dependency. The stabilization of MDH2 levels after loss of ambulation may be due to reduced muscle damage, reduced metabolic demand in non‐ambulant patients, or insufficient assay sensitivity. The decreasing levels of MDH2 are confirmed in a larger study comprising a total of 493 DMD samples not only in serum but also in plasma (manuscript submitted to *Journal of Neuromuscular Diseases*). Interestingly, treatment with prednisone and deflazacort were associated with increased MDH2 levels. Because MDH2 is elevated in DMD compared with healthy individuals, elevation due to steroid use needs careful interpretation. Our data, however, suggest that treatment with steroids does not result in an elevation of MDH2 levels, instead steroids delay the decrease of MDH2 caused by disease progression. This hypothesis was formally tested significant in a post hoc comparison including the interaction between age and treatment, clarifying that effects on MDH2 are not caused by treatment alone but are the result of the combination of treatment and time. Given that MDH2 is linked to the capacity of mitochondria to produce reducing equivalents, it is possible that therapies aiming at dystrophin restoration and improving muscle quality and energy output may actually cause a sharp reduction of MDH2 in serum without implying a worsening of the phenotype. Indeed, one could then argue that therapies aiming to slow down disease progression, such as steroids, could result in slower decline of MDH2 over time, while therapies correcting the genetic defect may cause loss or reduction of the MDH2 signal due to improved muscle quality. This interpretation is also supported by the fact the MDH2 levels are lower in BMD compared with DMD patients.^16^ Careful consideration of the drug related expected effects is needed to correctly interpret the observed changes in prospective studies. Further validation is required to assess the validity of MDH2 as a surrogate endpoint for clinical trials,^37^ by prospectively assessing the capacity of MDH2 to capture clinical benefit.

A total of 30 proteins showed significant association with age. At least 21 of them were already known in the DMD field as proteins able to discriminate between DMD patients and heathy controls in other recent studies in patients' sera.^15^, ^16^, ^18^, ^21^, ^25^, ^26^, ^38^, ^39^ Less evidence was available for the other nine proteins, namely, NES, BASP1, C4A, MAP 4, C4BPA, CFH, KRT10, RELB, and PDZK1. Strong correlation with age in DMD patients had previously been described for six of these 30 proteins (CA3, MDH2, MYL3, ETFA, TNNT3, and CK)^16^; however, these proteins did not correlate with age in healthy controls, suggesting that these associations are disease specific and not shared by healthy individuals. Most of the proteins showing a negative association with age are known to have a role in muscle contraction (DES, TTN, TNNT2, TNNT3, and MYL3^40^), muscle function (CA3, ANKRD2, and MAP 4^16^, ^41^, ^42^), and energy production (CK, MDH2, ETFA, LDHB, and ENO3^40^, ^43^). The effect of lack of dystrophin on energy production and more specifically on mitochondria are supported by a body of literature showing effects on respiration activity, metabolic dysfunction, and macroautophagy.^44^, ^45^, ^46^, ^47^, ^48^ Interestingly, we see opposite and significant relationships with age of two enzymes using as substrate malate, namely, MDH2 and fumarate hydratase (FH). The level of MDH2 is reduced as disease progresses, while FH increases. While it seems plausible that the reduction of MDH2 is linked to reduced mitochondrial capacity, the increase in FH may be related to another mechanism. It has been recently demonstrated that the role of FH is not only to produce reducing equivalents as part of the TCA cycle, but it also plays a role in DNA damage response^49^; recently, telomeres length and DNA damage response in non‐dividing cardiomyocytes have been connected to cardiomyopathy, thus suggesting that FH serum level could be associated to cardiac involvement in DMD.^50^ Another protein increasing with age was RELB, which is a member of the alternative NF‐κB complex and is known to promote mitochondrial biogenesis and transition from a glycolytic towards an oxidative metabolism in muscle fibres.^51^ The increase in RELB with age could perhaps mirror the shift from fast to slow twitch fibres observed in DMD.^52^ The increase of RELB could also be linked to reduced differentiation capacity of muscle in older patients, as NF‐κB activation by cytokines has been described to induce satellite cells proliferation and to negatively affect late differentiation.^53^ However, it is important to point out that the overall MFI of the FH, and especially GSN and RELB antibodies, was low, implying that abundance variation over time might be difficult to detect in the current assay. The identified associations of these markers with age would need to be validated using independent, quantitative methods, such as ELISA and MS. Four proteins of the complement cascade showed a positive association with age (CFH, C3, C4BPA, and C4A). This increase may be related to necrosis, as complement membrane attack complexes have been shown to specifically detect necrotic fibres and endomysial capillaries in muscular dystrophies as in inflammatory myopathies.^54^, ^55^ All three antibodies against C4A show positive association with age, but the correlation between the antibodies ranges between 0.39 and 0.77. Because the homology between the C4A and C4B is higher than 97%, the three antibodies would most likely recognize both proteins but with different specificities. The increase in complement factors and RELB together could also point to the inflammatory component of the disease as they have been reported as histopathological signs of myositis.^56^ Tissue damage, complement deposition, and increased calcium levels represent calcification triggers, which seem to be counteracted by expression of matrix Gla protein (MGP), which we found to be increasing with age in DMD patients. Other less obvious negative associations with age involve NES (primarily expressed in nerve cells but also in satellite cells and pericytes^57^, ^58^), HDAC2 (known to bind the dystrophin partner nNOS^59^), LCP1 (actin binder specific of the cells of the hematopoietic lineage^60^), and COL1A1 (related to fibrosis^61^ and bone disease^62^). The association of COL1A1 with both age and steroid treatment is especially interesting, as claims over different bone‐related side effects of prednisone and deflazacort have been made.^63^ Collagen 1 was negatively associated with age and further reduced by treatment with steroids. Comparison of deflazacort‐treated and prednisone‐treated patients showed reduced COL1A1 levels in deflazacort‐treated patients compared with prednisone treated ones. Interestingly, a recent report shows that treatment with deflazacort was associated with a higher number of vertebral fractures, shorter time to first fracture, and higher linear growth failure.^64^ Results of ongoing studies such as the FOR‐DMD study^65^ will potentially clarify the effect of these drugs and regimes on bone health and whether events are associated with or predicted by COL1A1 levels in blood.

The study described in this paper has been performed using a multiplexed immunoassay with monospecific polyclonal antibodies. Although all antibodies in this study have been developed using a standardized pipeline for validating antibody specificity, these findings remain to be validated using non‐antibody‐based methods and clinical chemistry grade assays in order to support the inclusion of these markers in clinical practice. This limitation is evident in the discrepancies across antibodies which may pinpoint to limitation of the assay, as well as real biological variation due to the presence of specific protein fragments in circulation. This is particularly important for MDH2 as different antibodies show unequal performance. In this case, the anti‐MDH2 antibody found to correlate with age also had a high pair‐wise correlation with other potential DMD biomarkers which had a clear age‐dependent decline, such as CA3, TNNT3, CKb, MYL3, and ETFA (File S3), while showing very little similarities with the rest of the assay. Antibodies towards C4A, C4BPA, and MGP, which were all found to increase with age, were also found to have a high pair‐wise correlation to one another and low or no correlation with the rest of the assay. Another limitation is the retrospective nature of the study with unphased and widely distributed samplings, which do not resemble the typical structure observed in clinical trials. Future studies should aim to evaluate the performance of the identified biomarkers in more controlled settings such as in samples obtained from clinical trials as well as to compare and/or combine their performance with readiological outcomes such as muscle fat fraction.

To summarize, we identified a number of serum protein profiles associated with disease progression and disease milestones, such as loss of ambulation and treatment with corticosteroids. Among the identified biomarkers, MDH2 seems to have sufficient characteristics to be included prospectively in clinical studies to test the prognostic potential and the ability to anticipate clinical benefit.

## Conflict of interest

The authors declare that they have no conflict of interest related to the work described.

## Supporting information

Data S1 Supporting information

Table S1 List of targets considered in the analysis.

Table S2 Test for differential expression across hospitals.

Table S3 Linear mixed models for MDH2, COL1A1 and ANKRD2 containing interaction terms between age and treatment with glucocorticosteroids.

File S1 Comparison of fluorescence intensity for proteins measured through different antibodies.

File S2 Trajectory plots for all proteins significantly associated with age.

File S3 Heatmap with pairwise Pearson's correlation coefficients between all antibodies throughout assay
