# Supplementary material for: Longitudinal serum biomarker screening identifies malate dehydrogenase 2 as candidate prognostic biomarker for Duchenne muscular dystrophy
Source: J Cachexia Sarcopenia Muscle. 2019 Dec 27;11(2):505–17. doi: 10.1002/jcsm.12517 (PMC7113516; doi:10.1002/jcsm.12517)
Supplement: Supplementary file 1 — Data S1 Supporting information [file JCSM-11-505-s007.docx]

**SUPPLEMENTARY INFORMATION**

**Supplementary Table 1**

**List of targets considered in the analysis.**

The table lists the 240 targets considered in the study.

**Supplementary Table 2**

**Test for differential expression across hospitals.**

The table illustrates the results of an F test that verifies the equality of protein expression levels across hospitals. The columns beta_UCL and beta_UNEW contain the parameter estimates of the regression coefficients for UCL and UNEW (reference mode is LUMC), p_ftest is the p-value of the F test and FDR the p-value after application of the Benjamini-Hochberg multiple testing correction.

**Supplementary Table 3**

**Linear mixed models for MDH2, COL1A1 and ANKRD2 containing interaction terms between age and treatment with glucocorticosteroids.**

To clarify the interplay between age and corticosteroids, we included in the linear mixed model the interaction between age and treatment for MDH2, COL1A1 and ANKRD2. For each protein we report the p-value of the F test on the two interaction terms, followed by a table with the maximum likelihood estimates of the regression coefficients, the estimated standard errors and the Wald test p-values. The interaction was found to be significant at 5% level for MDH2 (p = 0.009) and COL1A1 (p = 0.006). Both prednisone and deflazacort were found to attenuate the decrease of MDH2 with age. Only deflazacort, instead, was found to further reduce the levels of COL1A1 with age.

**Supplementary File 1**

**Comparison of fluorescence intensity for proteins measured through different antibodies.**

81 of the proteins considered in this study were measured through more than one antibody. For each of these proteins, we provide a scatterplot matrix that compares the median fluorescence intensity of the different antibodies used to measure it. The upper diagonal contains a scatterplot for each pair of antibodies, whereas the lower diagonal reports the value of Pearson’s correlation coefficient.

**Supplementary File 2**

**Trajectory plots for all proteins significantly associated with age.**

The file contains trajectory plots showing the dynamic evolution of protein levels for each subject considered in this study. Proteins plotted in red are those whose levels significantly increase with age, whereas those in blue are those that significantly decrease.

**Supplementary File 3**

**Heatmap with pairwise Pearson’s correlation coefficients between all antibodies throughout assay**

256 Luminex MagPlex beads were coupled to 248 target-specific antibodies, 5 positive control antibodies and 3 negative controls. A heatmap was constructed from Pearson’s correlation coefficients between log-MFI to simultaneously check for pairwise correlations between both antibodies towards the same target, antibodies towards different targets and correlations towards positive and negative controls. The heatmap showed that the 5 positive controls (together with all antibodies towards SERPINA1) correlated with each other but with no or very little similarities to the rest of the assay. Another distinct cluster included antibodies found to correlate negatively with age in the linear mixed model, as well as a third cluster with C4A, CFH and MGP, which were found to correlate positively with age in the linear mixed model.
