# Supplementary material for: Longitudinal serum biomarker screening identifies malate dehydrogenase 2 as candidate prognostic biomarker for Duchenne muscular dystrophy
Source: J Cachexia Sarcopenia Muscle. 2019 Dec 27;11(2):505–17. doi: 10.1002/jcsm.12517 (PMC7113516; doi:10.1002/jcsm.12517)

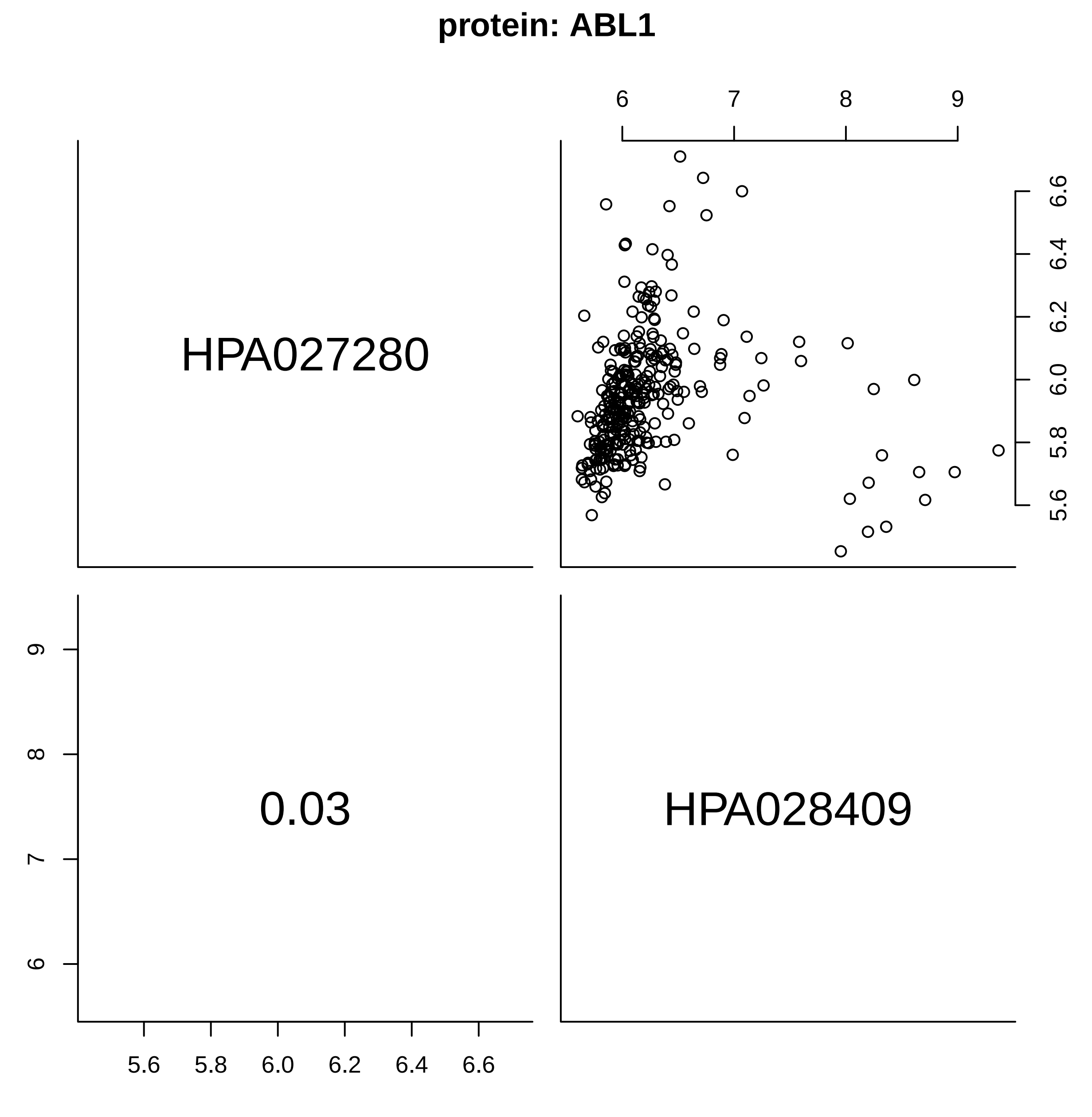
protein: ABL1
HPA027280
0.03
HPA028409
6
7
8
9
5.6
5.8
6.0
6.2
6.4
6.6

**protein: ACTA2**

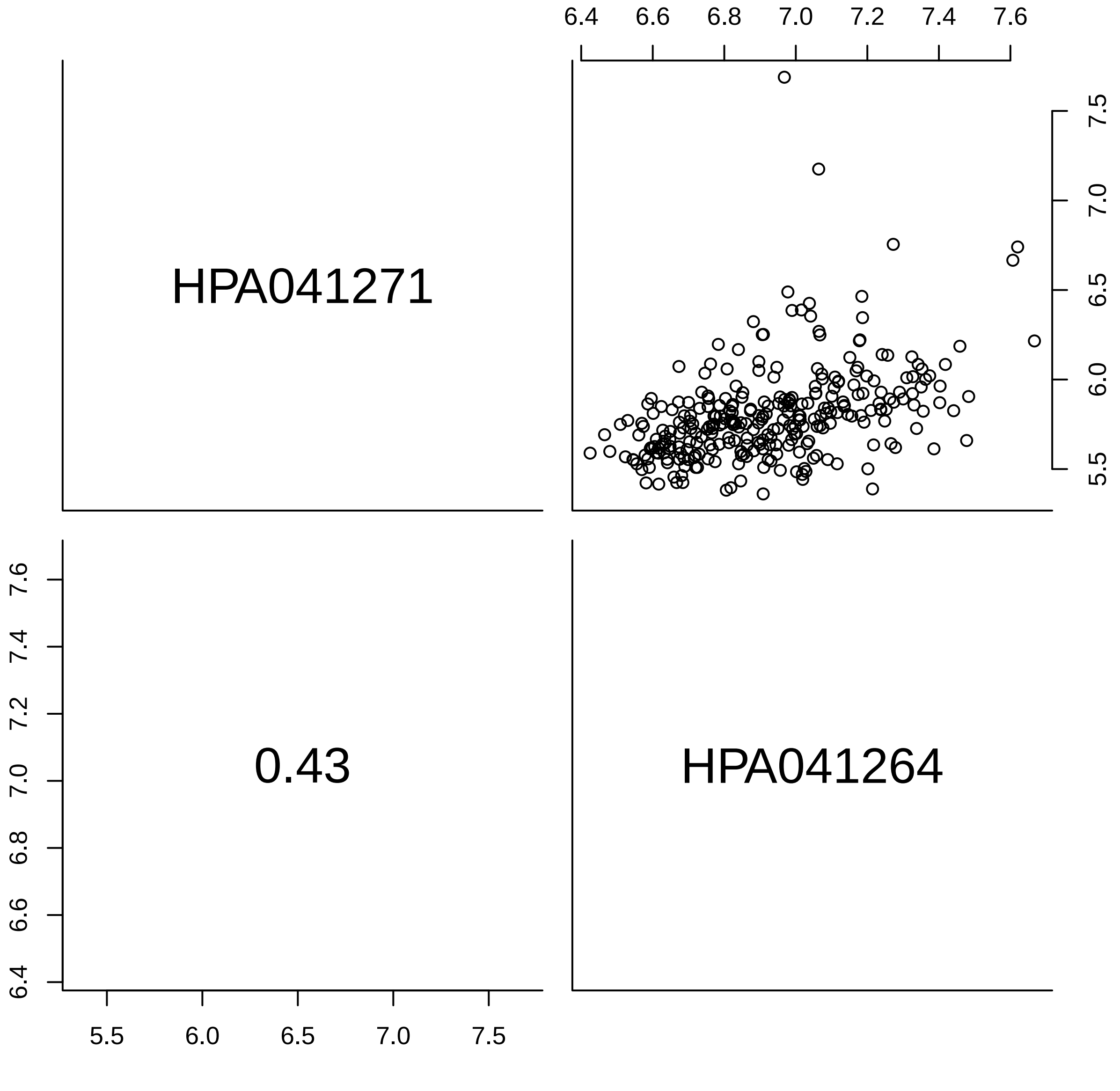

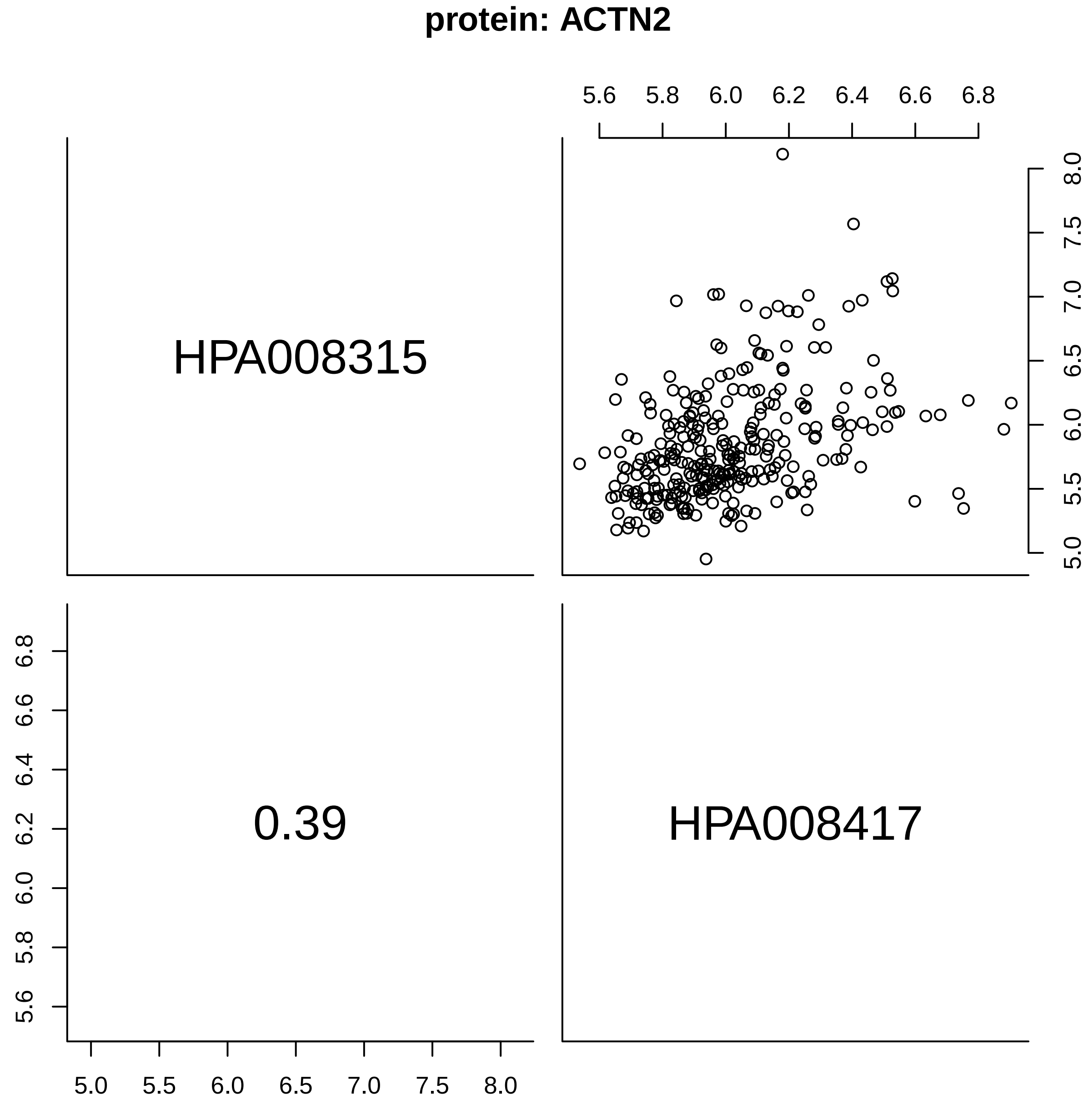
protein: ACTN2
HPA008315
HPA008417
0.39
5.6
5.8
6.0
6.2
6.4
6.6
6.8
5.0
5.5
6.0
6.5
7.0
7.5
8.0

# protein: AKAP1

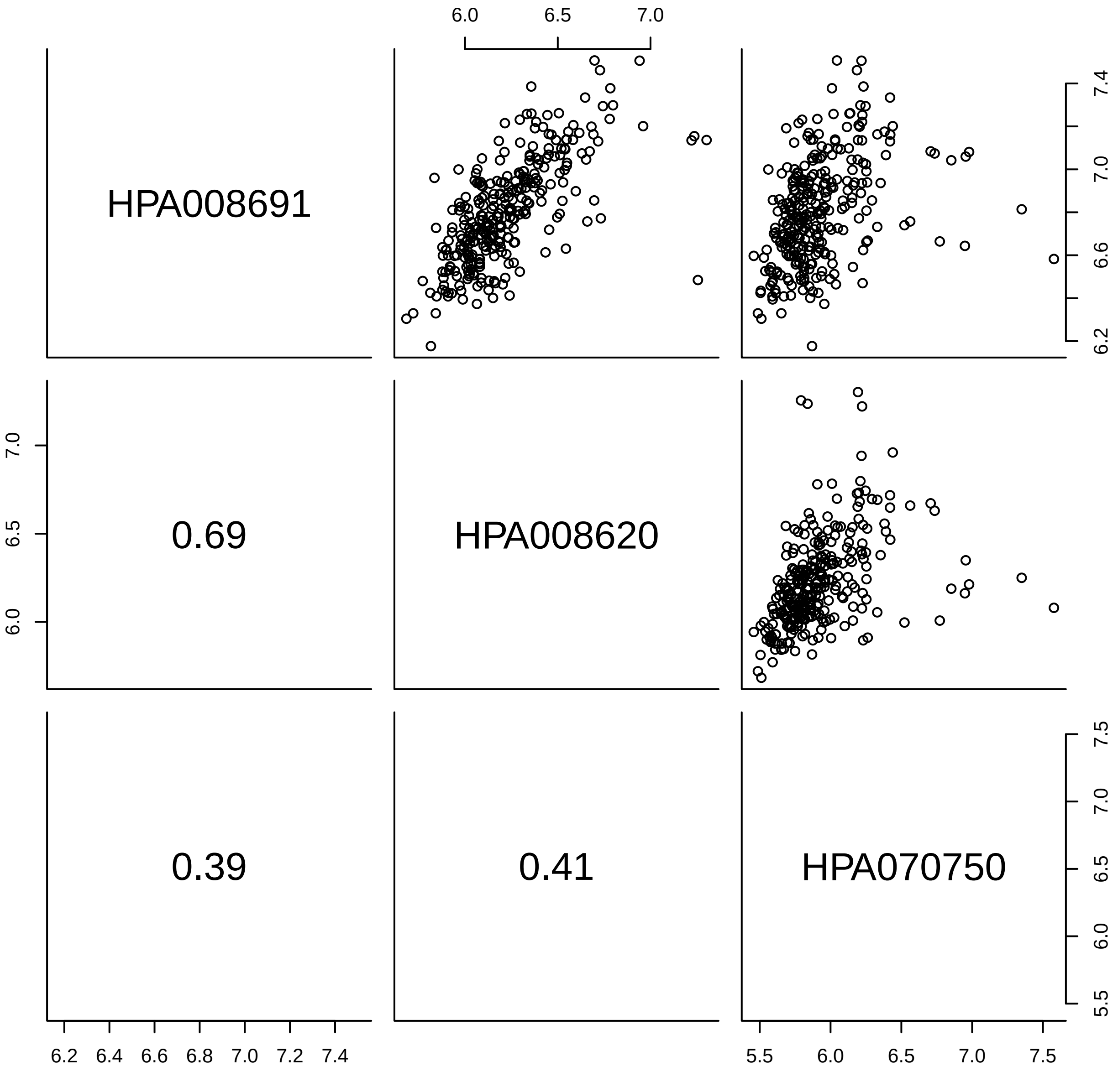

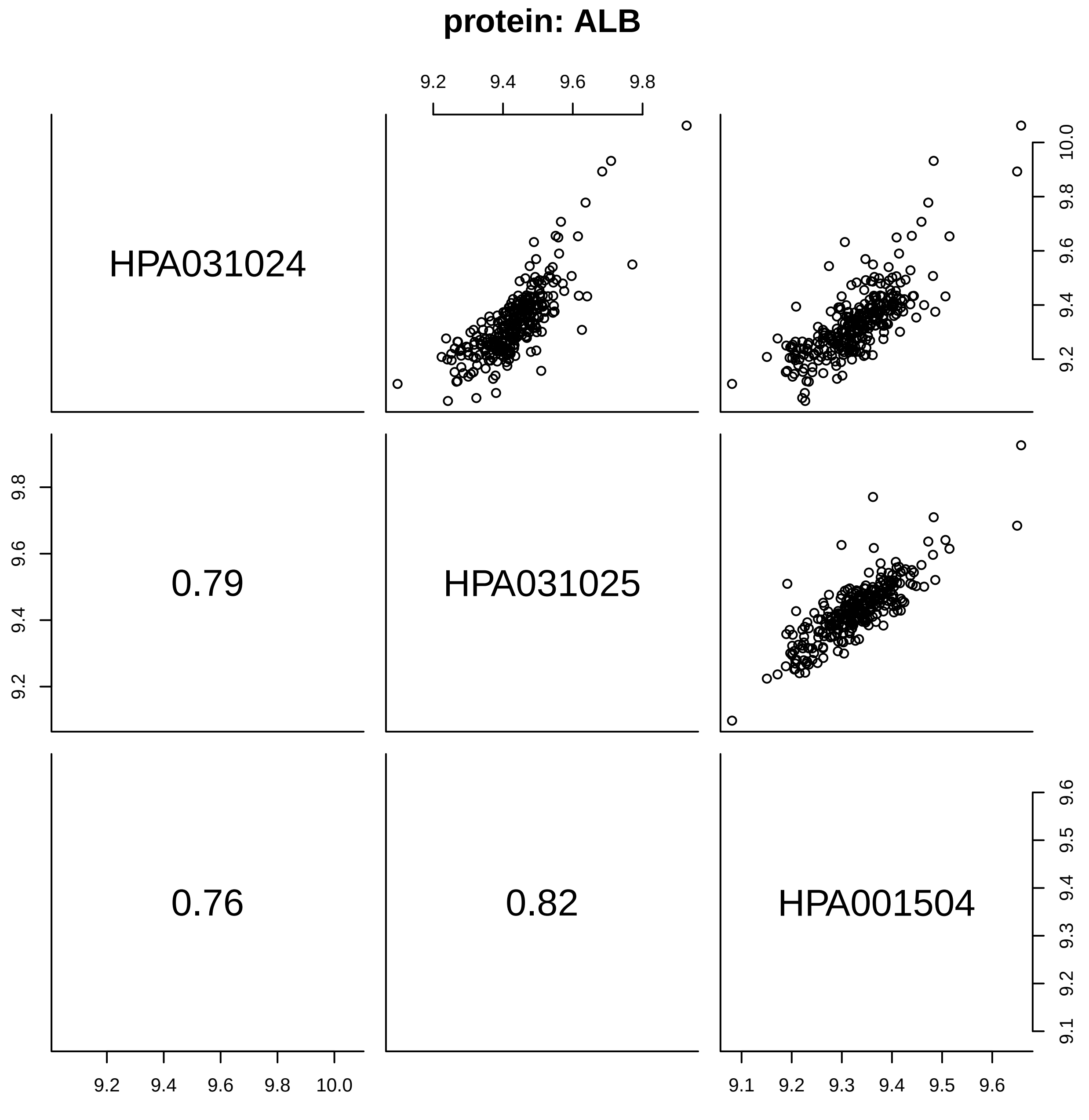
protein: ALB
HPA031024
HPA031025
HPA001504
0.79
0.76
0.82
9.2
9.4
9.6
9.8
10.0
9.1
9.3
9.5

**protein: ANKRD2**

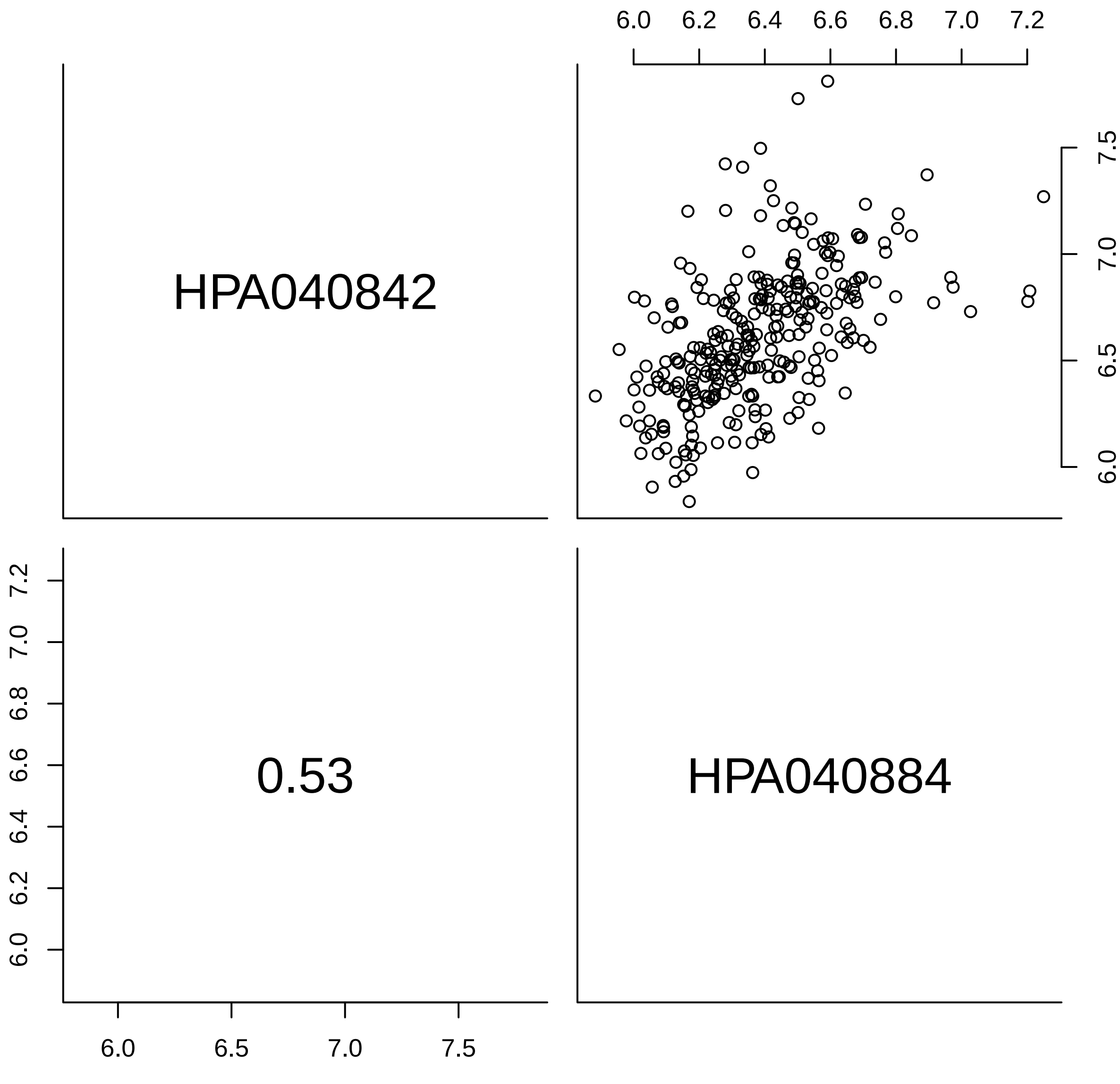

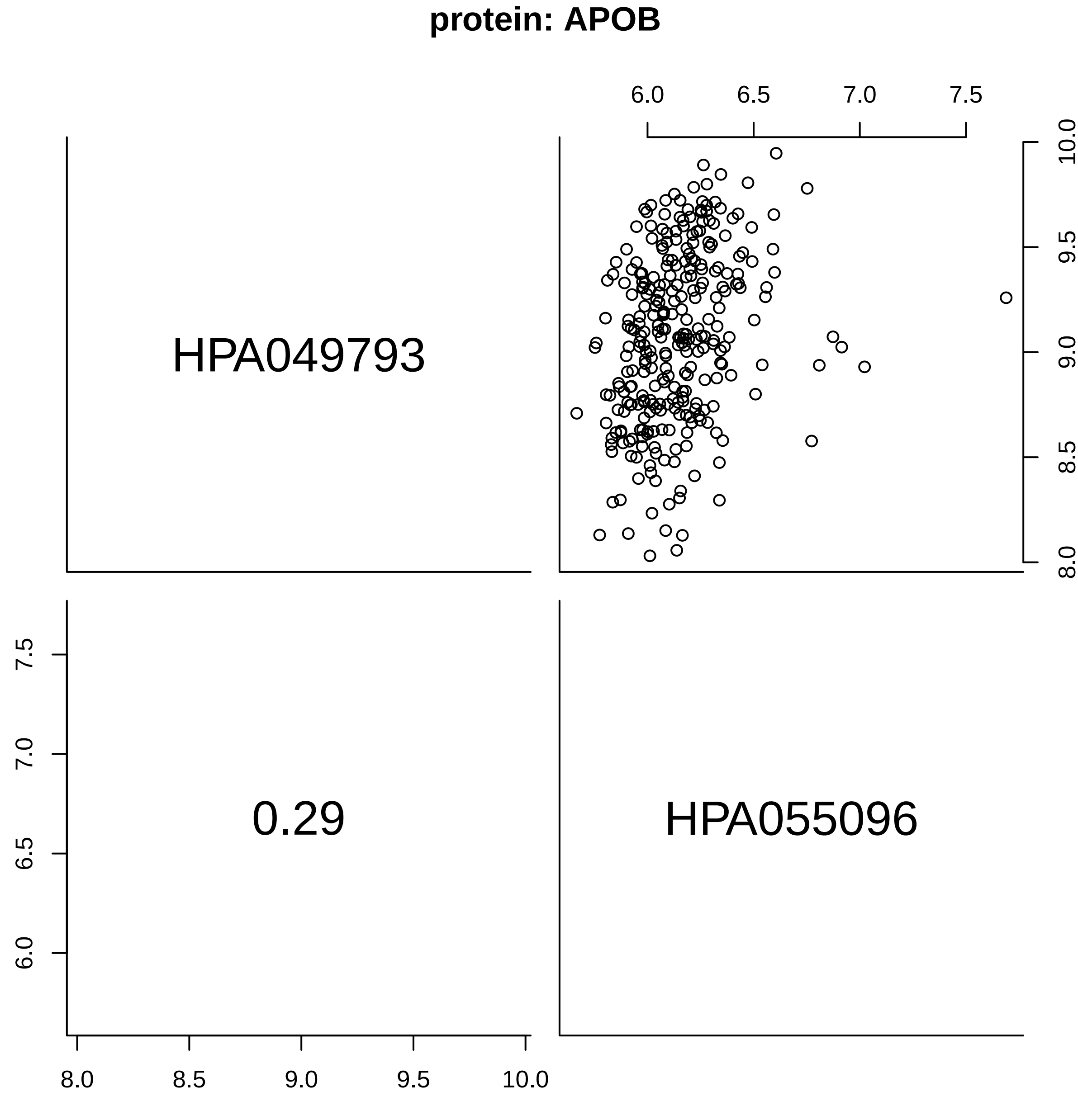

protein: APOB
HPA049793
0.29
HPA055096
6.0
6.5
7.0
7.5
10.0
9.5
9.0
8.5
8.0

## protein: APOE

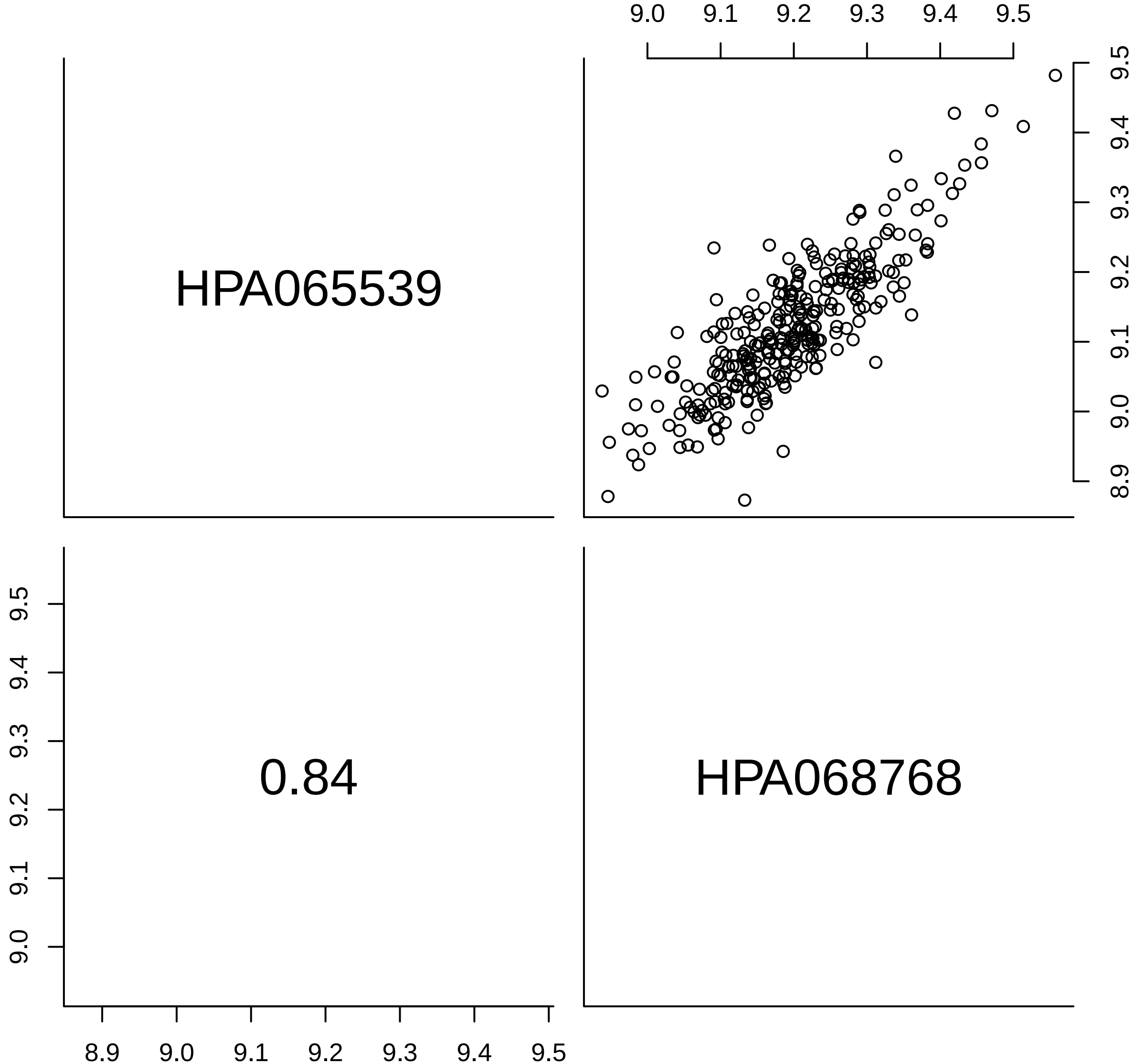

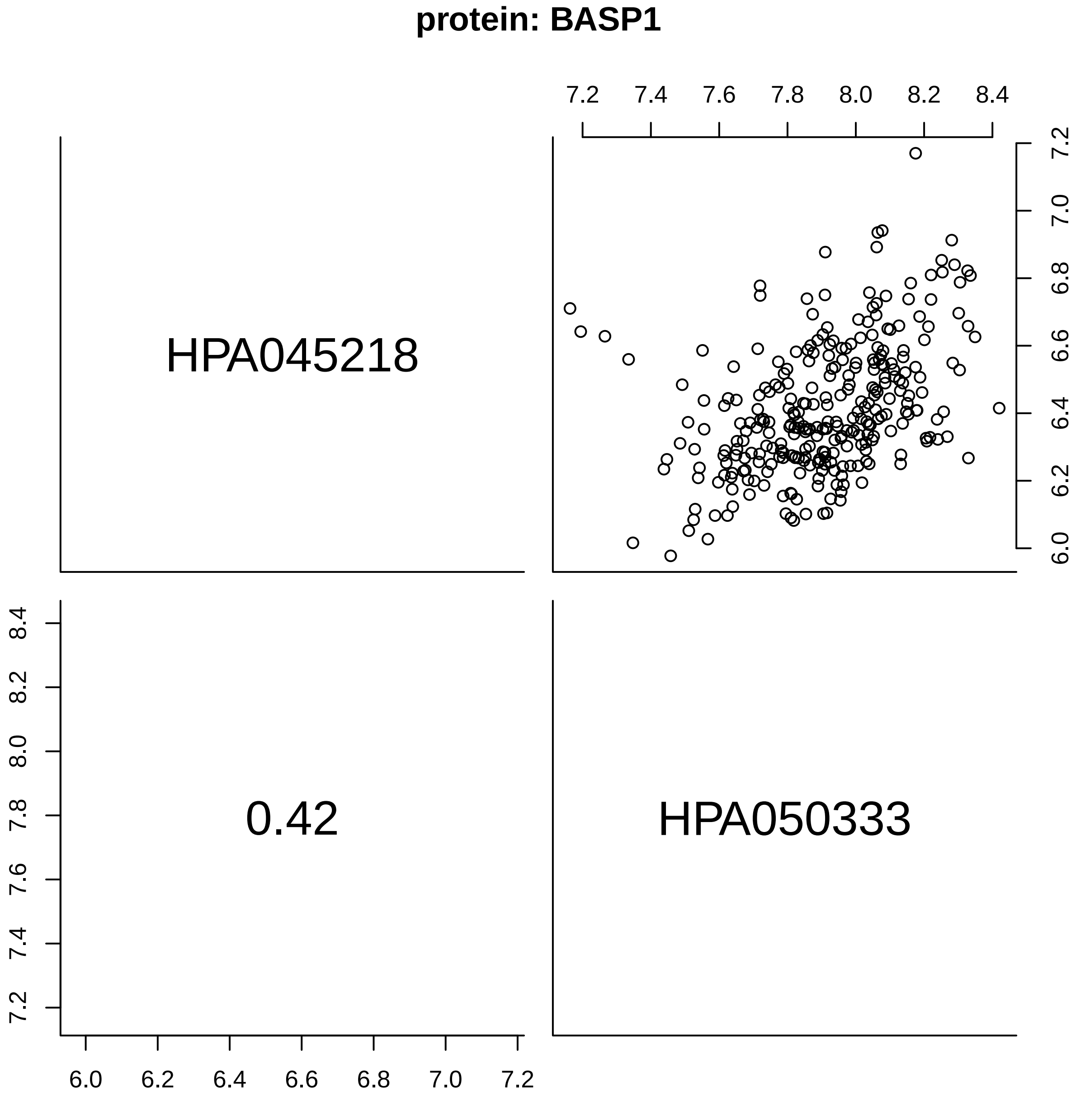
protein: BASP1
HPA045218
HPA050333
0.42
7.2
7.4
7.6
7.8
8.0
8.2
8.4
6.0
6.2
6.4
6.6
6.8
7.0
7.2

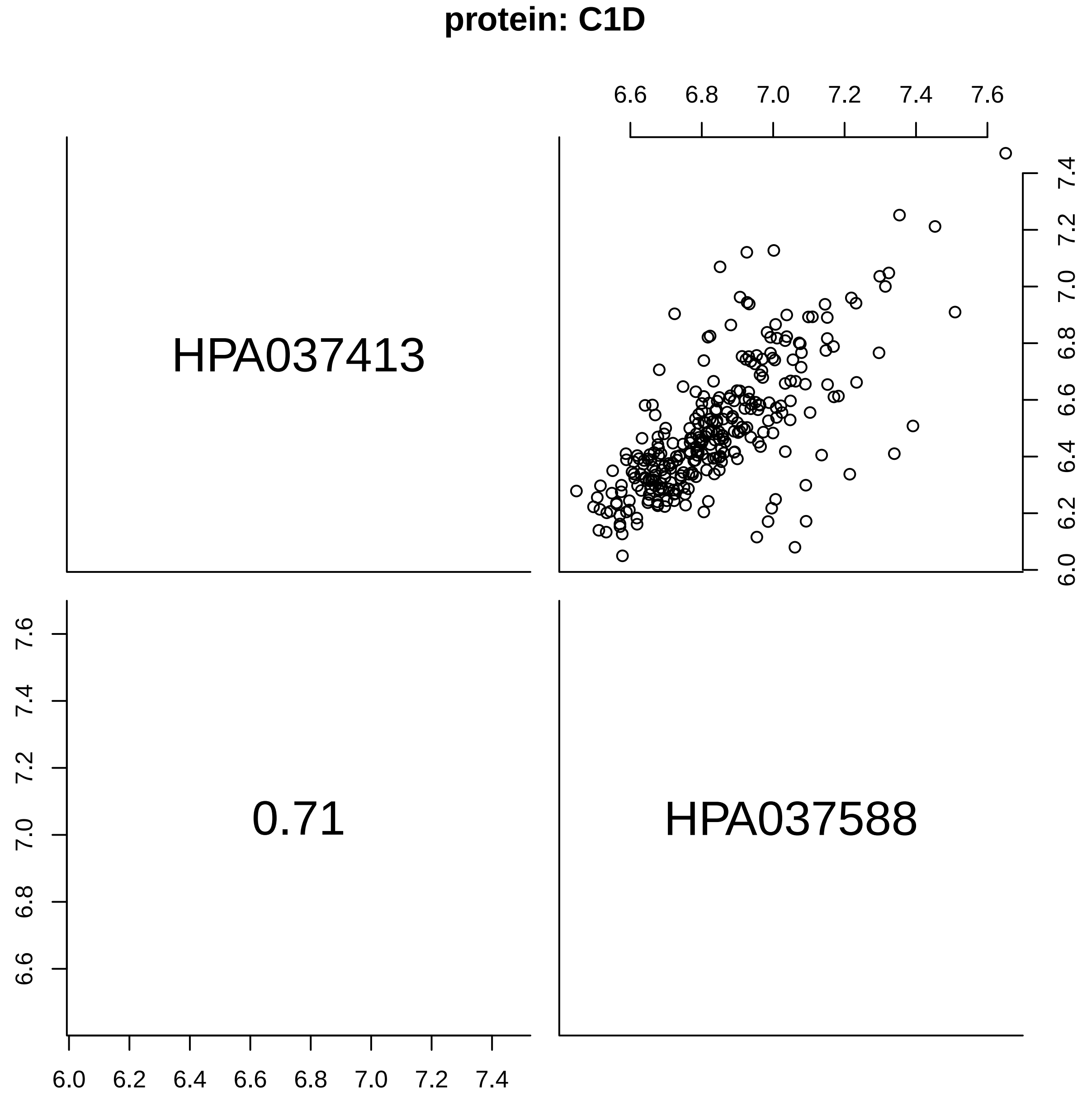

protein: C1D
HPA037413
0.71
HPA037588
6.6
6.8
7.0
7.2
7.4
7.6
6.0
6.2
6.4
6.6
6.8
7.0
7.2
7.4

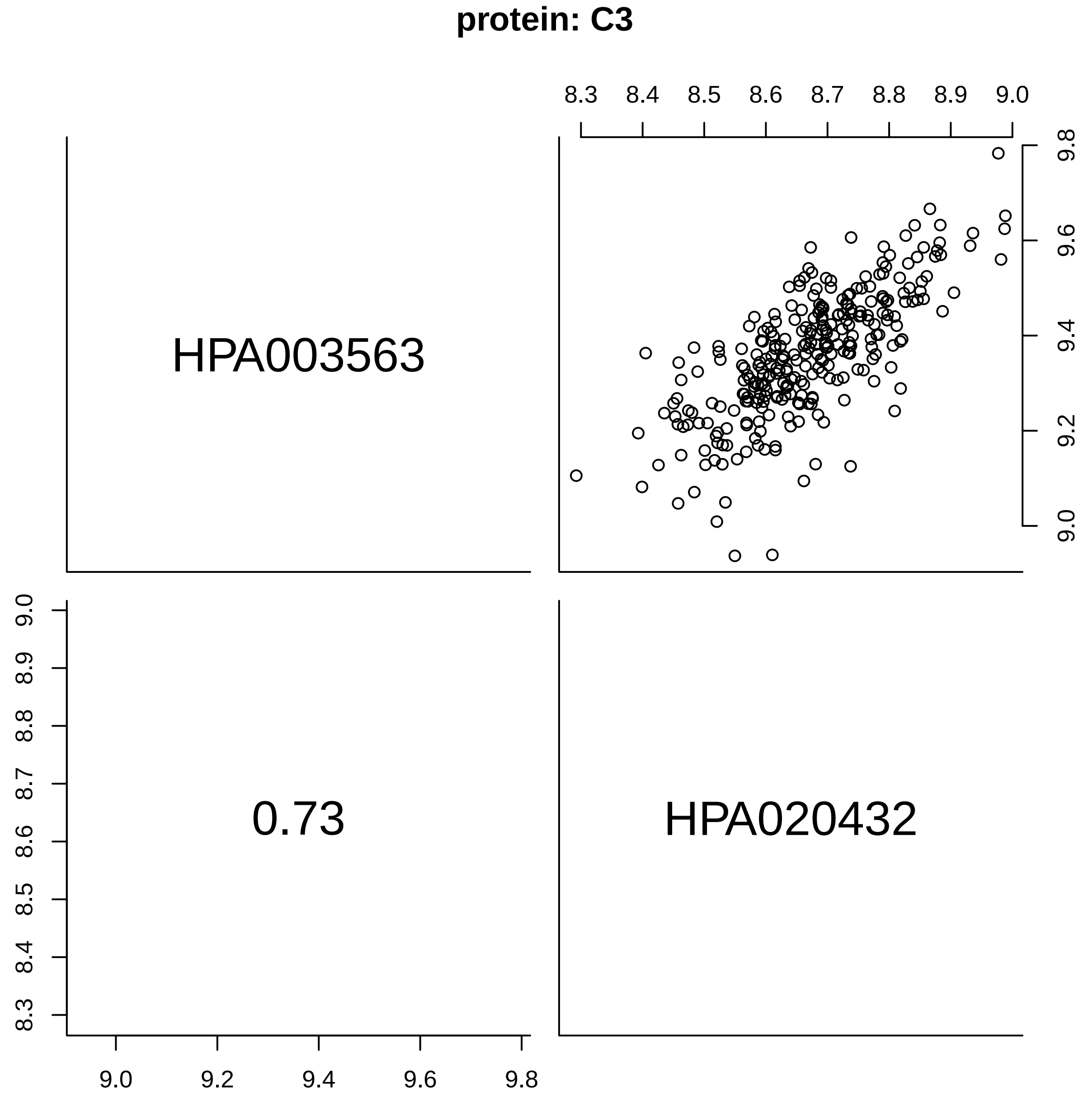

protein: C3
HPA003563
0.73
HPA020432
8.3 8.4 8.5 8.6 8.7 8.8 8.9 9.0
9.0 9.2 9.4 9.6 9.8

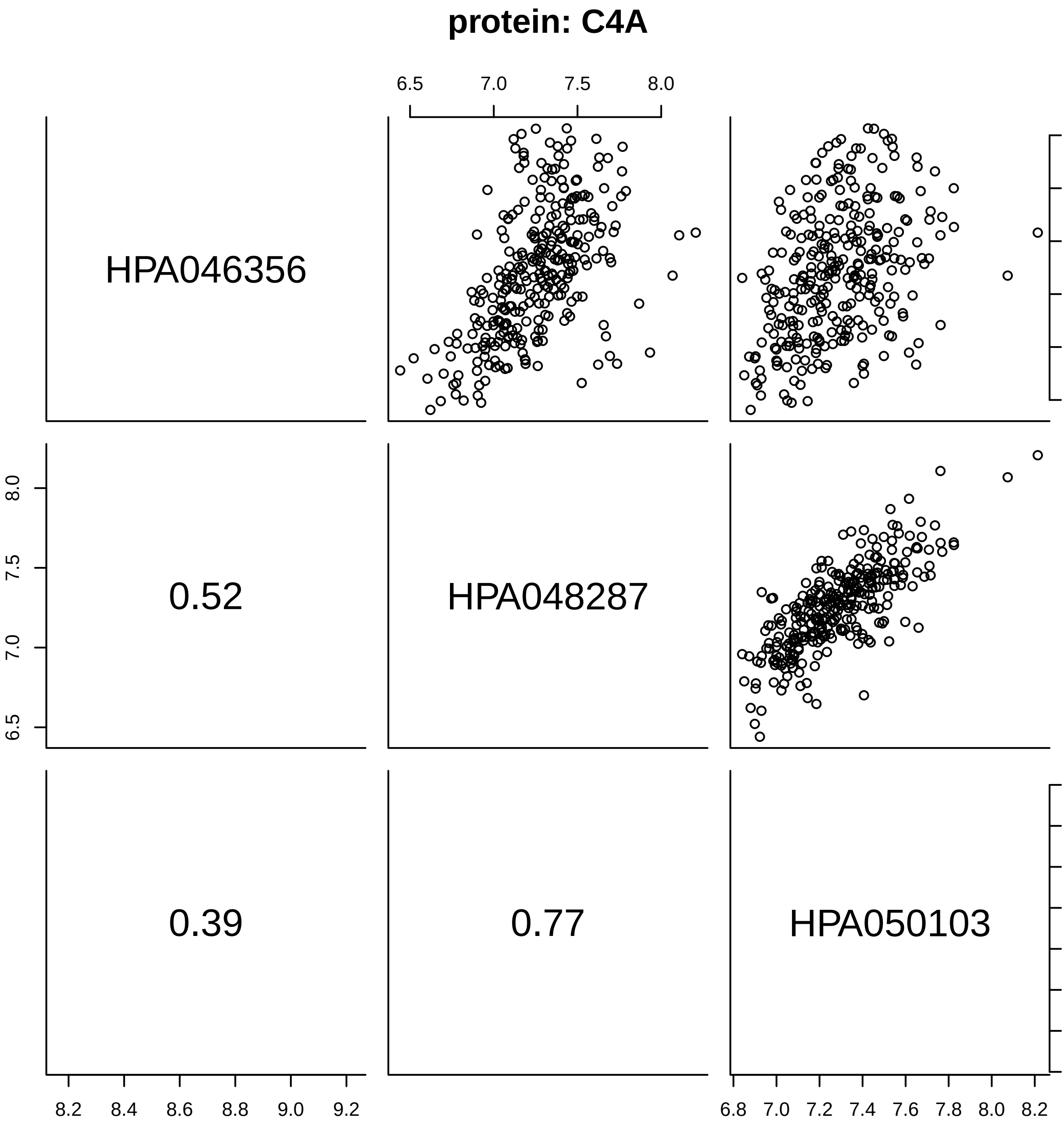
protein: C4A
HPA046356
HPA048287
HPA050103
0.52
0.39
0.77
6.5
7.0
7.5
8.0
8.2
8.4
8.6
8.8
9.0
9.2
6.8
7.2
7.6

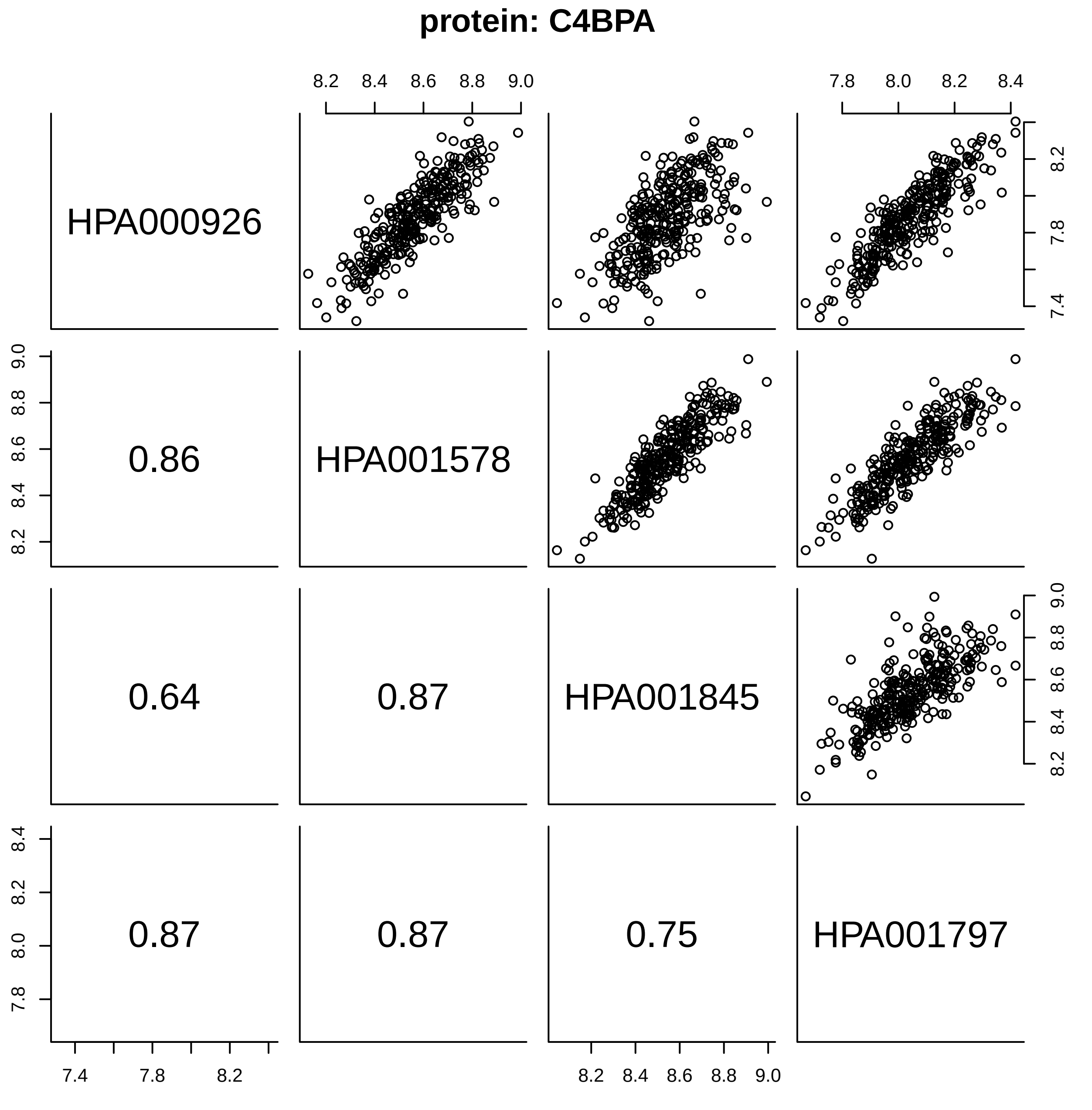

protein: C4BPA
HPA000926
HPA001578
HPA001845
HPA001797
0.86
0.64
0.87
0.87
0.87
0.75
8.2 8.4 8.6 8.8 9.0
7.8 8.0 8.2 8.4
7.4 7.8 8.2

## protein: C5

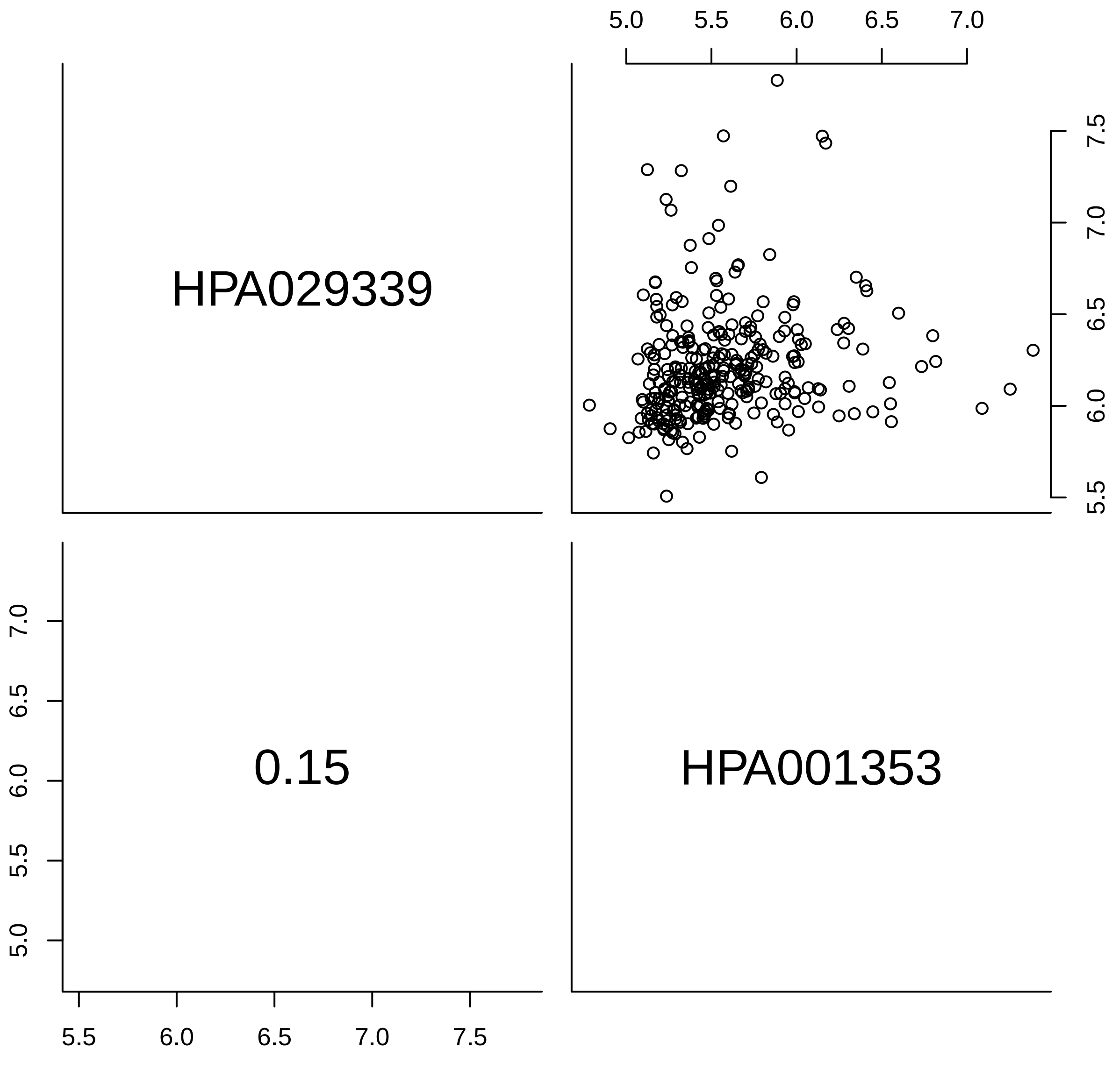

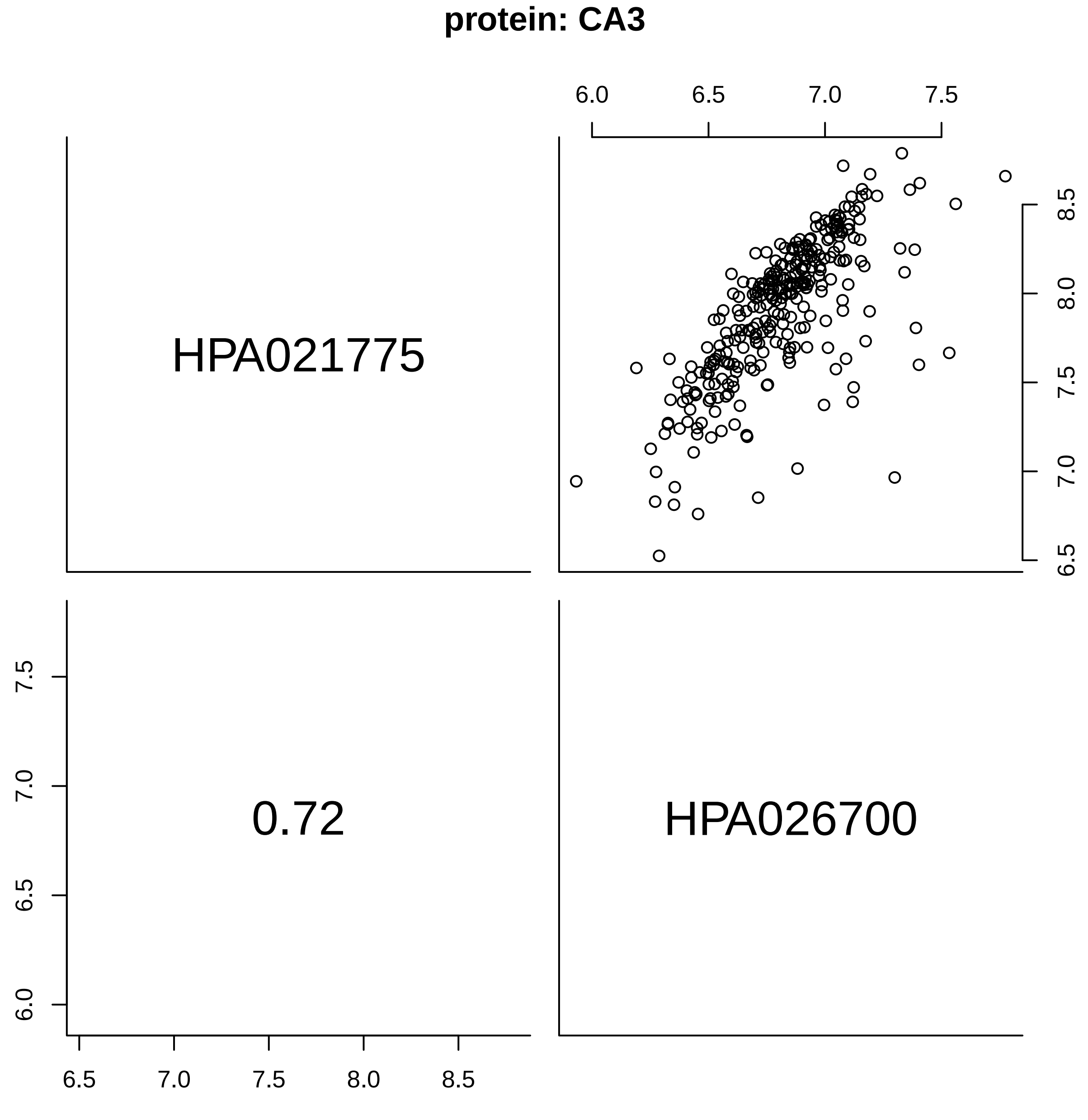

protein: CA3
HPA021775
0.72
HPA026700
6.0
6.5
7.0
7.5
8.0
8.5

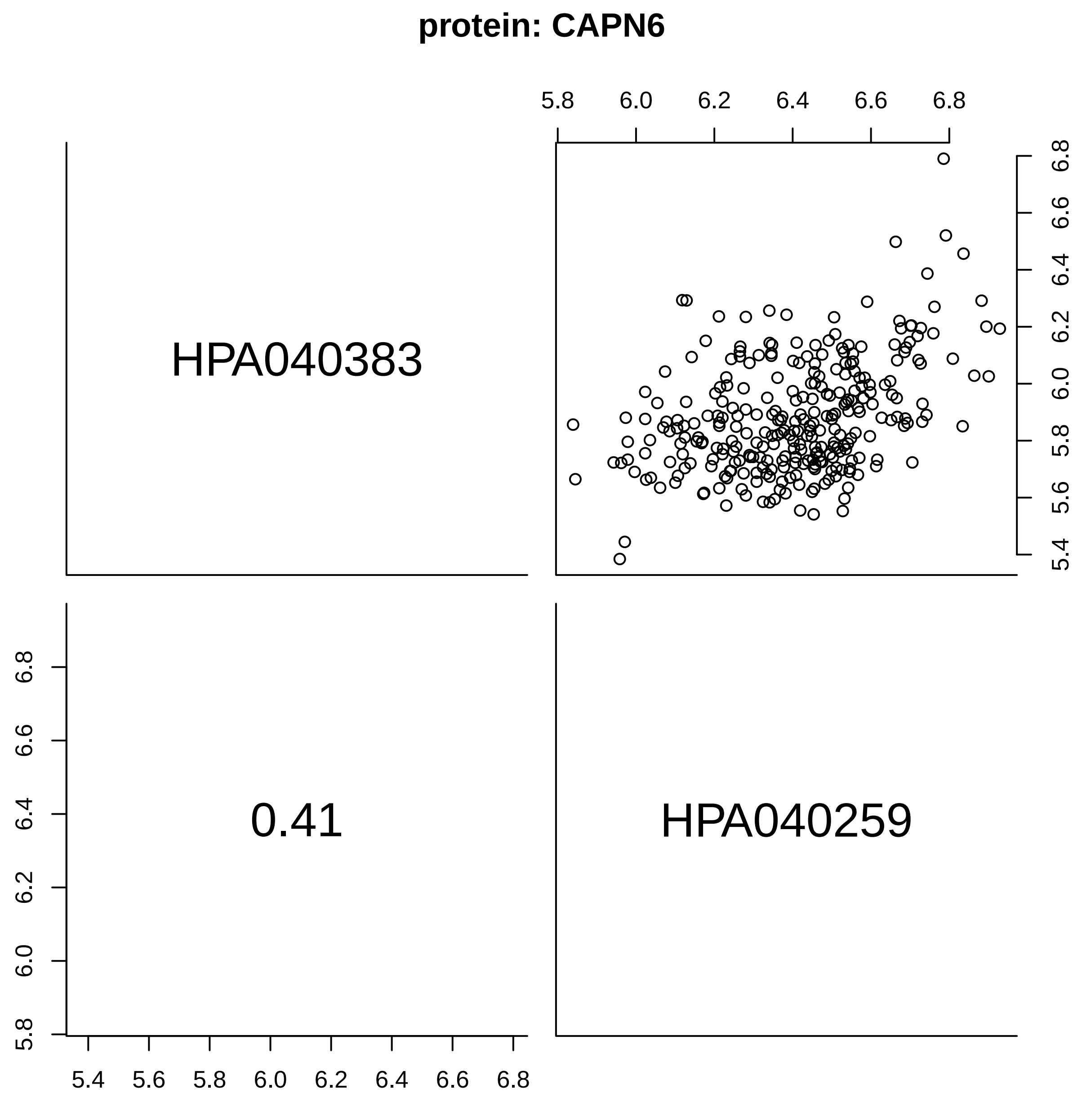
protein: CAPN6
HPA040383
0.41
HPA040259
5.8
6.0
6.2
6.4
6.6
6.8
5.4
5.6

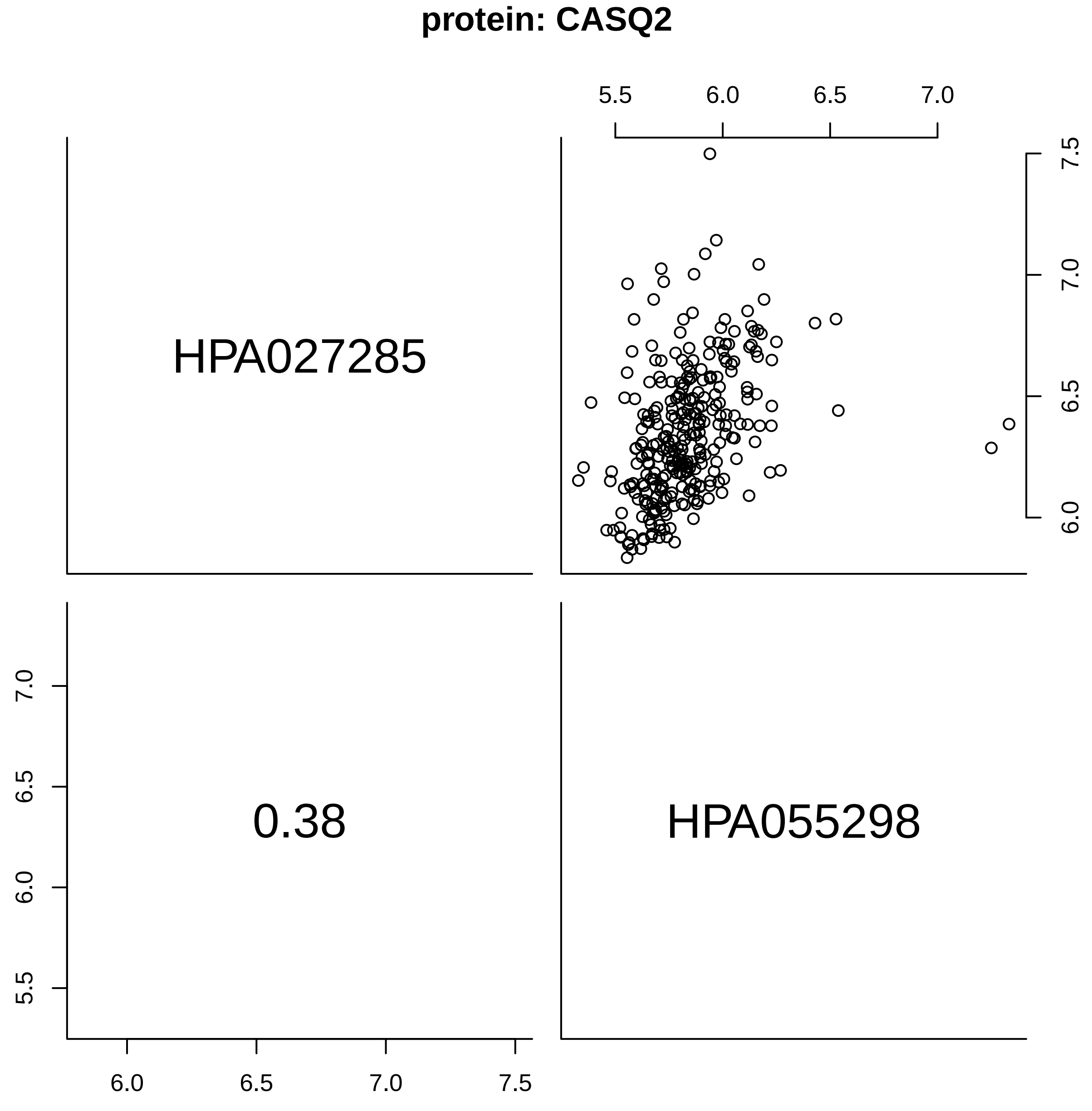
protein: CASQ2
HPA027285
HPA055298
0.38
5.5
6.0
6.5
7.0
7.5

## protein: CCNB1

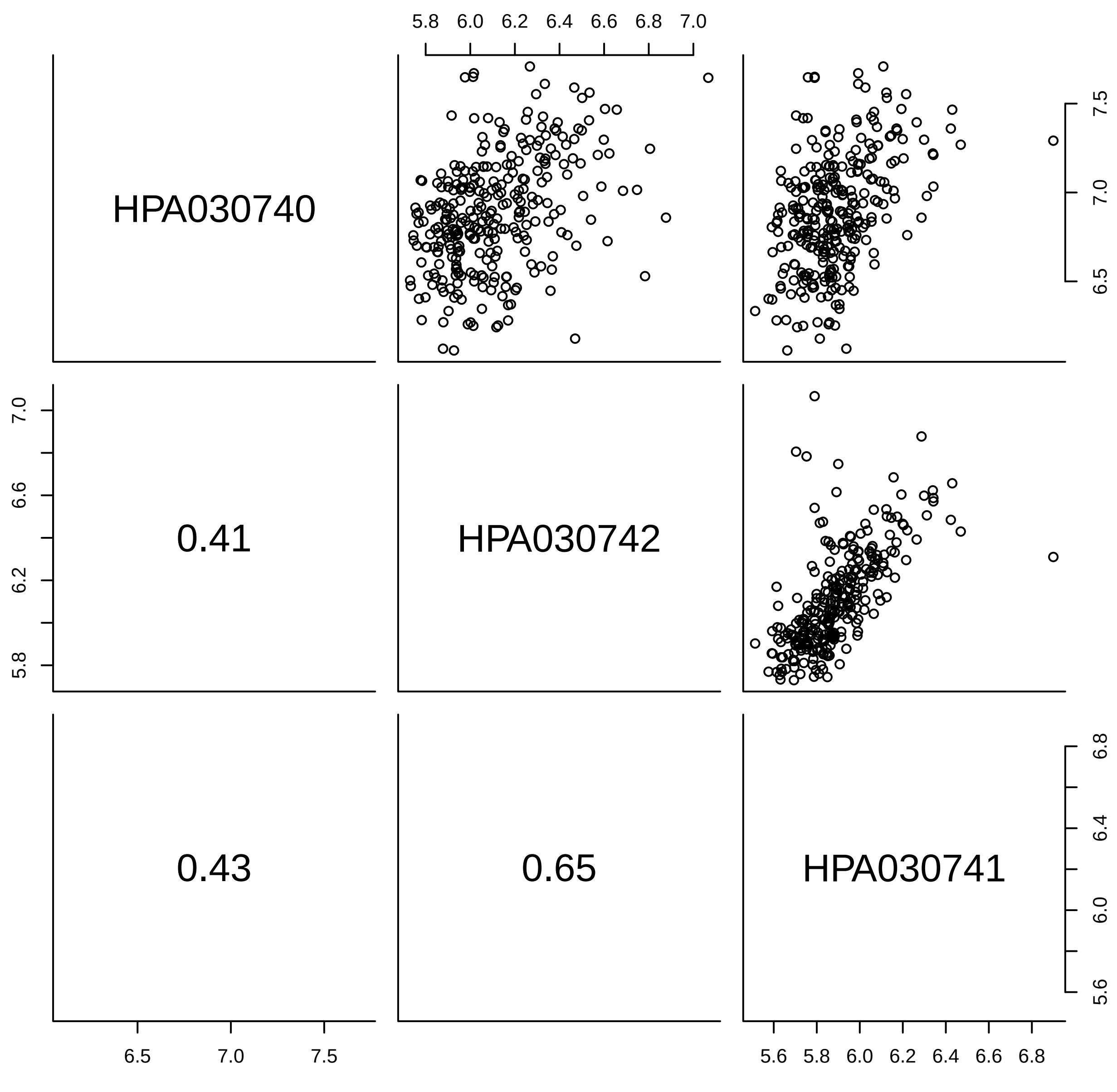

# protein: CDK14

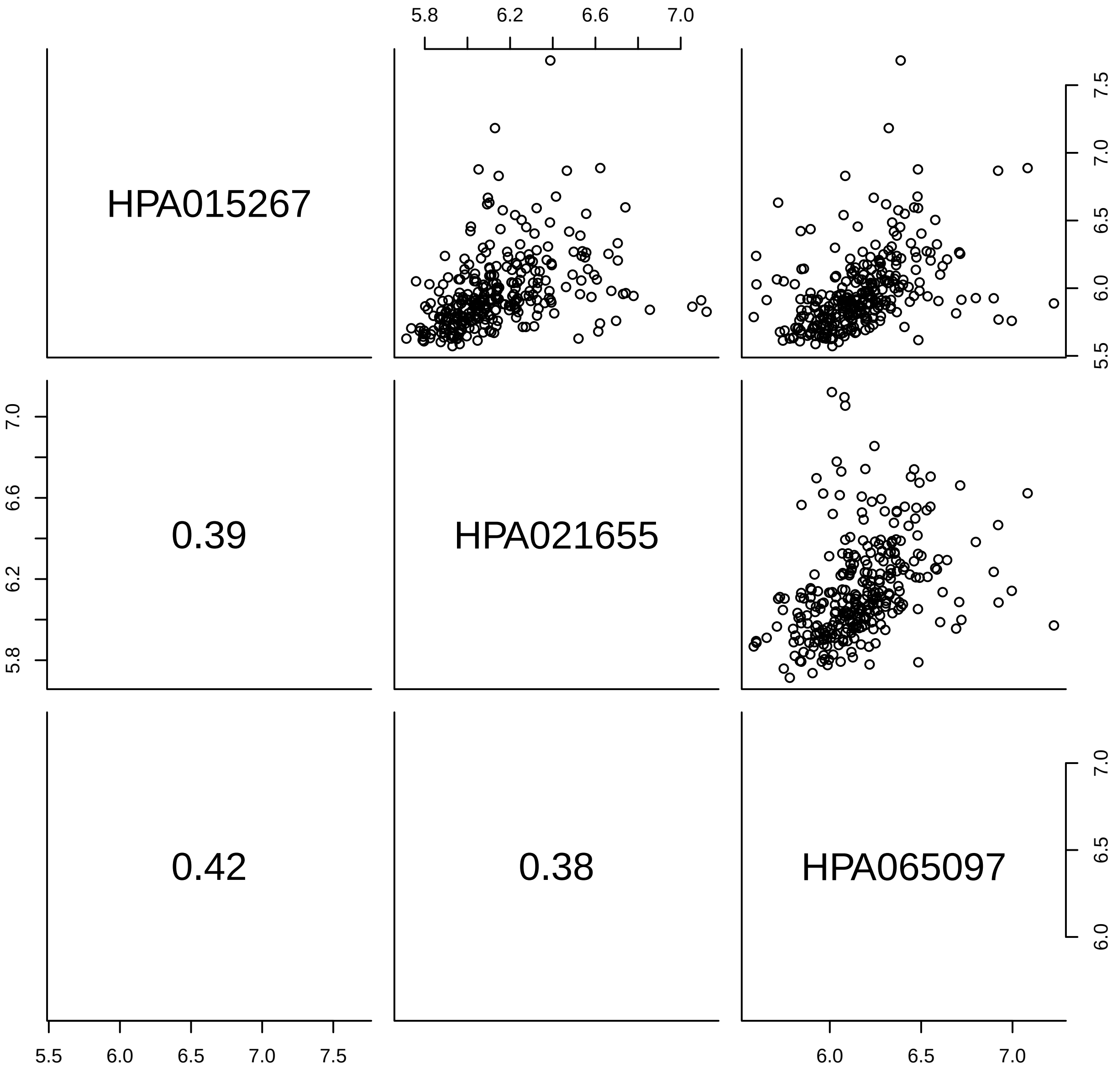

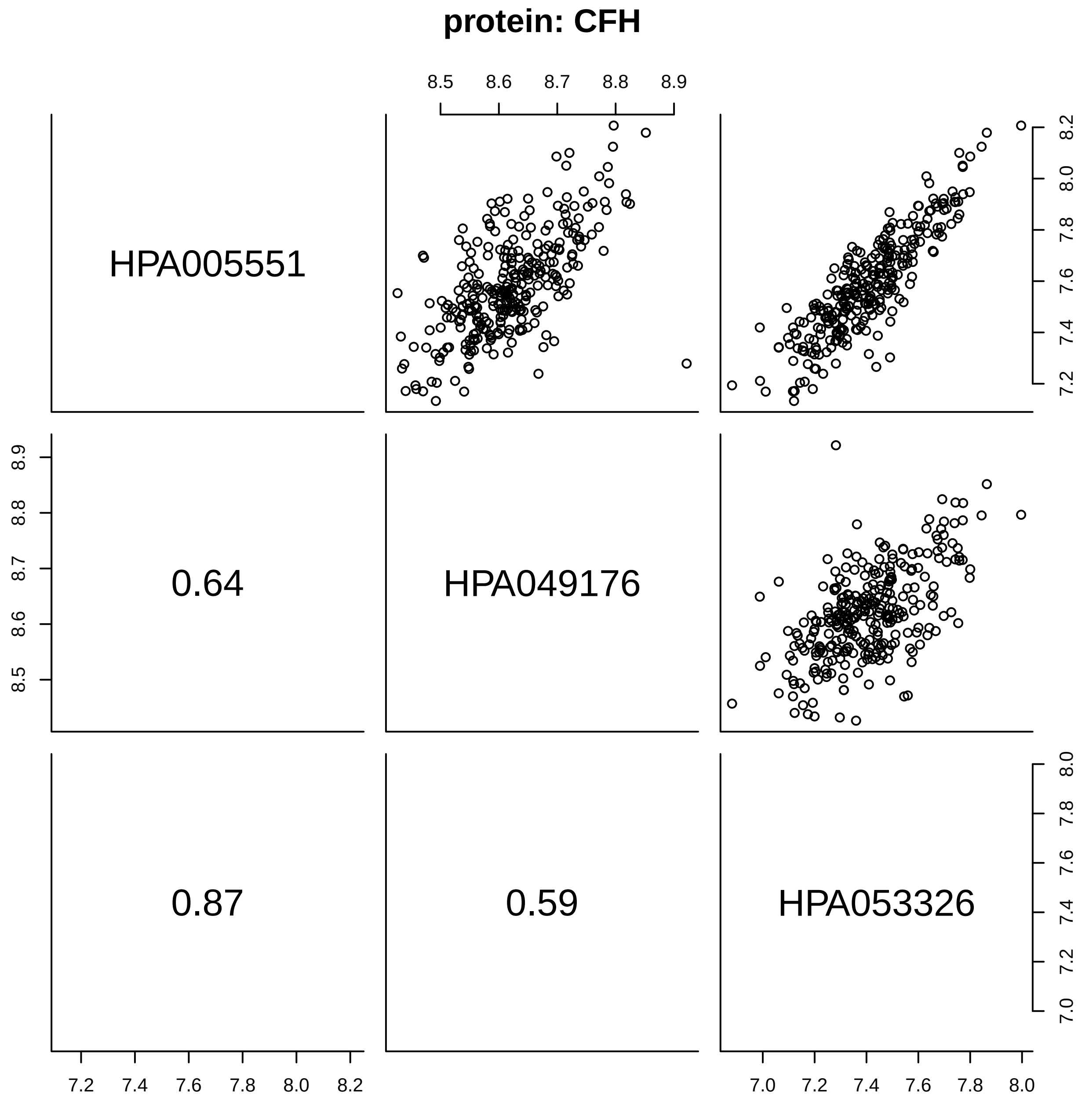

protein: CFH
HPA005551
HPA049176
HPA053326
0.64
0.87
0.59
8.5 8.6 8.7 8.8 8.9
7.2 7.4 7.6 7.8 8.0 8.2
7.0 7.2 7.4 7.6 7.8 8.0

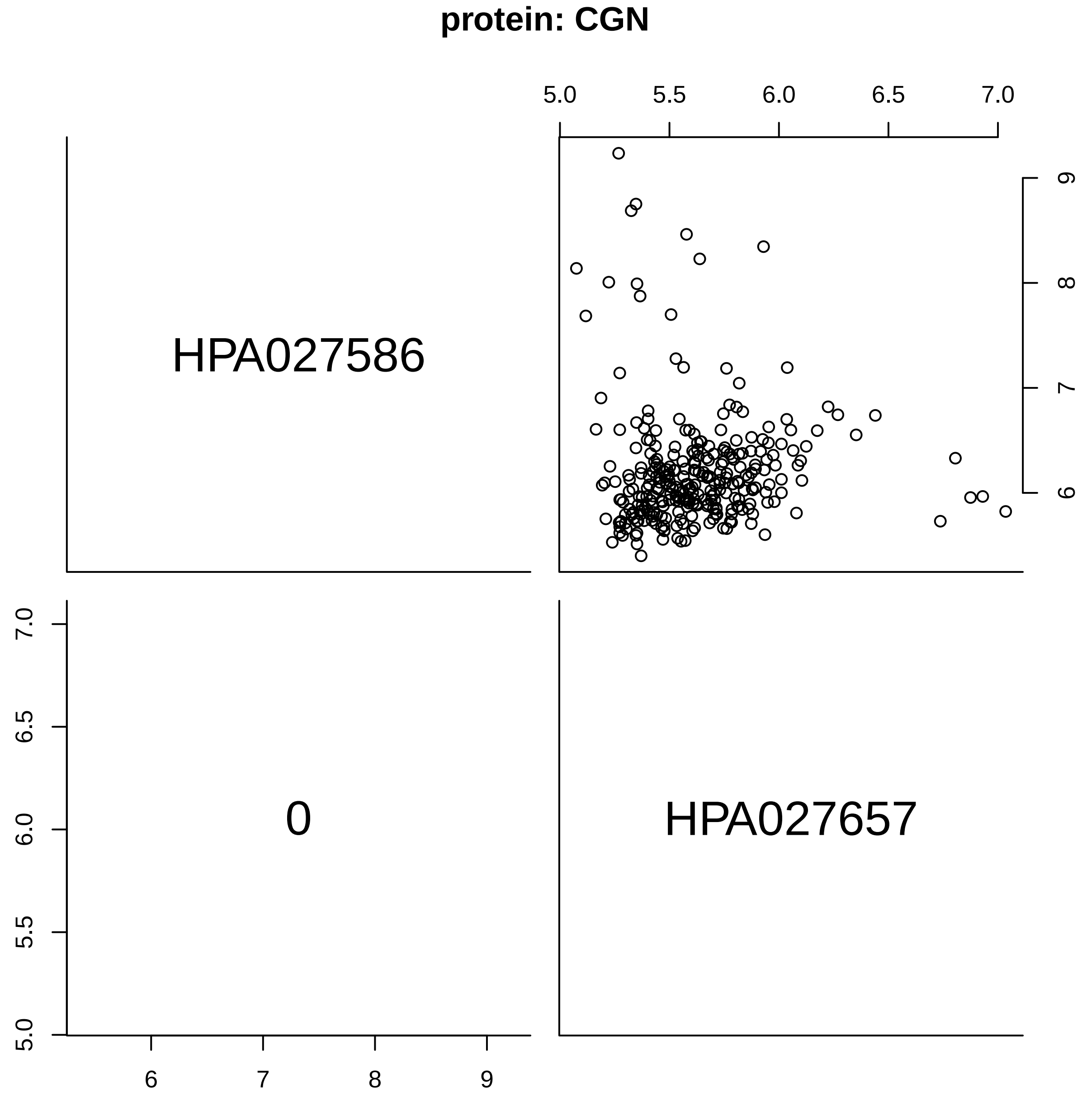
protein: CGN
HPA027586
0
HPA027657
5.0
5.5
6.0
6.5
7.0
6
7
8
9

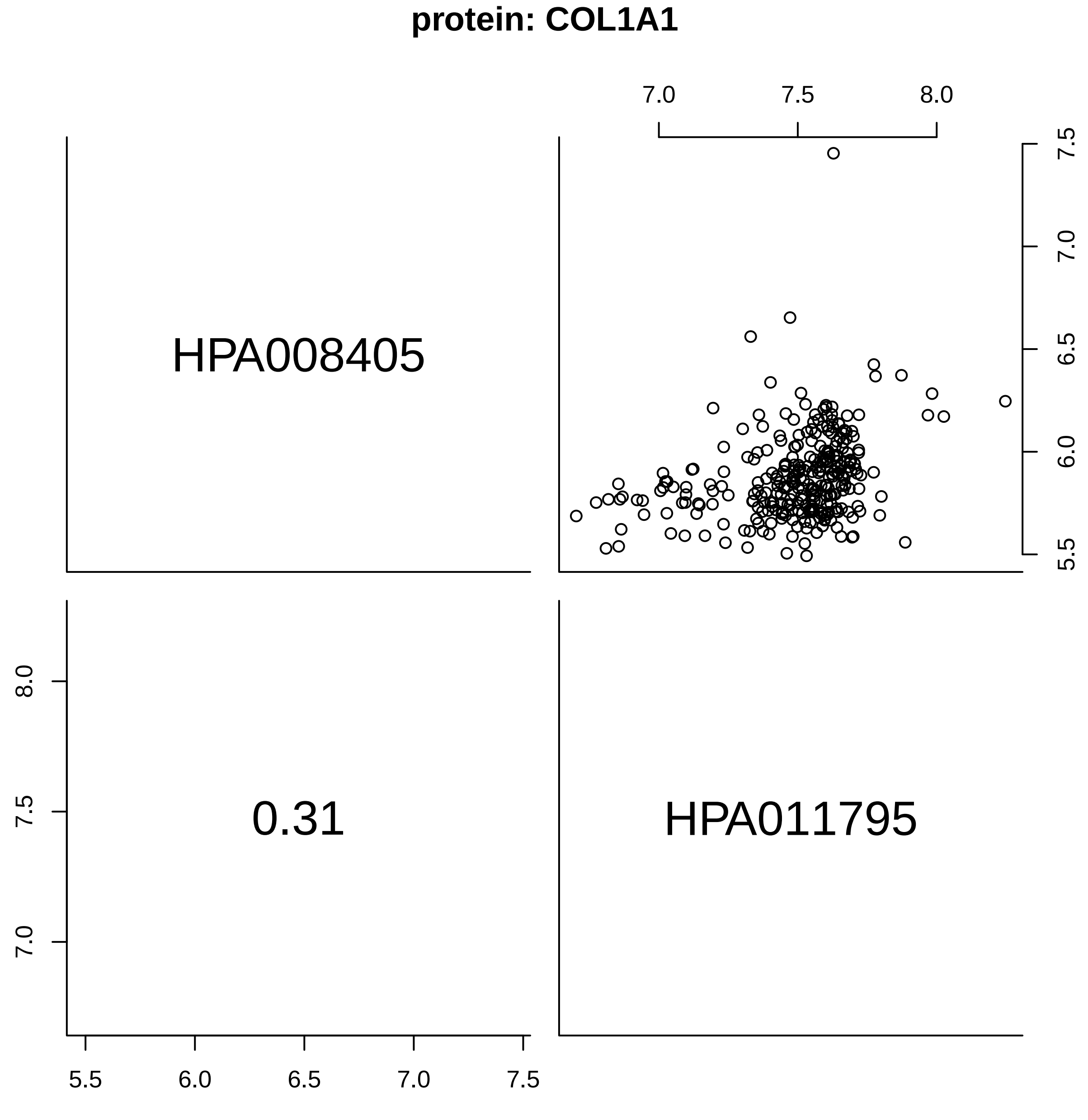
protein: COL1A1
HPA008405
HPA011795
0.31
7.0
7.5
8.0
5.5
6.0
6.5
7.0
7.5
8.0
7.5
7.0
5.5
6.0
6.5
7.0
7.5

## protein: COL6A1

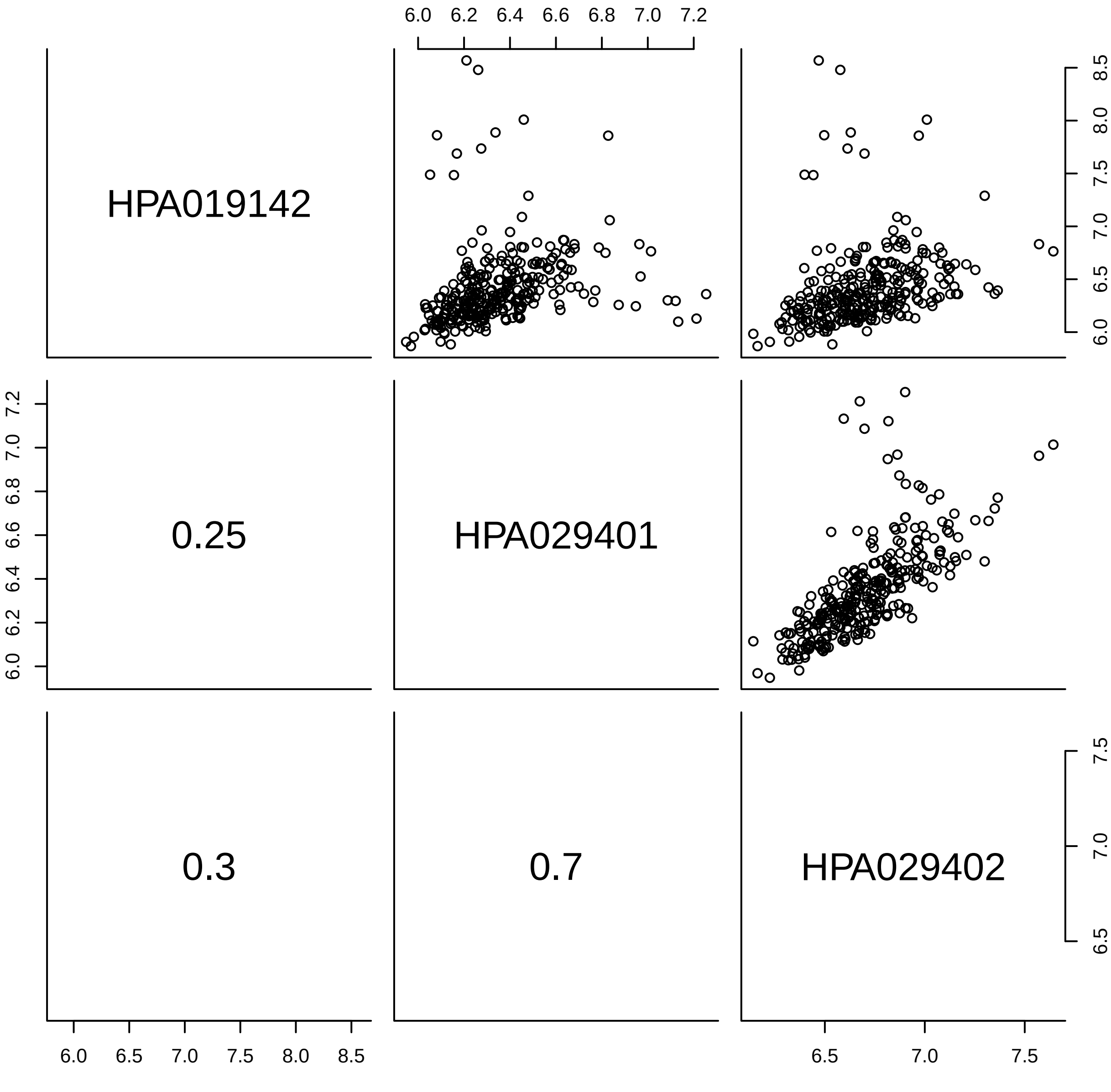

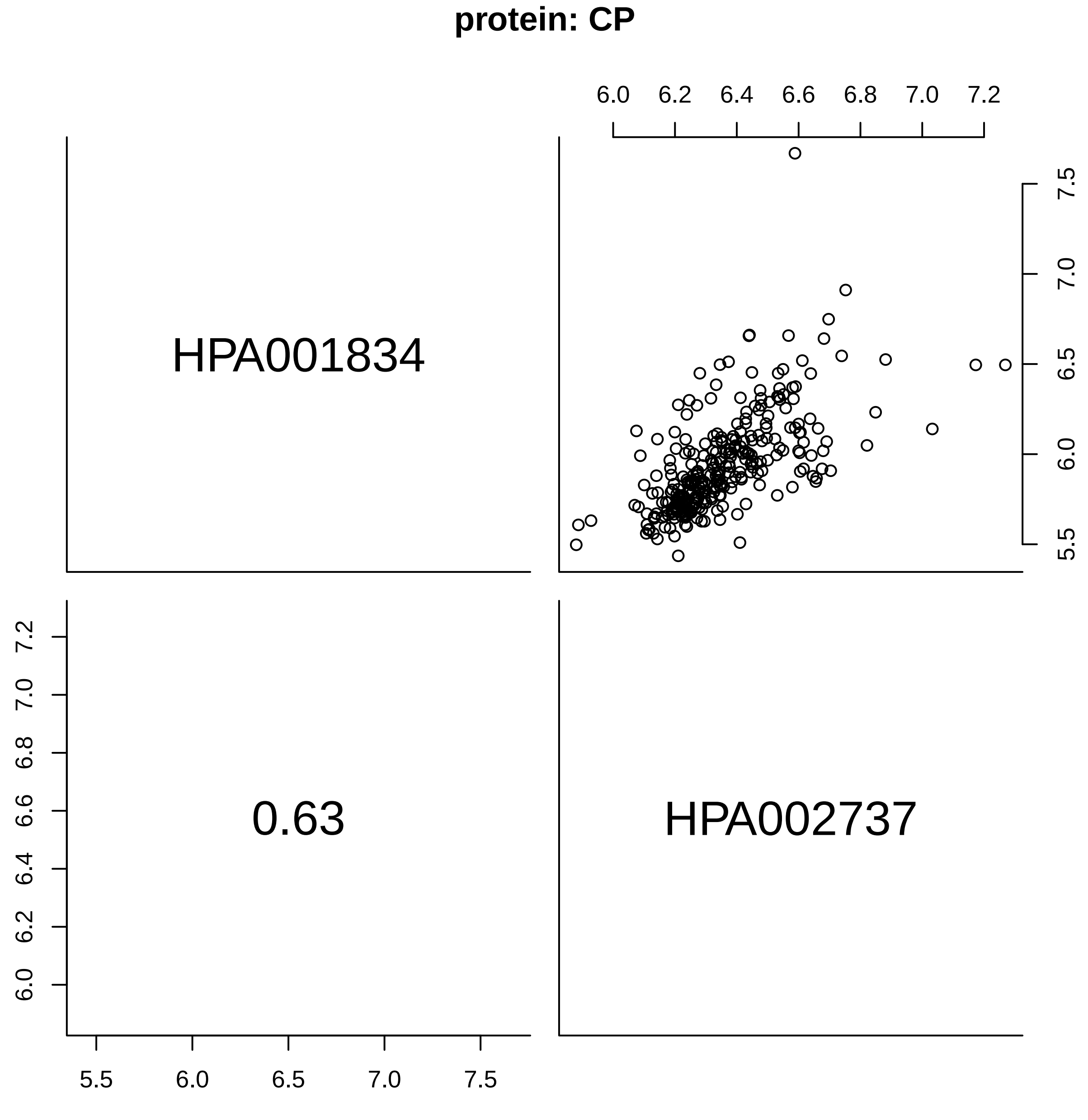

protein: CP
HPA001834
HPA002737
0.63
6.0
6.2
6.4
6.6
6.8
7.0
7.2
5.5
6.0
6.5
7.0
7.5

**protein: ETFA**

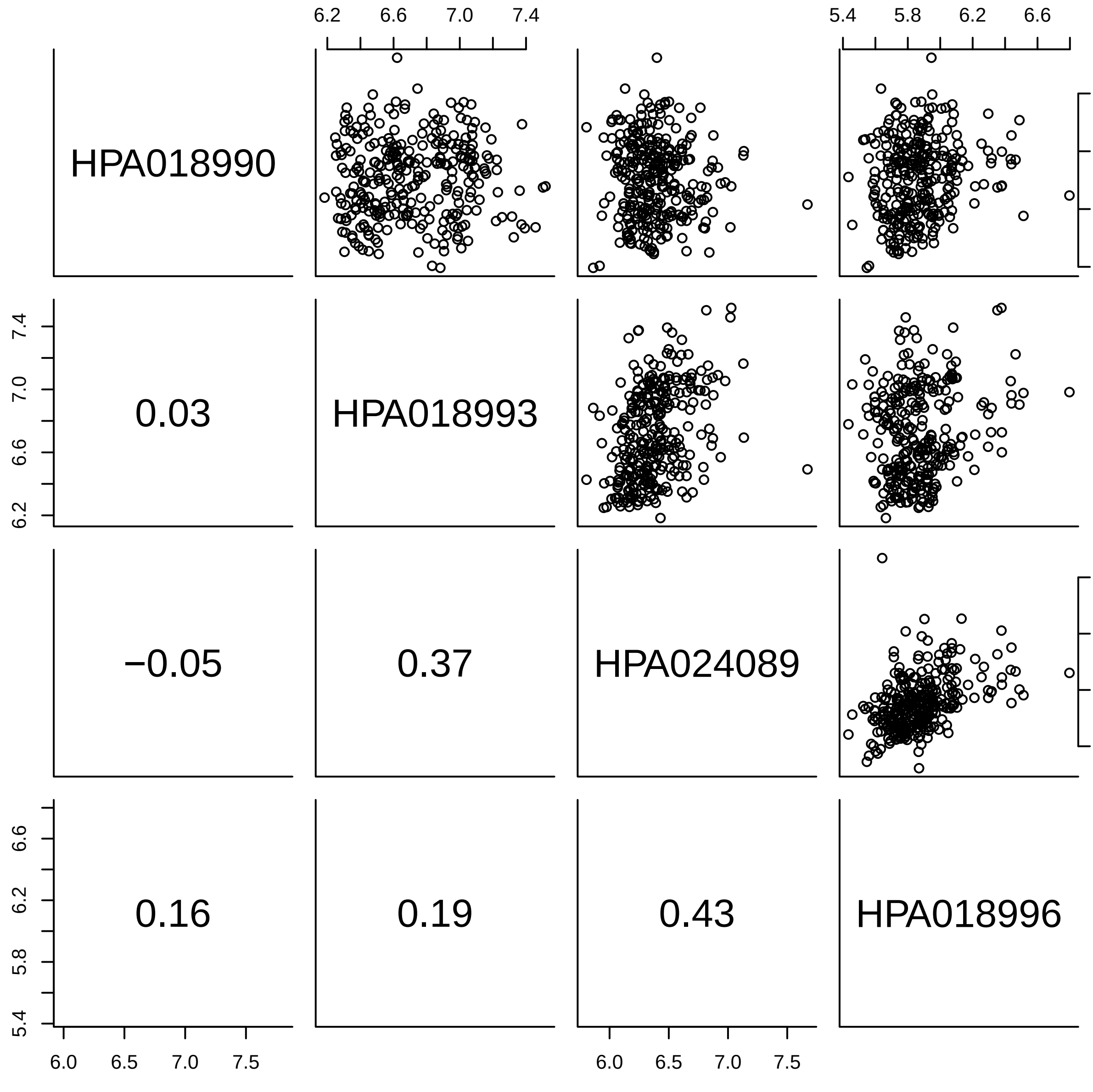

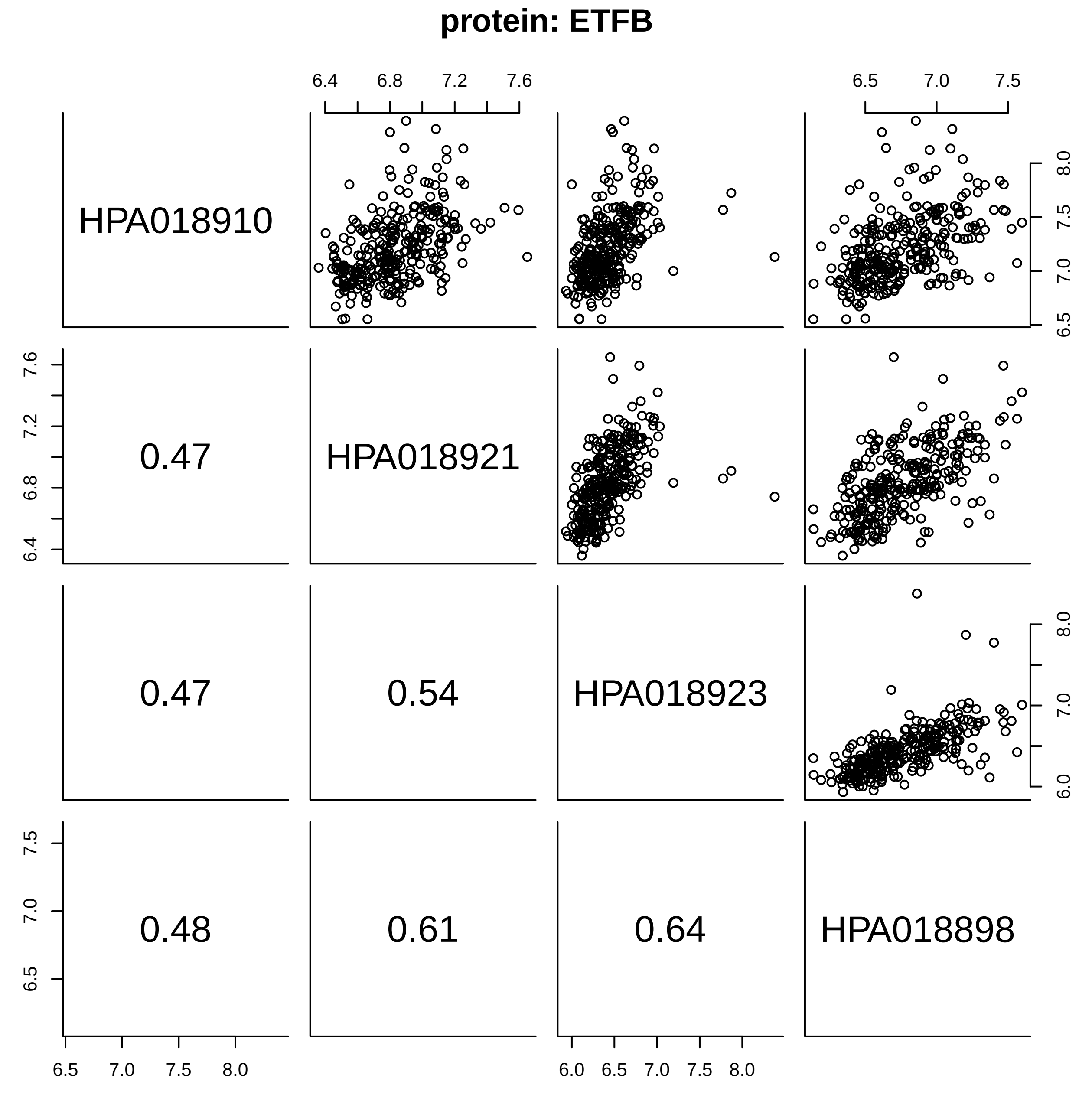

protein: ETFB
HPA018910
HPA018921
HPA018923
HPA018898
0.47
0.47
0.54
0.48
0.61
0.64
6.4
6.8
7.2
7.6
6.5
7.0
7.5
8.0
6.0

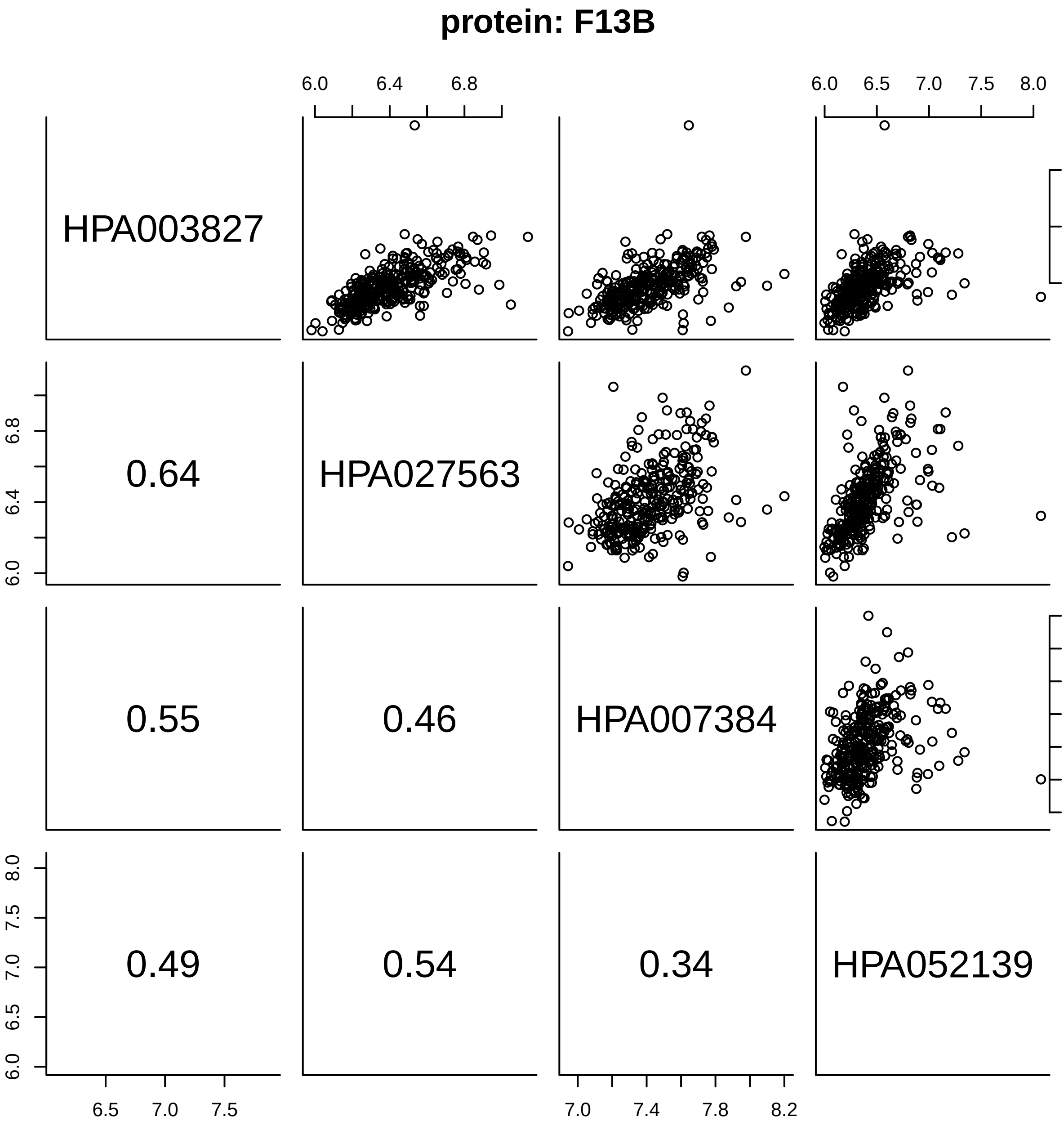
protein: F13B
HPA003827
HPA027563
HPA007384
HPA052139
0.64
0.55
0.46
0.49
0.54
0.34

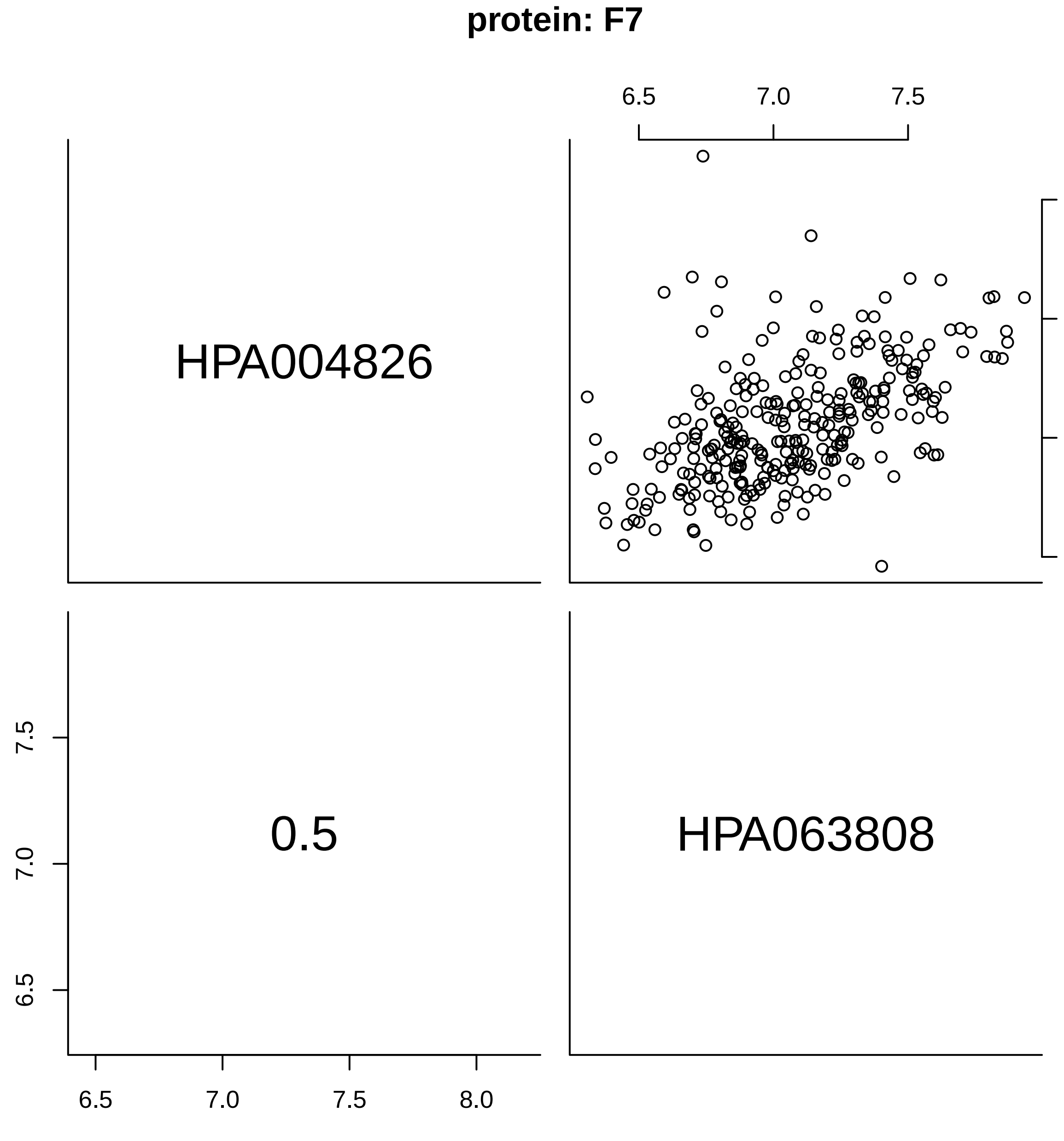

protein: F7
HPA004826
0.5
HPA063808
6.5
7.0
7.5
8.0

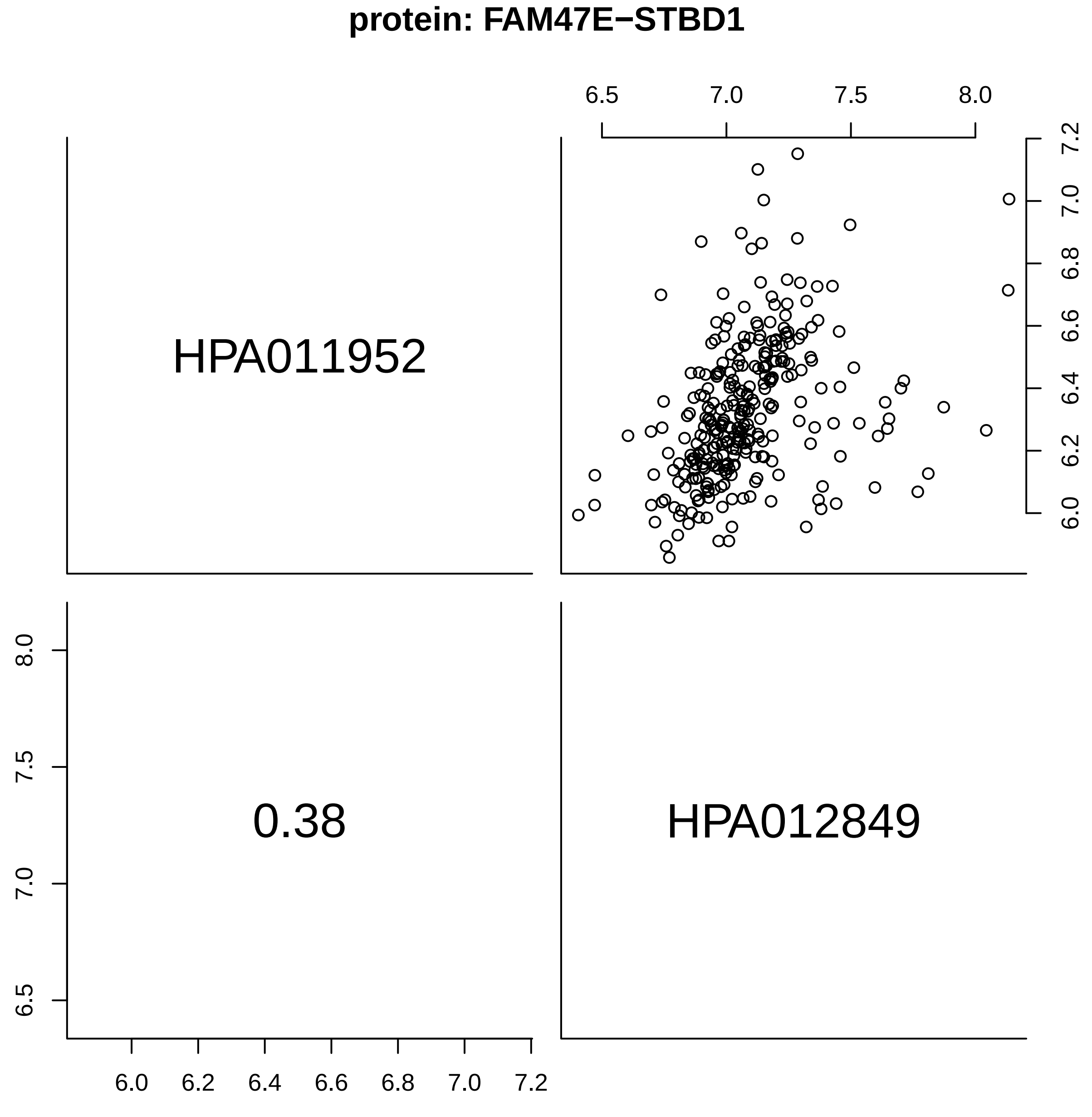

protein: FAM47E−STBD1
HPA011952
0.38
HPA012849
6.5
7.0
7.5
8.0
6.0
6.2
6.4
6.6
6.8
7.0
7.2

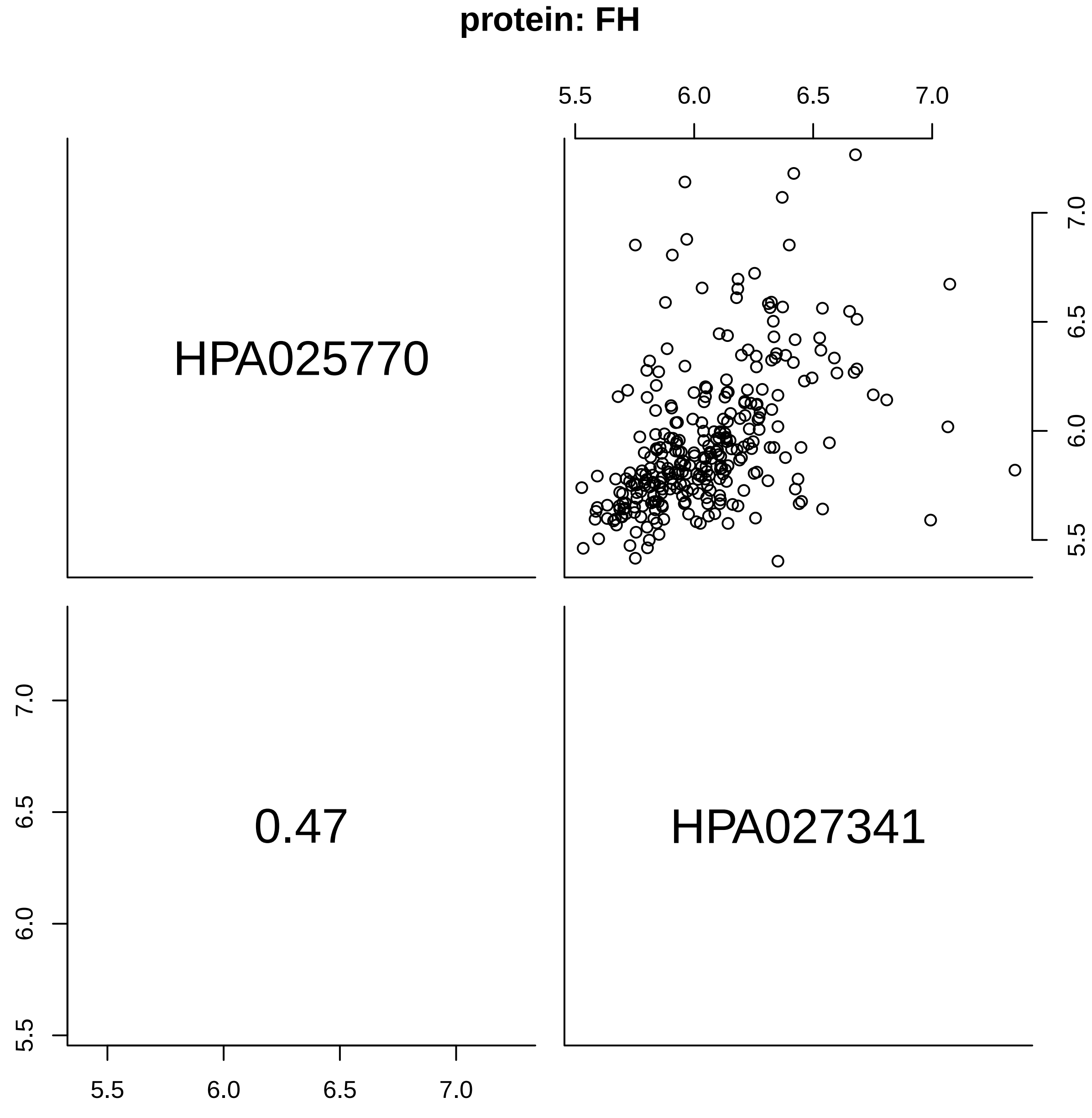
protein: FH
HPA025770
0.47
HPA027341
5.5
6.0
6.5
7.0

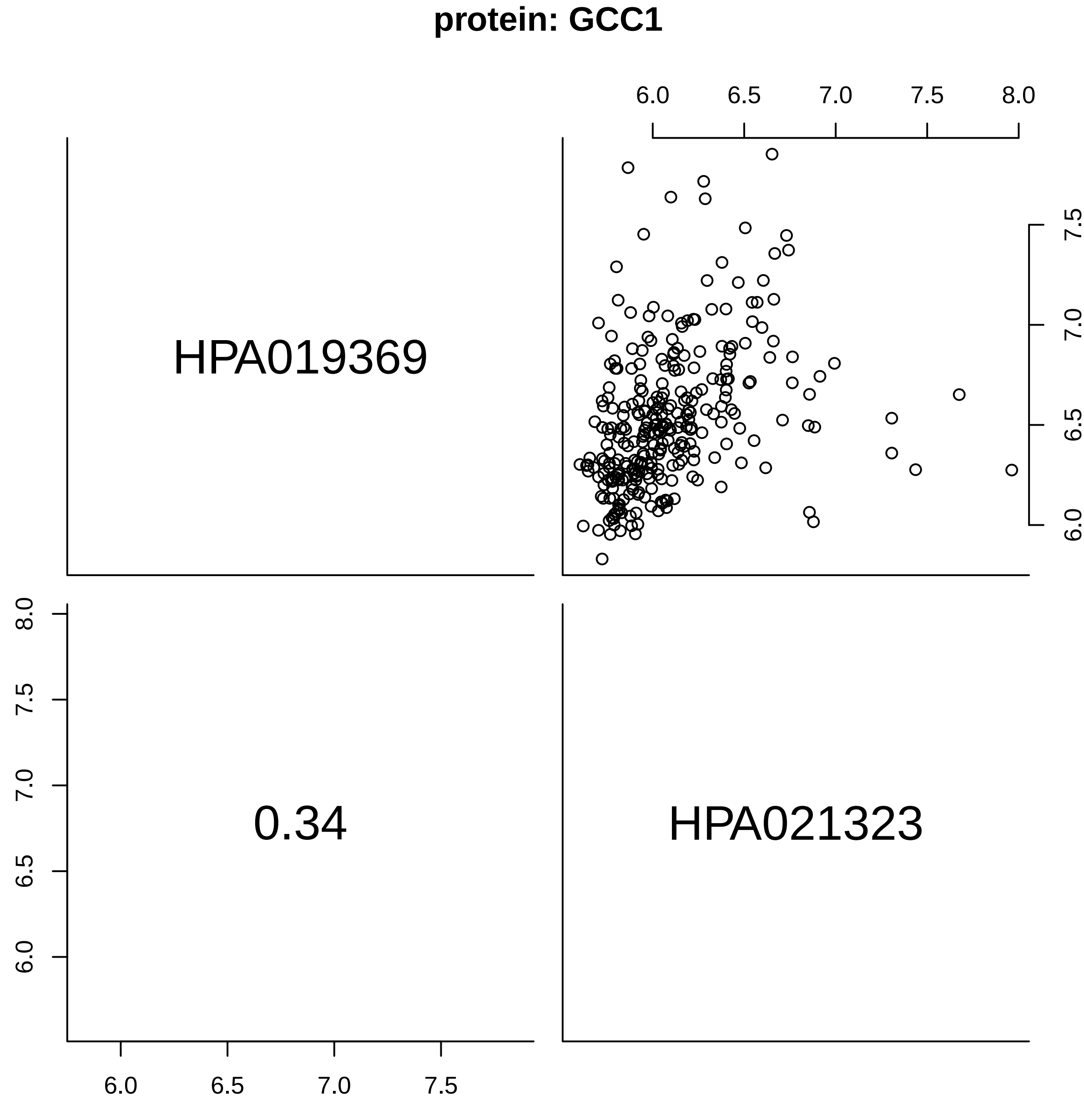
protein: GCC1
HPA019369
0.34
HPA021323
6.0
6.5
7.0
7.5
8.0

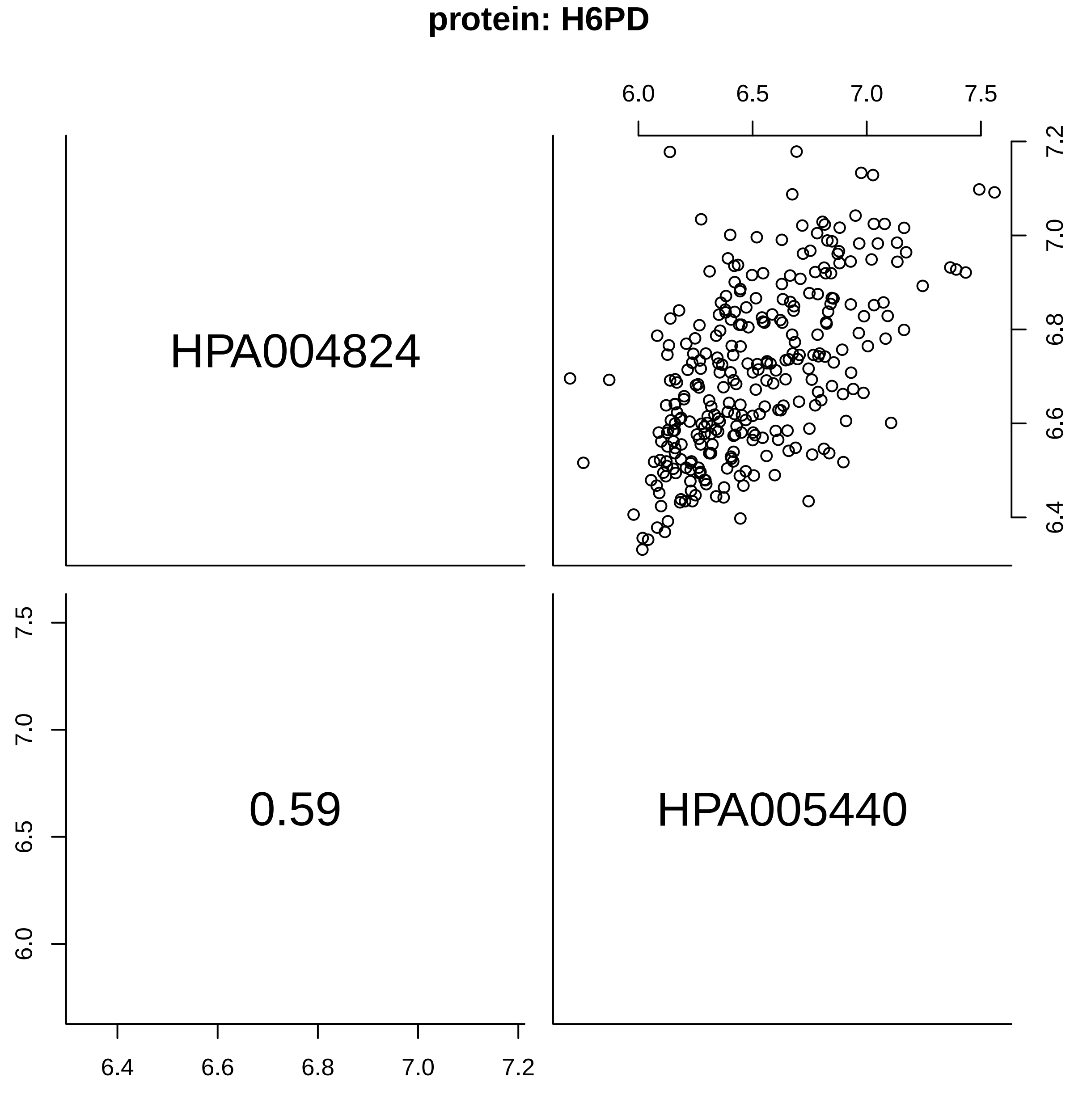
protein: H6PD
HPA004824
0.59
HPA005440
6.0
6.5
7.0
7.5
6.4
6.6
6.8
7.0
7.2

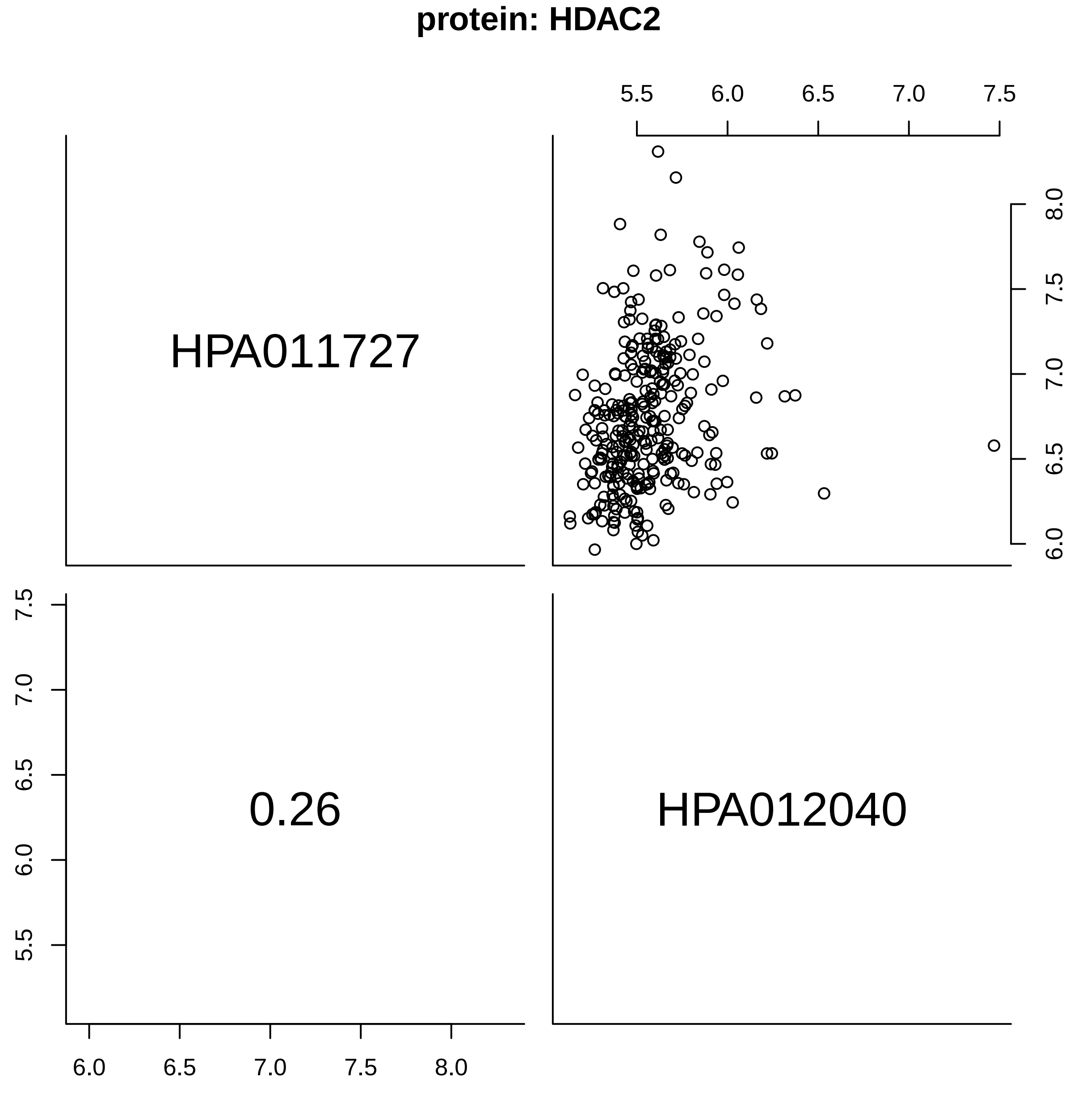
protein: HDAC2
HPA011727
0.26
HPA012040
5.5
6.0
6.5
7.0
7.5
8.0

## protein: ITGA2B

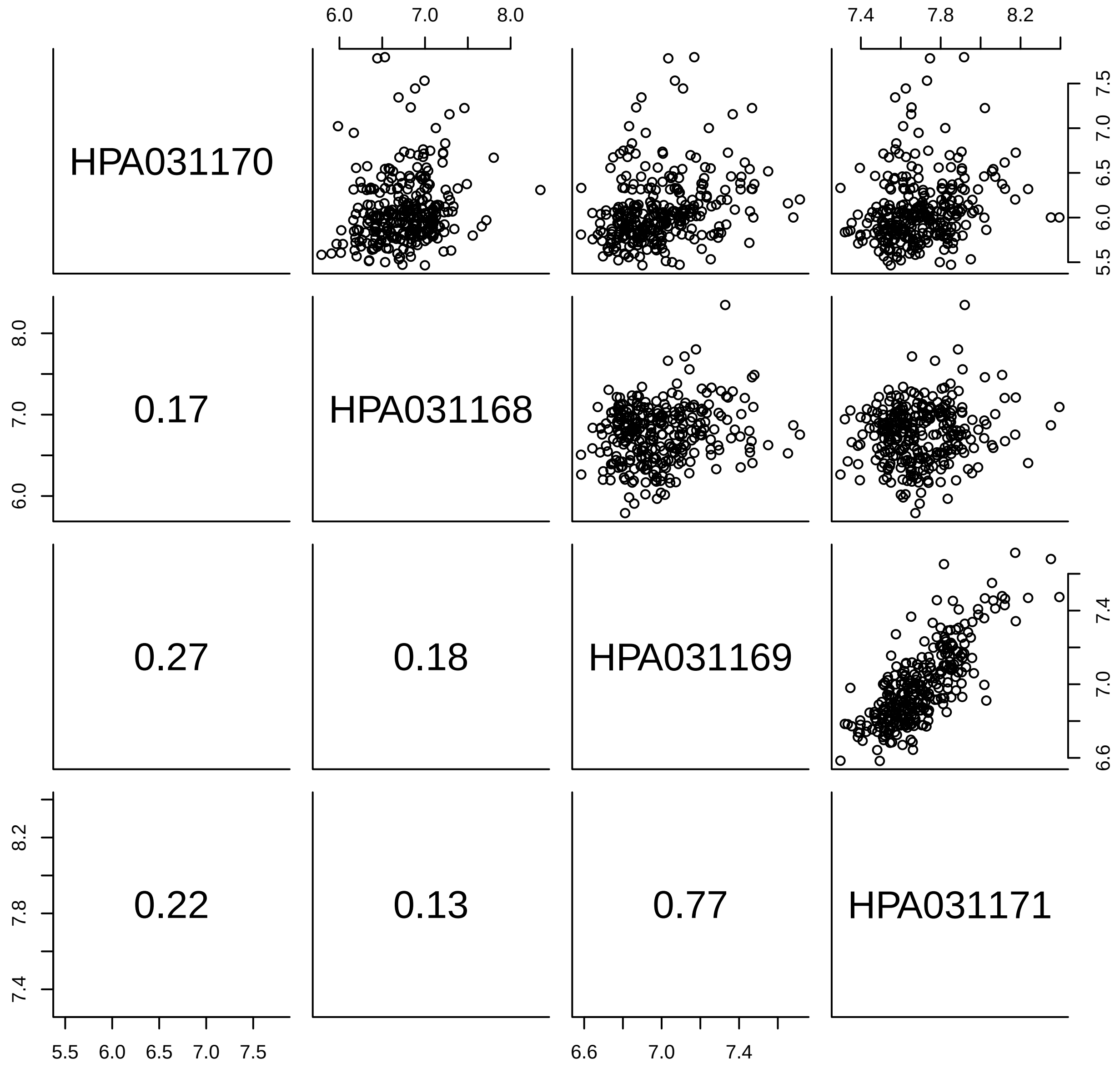

**protein: JAM3**

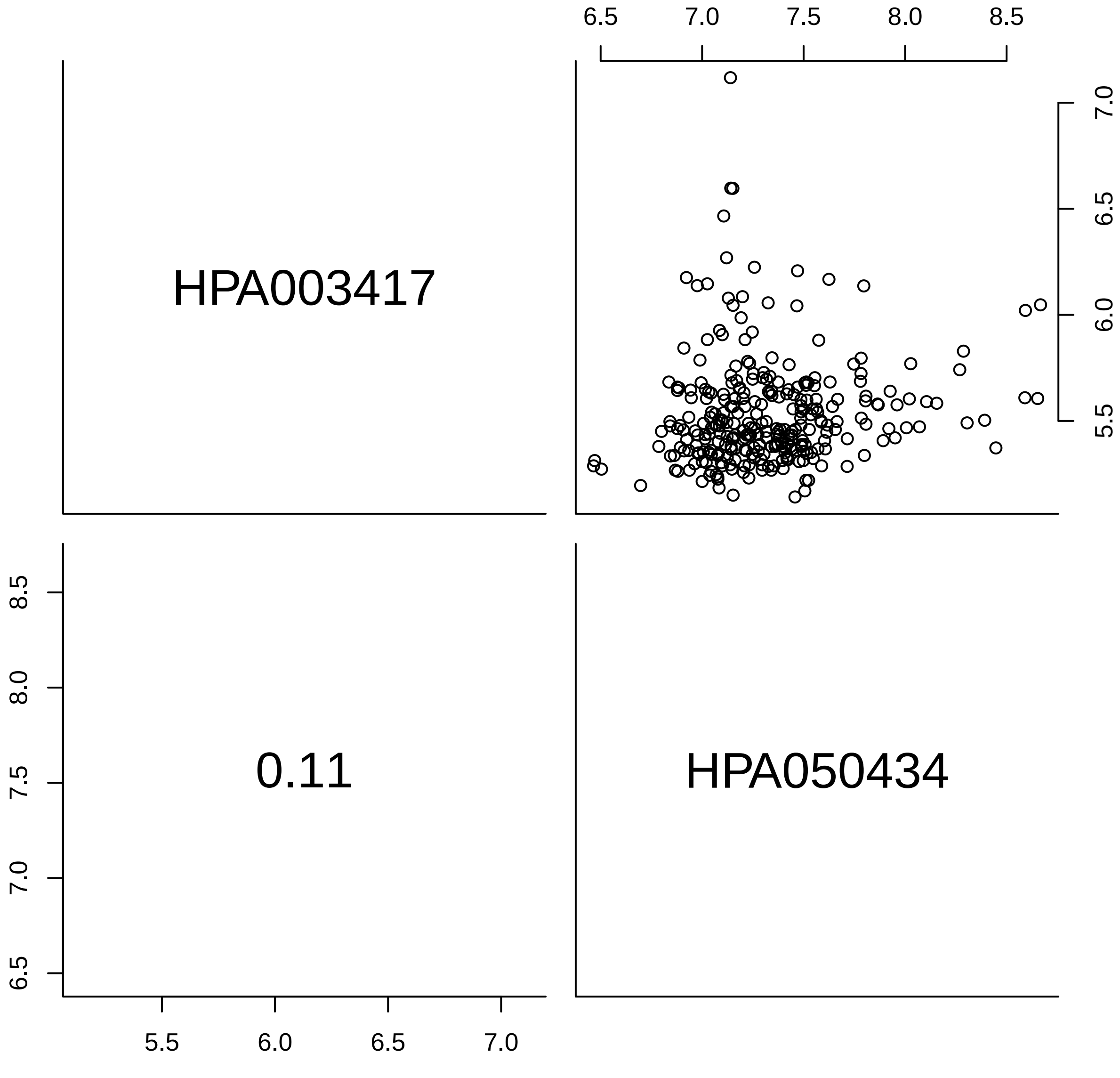

## protein: LAMC1

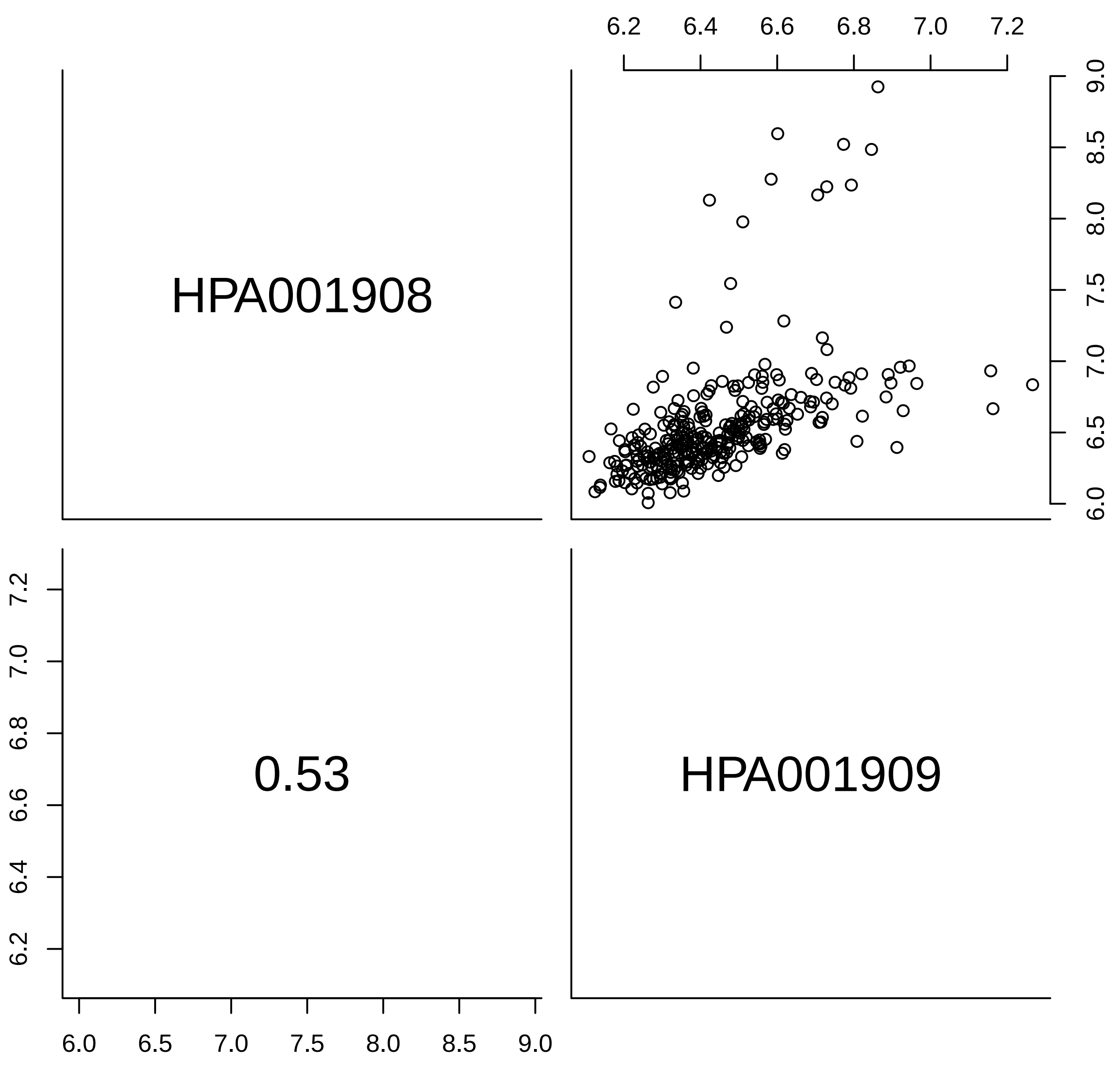

# protein: LMOD1

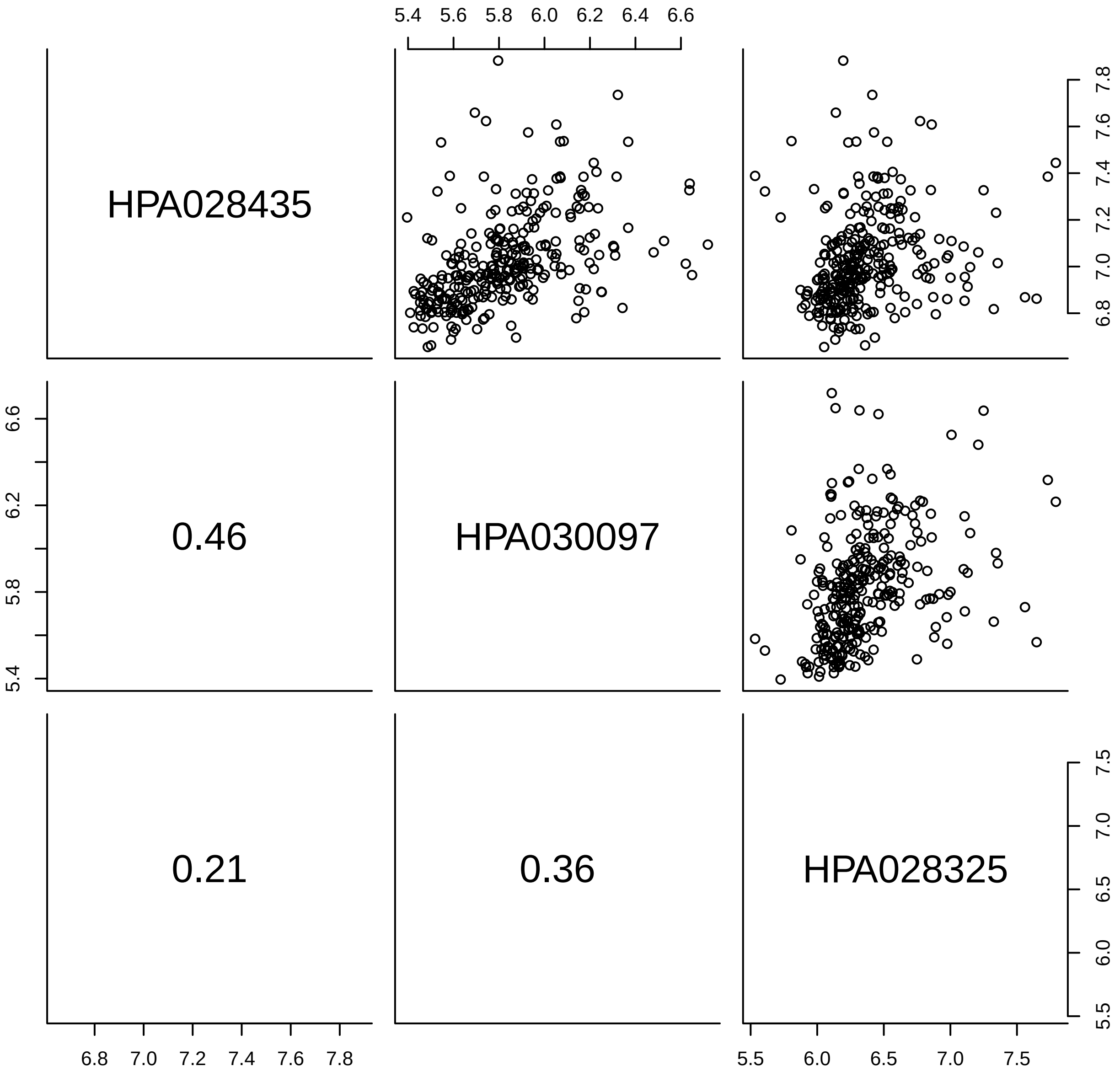

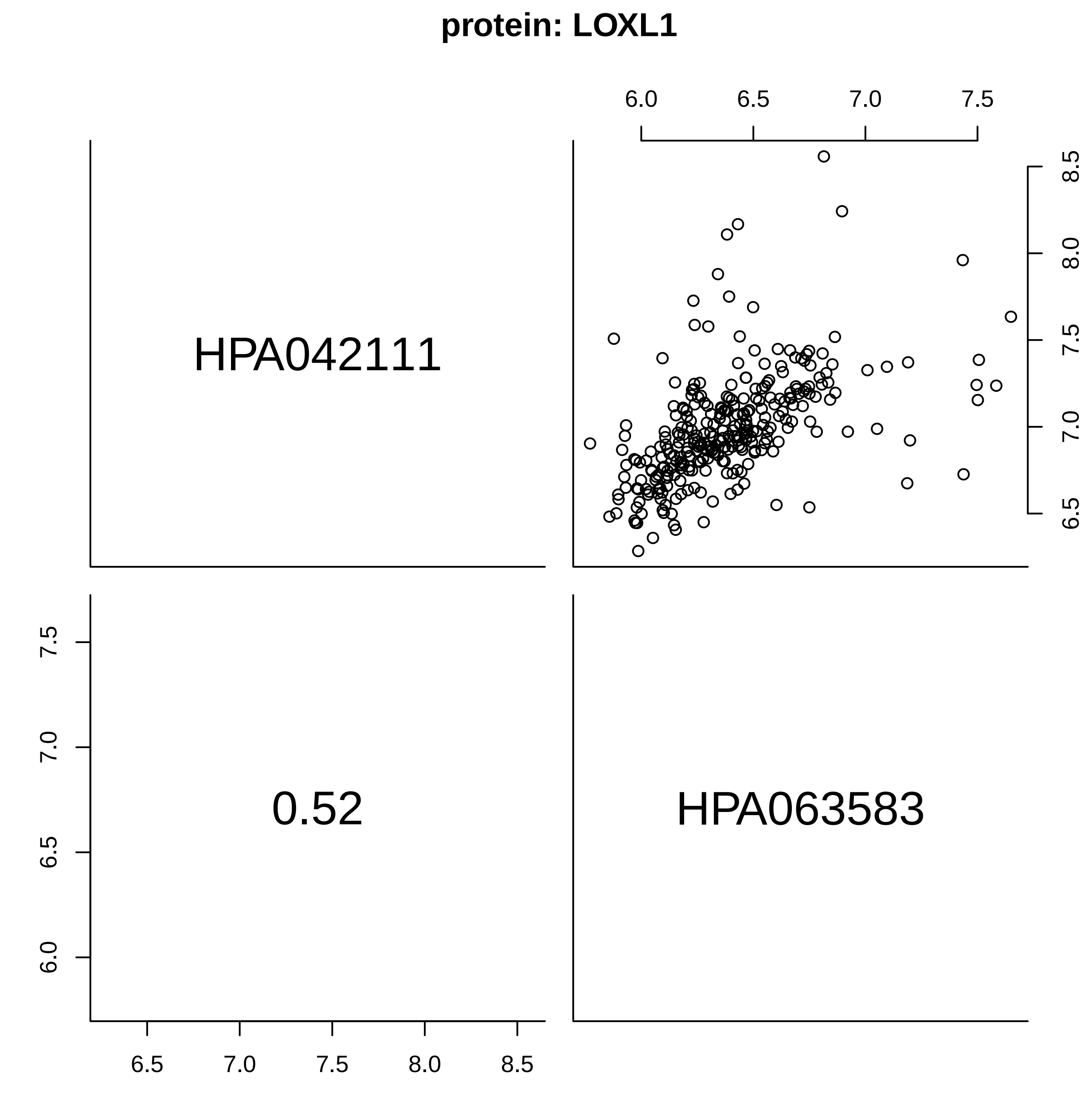

protein: LOXL1
HPA042111
0.52
HPA063583
6.0
6.5
7.0
7.5
8.0
8.5

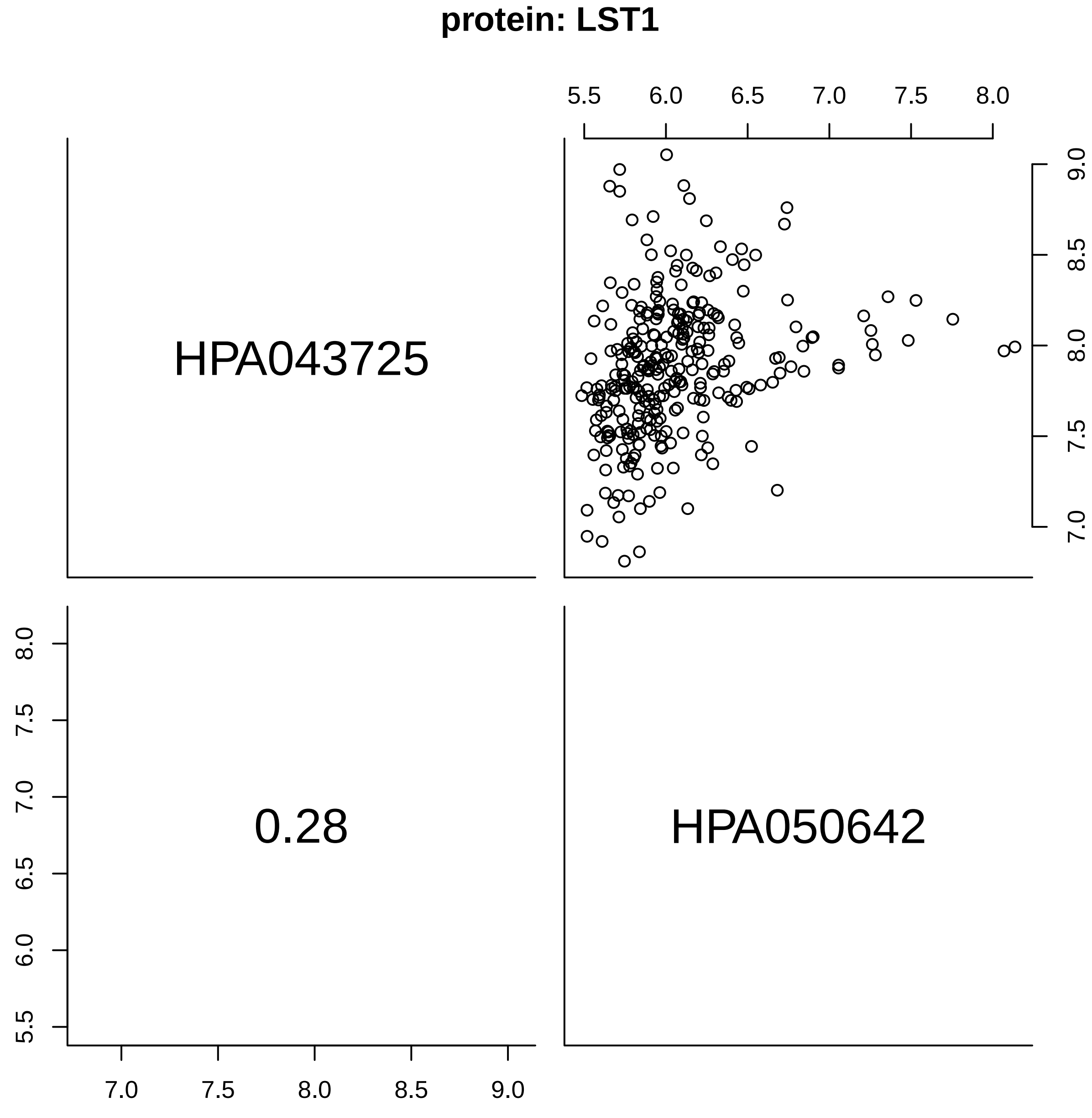
protein: LST1
HPA043725
0.28
HPA050642
5.5
6.0
6.5
7.0
7.5
8.0
8.5
9.0

**protein: LTBP4**

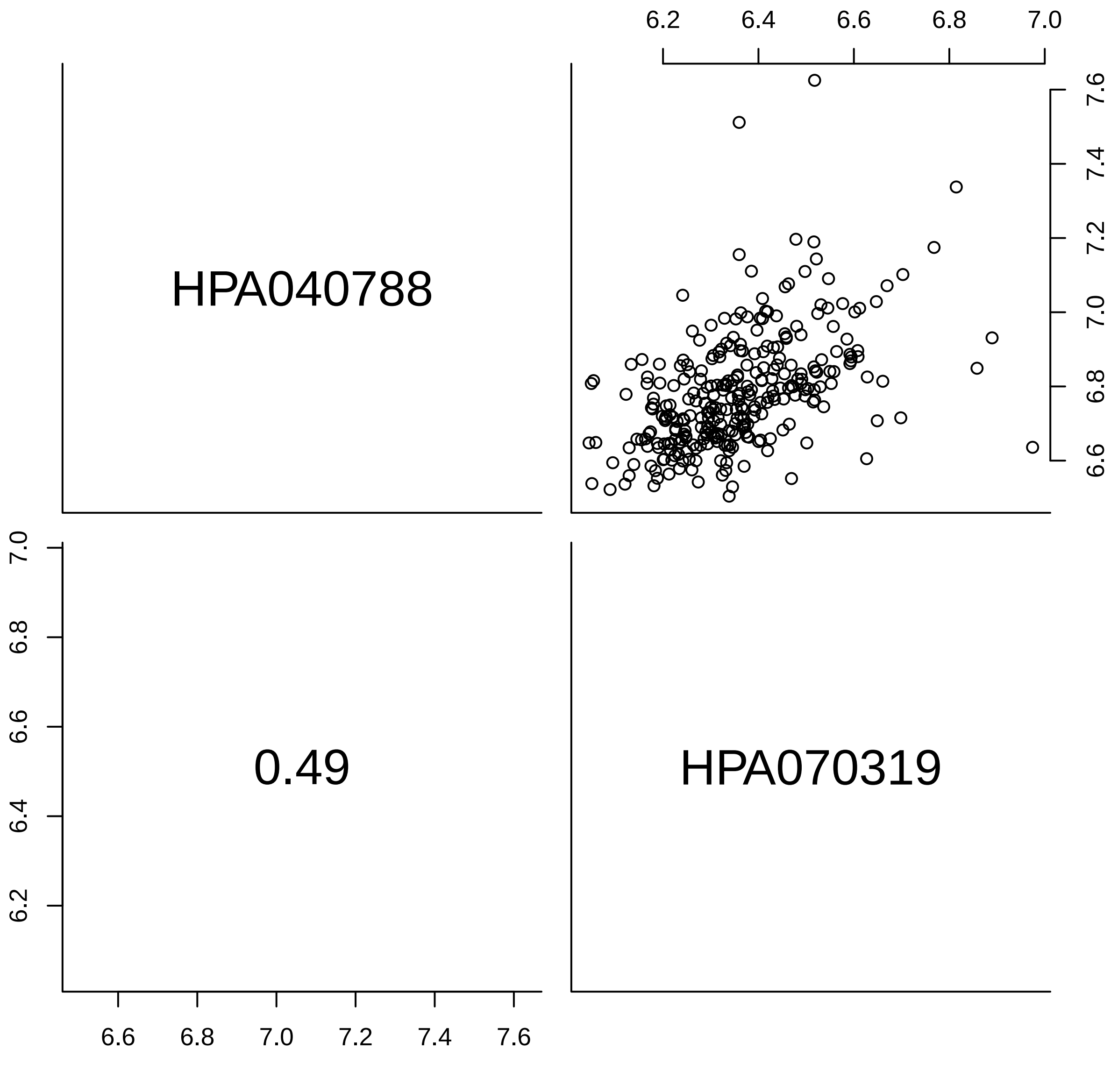

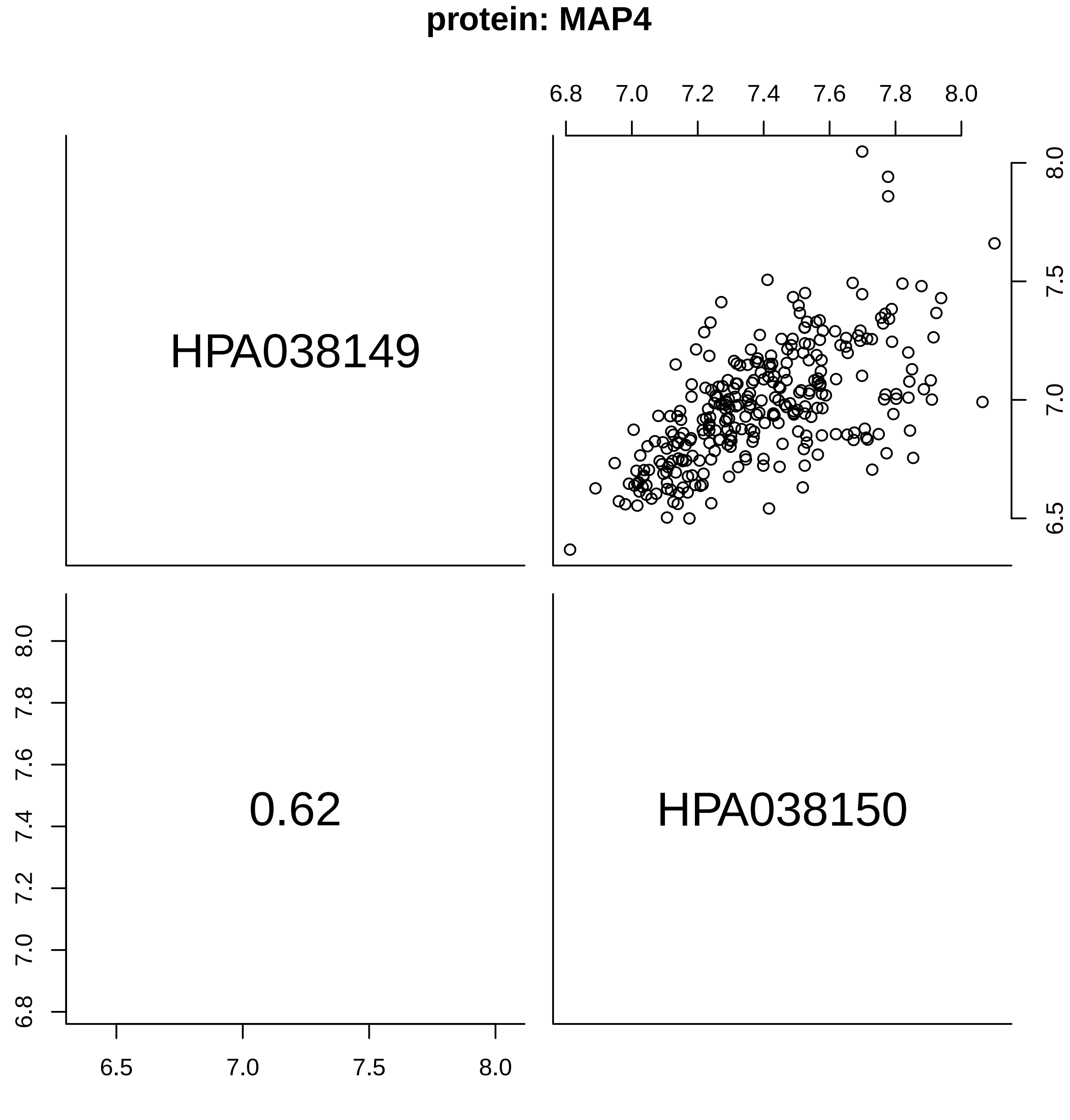
protein: MAP4
HPA038149
HPA038150
0.62
6.8
7.0
7.2
7.4
7.6
7.8
8.0
6.5
7.0
7.5
8.0

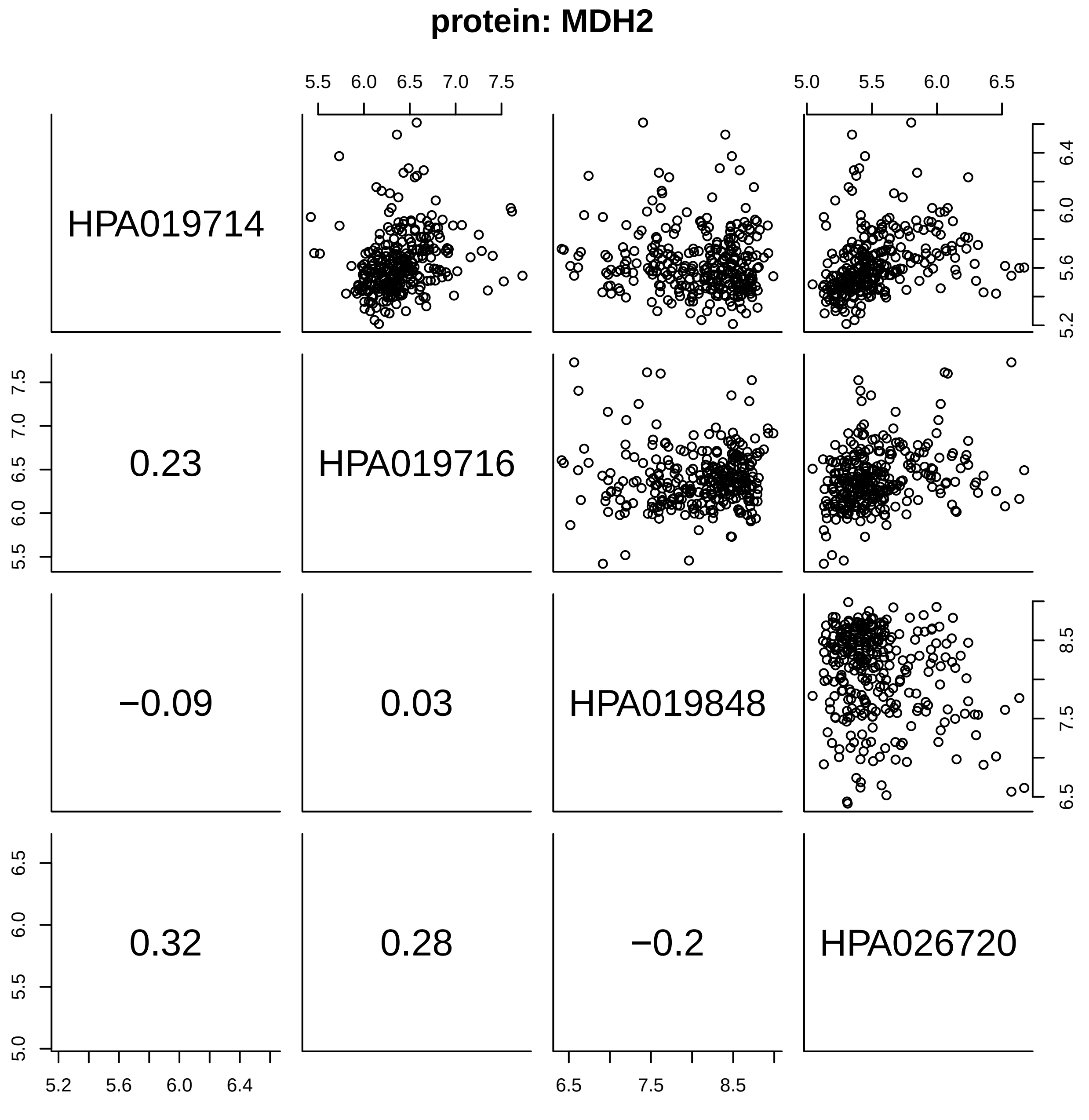
protein: MDH2
HPA019714
HPA019716
HPA019848
HPA026720
0.23
−0.09
0.03
0.32
0.28
−0.2
5.5
6.0
6.5
7.0
7.5
5.0
5.5
6.0
6.5
5.2
5.6
6.0
6.4
8.5
7.5
6.5

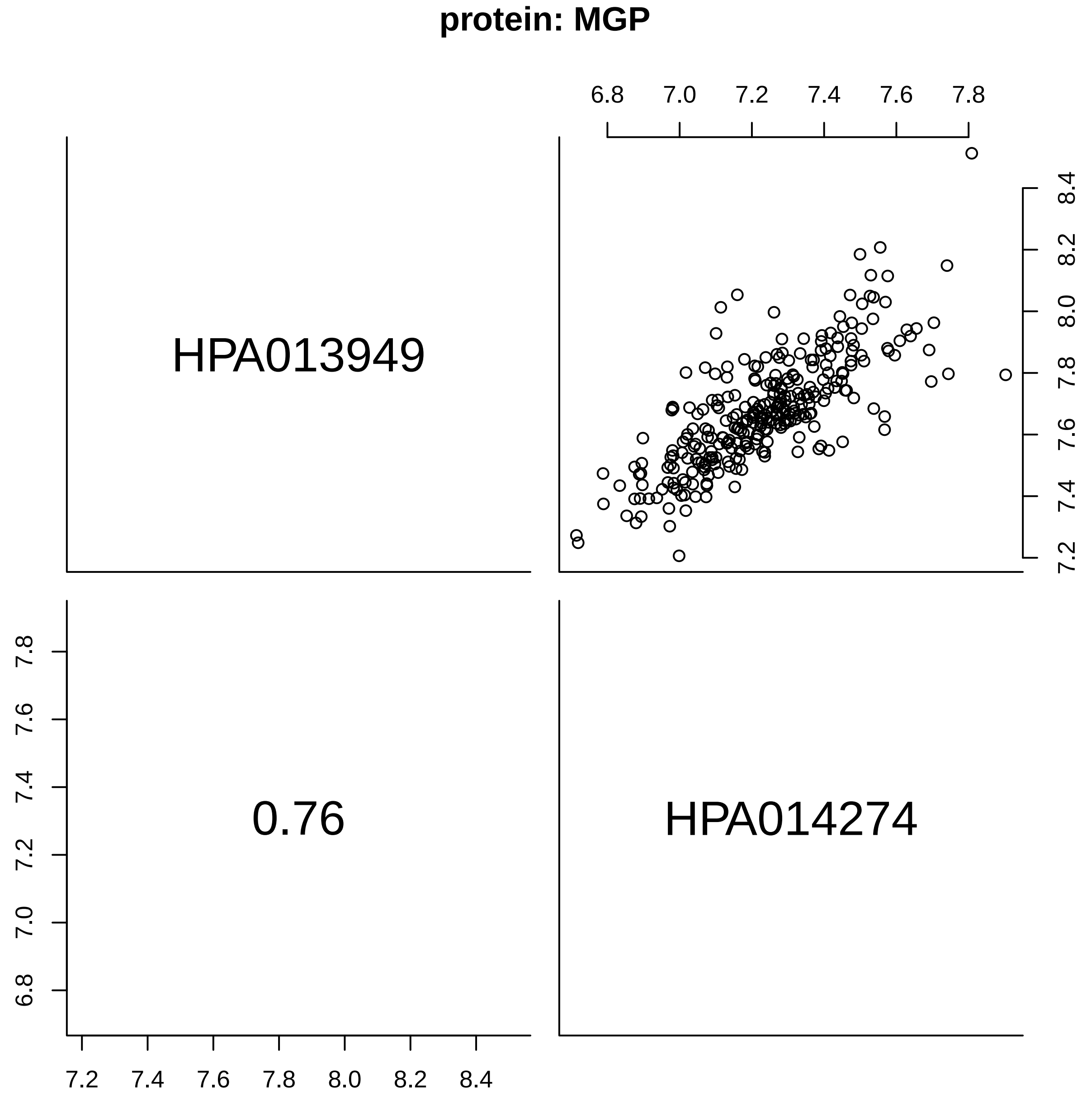

protein: MGP
6.8
7.0
7.2
7.4
7.6
7.8
8.4
8.2
8.0
7.8
7.6
7.4
7.2
HPA013949
7.8
7.6
7.4
7.2
7.0
6.8
0.76
HPA014274
7.2
7.4
7.6
7.8
8.0
8.2
8.4

**protein: MMP9**

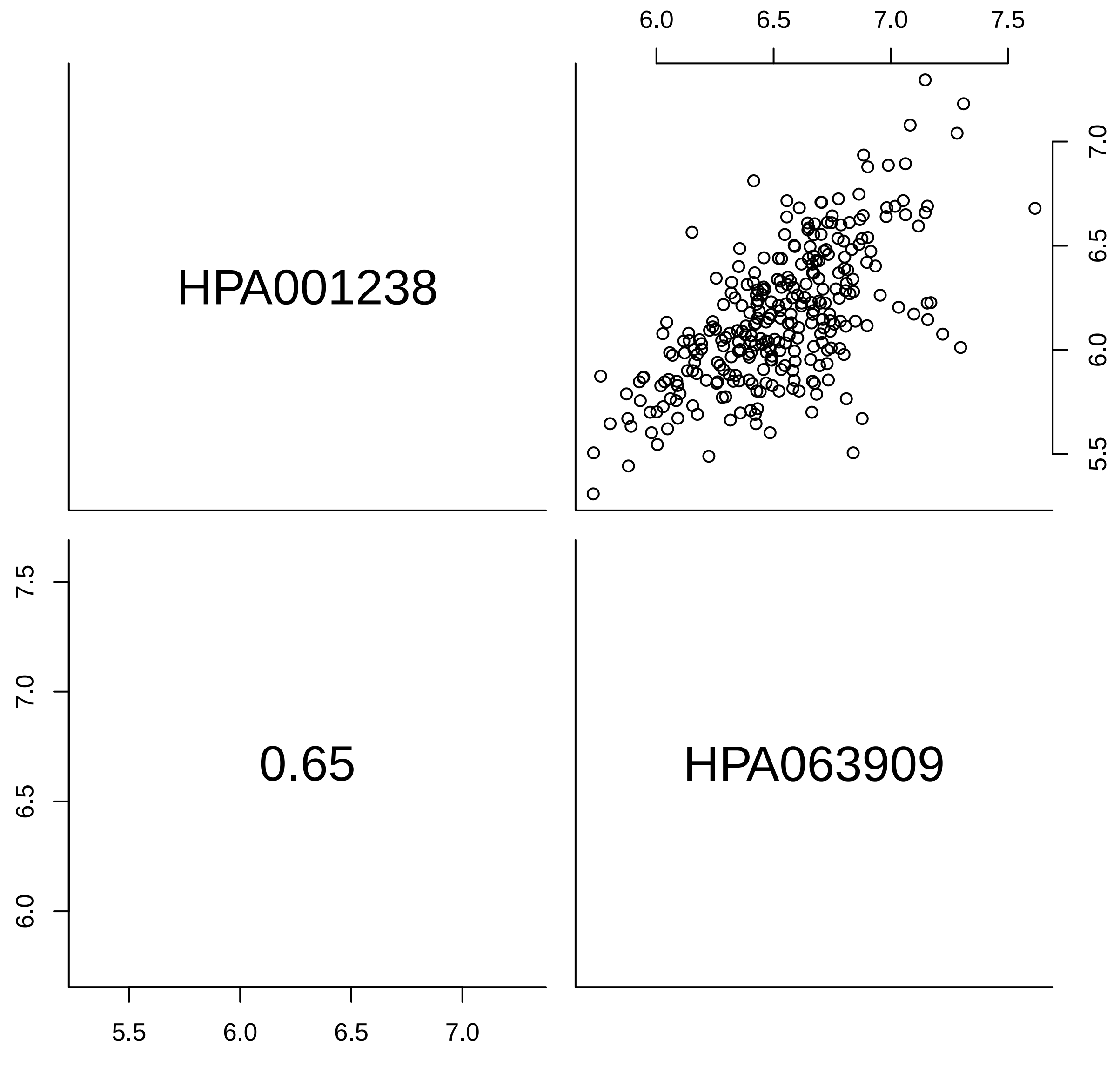

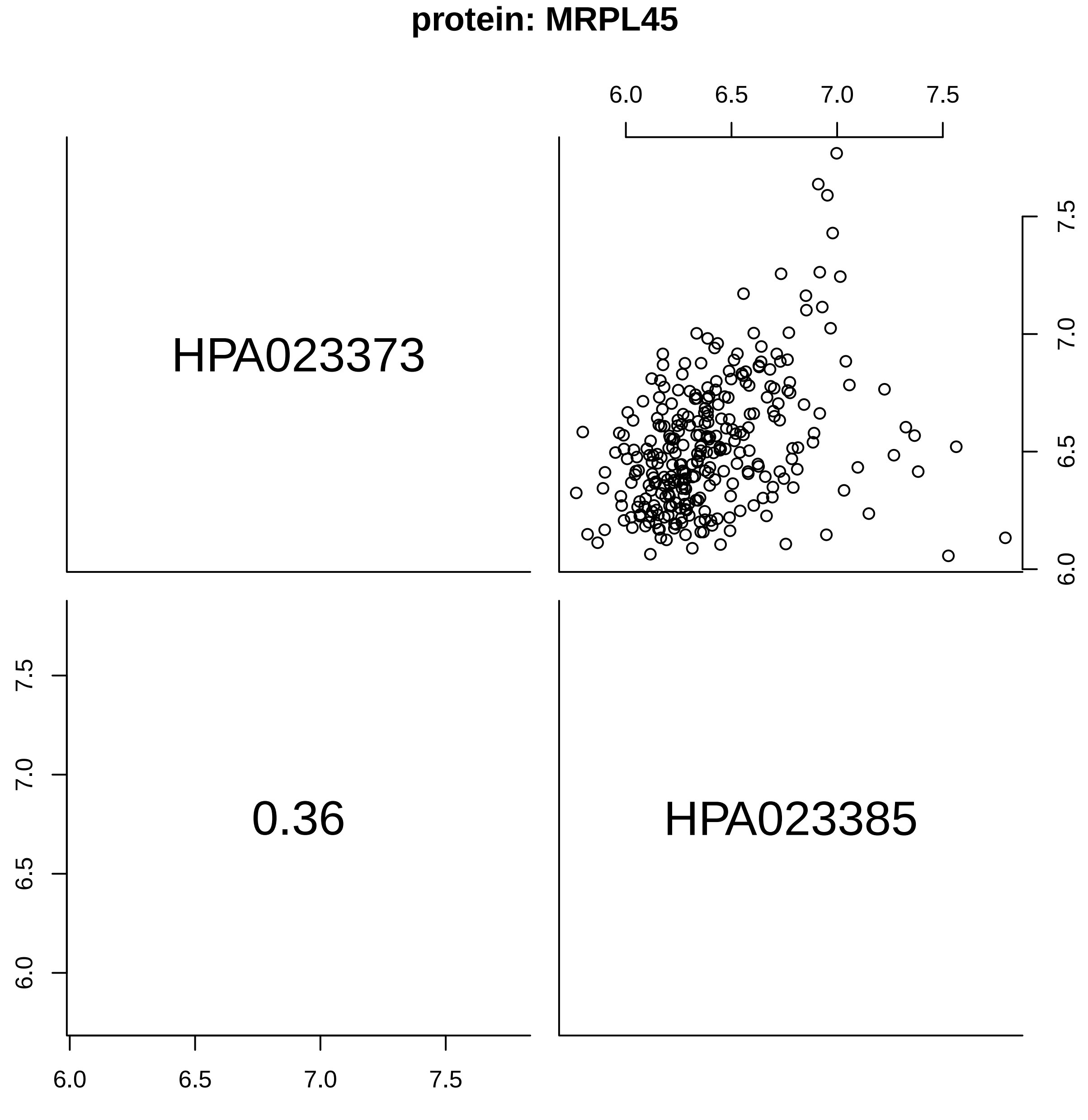
protein: MRPL45
HPA023373
0.36
HPA023385
6.0
6.5
7.0
7.5

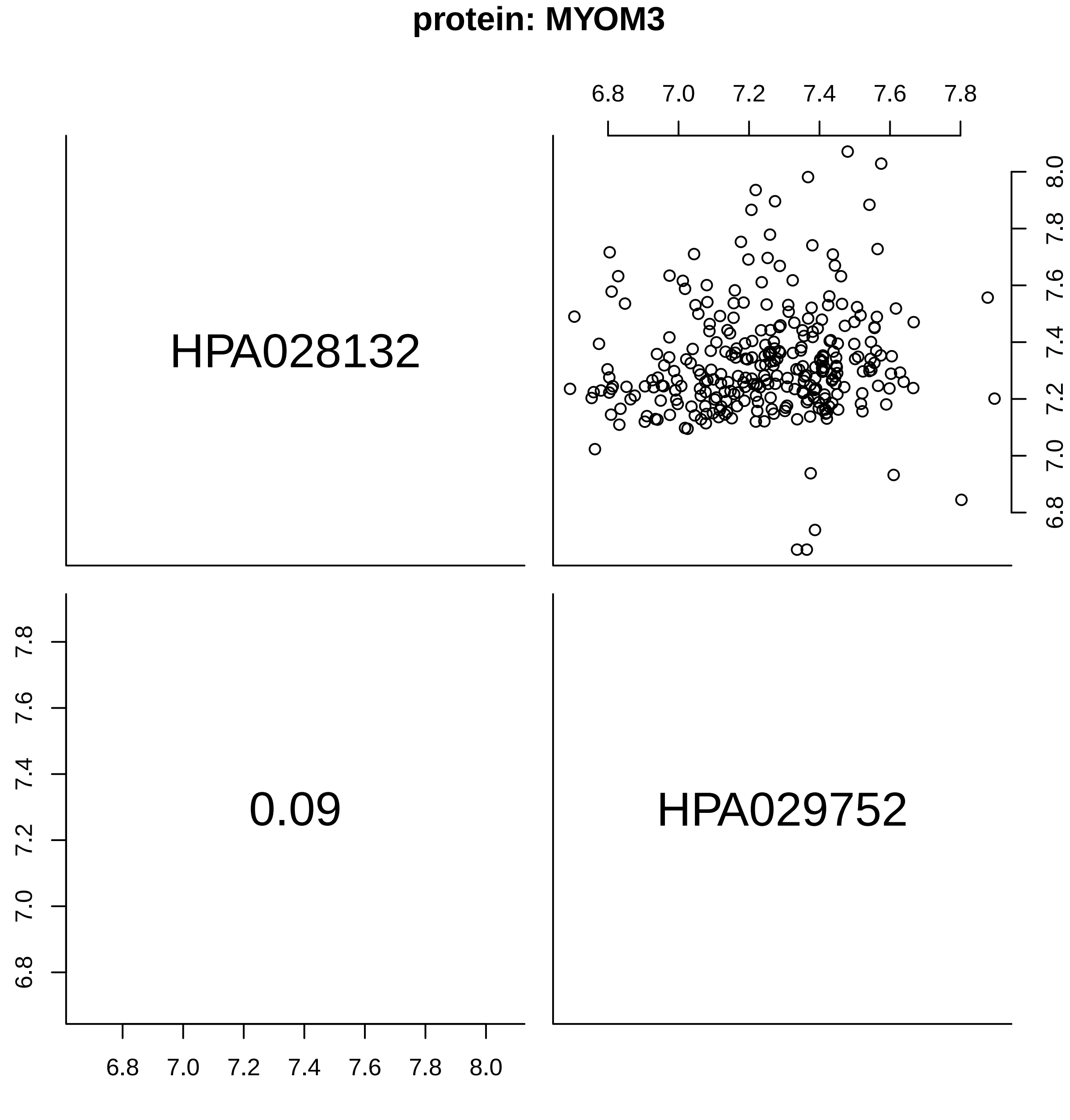
protein: MYOM3
HPA028132
HPA029752
0.09
6.8
7.0
7.2
7.4
7.6
7.8
8.0

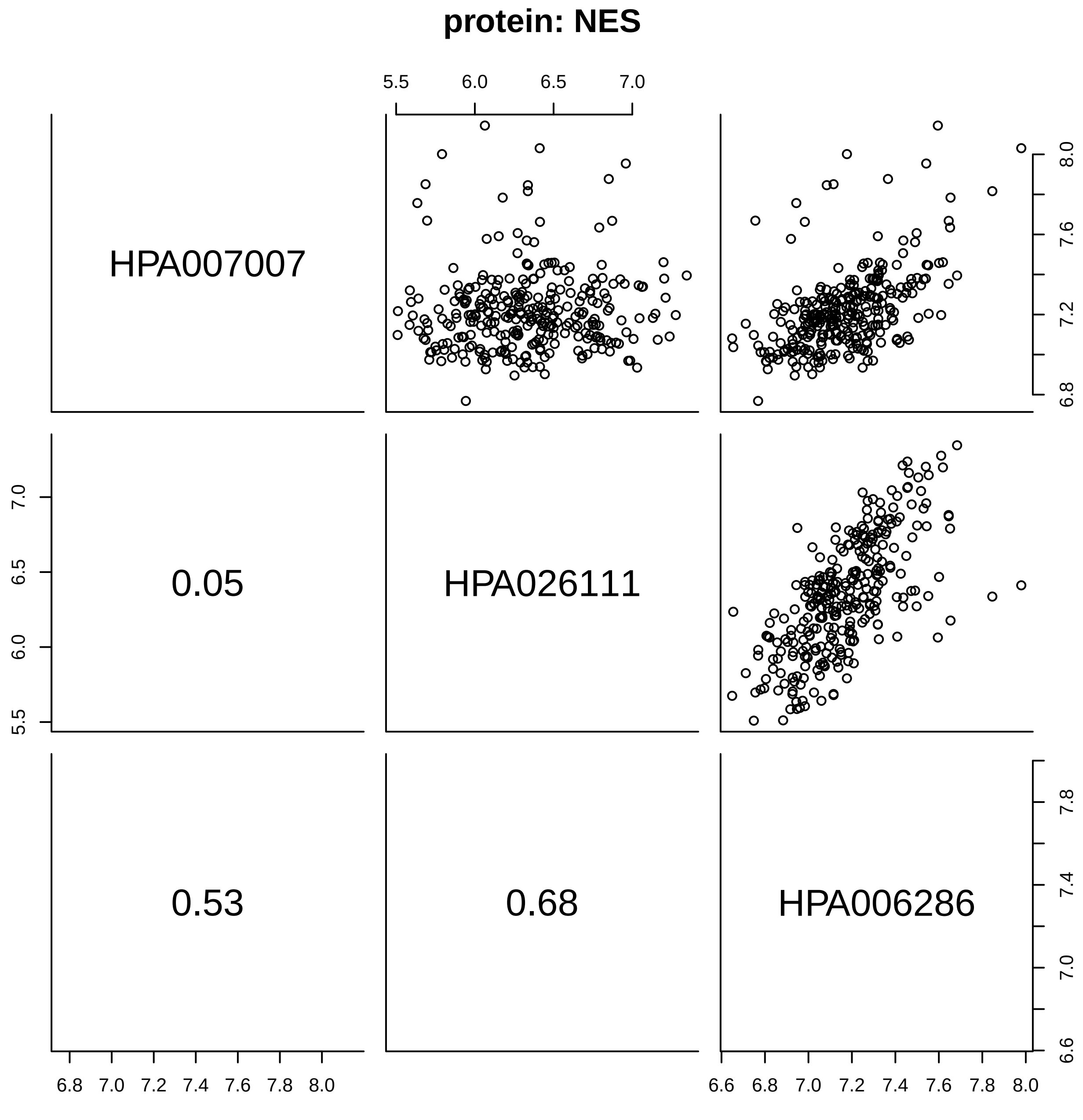
protein: NES
HPA007007
HPA026111
HPA006286
0.05
0.53
0.68
5.5
6.0
6.5
7.0
6.8
7.0
7.2
7.4
7.6
7.8
8.0
6.6

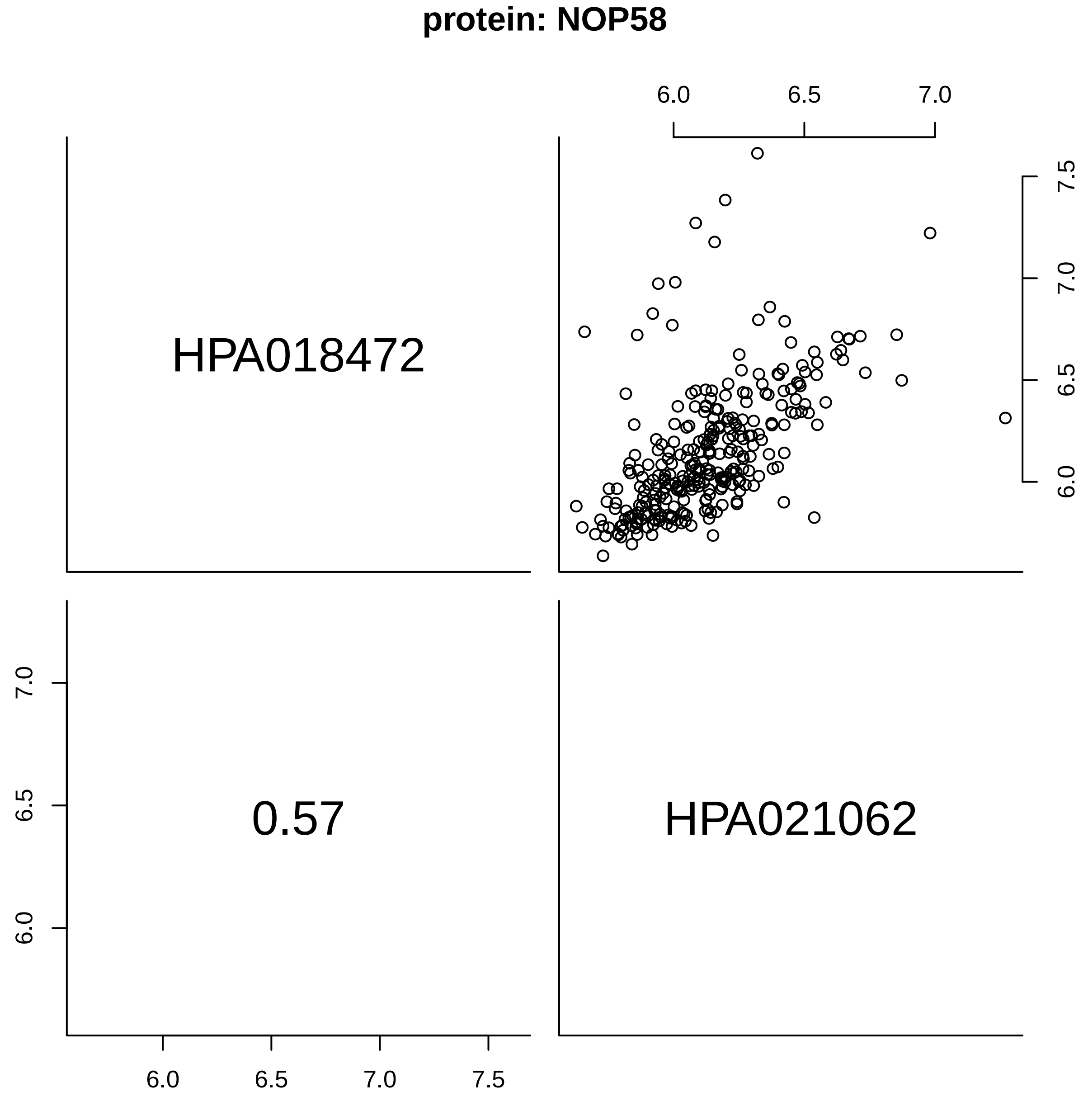

protein: NOP58
HPA018472
0.57
HPA021062
6.0
6.5
7.0
7.5

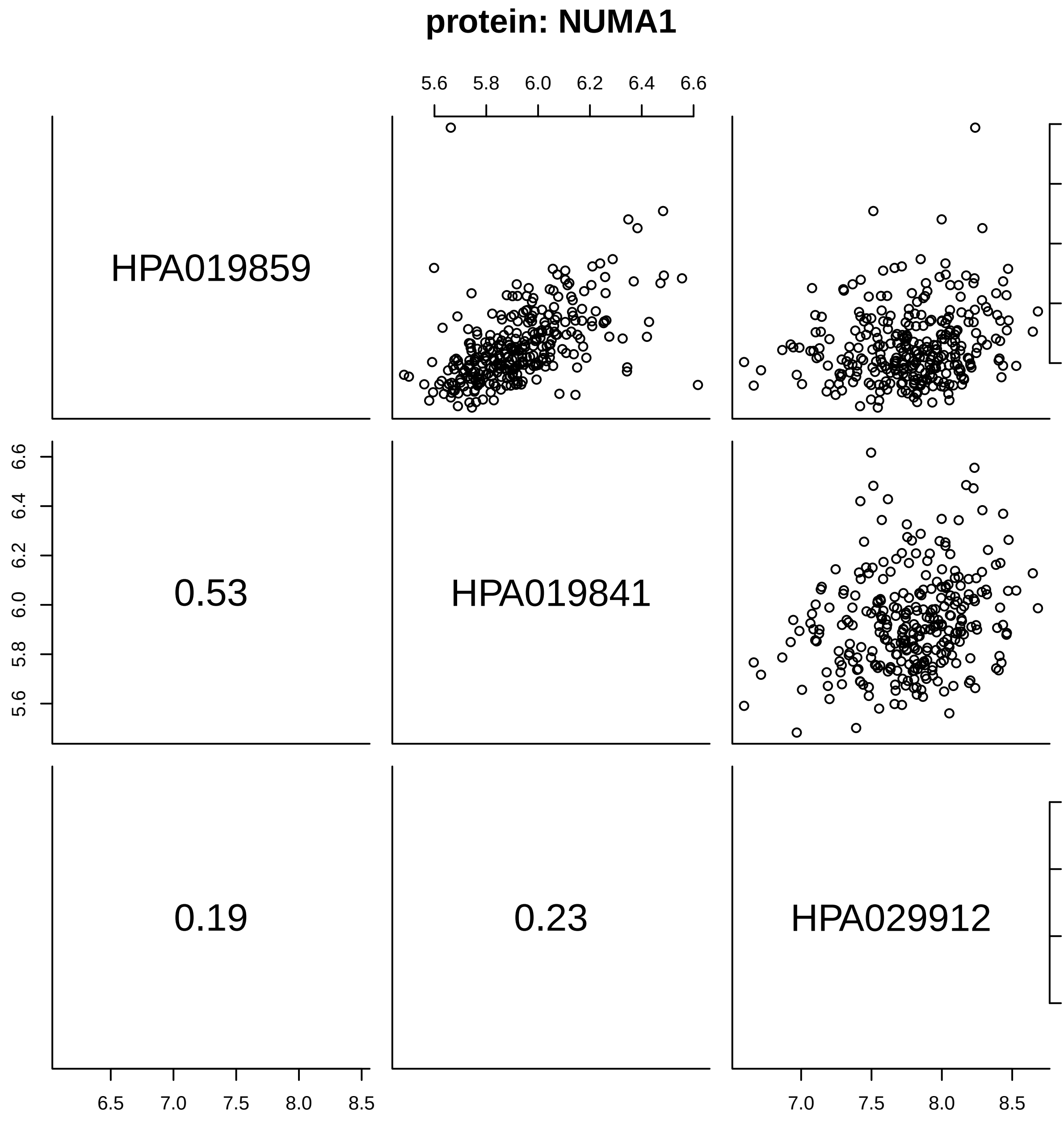
protein: NUMA1
HPA019859
HPA019841
HPA029912
0.53
0.19
0.23
5.6
5.8
6.0
6.2
6.4
6.6
6.5
7.0
7.5
8.0
8.5

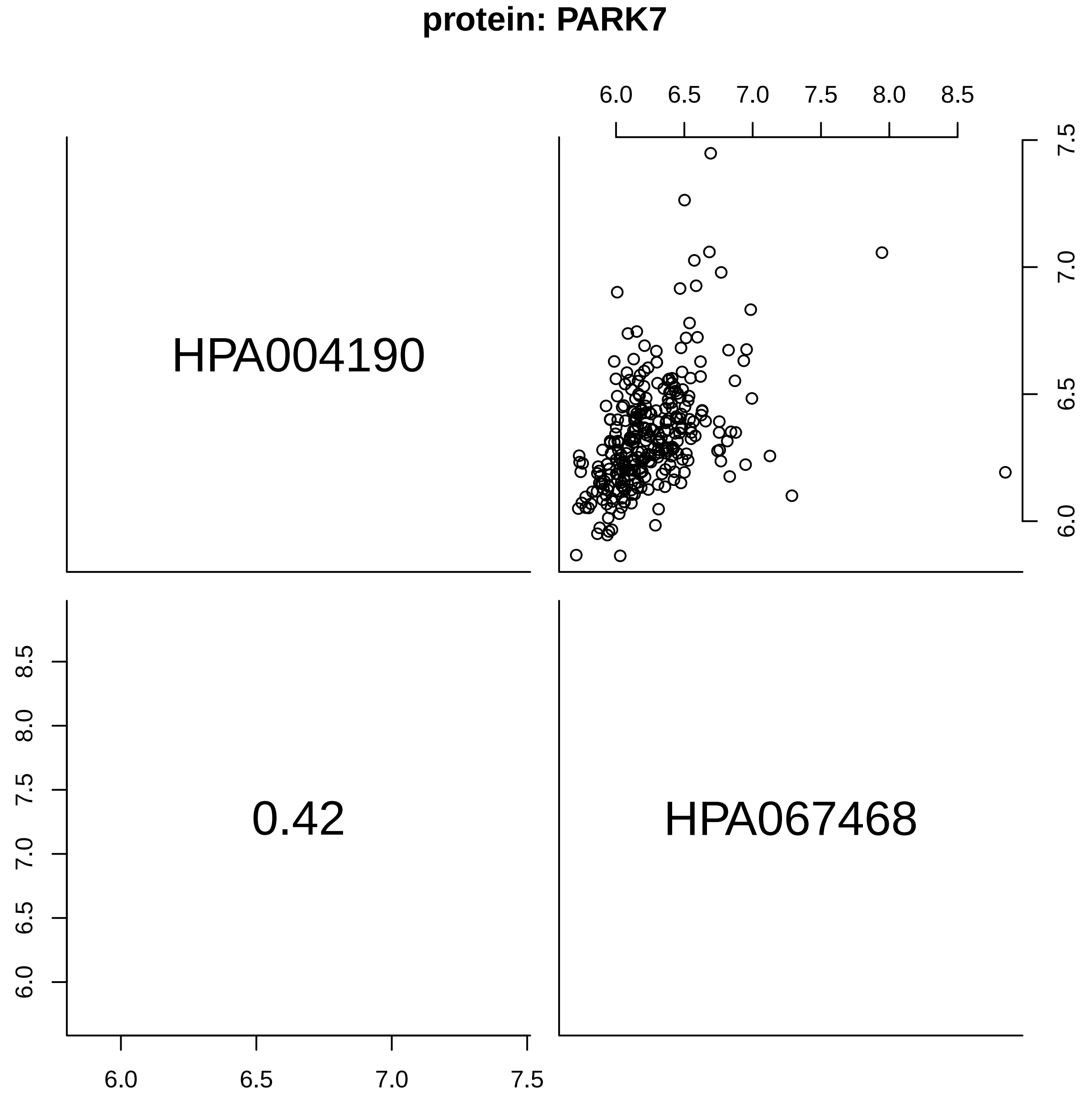

protein: PARK7
HPA004190
0.42
HPA067468
6.0
6.5
7.0
7.5
8.0
8.5

## protein: PCM1

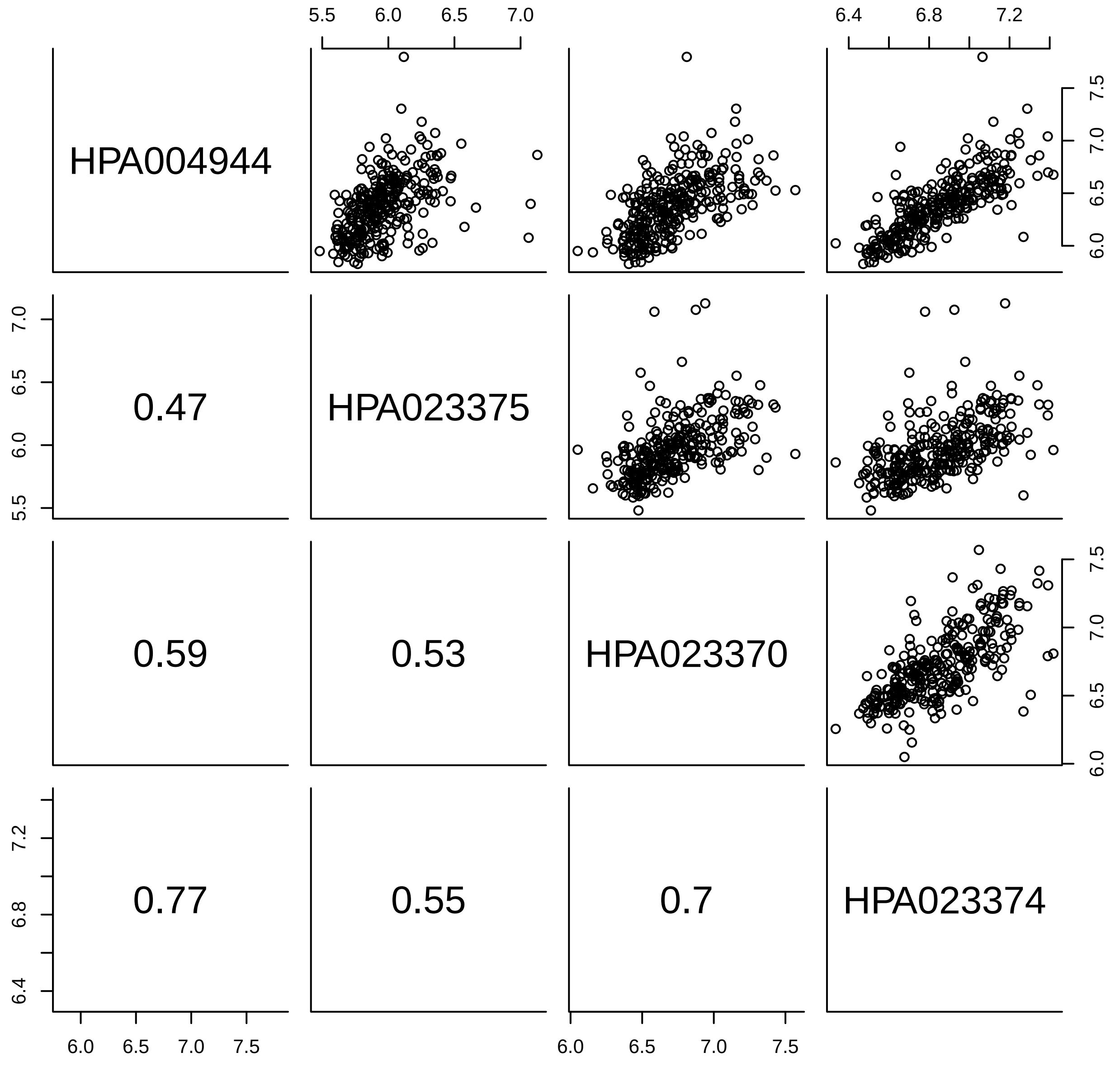

## protein: PCMT1

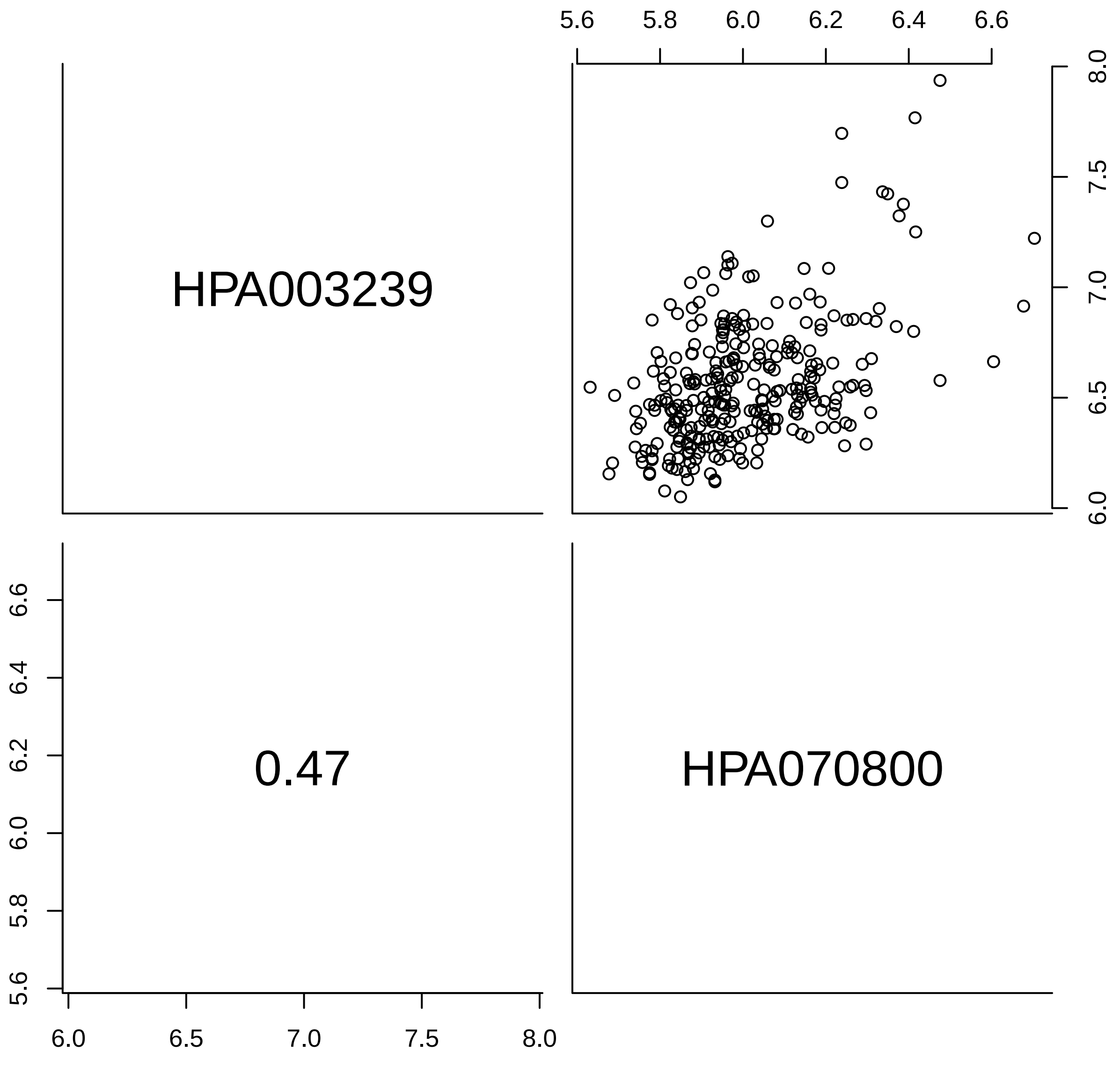

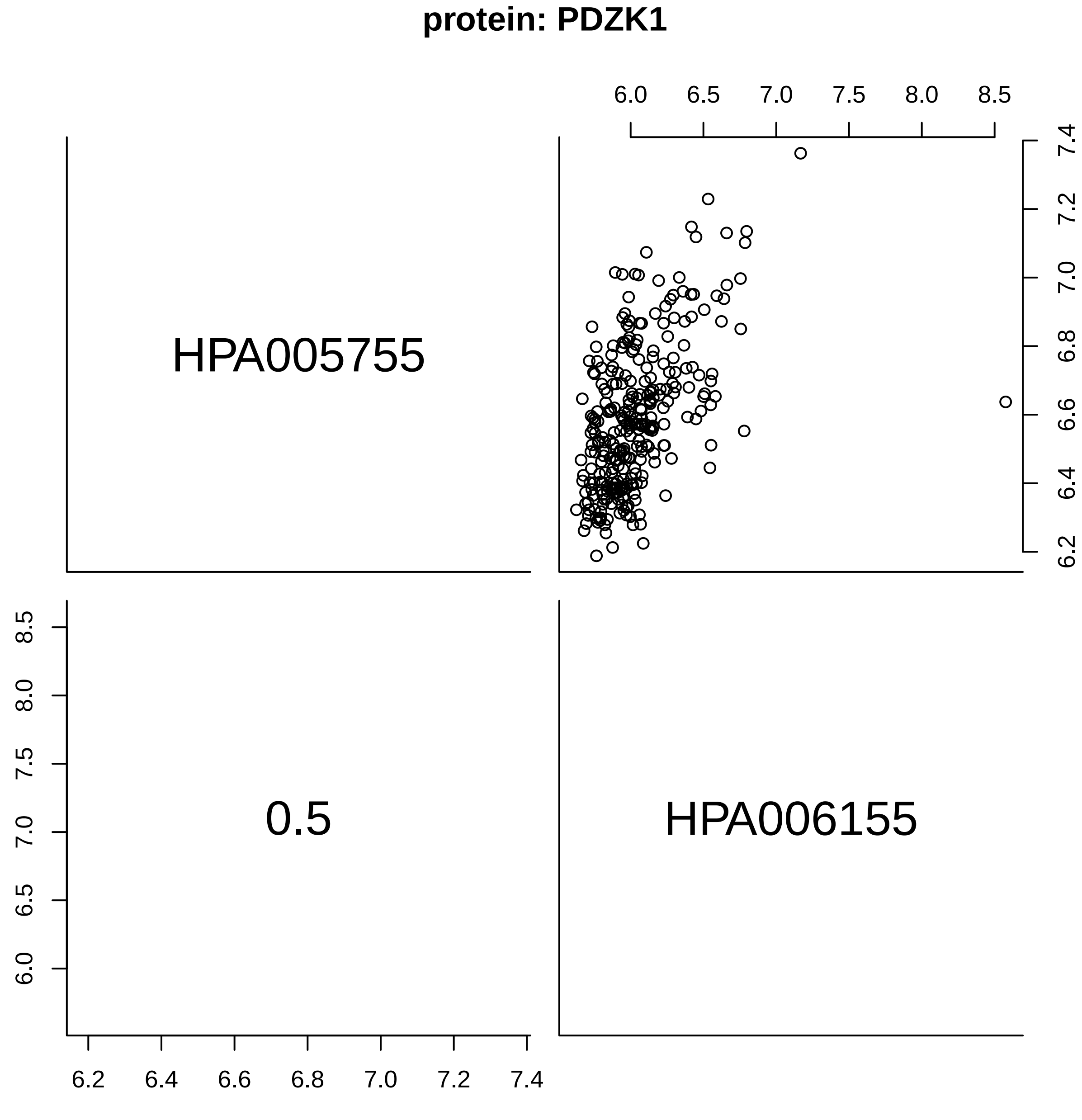

protein: PDZK1
HPA005755
0.5
HPA006155
6.0 6.5 7.0 7.5 8.0 8.5
6.2 6.4 6.6 6.8 7.0 7.2 7.4

## protein: PGM1

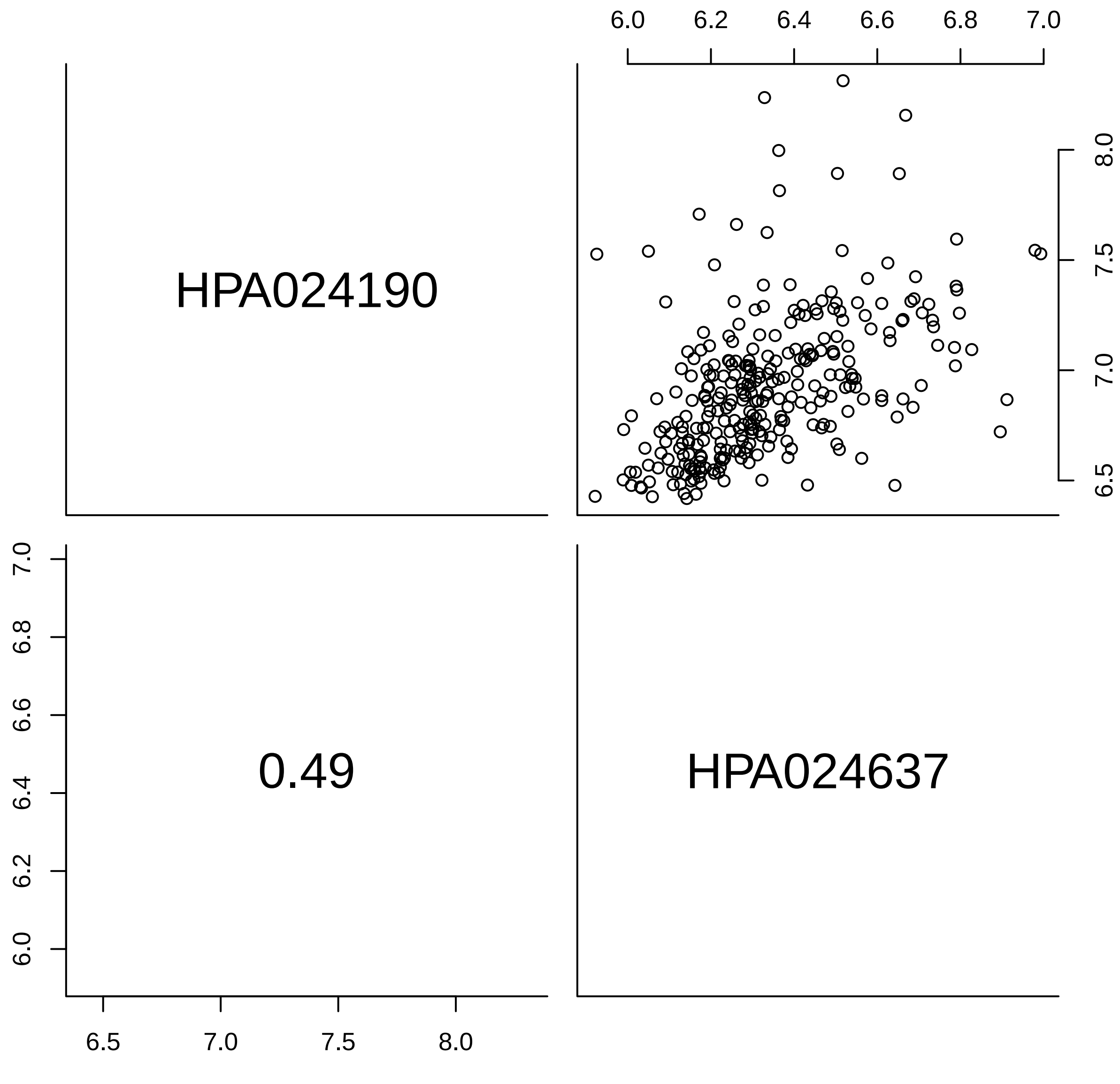

## protein: POLR2A

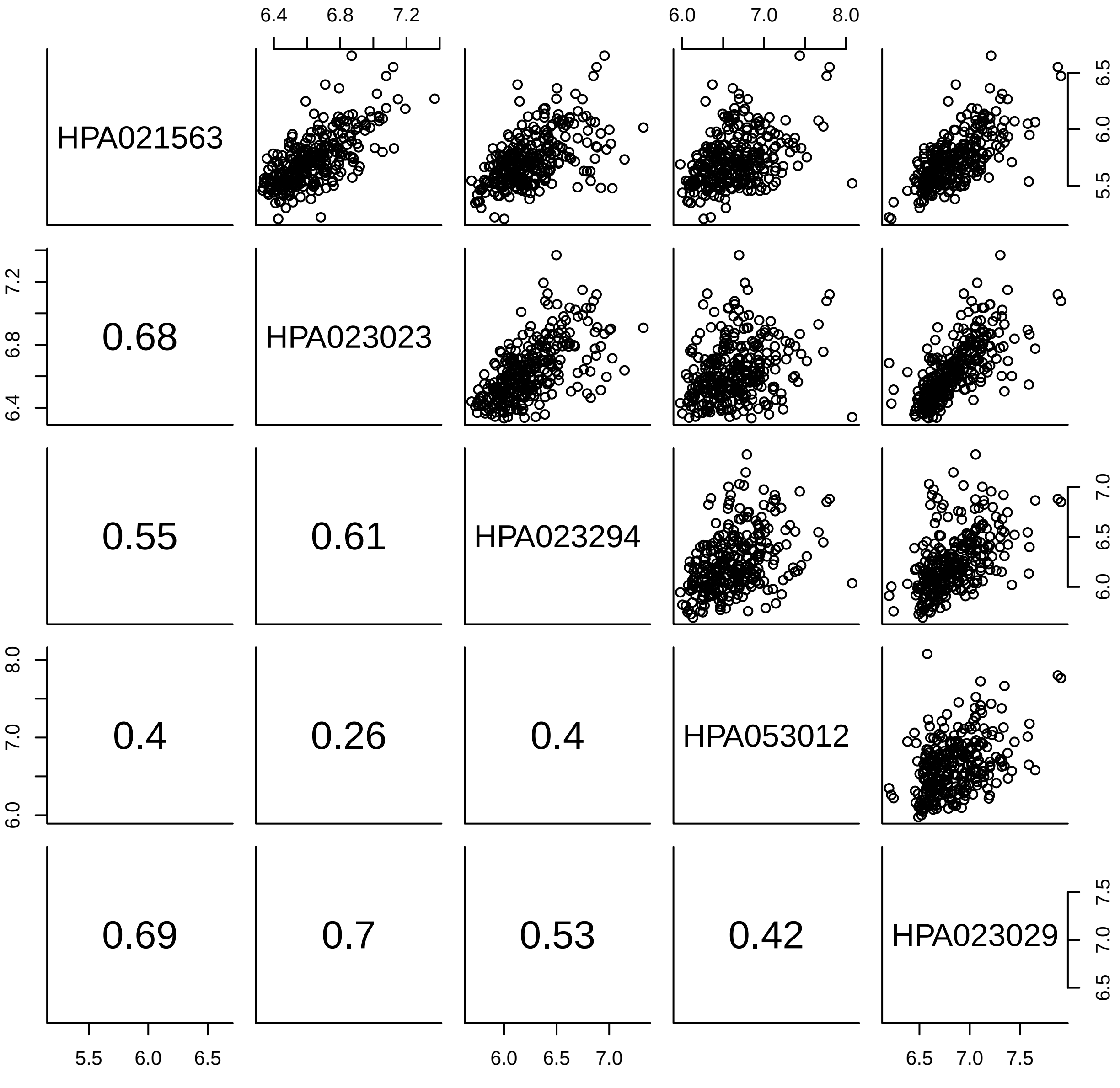

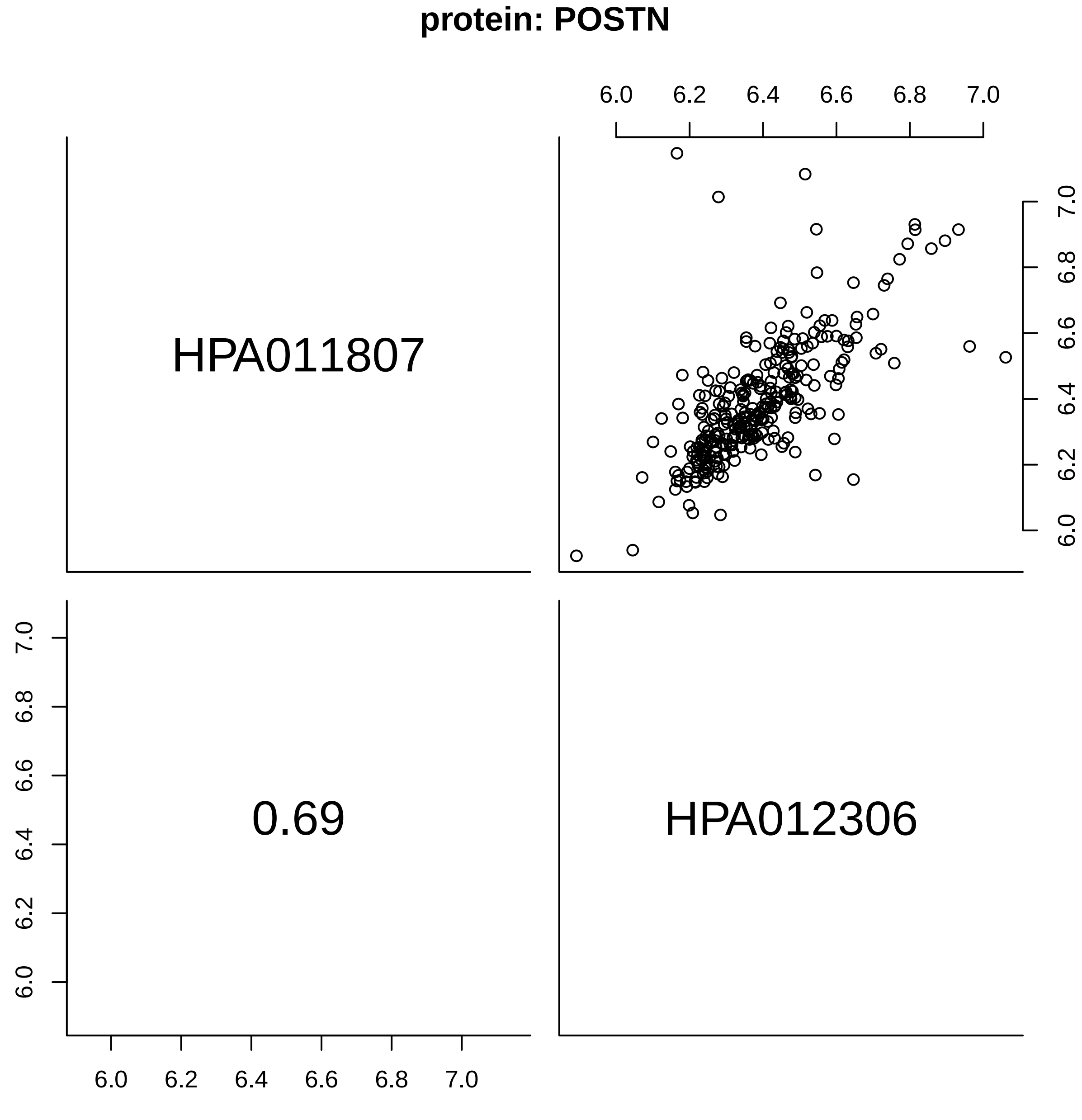

protein: POSTN
HPA011807
0.69
HPA012306
6.0
6.2
6.4
6.6
6.8
7.0

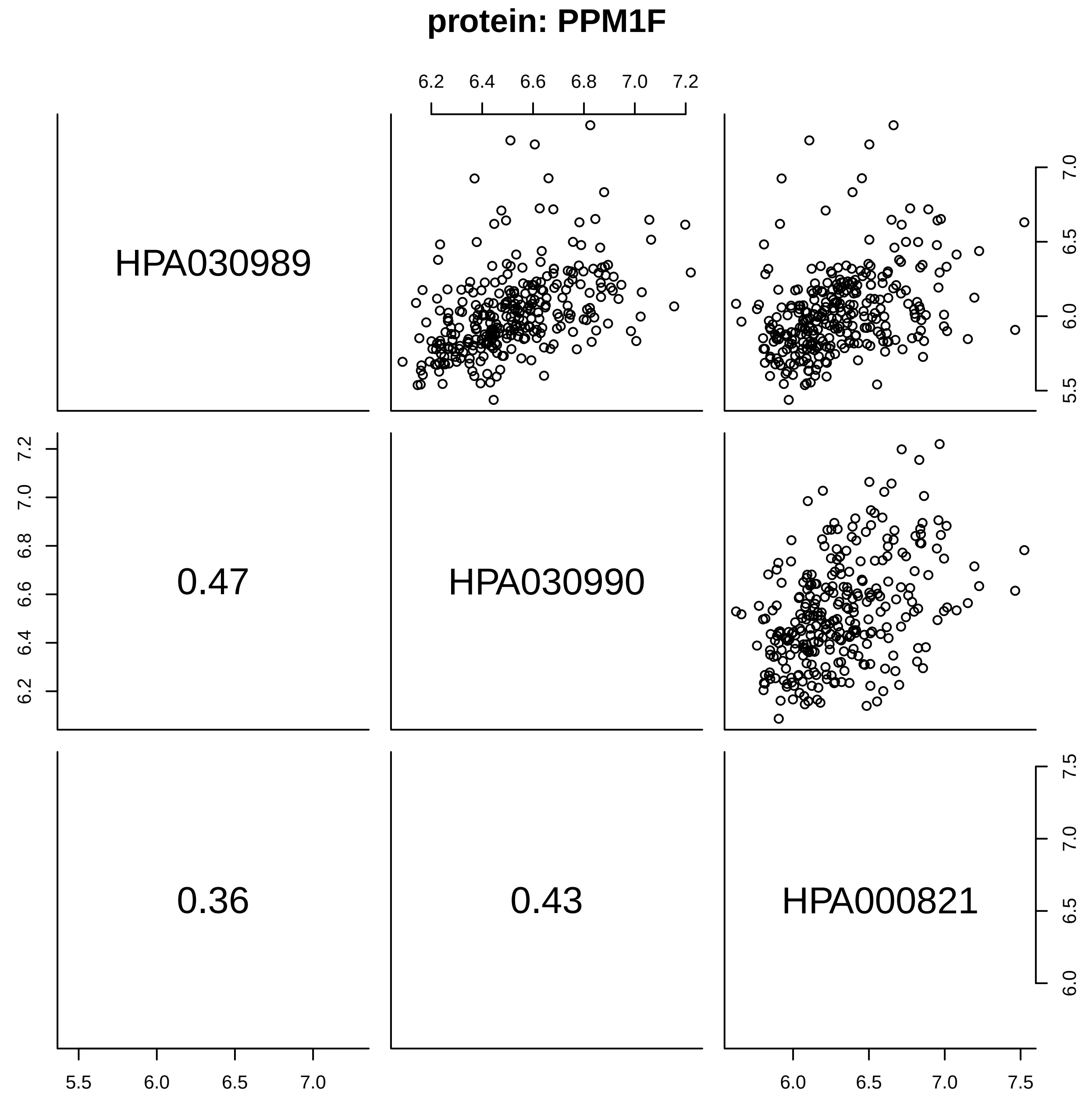
protein: PPM1F
HPA030989
HPA030990
HPA000821
0.47
0.36
0.43
6.2 6.4 6.6 6.8 7.0 7.2
5.5 6.0 6.5 7.0
6.0 6.5 7.0 7.5

## protein: PTCD3

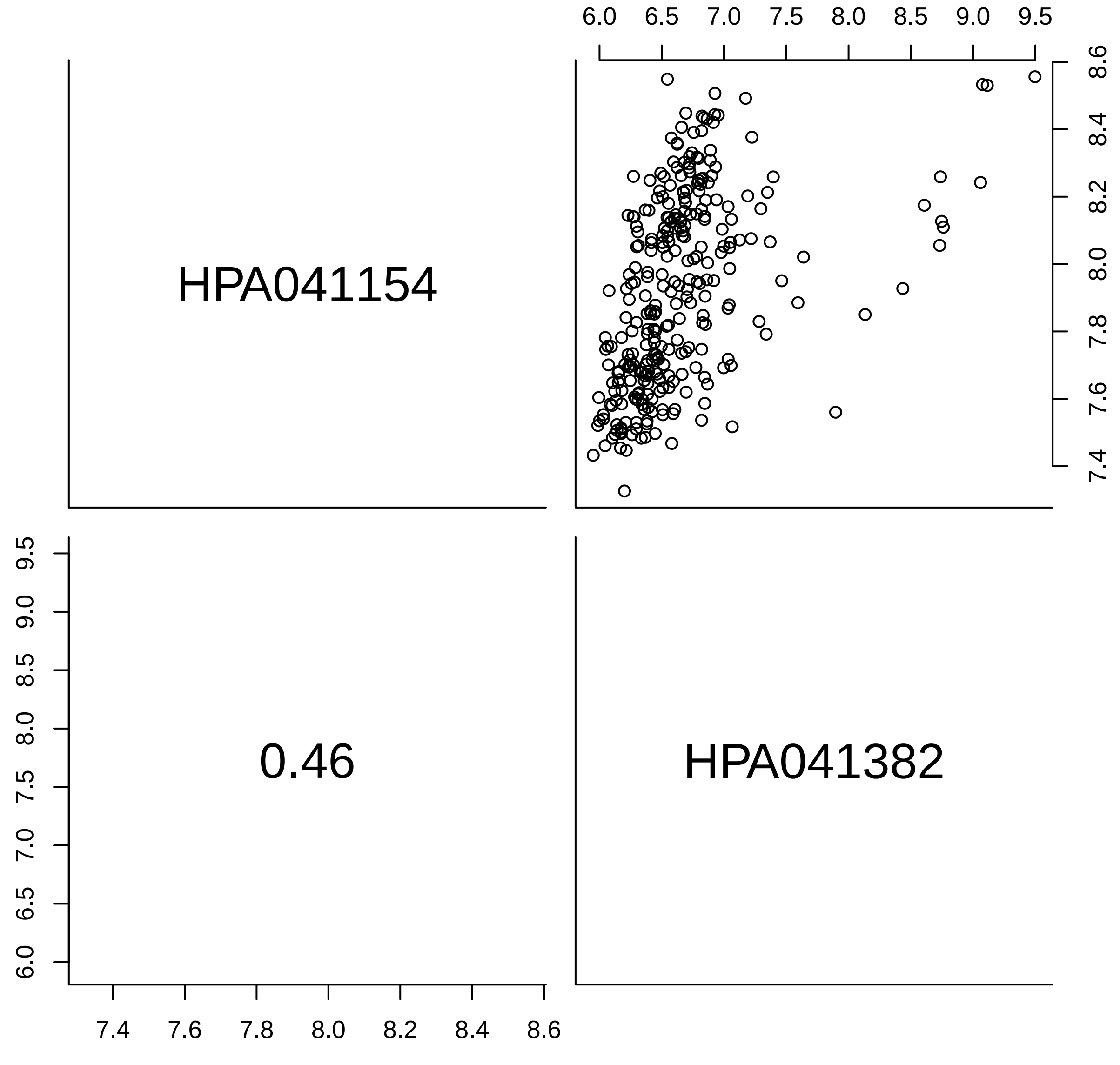

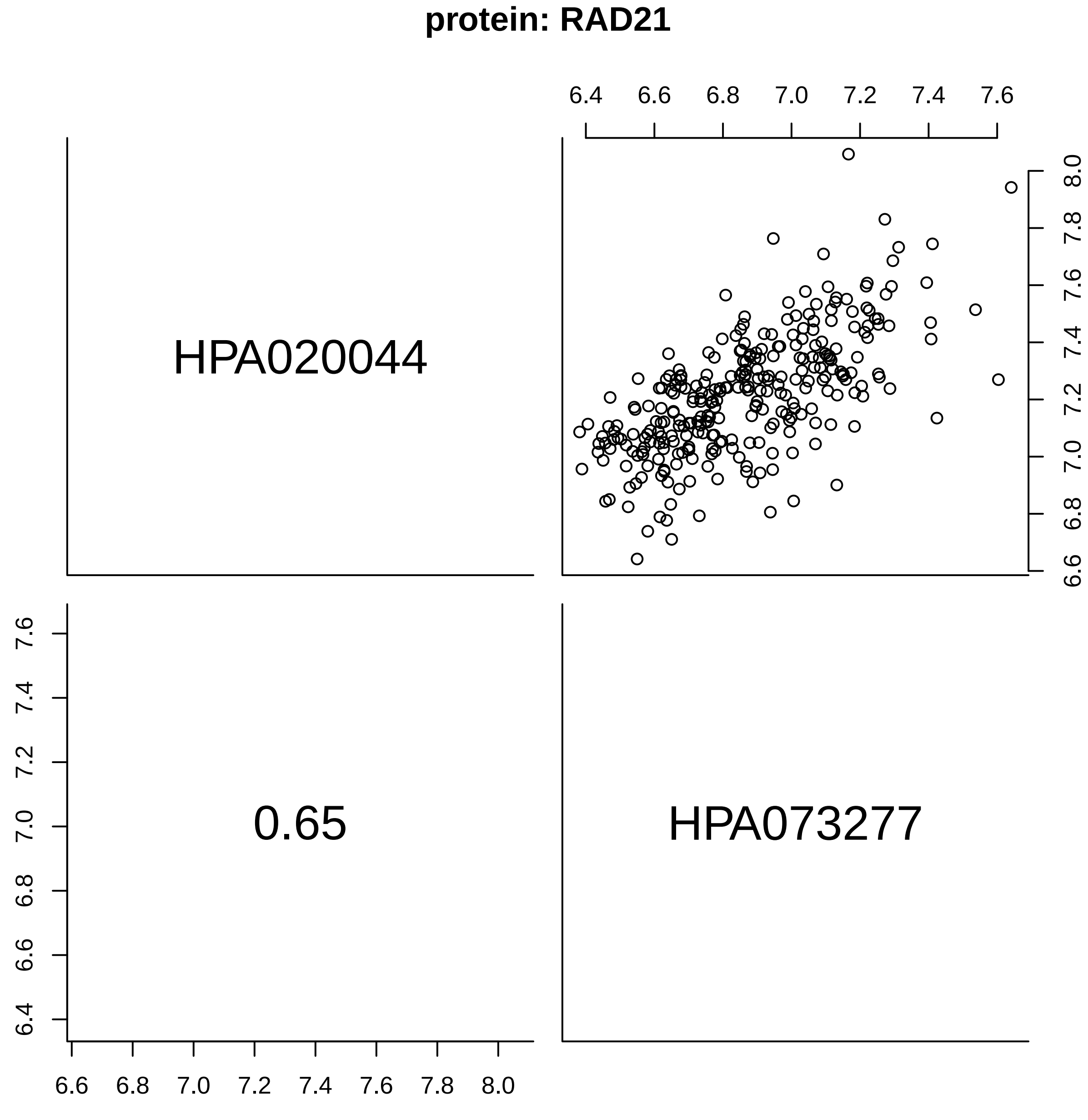

protein: RAD21
HPA020044
0.65
HPA073277
6.4
6.6
6.8
7.0
7.2
7.4
7.6
8.0
7.8

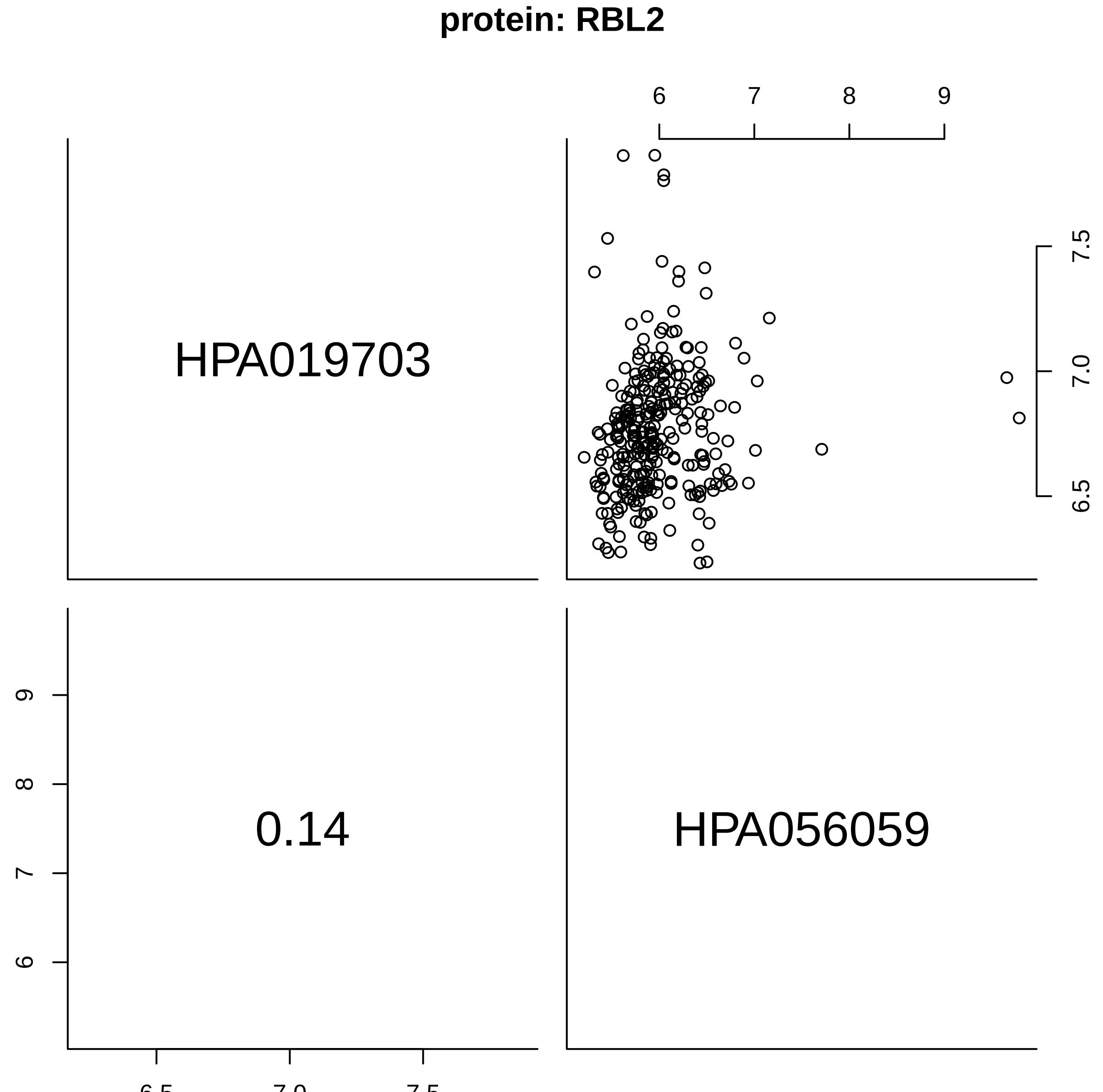

protein: RBL2
HPA019703
0.14
HPA056059
6
7
8
9
6.5
7.0
7.5

# protein: RBM6

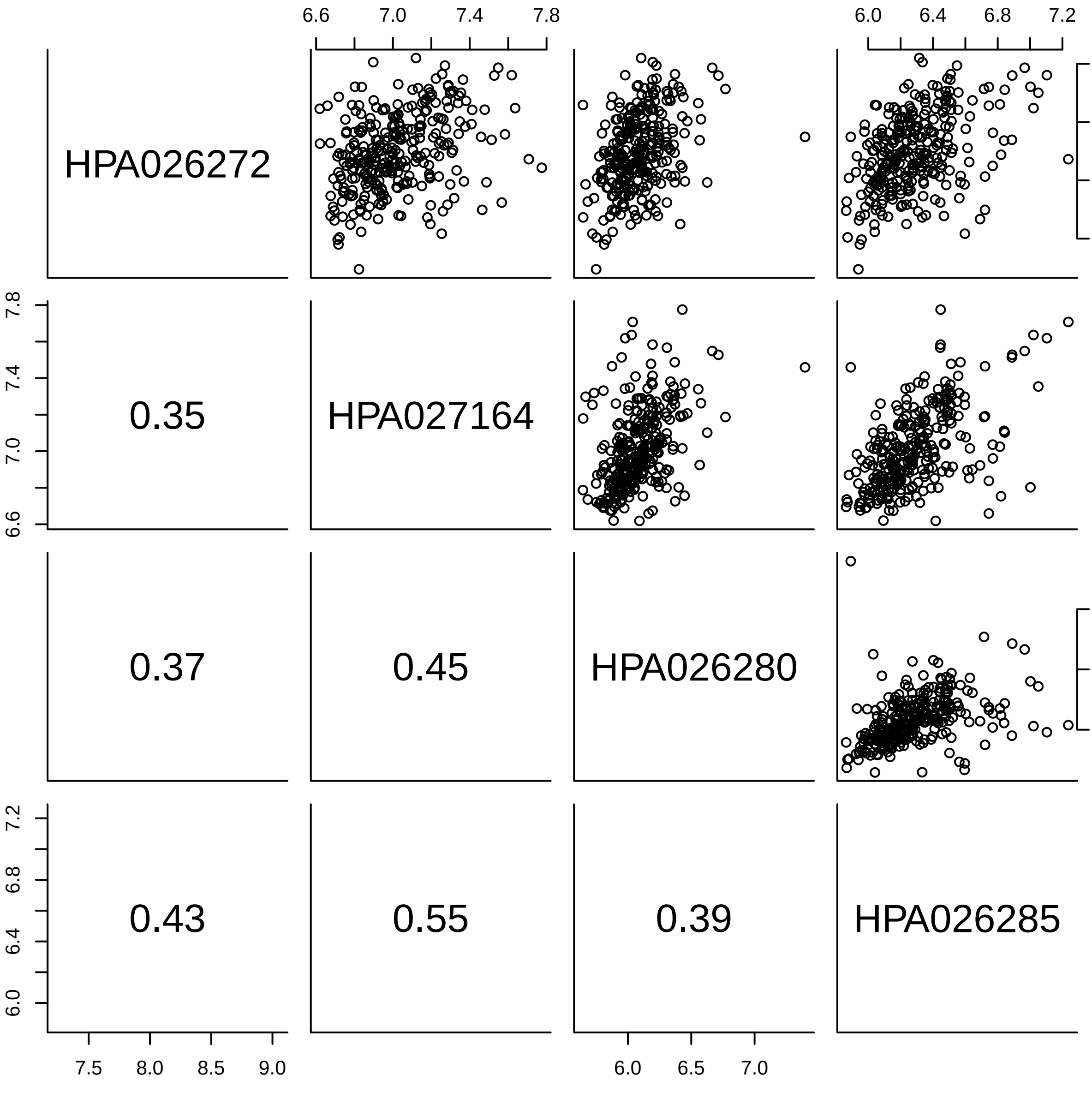

# protein: RELB

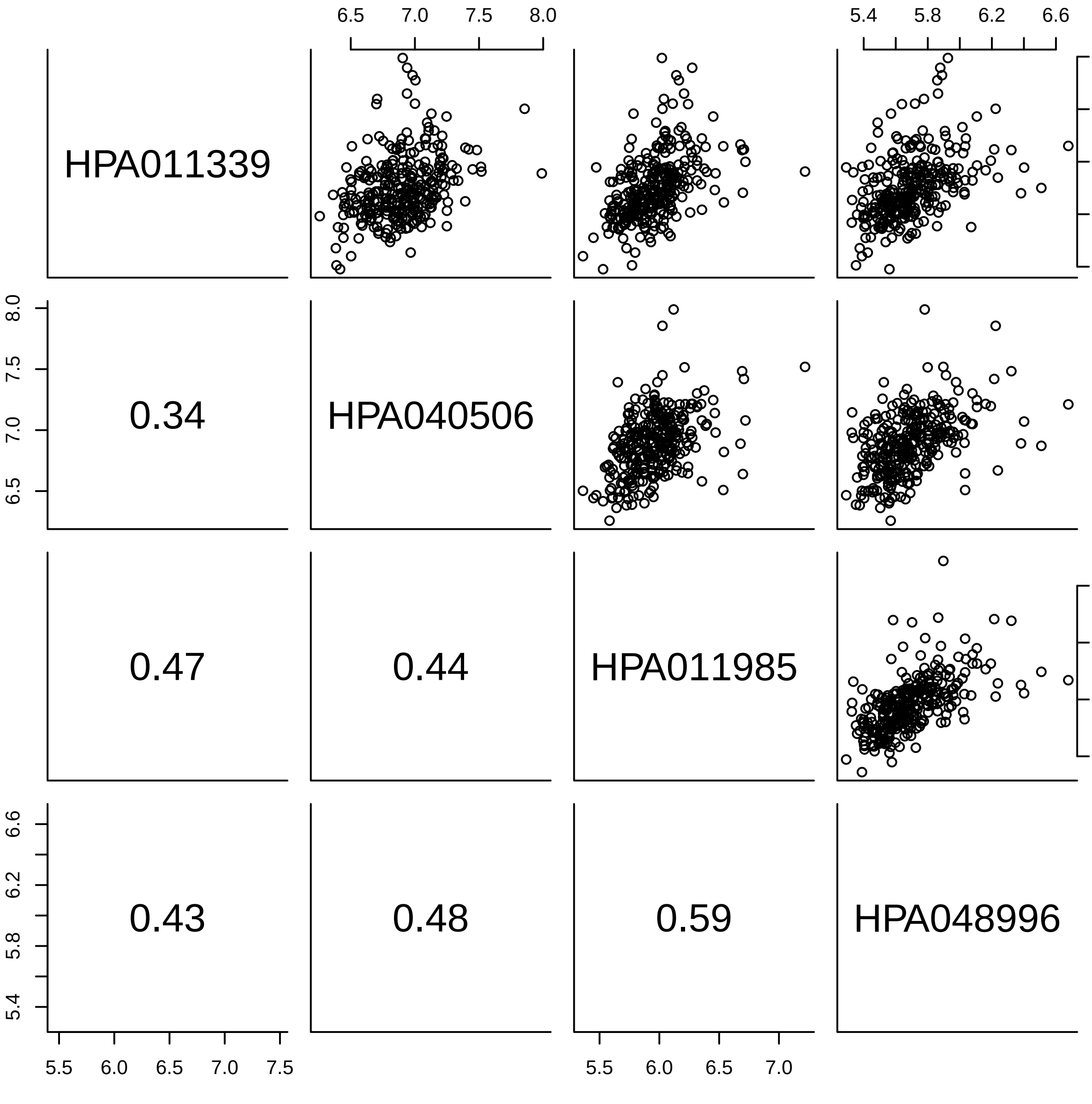

## protein: RTN3

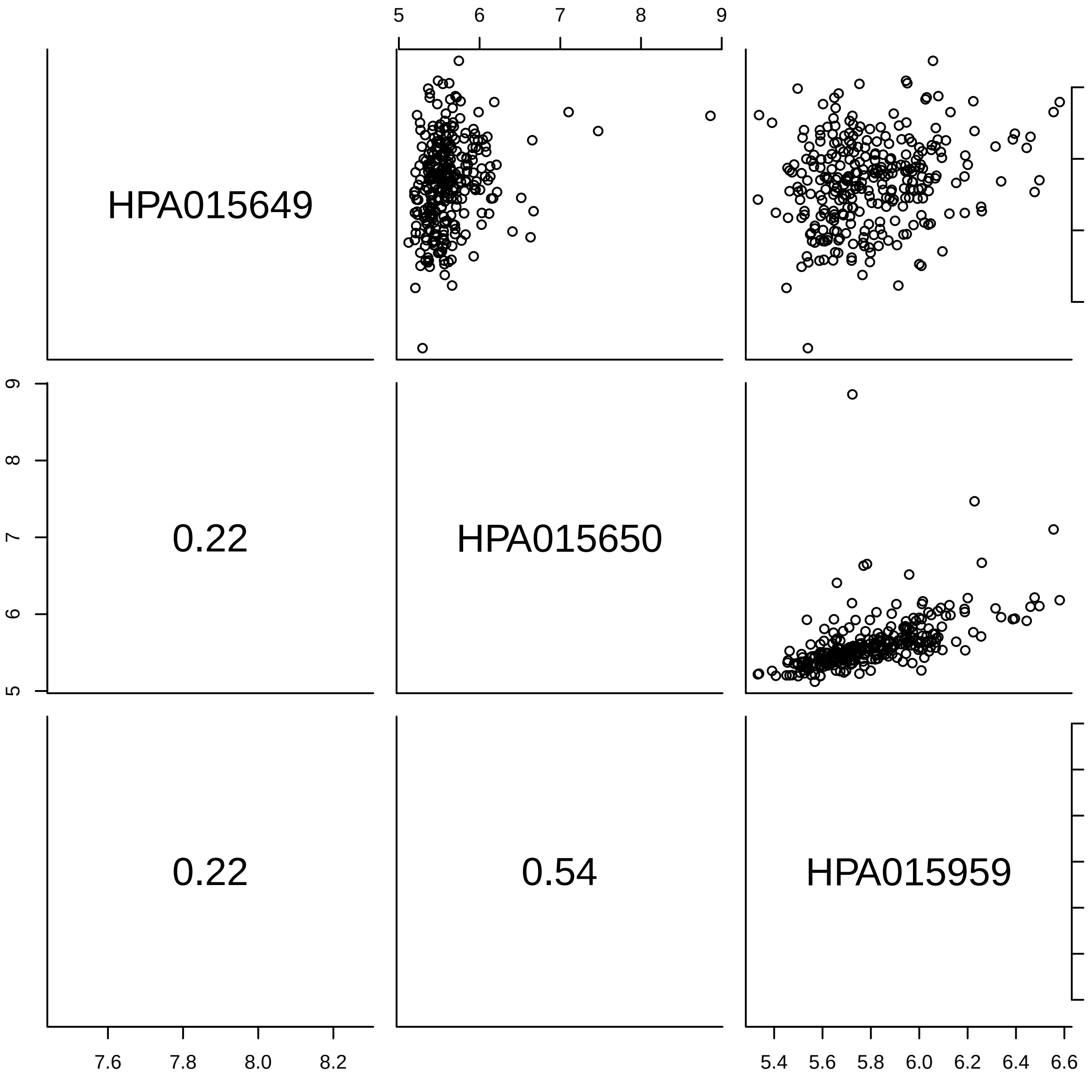

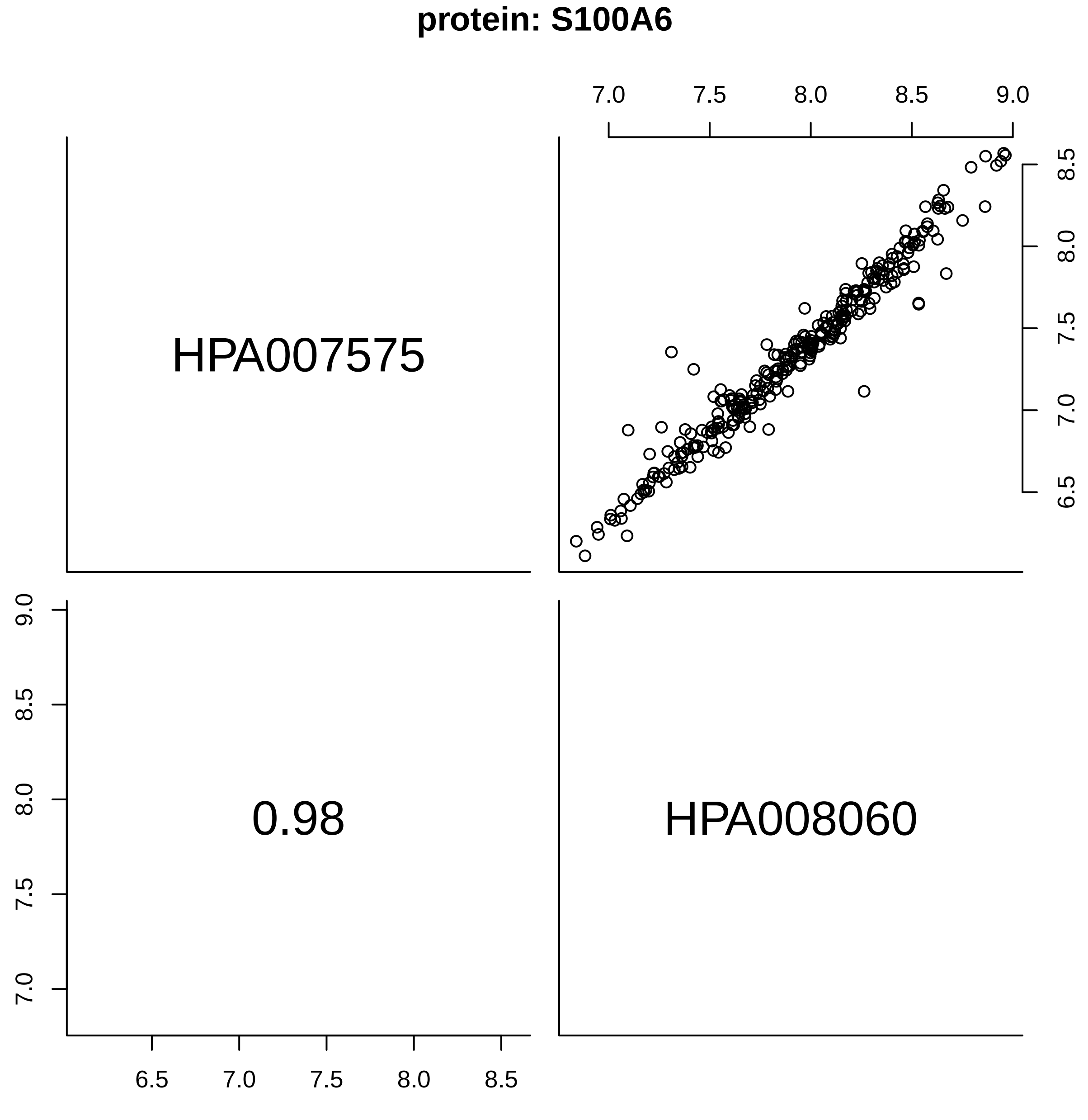
protein: S100A6
HPA007575
0.98
HPA008060
7.0 7.5 8.0 8.5 9.0
6.5 7.0 7.5 8.0 8.5
7.0 7.5 8.0 8.5 9.0
6.5 7.0 7.5 8.0 8.5

## protein: SCAF4

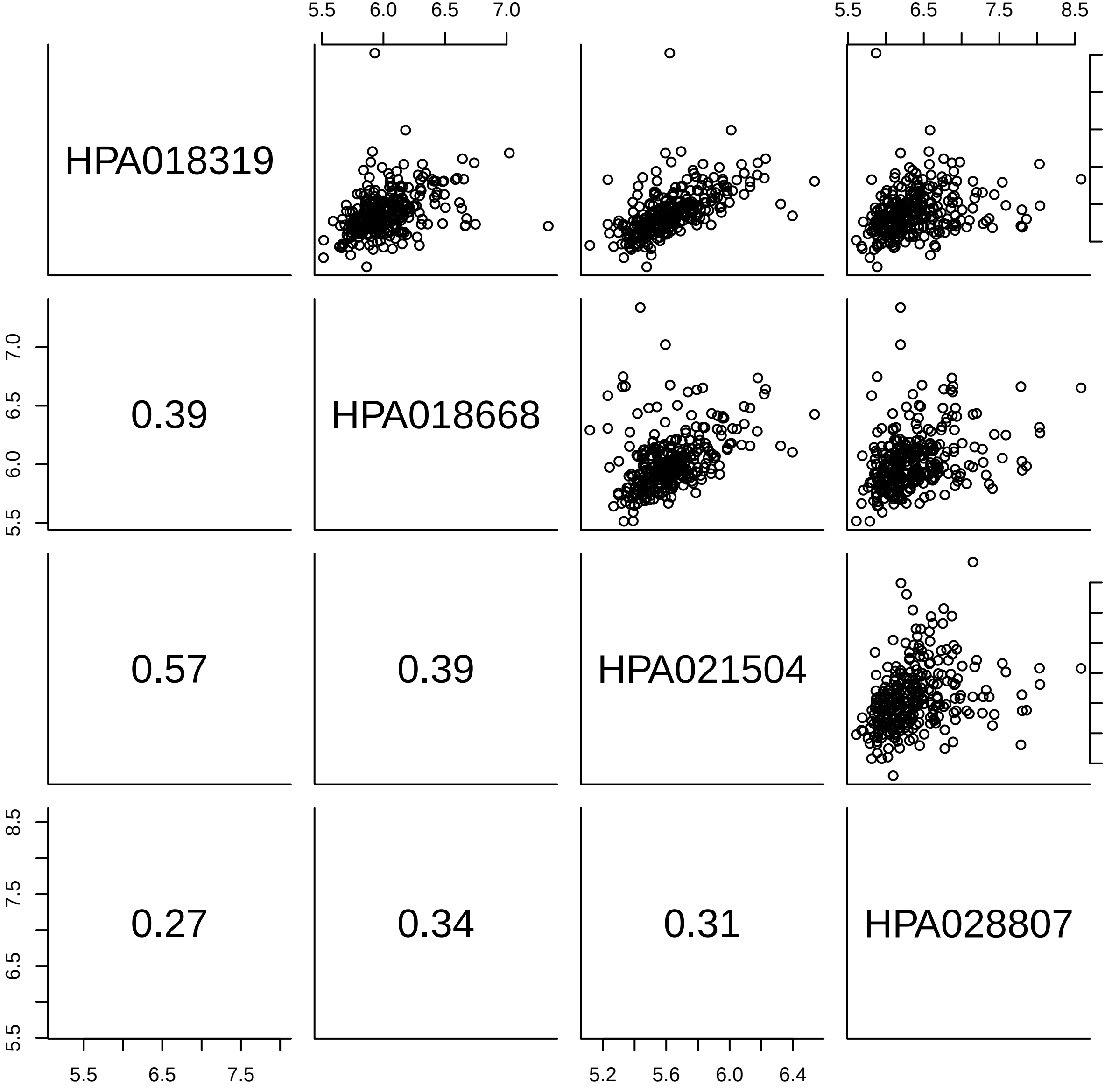

## protein: SERPINA1

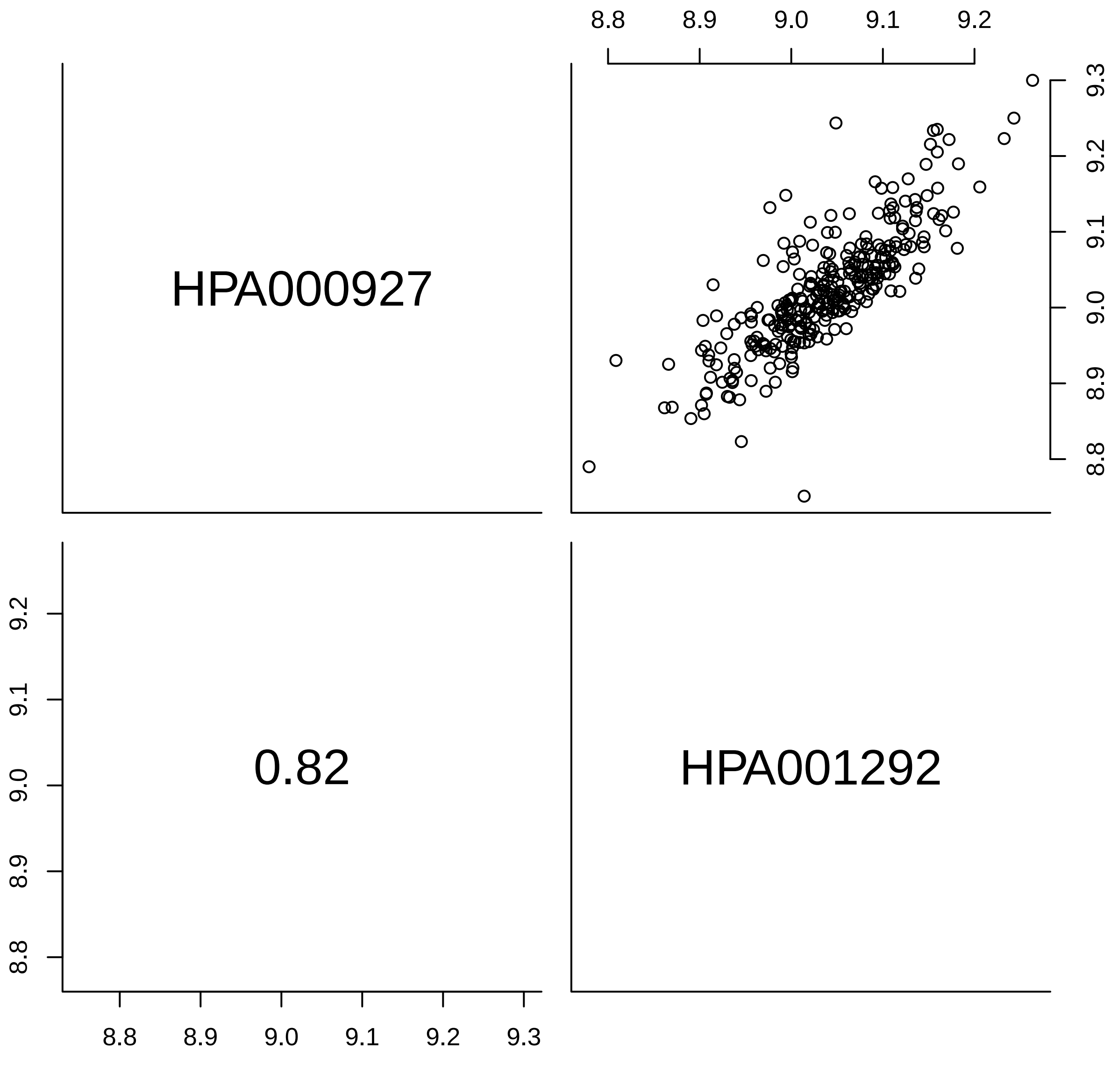

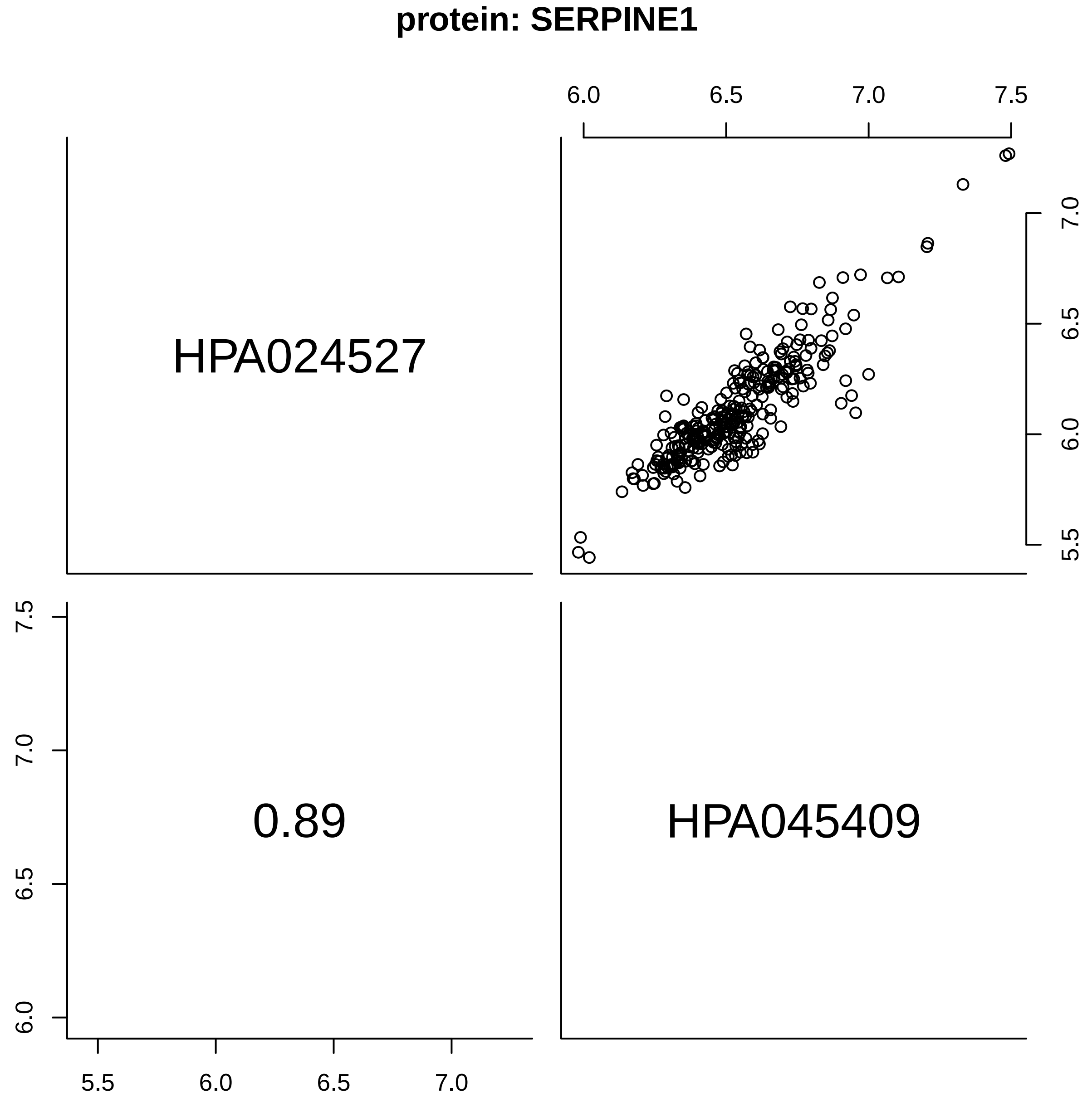
protein: SERPINE1
HPA024527
0.89
HPA045409
6.0 6.5 7.0 7.5
5.5 6.0 6.5 7.0

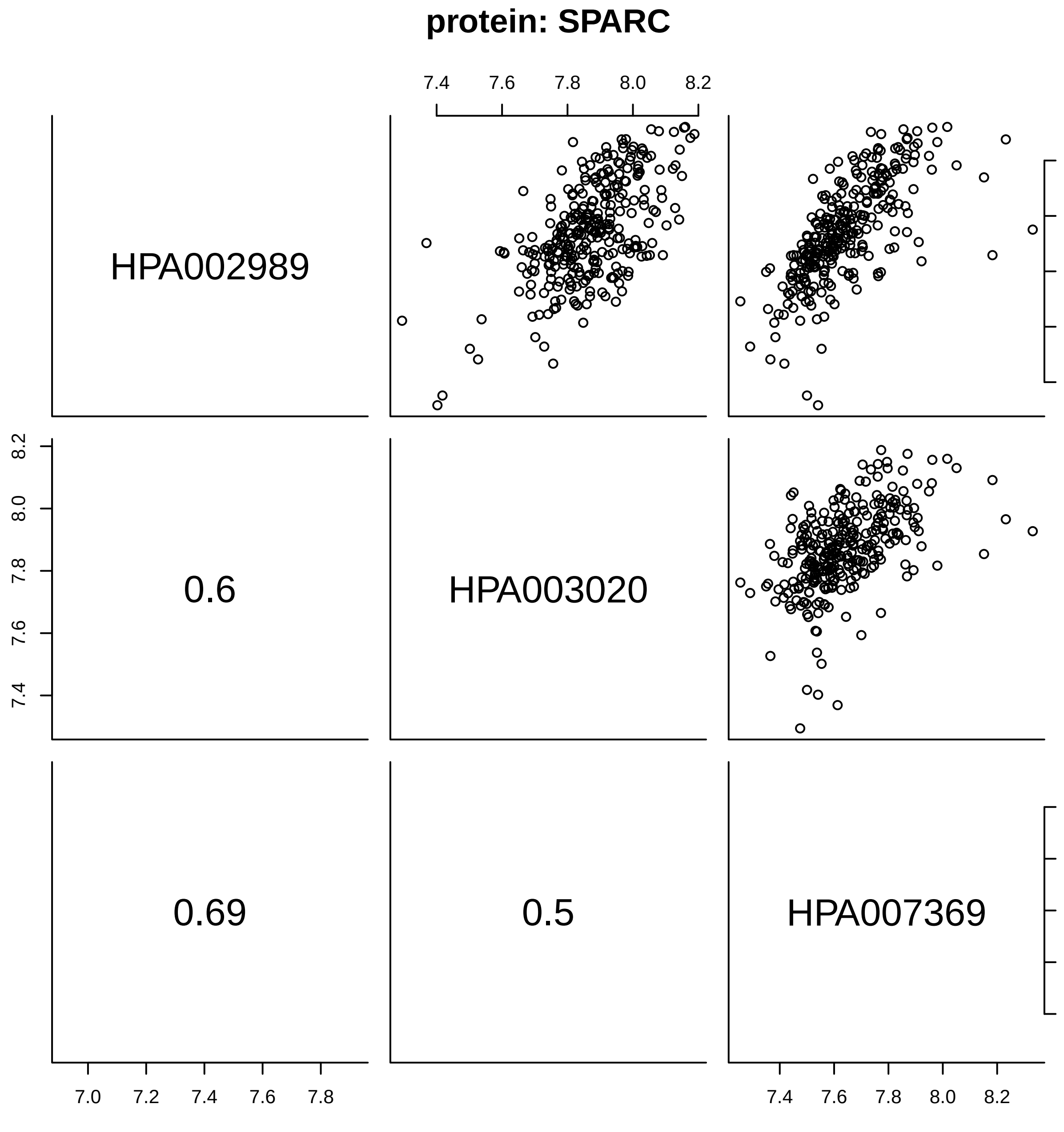
protein: SPARC
HPA002989
HPA003020
HPA007369
0.6
0.69
0.5
7.4 7.6 7.8 8.0 8.2
7.0 7.2 7.4 7.6 7.8
7.4 7.6 7.8 8.0 8.2

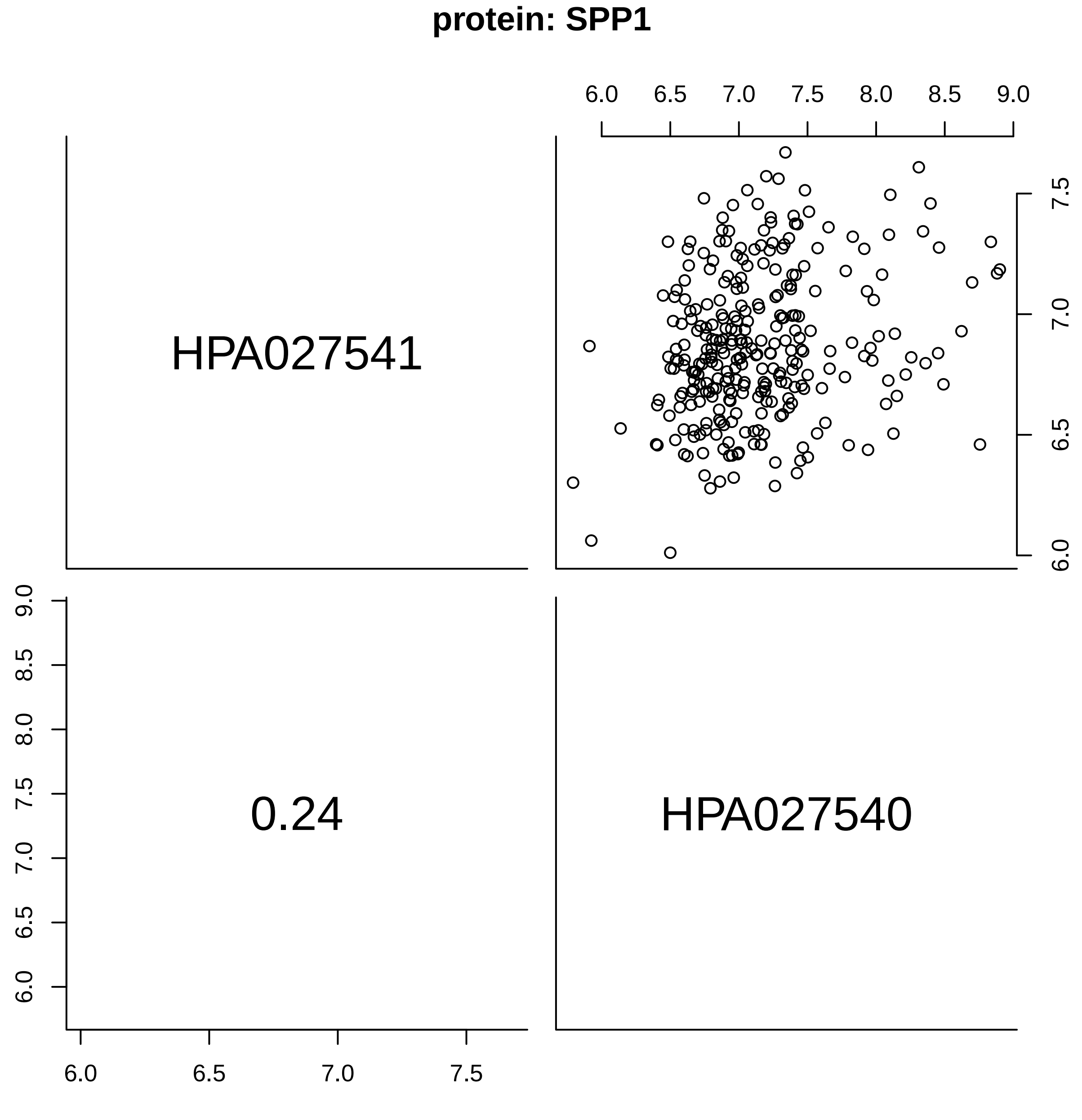
protein: SPP1
HPA027541
HPA027540
0.24
6.0
6.5
7.0
7.5
8.0
8.5
9.0

# protein: STUB1

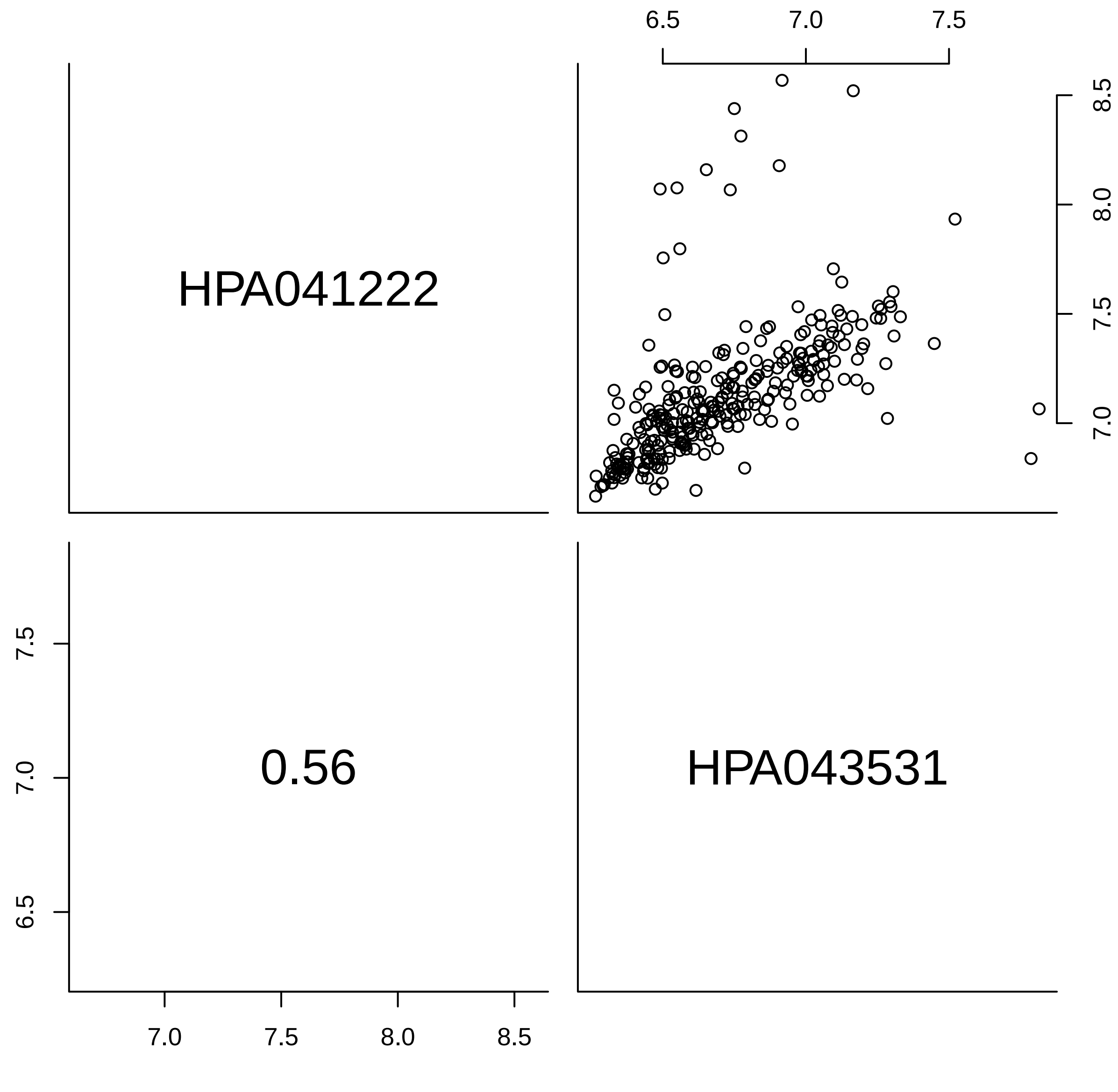

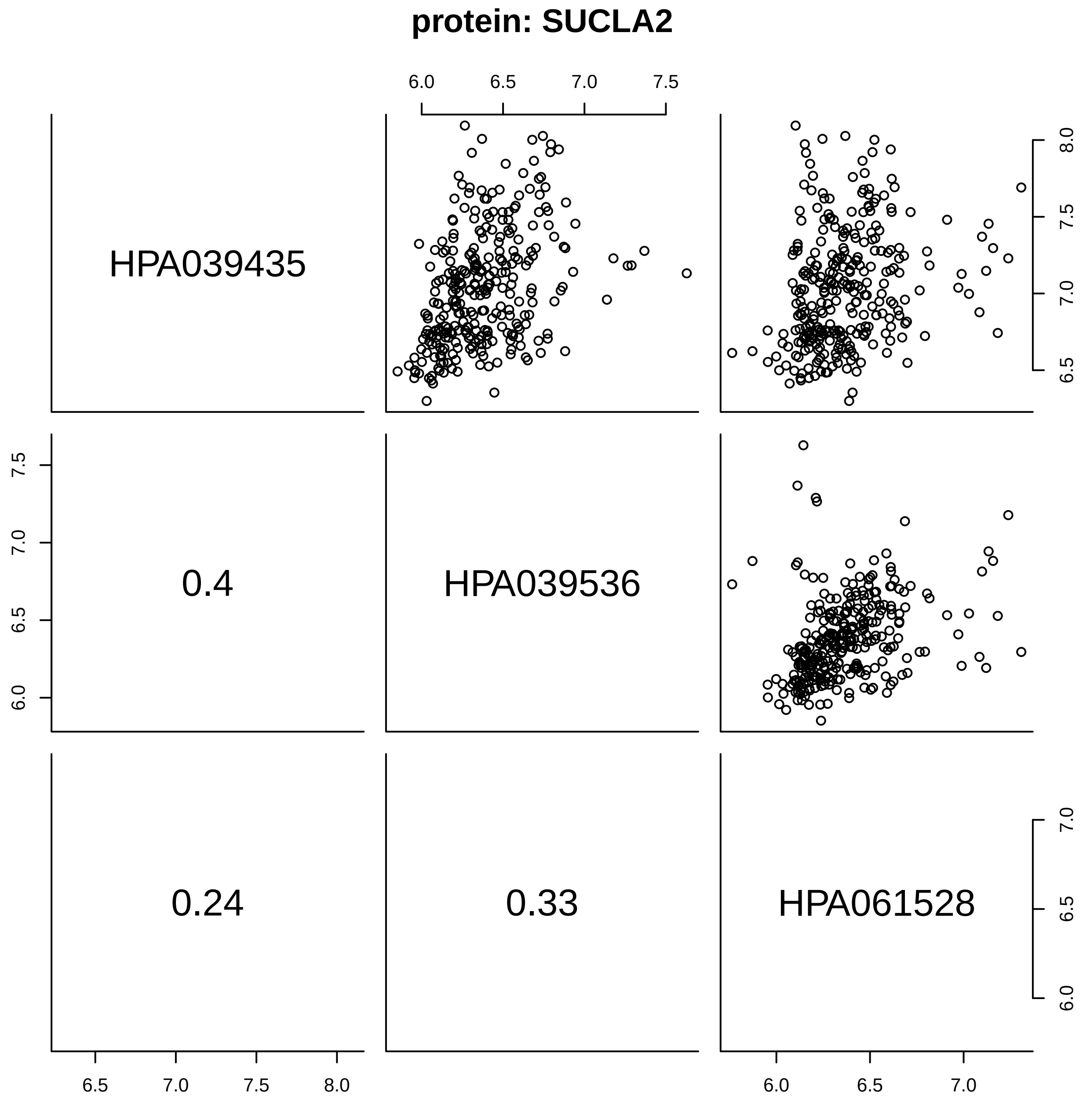

protein: SUCLA2
HPA039435
HPA039536
HPA061528
0.4
0.24
0.33
6.0
6.5
7.0
7.5
8.0

# protein: SUN1

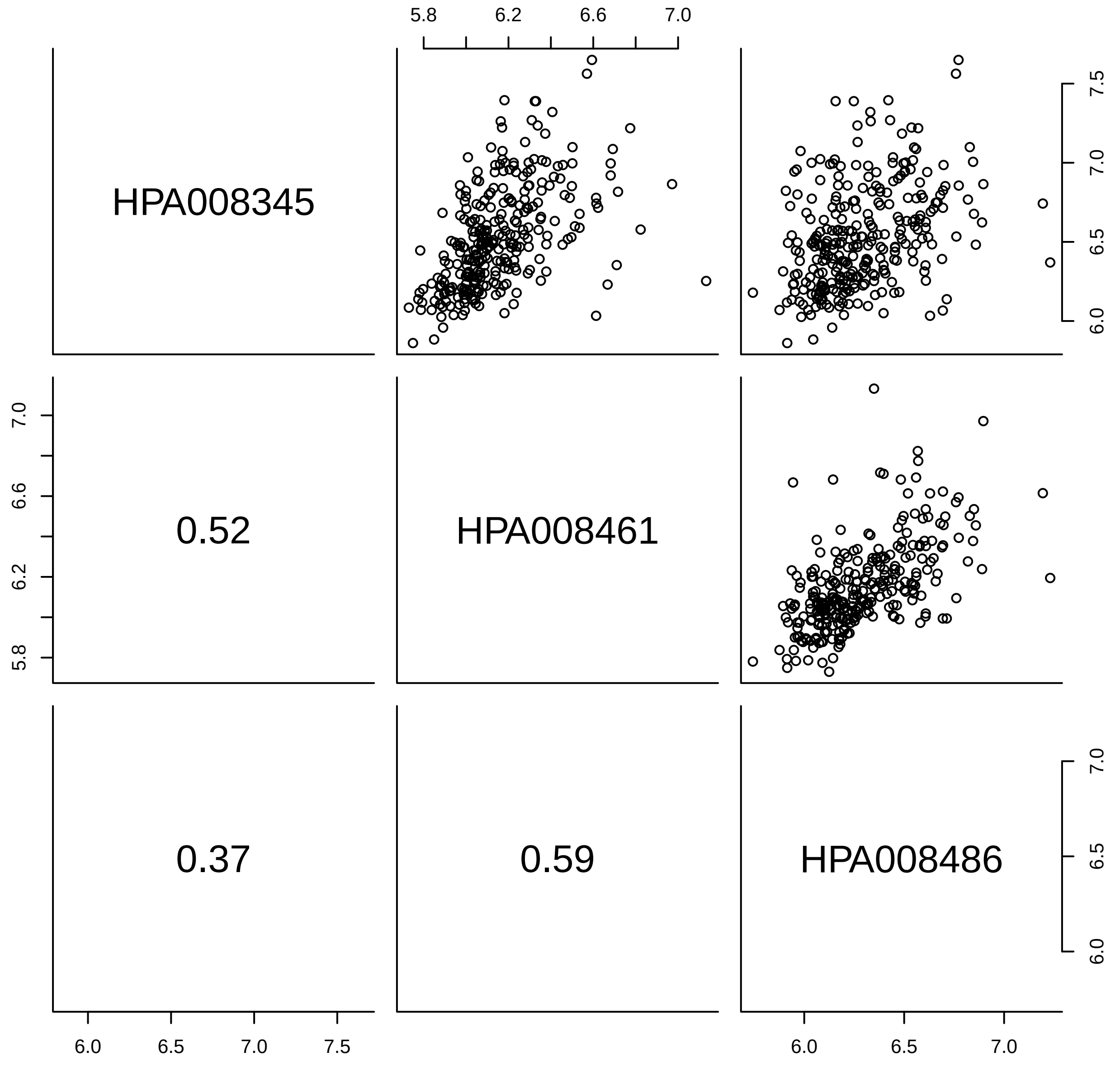

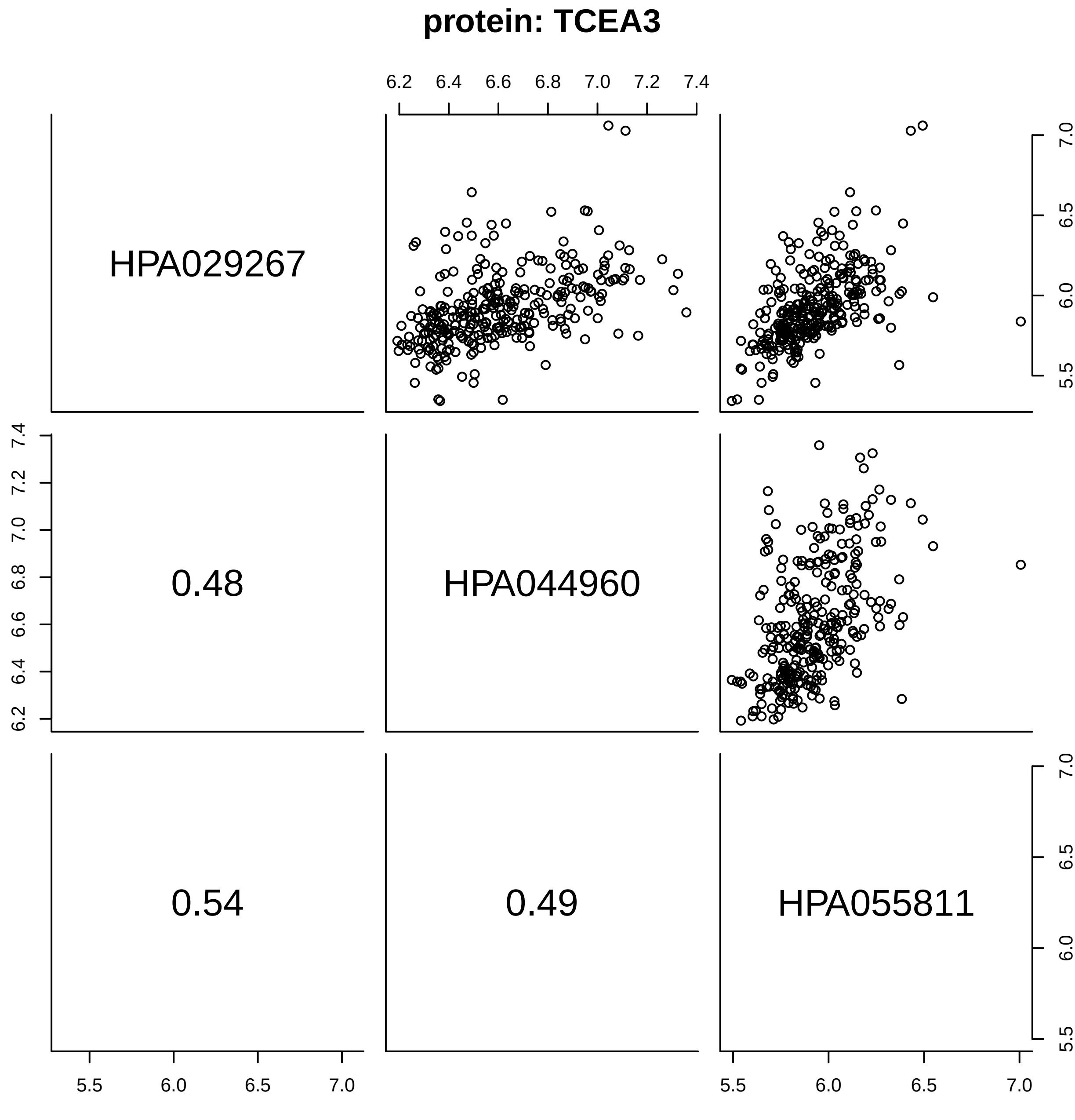
protein: TCEA3
HPA029267
HPA044960
HPA055811
0.48
0.54
0.49
6.2
6.4
6.6
6.8
7.0
7.2
7.4
5.5
6.0
6.5
7.0

**protein: TF**

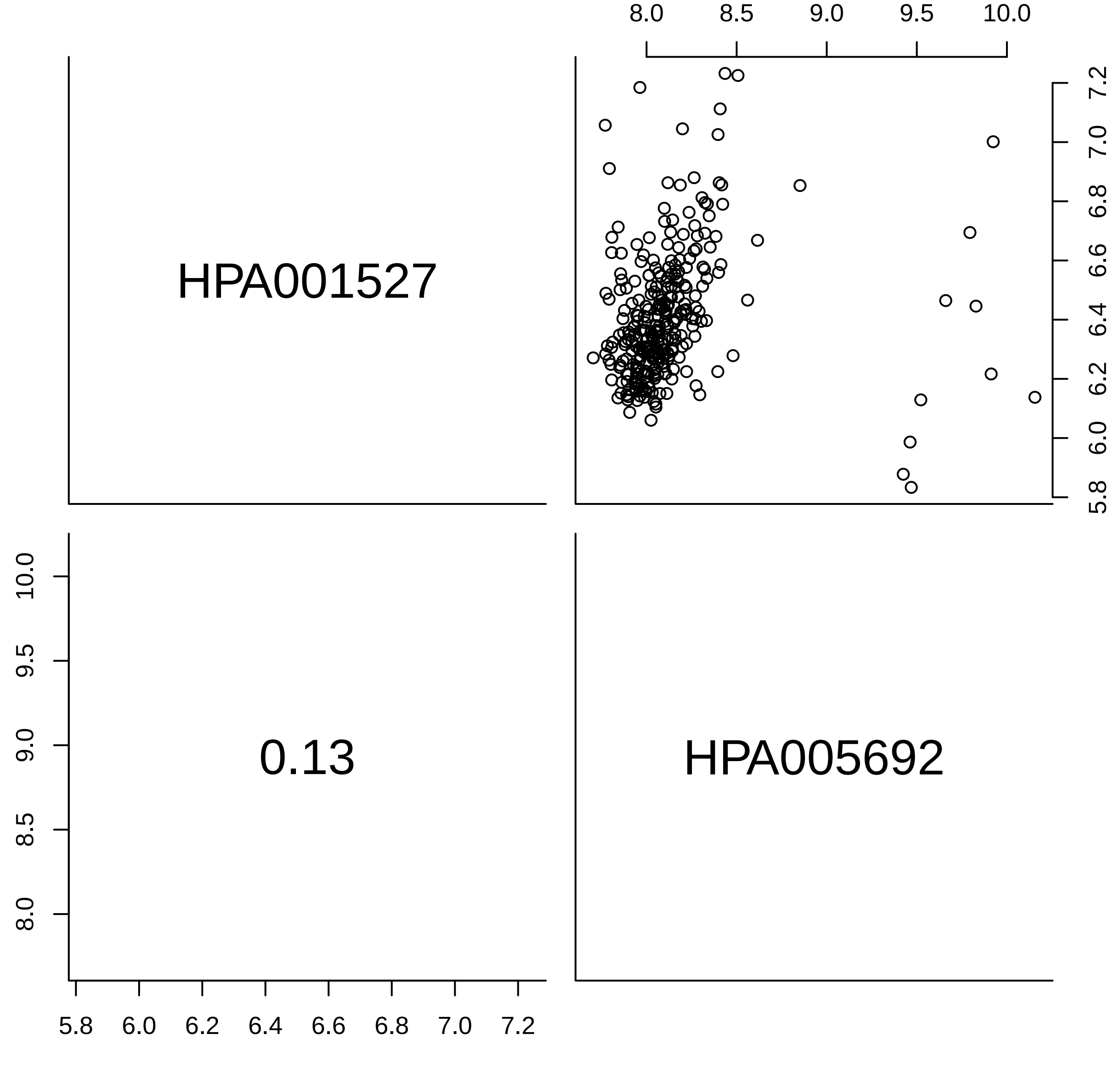

8.0
8.5
9.0
9.5
10.0
7.2
7.0
6.8
6.6
6.4
6.2
6.0
5.8
HPA001527
0.13
HPA005692
10.0
9.5
9.0
8.5
8.0
5.8
6.0
6.2
6.4
6.6
6.8
7.0
7.2

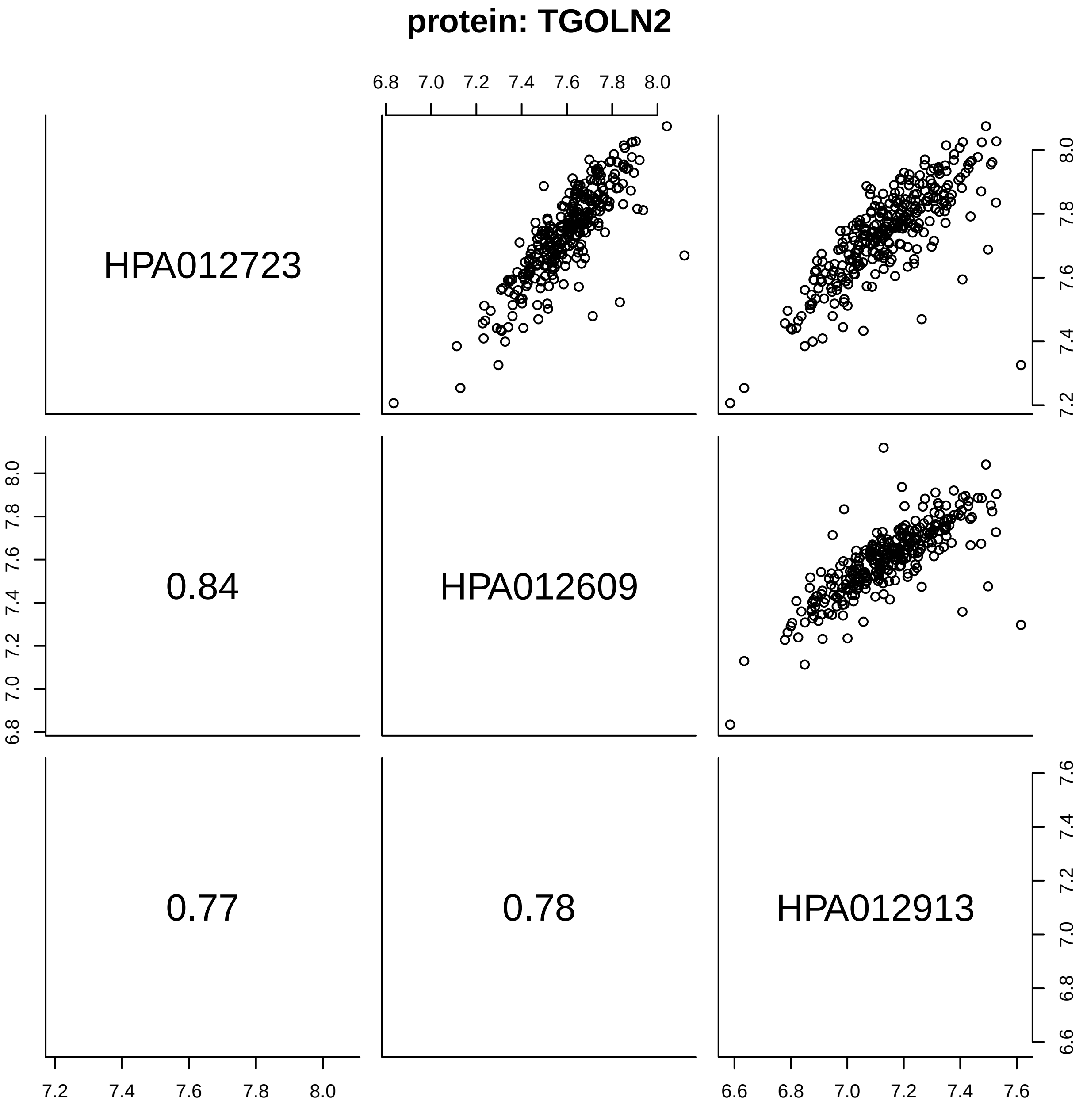
protein: TGOLN2
HPA012723
HPA012609
HPA012913
0.84
0.77
0.78
6.8 7.0 7.2 7.4 7.6 7.8 8.0
7.2 7.4 7.6 7.8 8.0
6.6 6.8 7.0 7.2 7.4 7.6

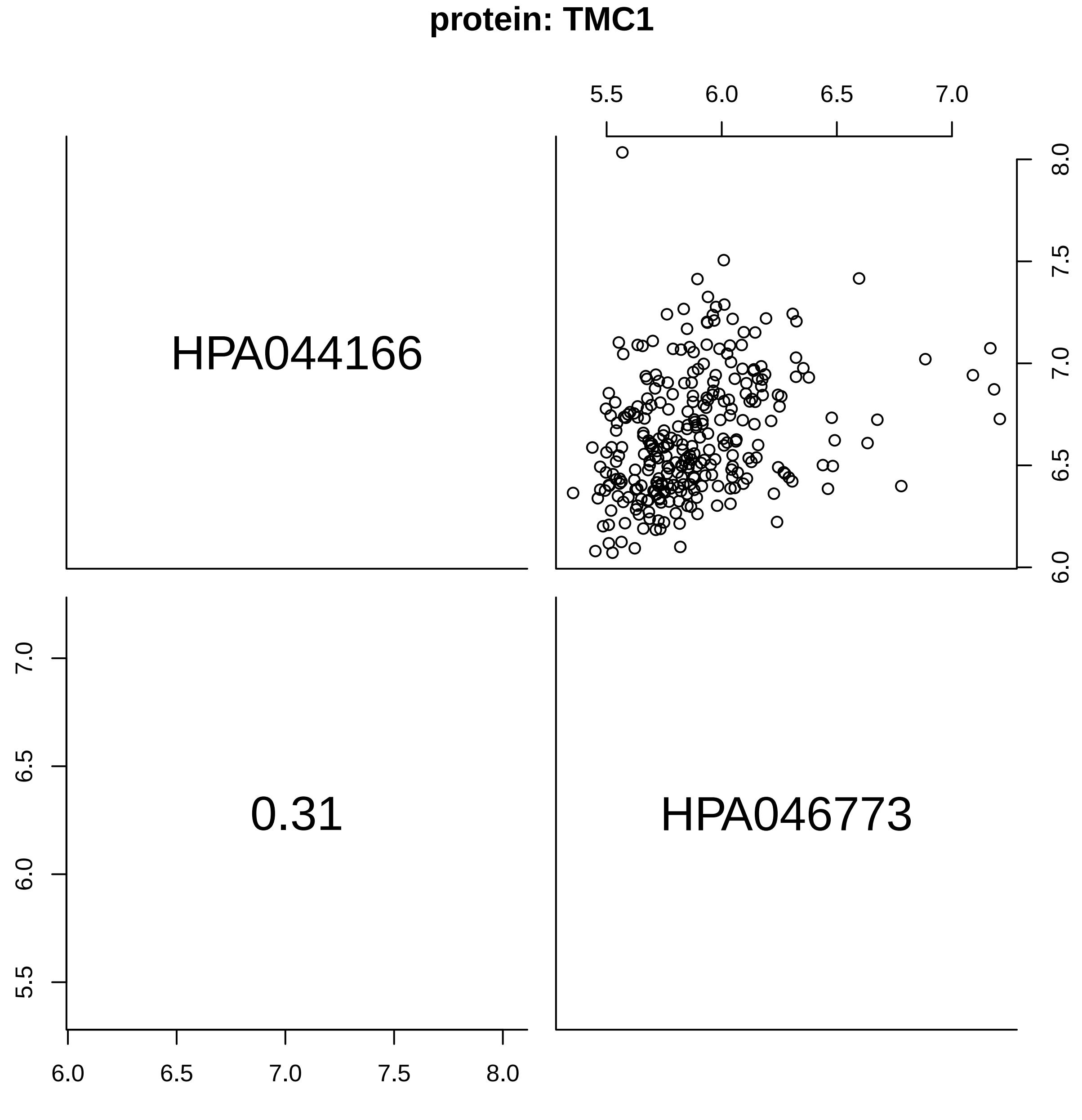

protein: TMC1
HPA044166
HPA046773
0.31
5.5
6.0
6.5
7.0
7.5
8.0

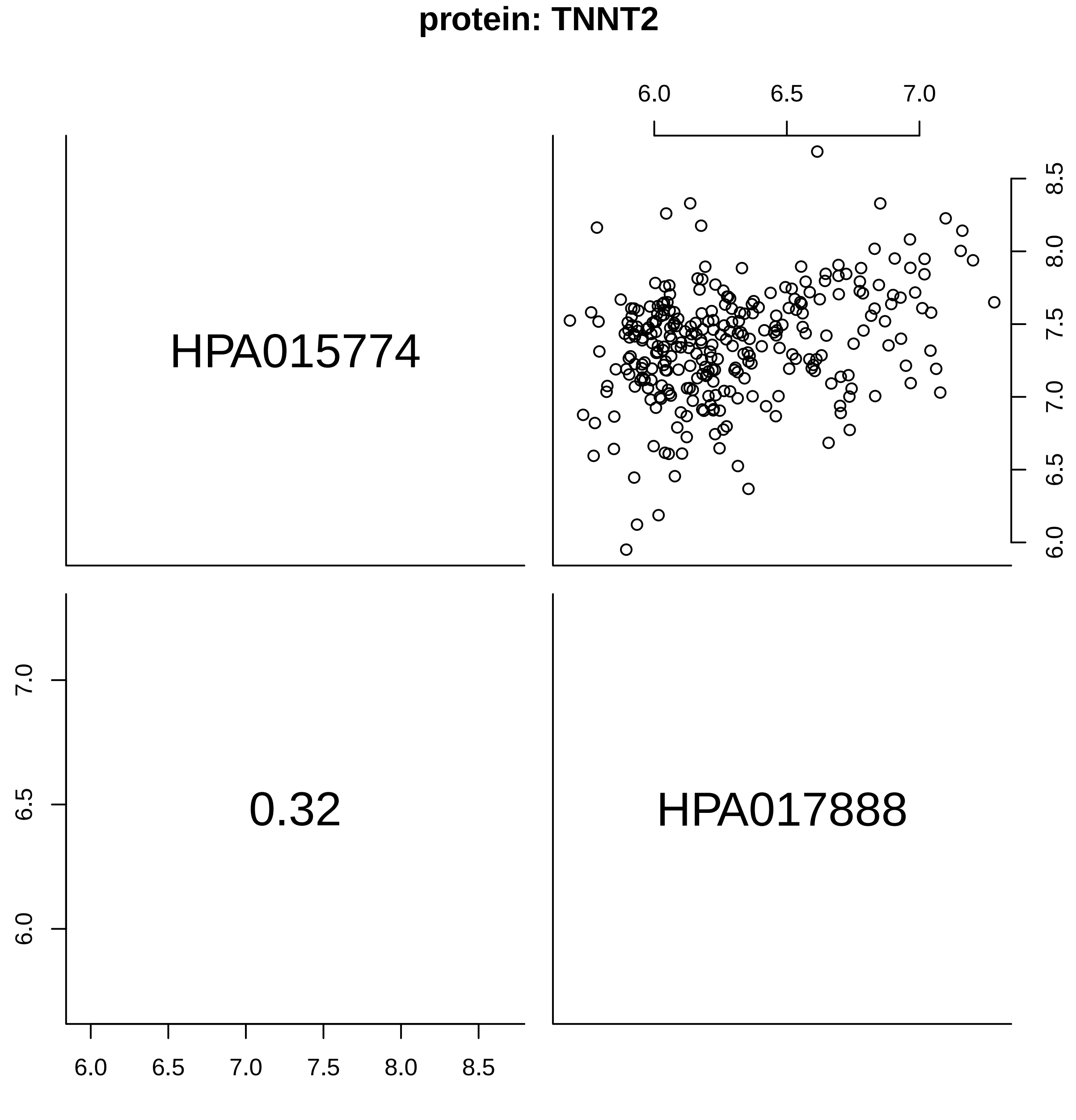
protein: TNNT2
HPA015774
HPA017888
0.32
6.0
6.5
7.0
7.5
8.0
8.5

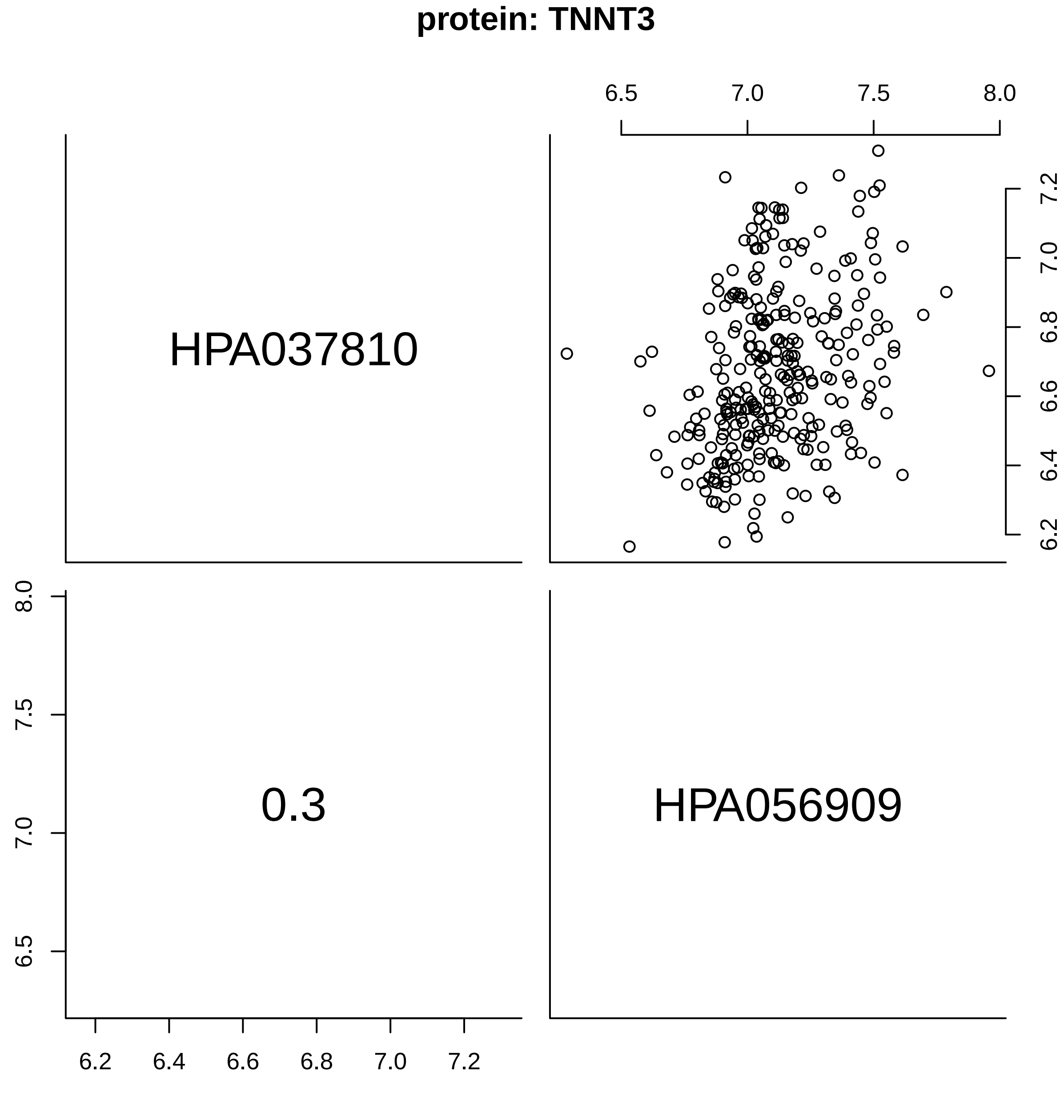
protein: TNNT3
HPA037810
0.3
HPA056909
6.5
7.0
7.5
8.0
6.2
6.4
6.6
6.8
7.0
7.2

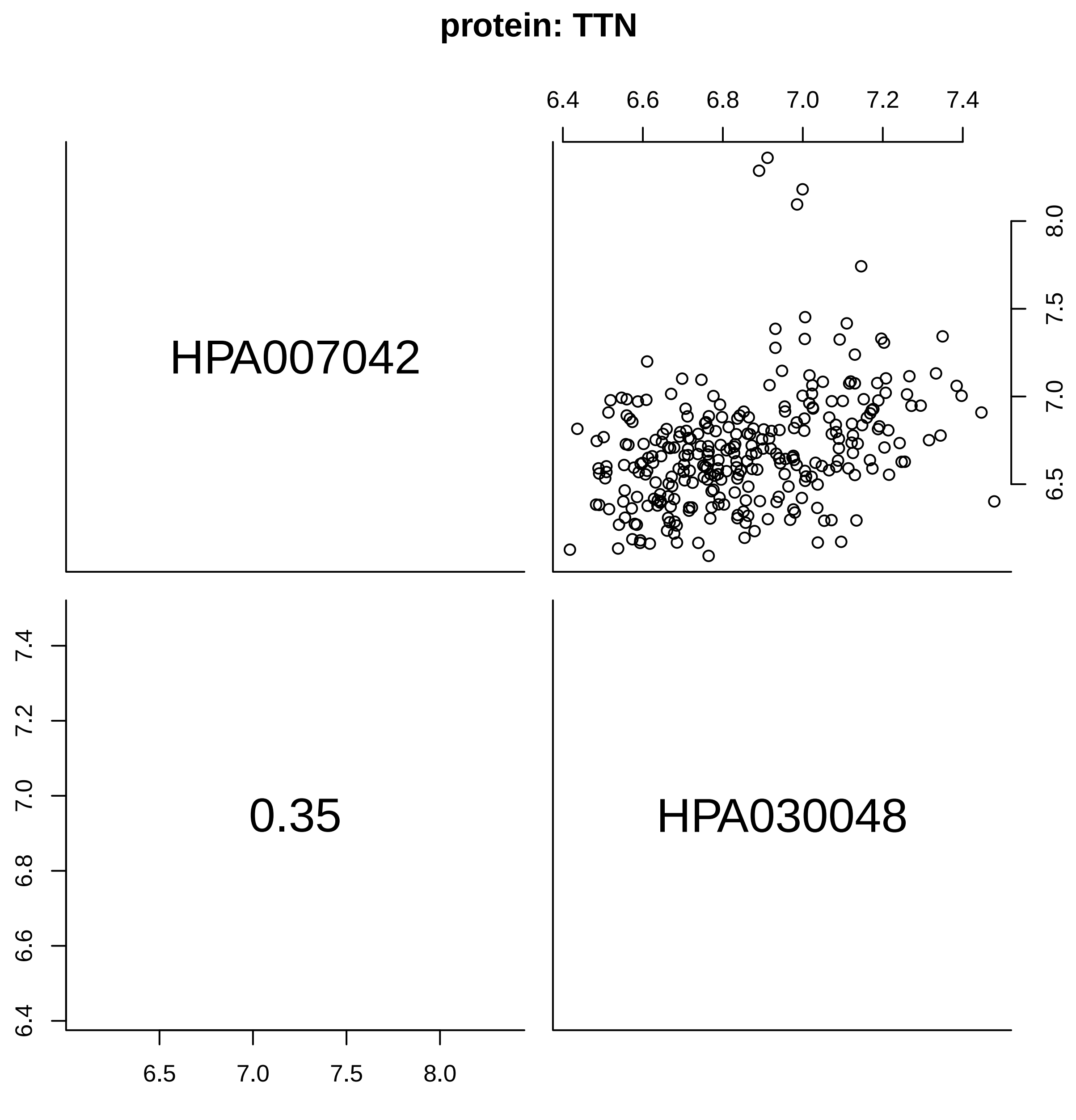

protein: TTN
HPA007042
HPA030048
0.35
6.4
6.6
6.8
7.0
7.2
7.4
6.5
7.0
7.5
8.0

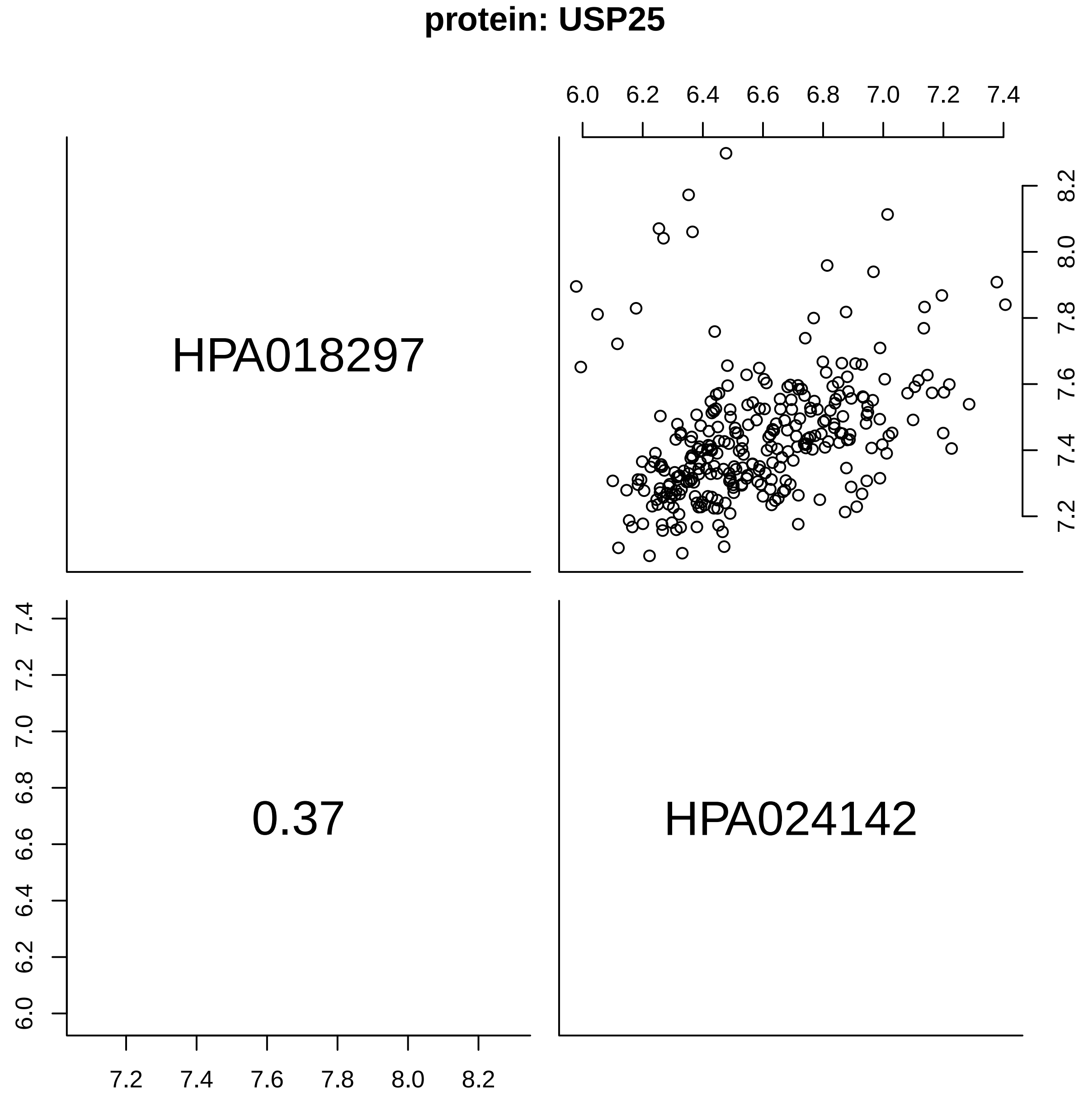

protein: USP25
HPA018297
HPA024142
0.37
6.0
6.2
6.4
6.6
6.8
7.0
7.2
7.4
8.2
8.0
7.8
7.6
7.4
7.2
7.4
7.2
7.0
6.8
6.6
6.4
6.2
6.0
7.2
7.4
7.6
7.8
8.0
8.2

## protein: XRCC4

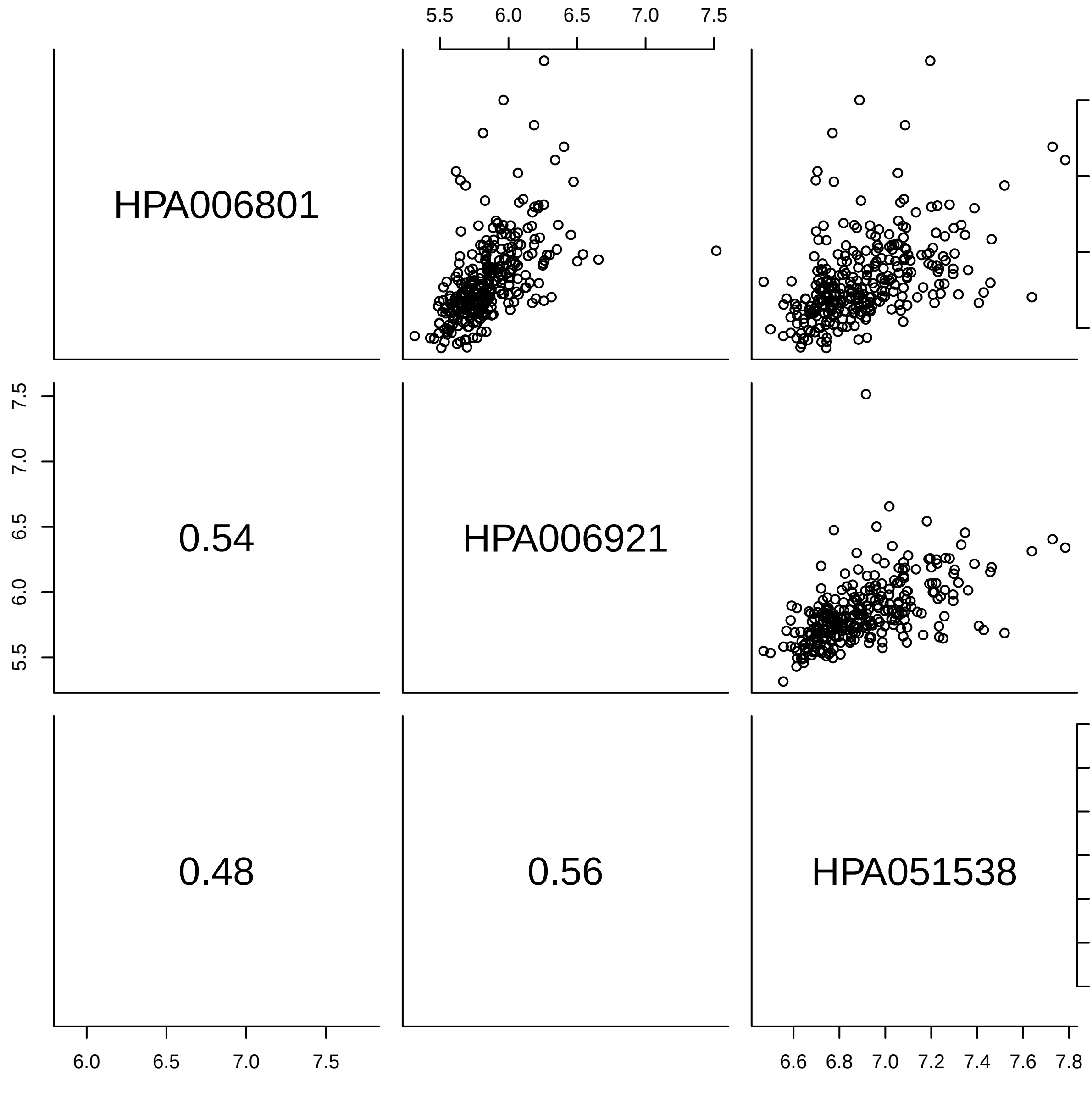

Supplement: Supplementary file 5 — File S1 Comparison of fluorescence intensity for proteins measured through different antibodies. [file JCSM-11-505-s001.pdf]
