# Supplementary material for: Longitudinal serum biomarker screening identifies malate dehydrogenase 2 as candidate prognostic biomarker for Duchenne muscular dystrophy
Source: J Cachexia Sarcopenia Muscle. 2019 Dec 27;11(2):505–17. doi: 10.1002/jcsm.12517 (PMC7113516; doi:10.1002/jcsm.12517)
Supplement: Supplementary file 6 — File S2 Trajectory plots for all proteins significantly associated with age. [file JCSM-11-505-s002.pdf]

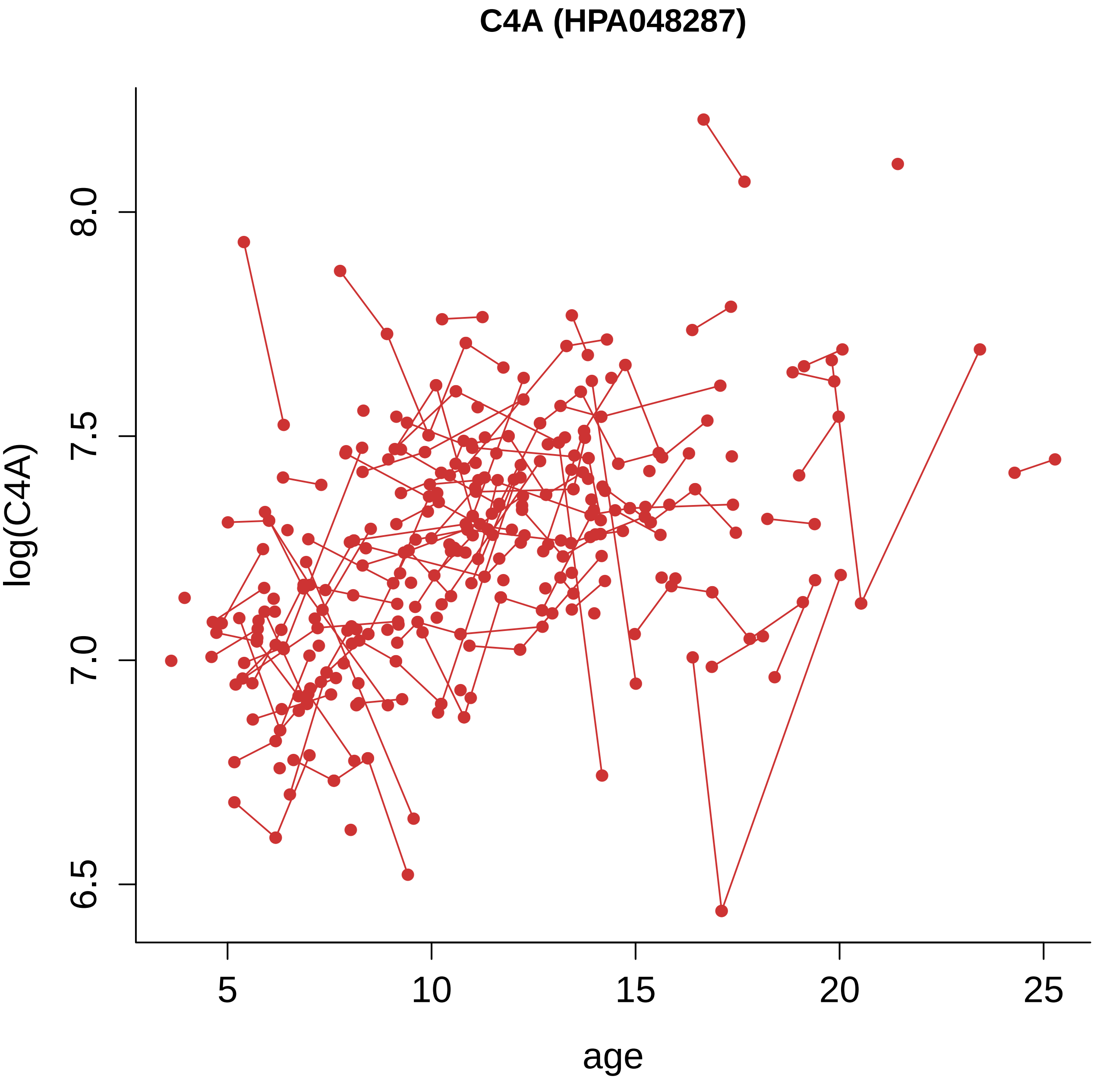
C4A (HPA048287)
log(C4A)
8.0
7.5
7.0
6.5
5
10
15
20
25
age

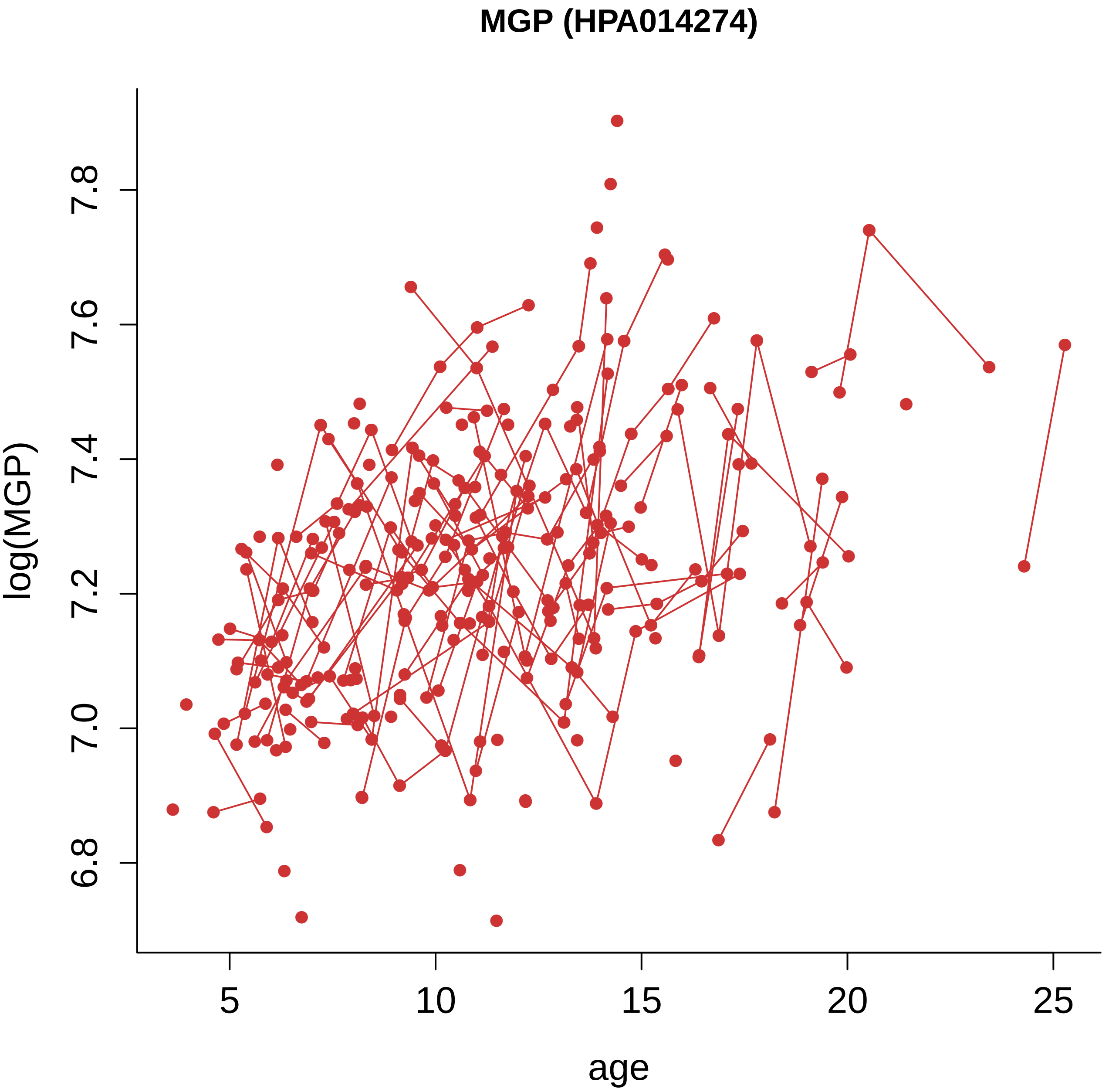
MGP (HPA014274)
log(MGP)
6.8
7.0
7.2
7.4
7.6
7.8
5
10
15
20
25
age

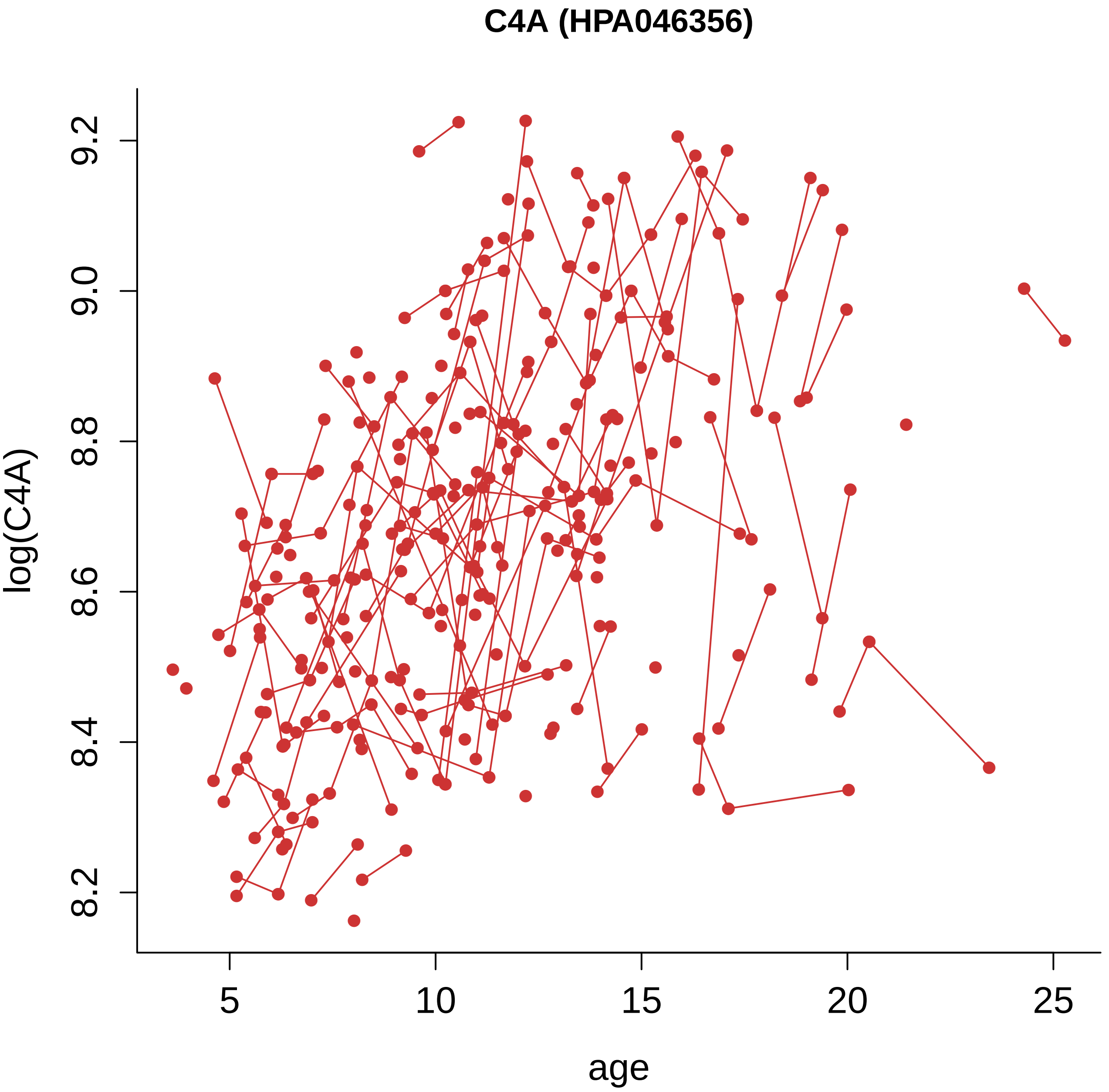

C4A (HPA046356)
log(C4A)
9.2
9.0
8.8
8.6
8.4
8.2
5
10
15
20
25
age

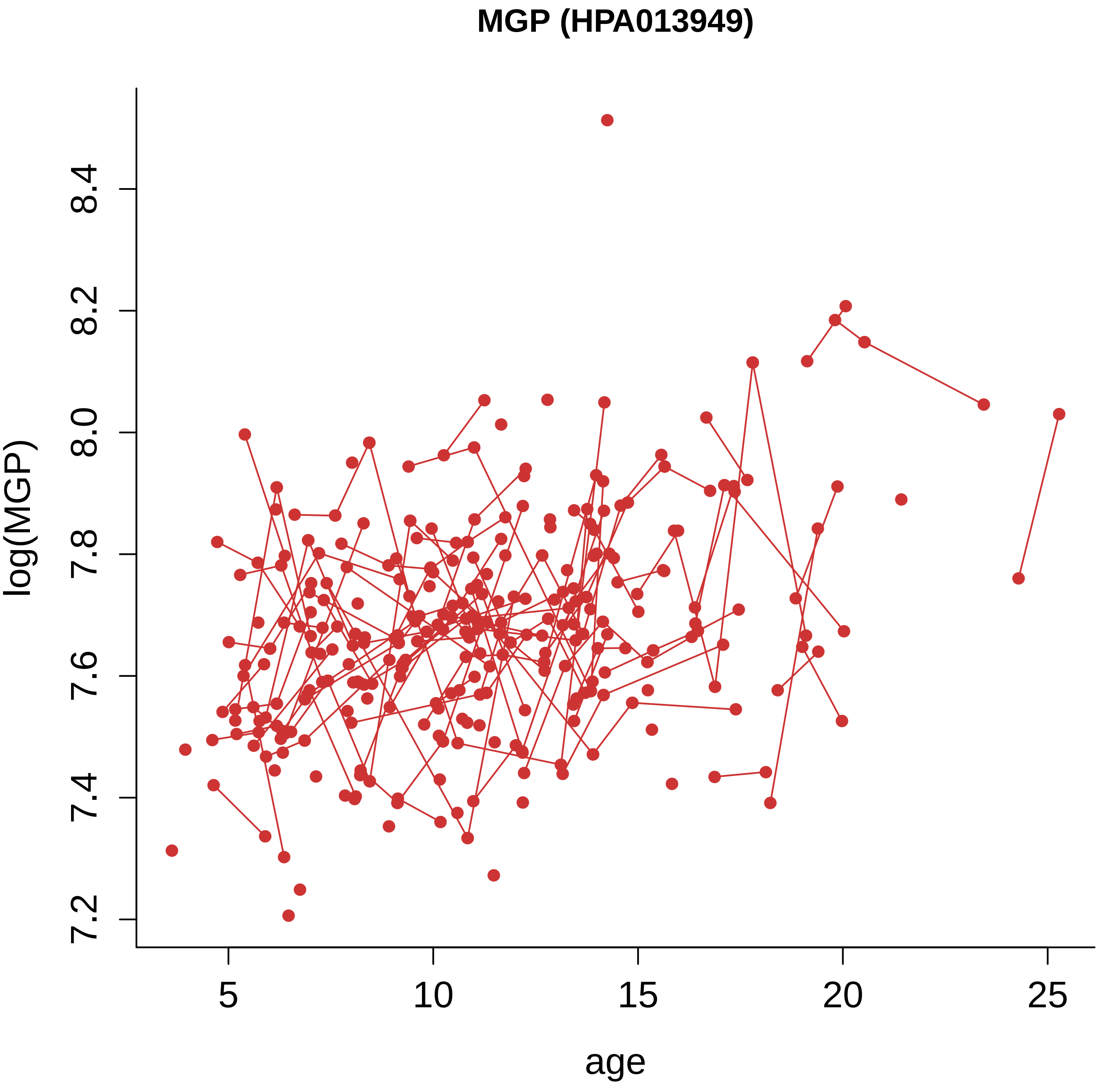

MGP (HPA013949)
log(MGP)
7.2
7.4
7.6
7.8
8.0
8.2
8.4
age
5
10
15
20
25

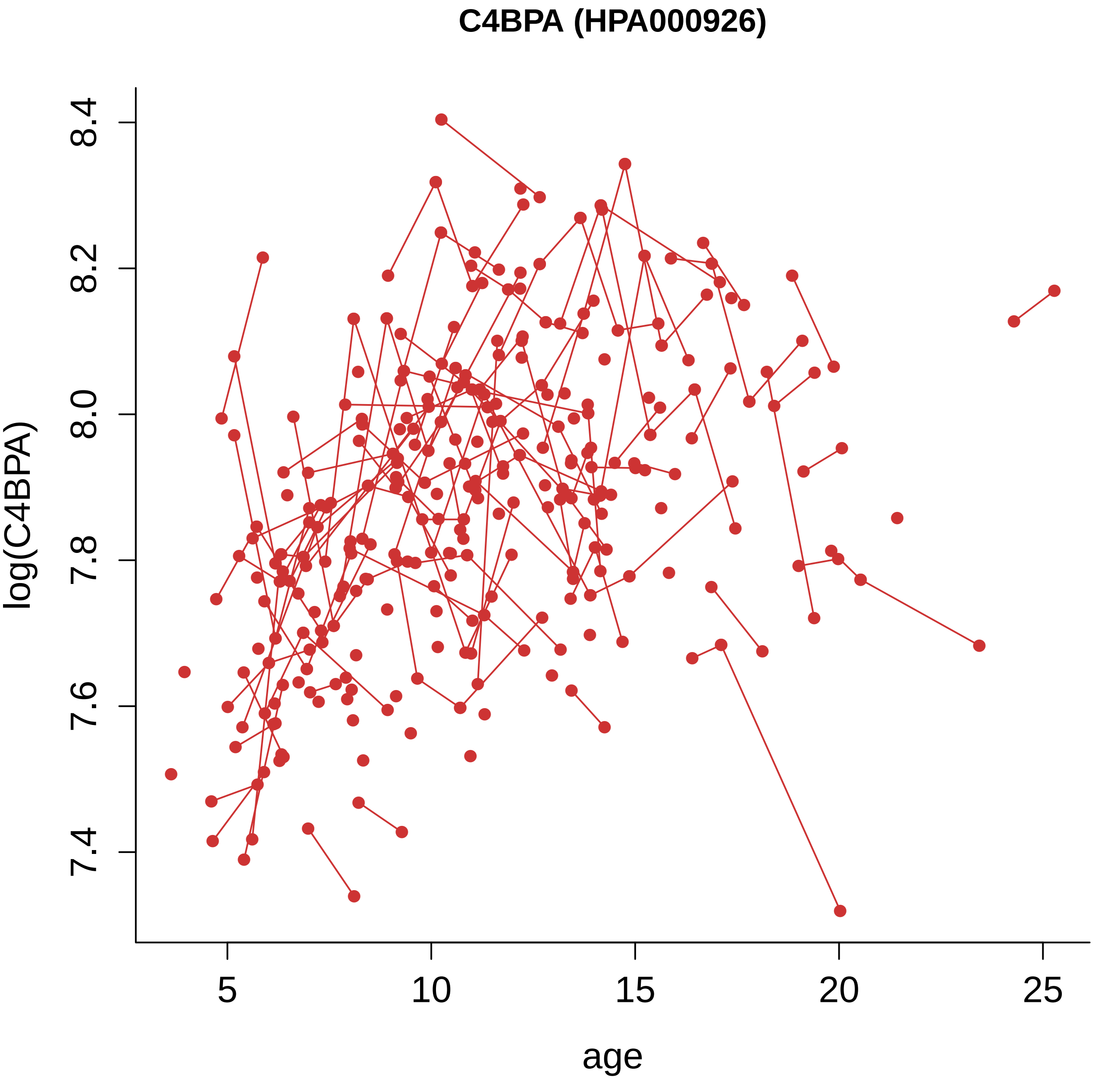
C4BPA (HPA000926)
log(C4BPA)
8.4
8.2
8.0
7.8
7.6
7.4
5
10
15
20
25
age

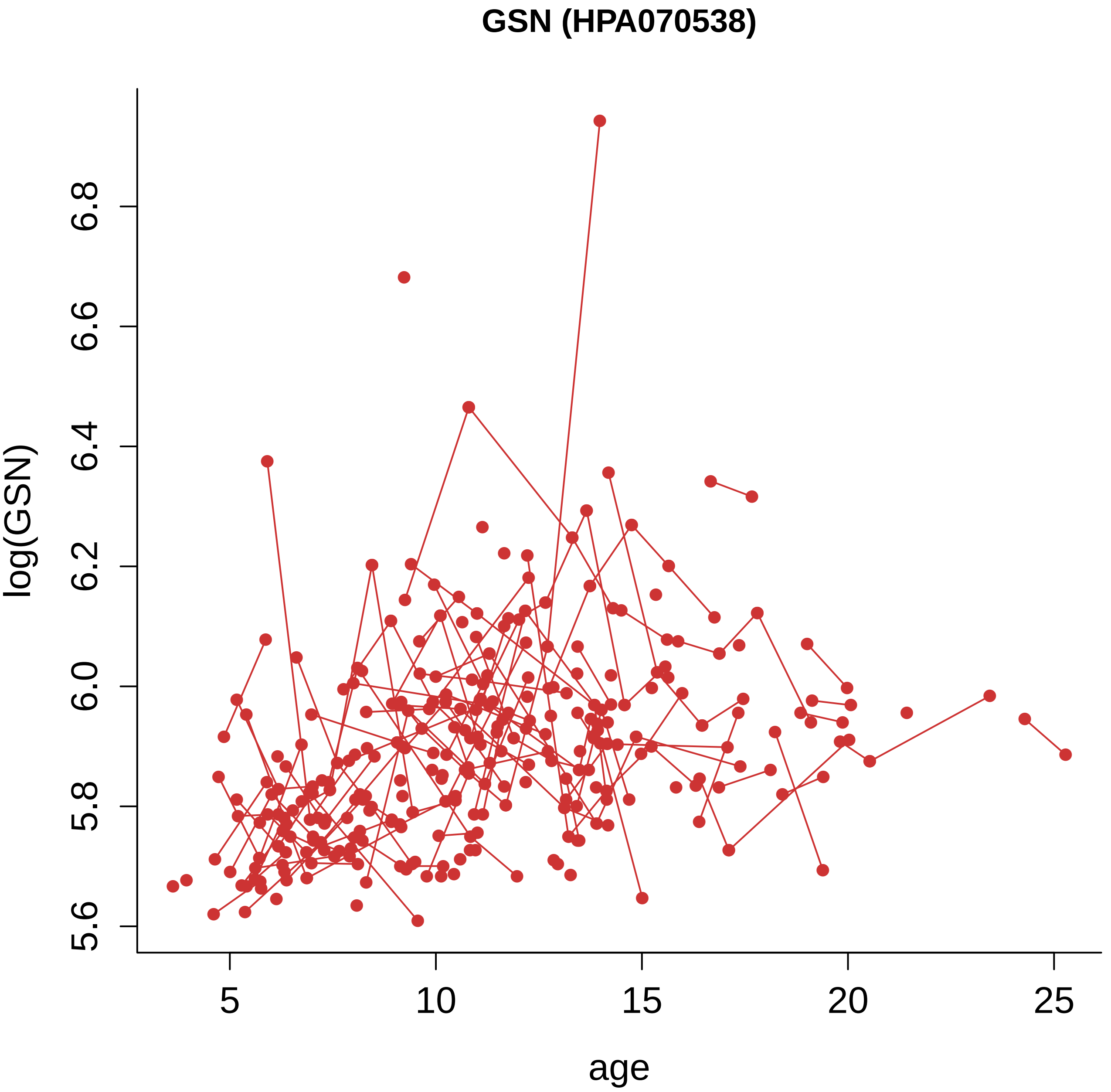

GSN (HPA070538)
log(GSN)
6.8
6.6
6.4
6.2
6.0
5.8
5.6
5
10
15
20
25
age

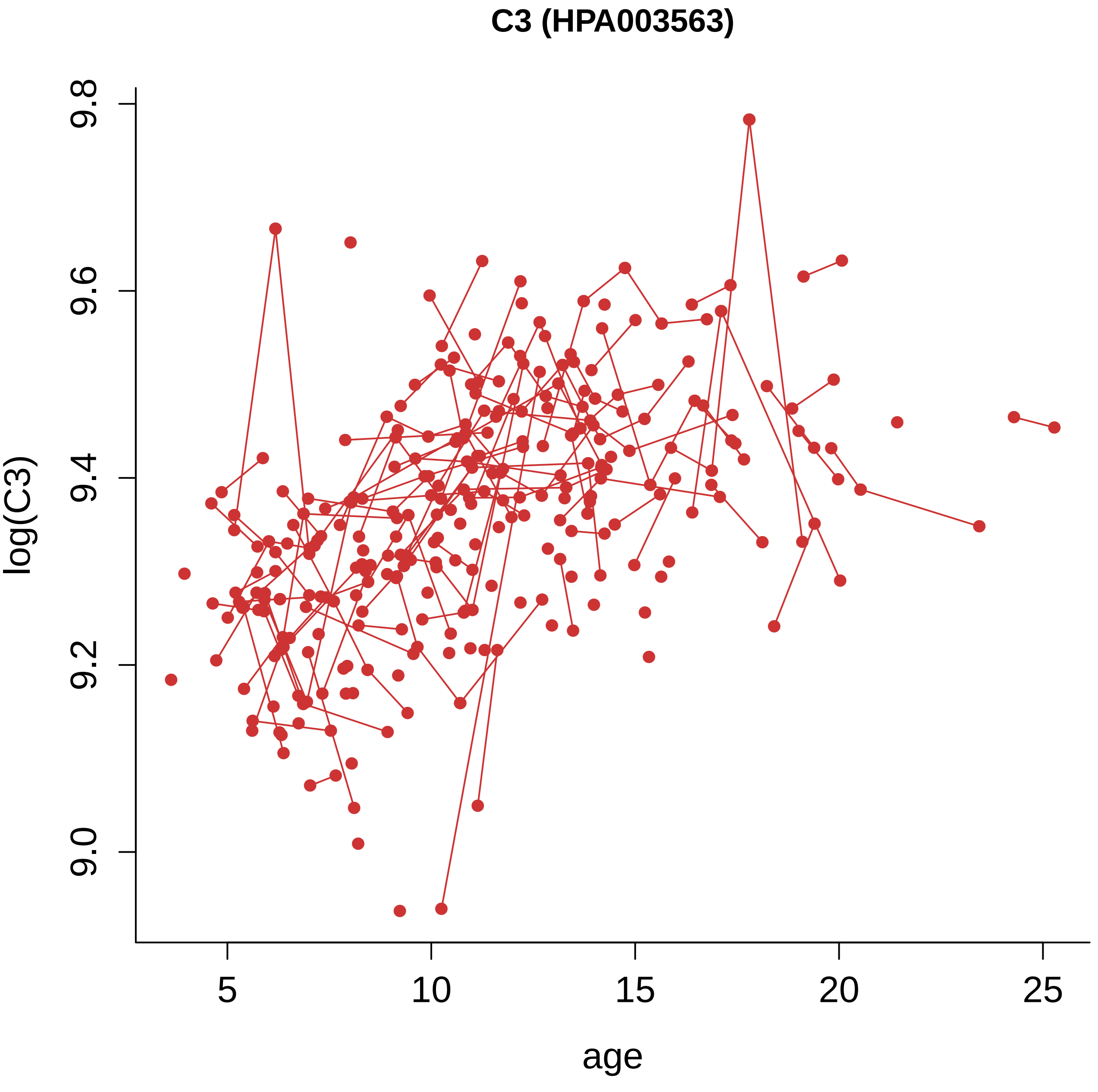
C3 (HPA003563)
log(C3)
9.0
9.2
9.4
9.6
9.8
age
5
10
15
20
25

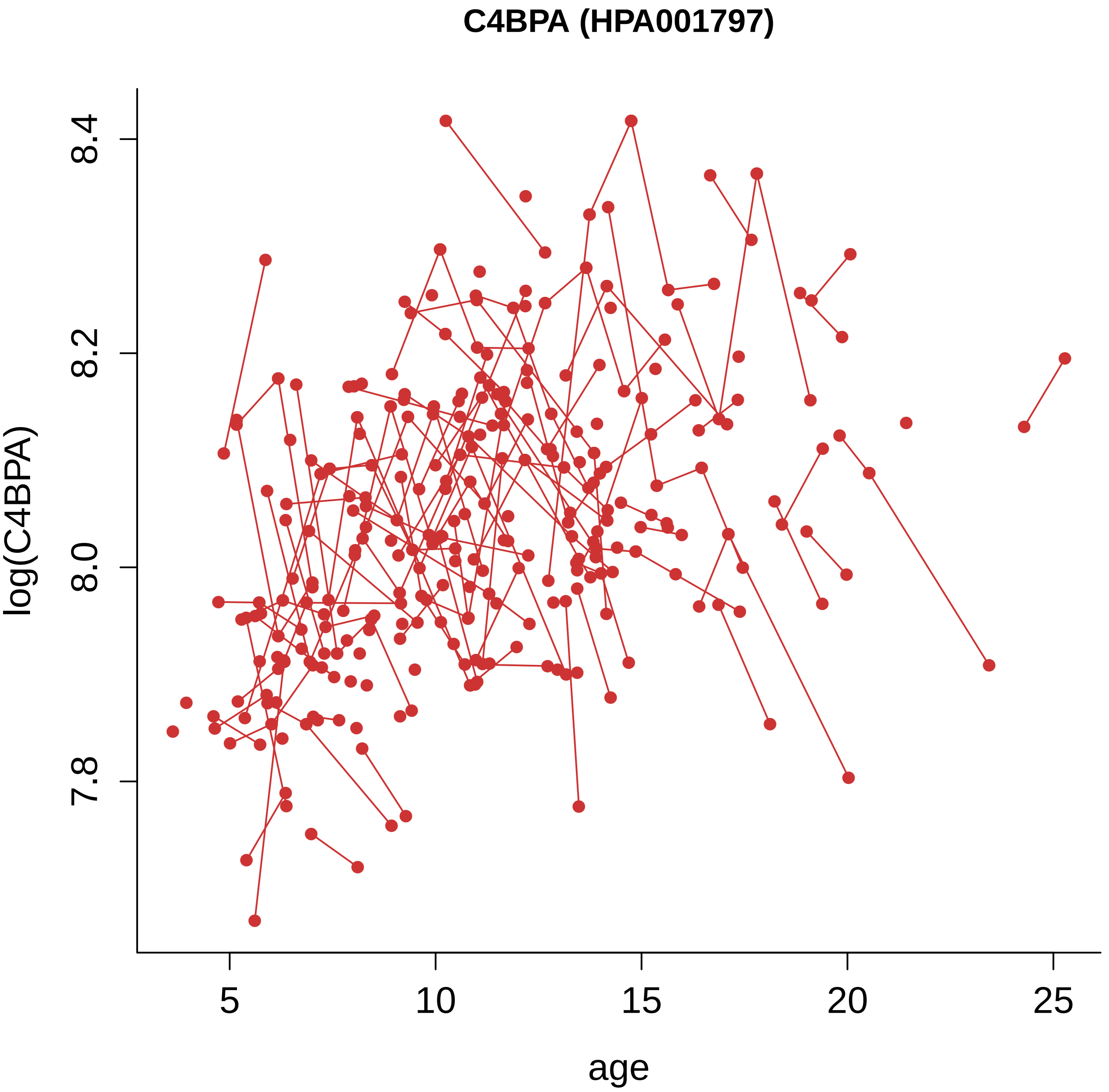
C4BPA (HPA001797)
log(C4BPA)
8.4
8.2
8.0
7.8
5
10
15
20
25
age

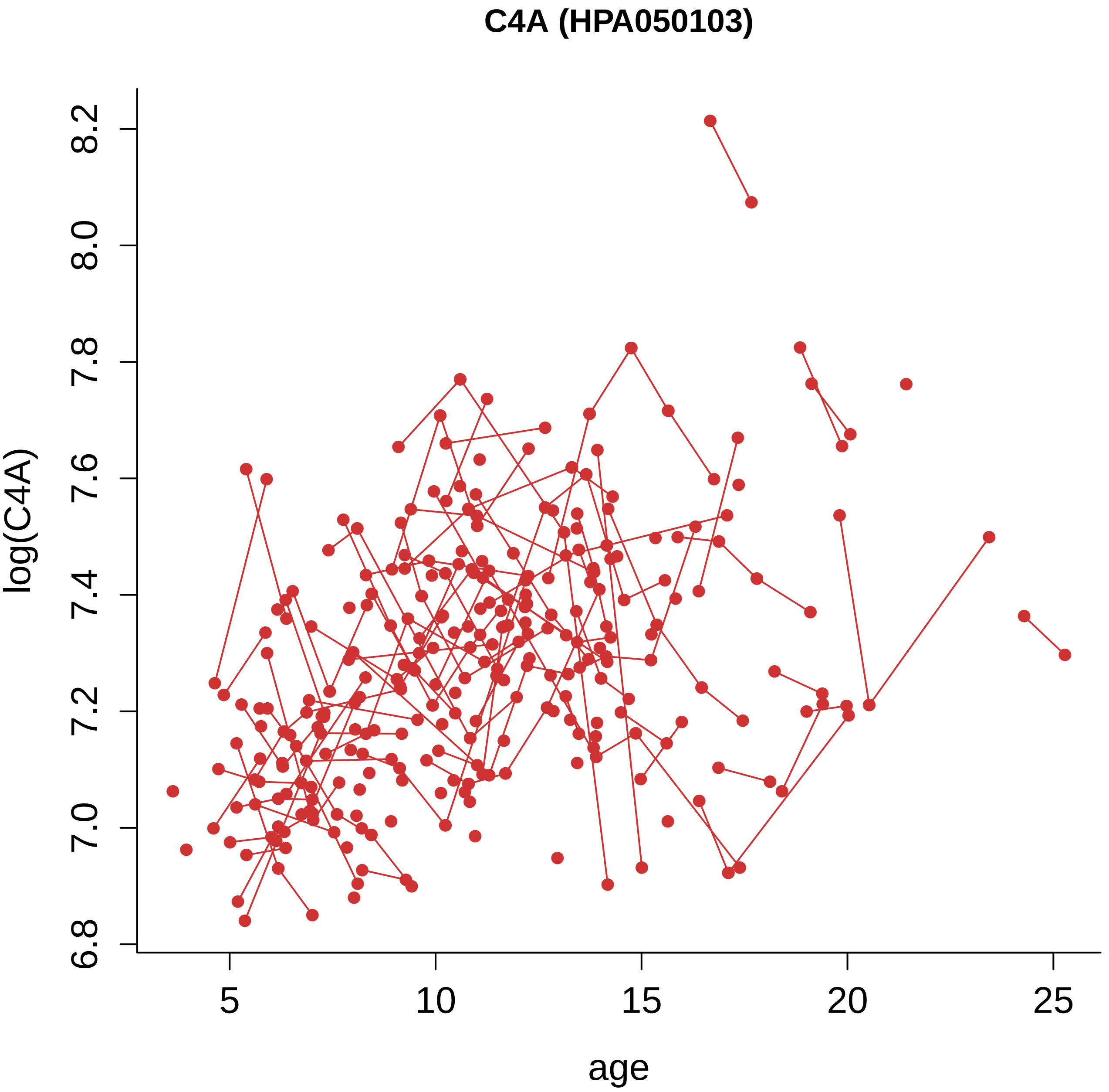
C4A (HPA050103)
log(C4A)
6.8
7.0
7.2
7.4
7.6
7.8
8.0
8.2
5
10
15
20
25
age

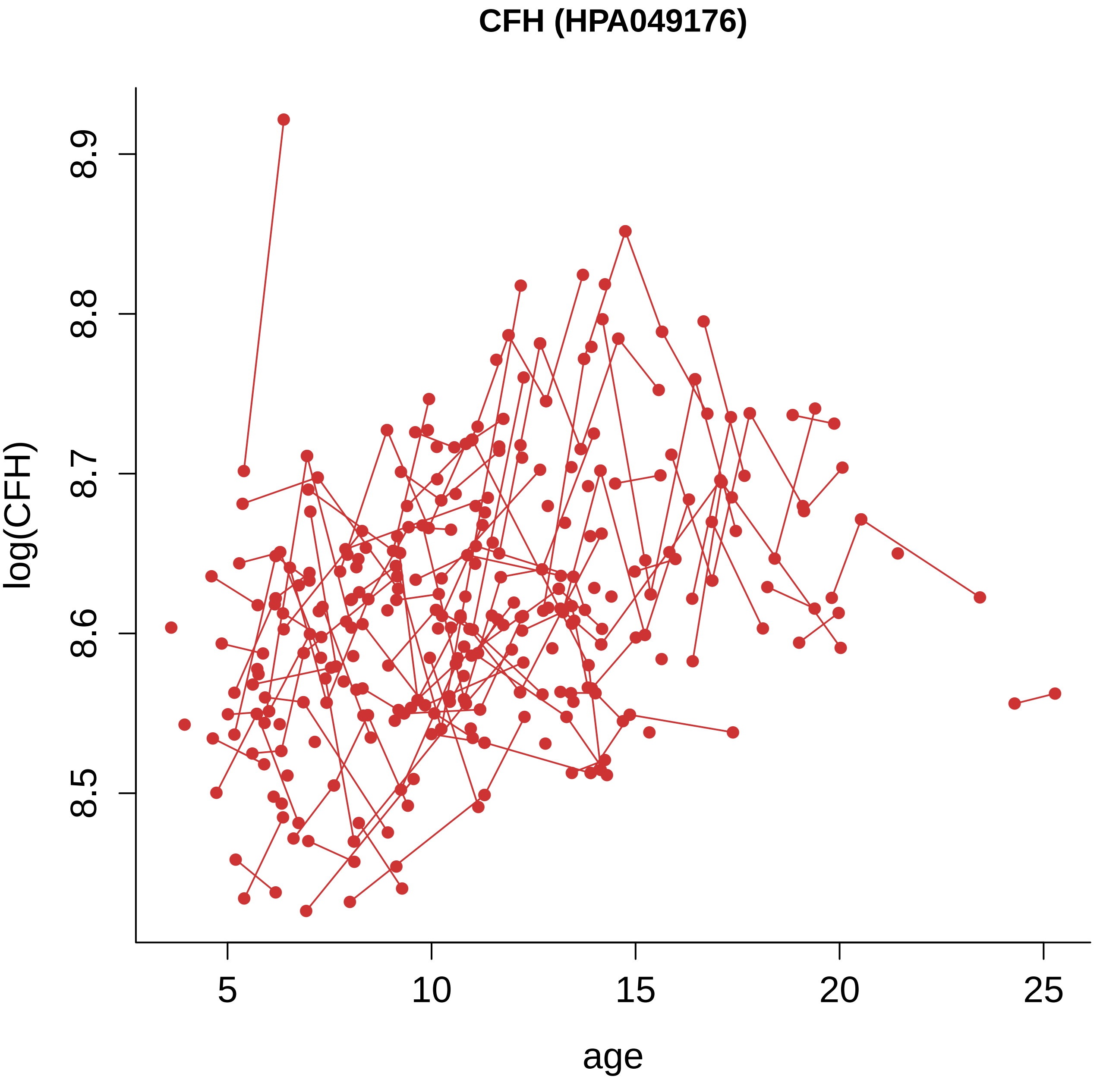
CFH (HPA049176)
log(CFH)
8.5
8.6
8.7
8.8
8.9
age
5
10
15
20
25

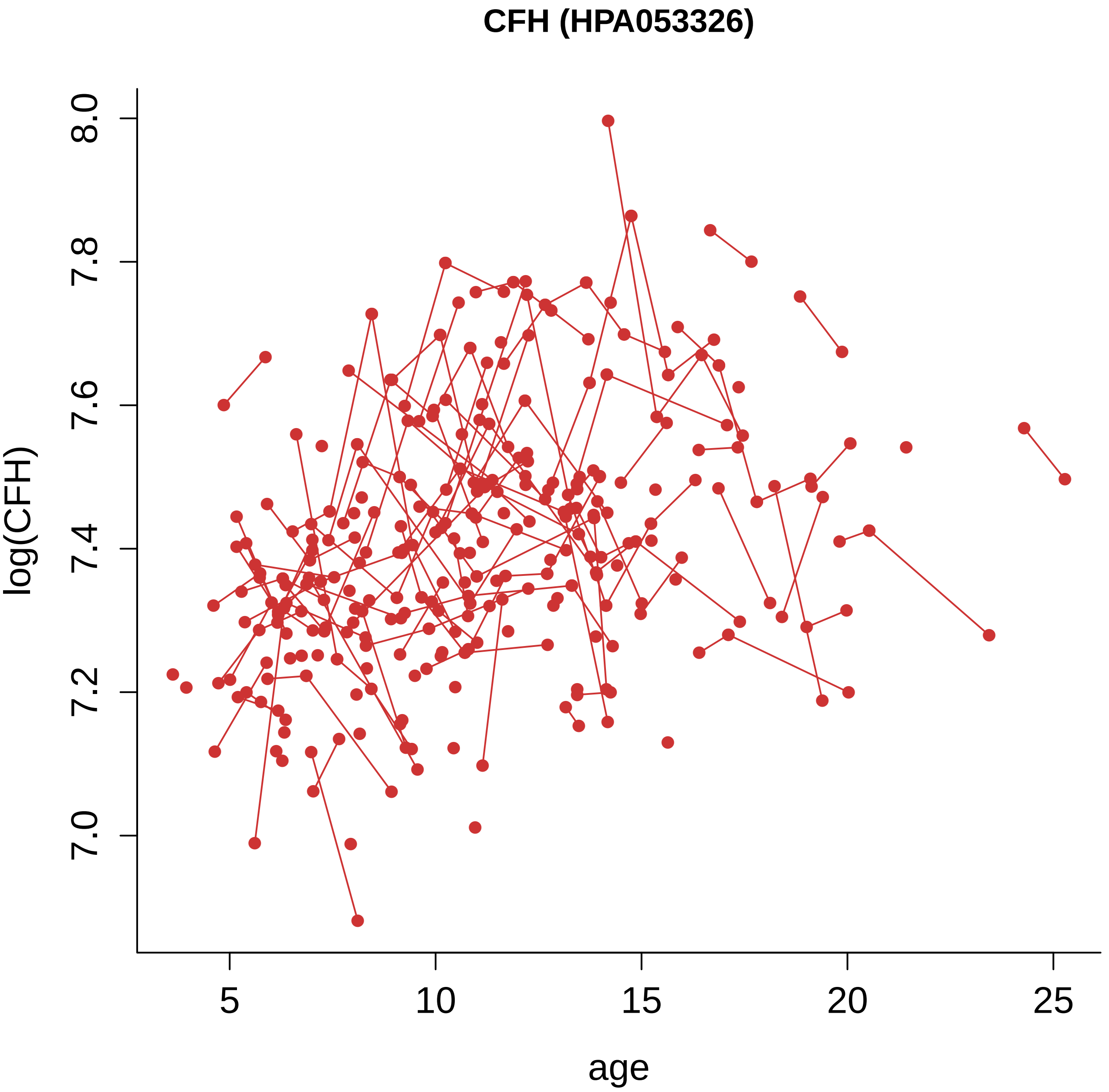

CFH (HPA053326)
log(CFH)
8.0
7.8
7.6
7.4
7.2
7.0
5
10
15
20
25
age

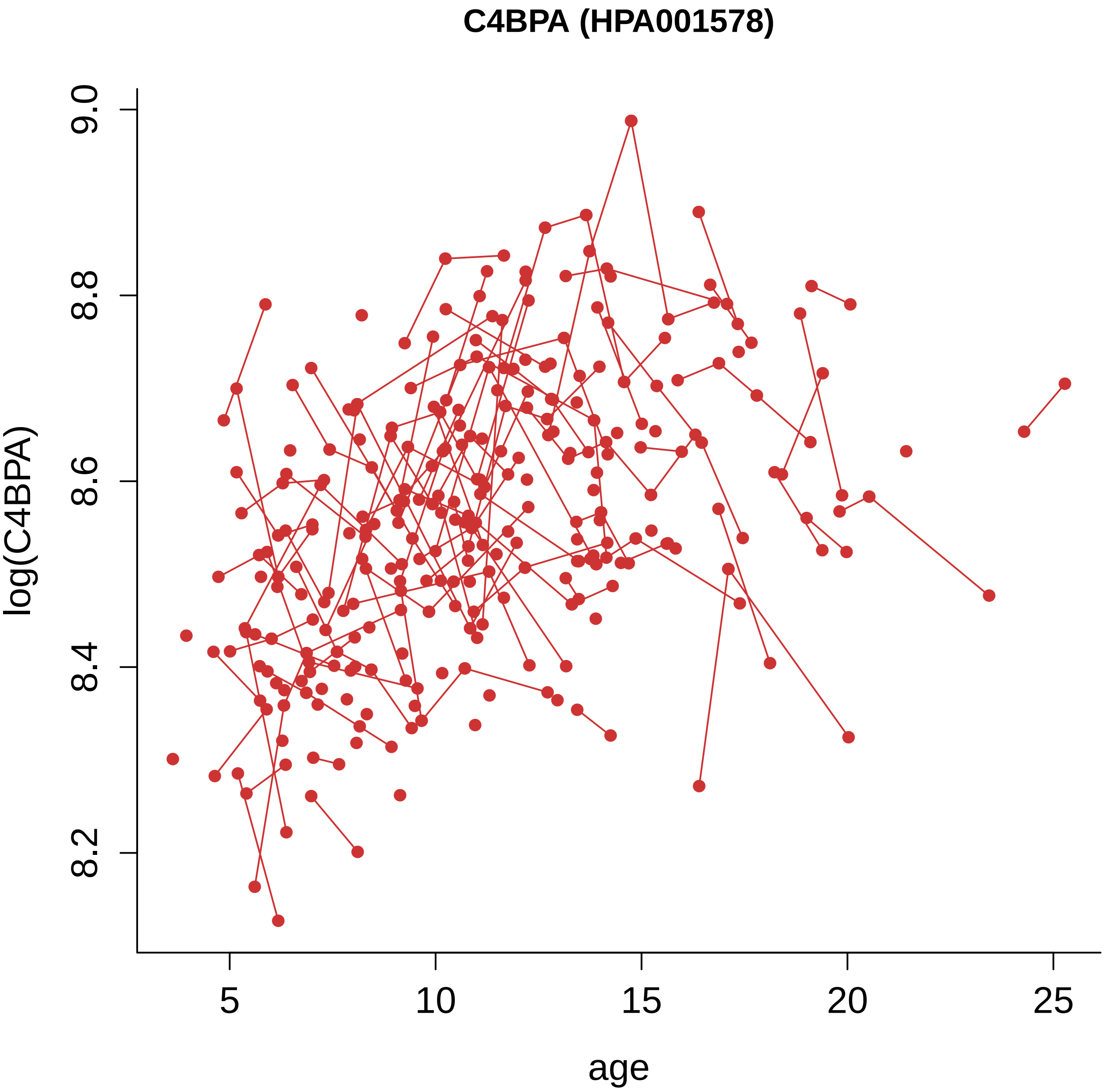

C4BPA (HPA001578)
log(C4BPA)
9.0
8.8
8.6
8.4
8.2
age
5
10
15
20
25

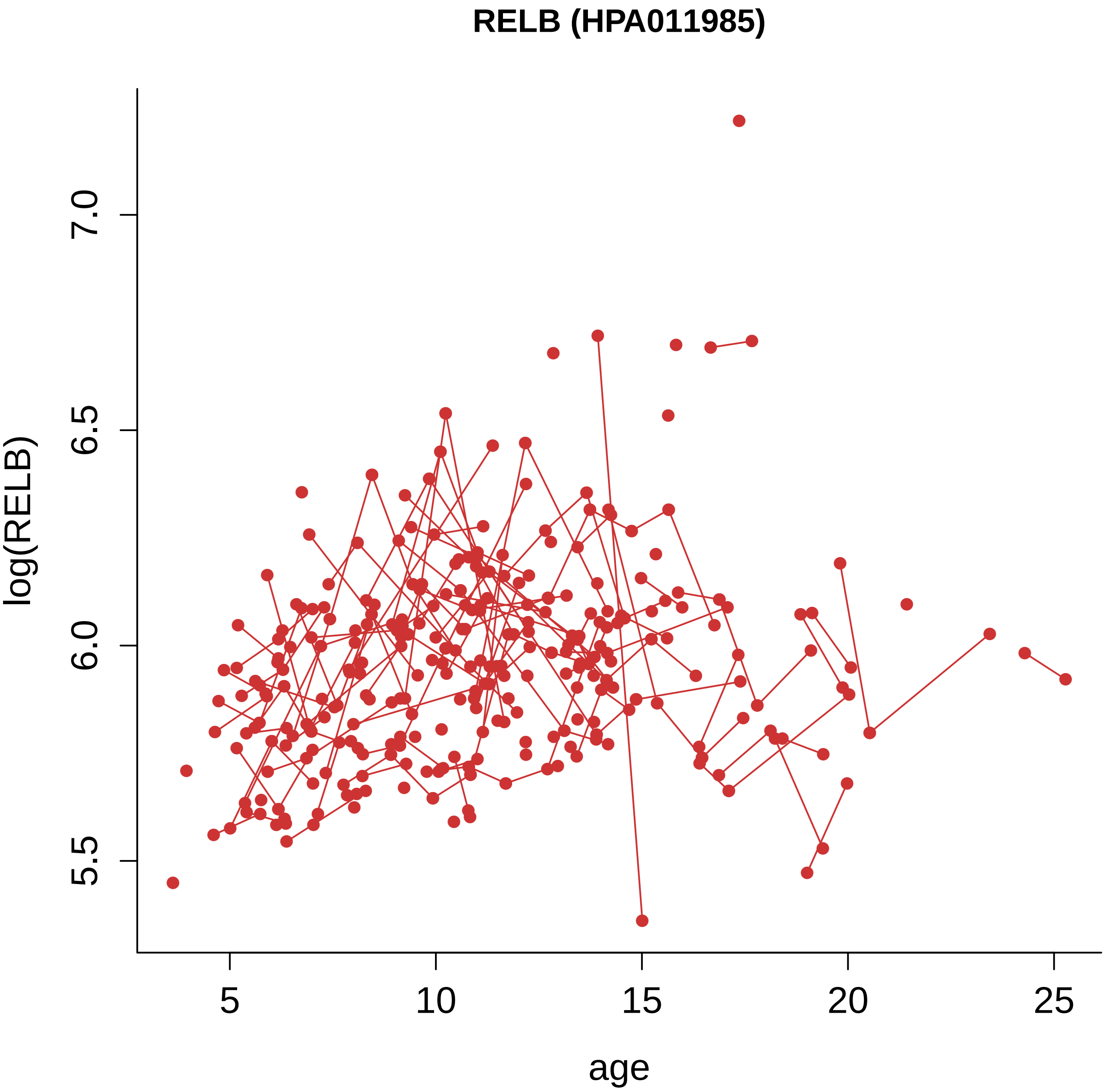
RELB (HPA011985)
log(RELB)
7.0
6.5
6.0
5.5
5
10
15
20
25
age

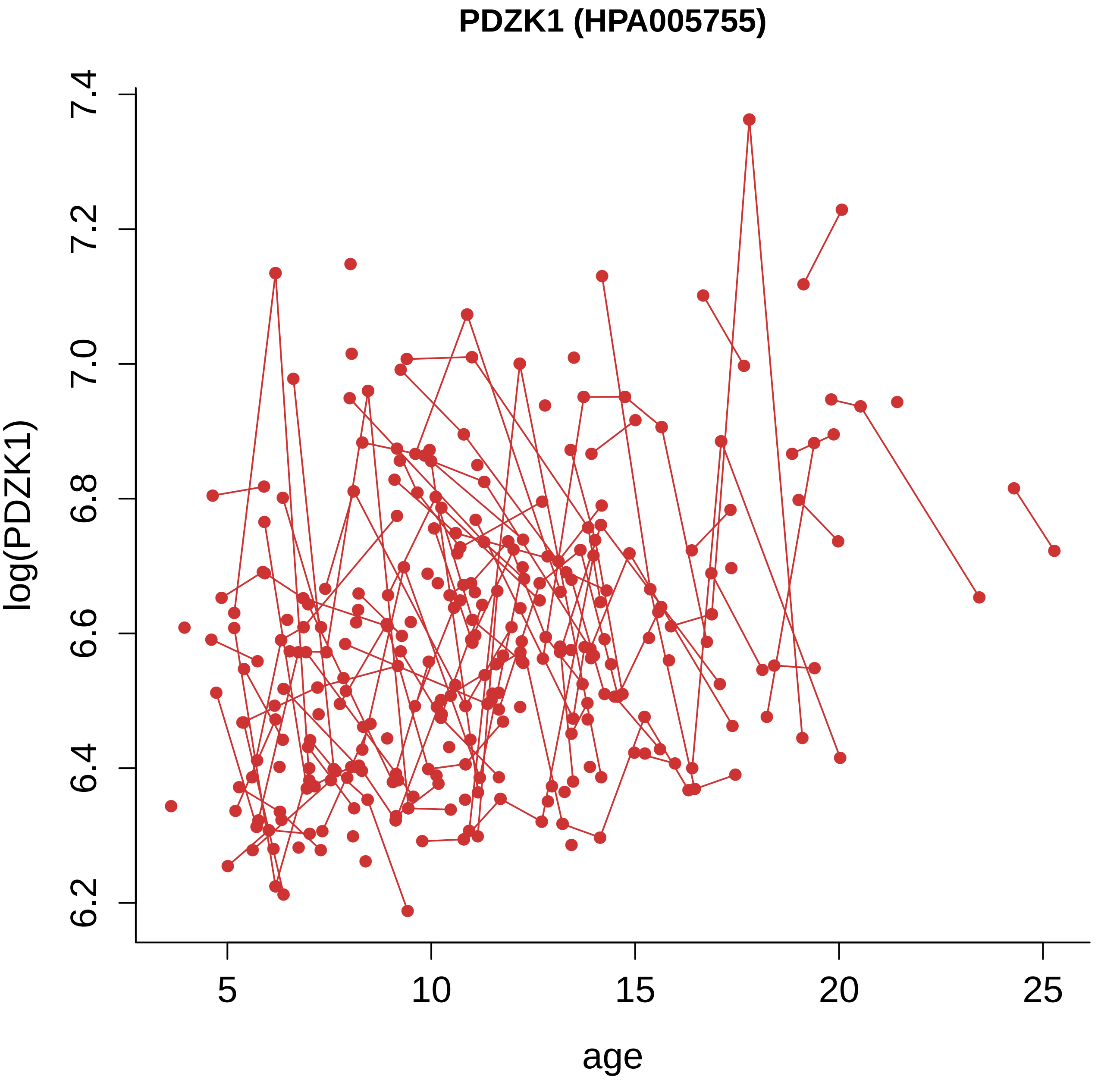

PDZK1 (HPA005755)
log(PDZK1)
age
7.4
7.2
7.0
6.8
6.6
6.4
6.2
5
10
15
20
25

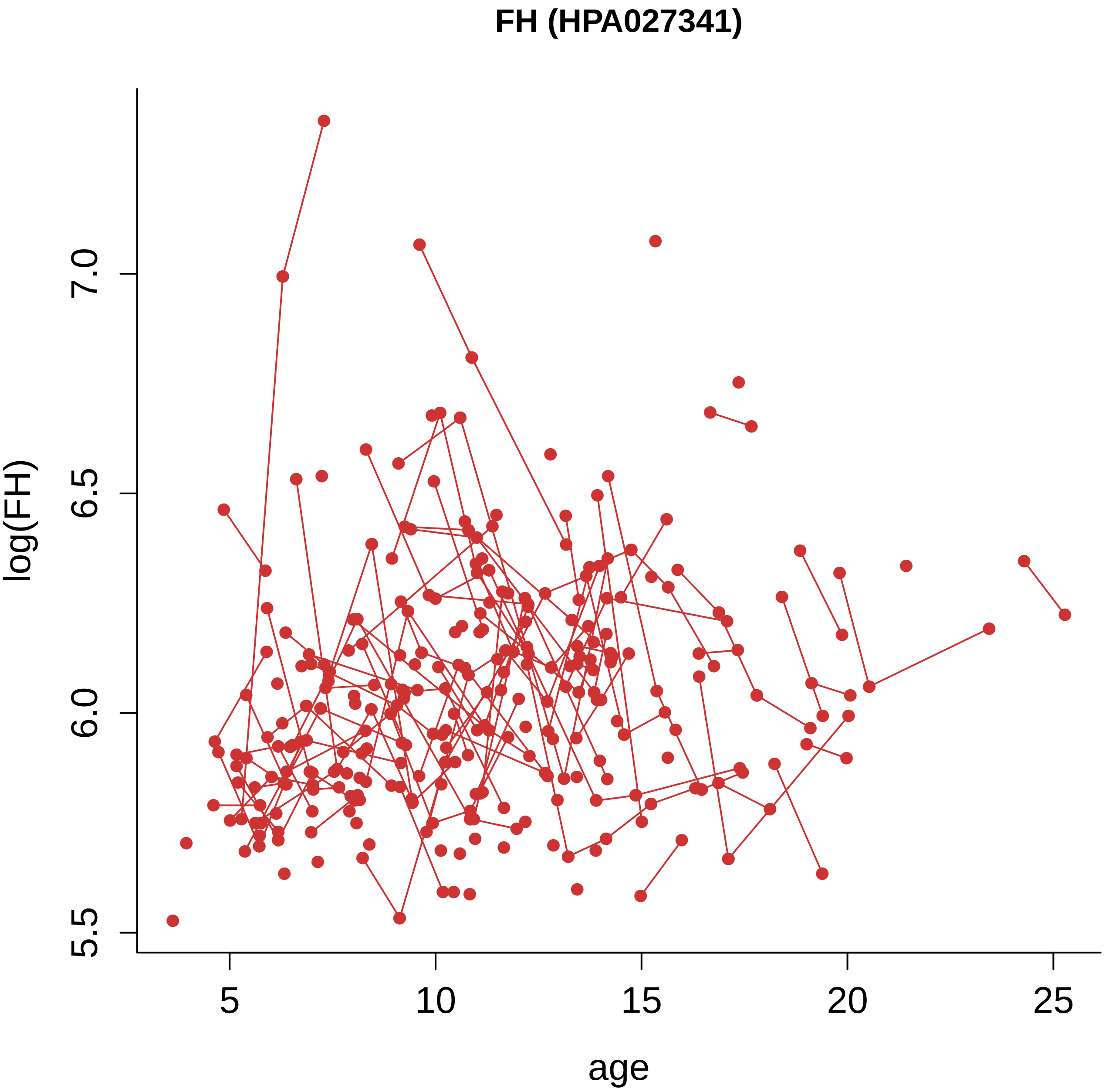
FH (HPA027341)
log(FH)
7.0
6.5
6.0
5.5
5
10
15
20
25
age

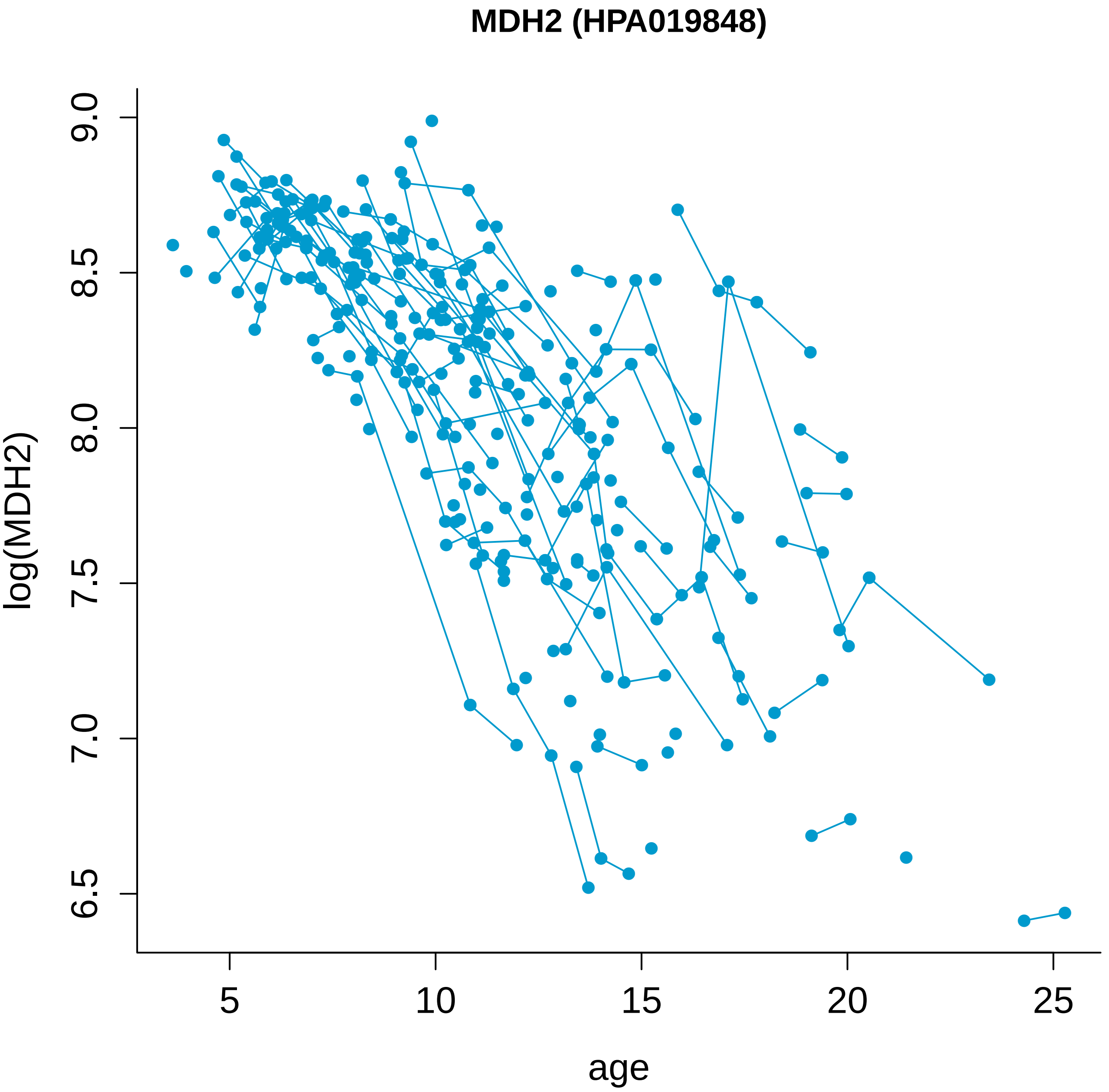
MDH2 (HPA019848)
log(MDH2)
9.0
8.5
8.0
7.5
7.0
6.5
5
10
15
20
25
age

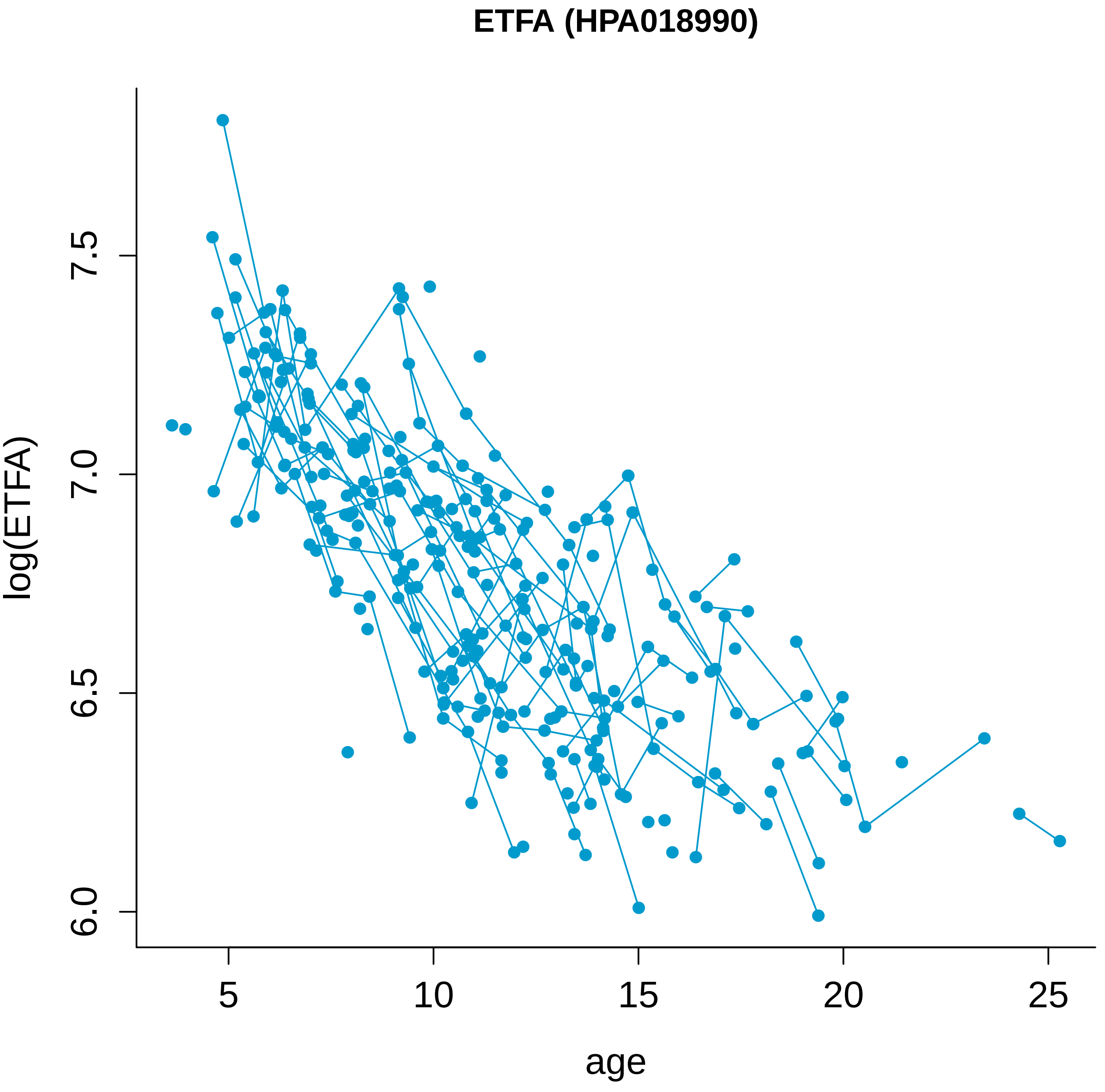
ETFA (HPA018990)
log(ETFA)
6.0
6.5
7.0
7.5
age
5
10
15
20
25

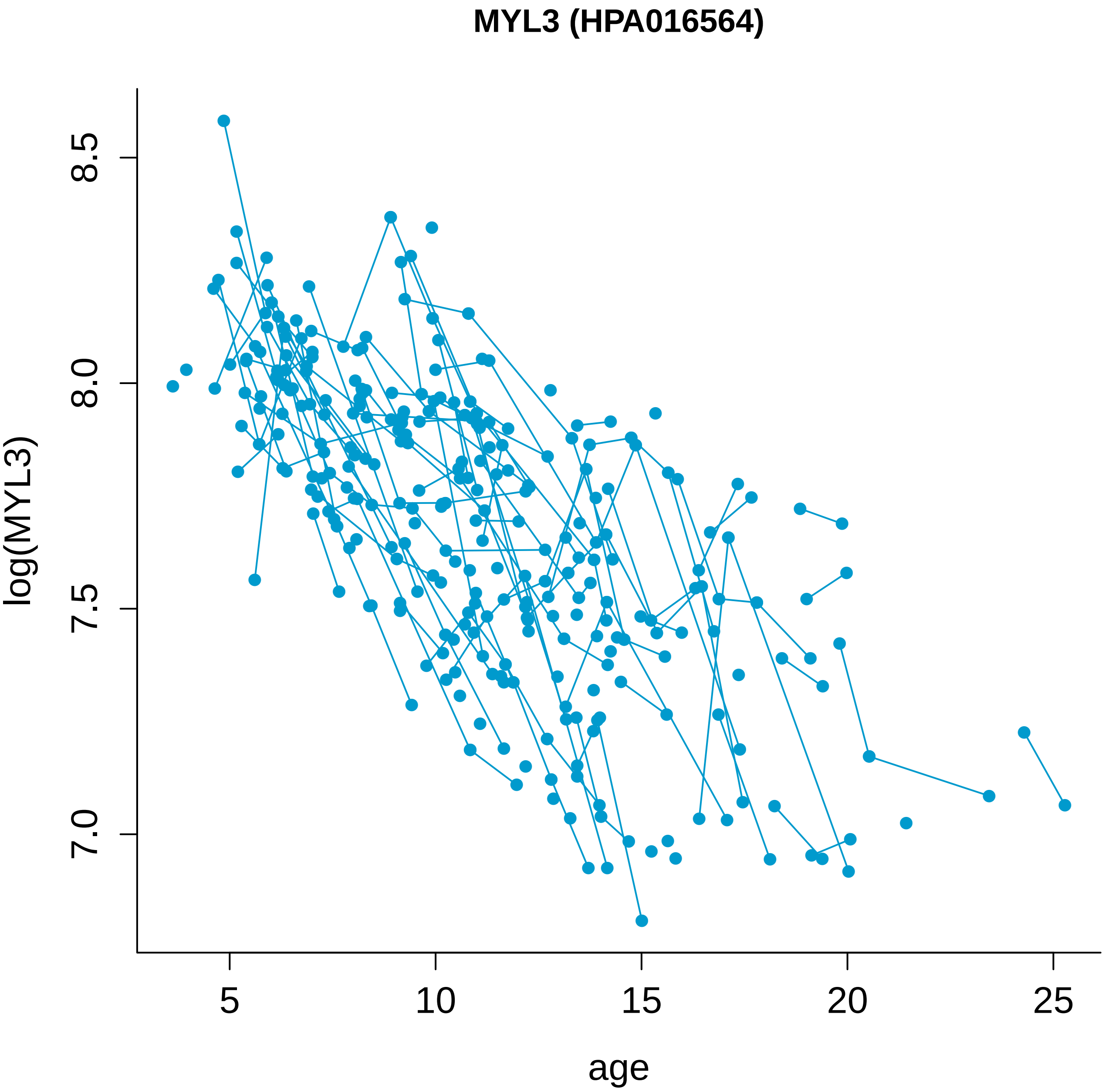
MYL3 (HPA016564)
log(MYL3)
8.5
8.0
7.5
7.0
5
10
15
20
25
age

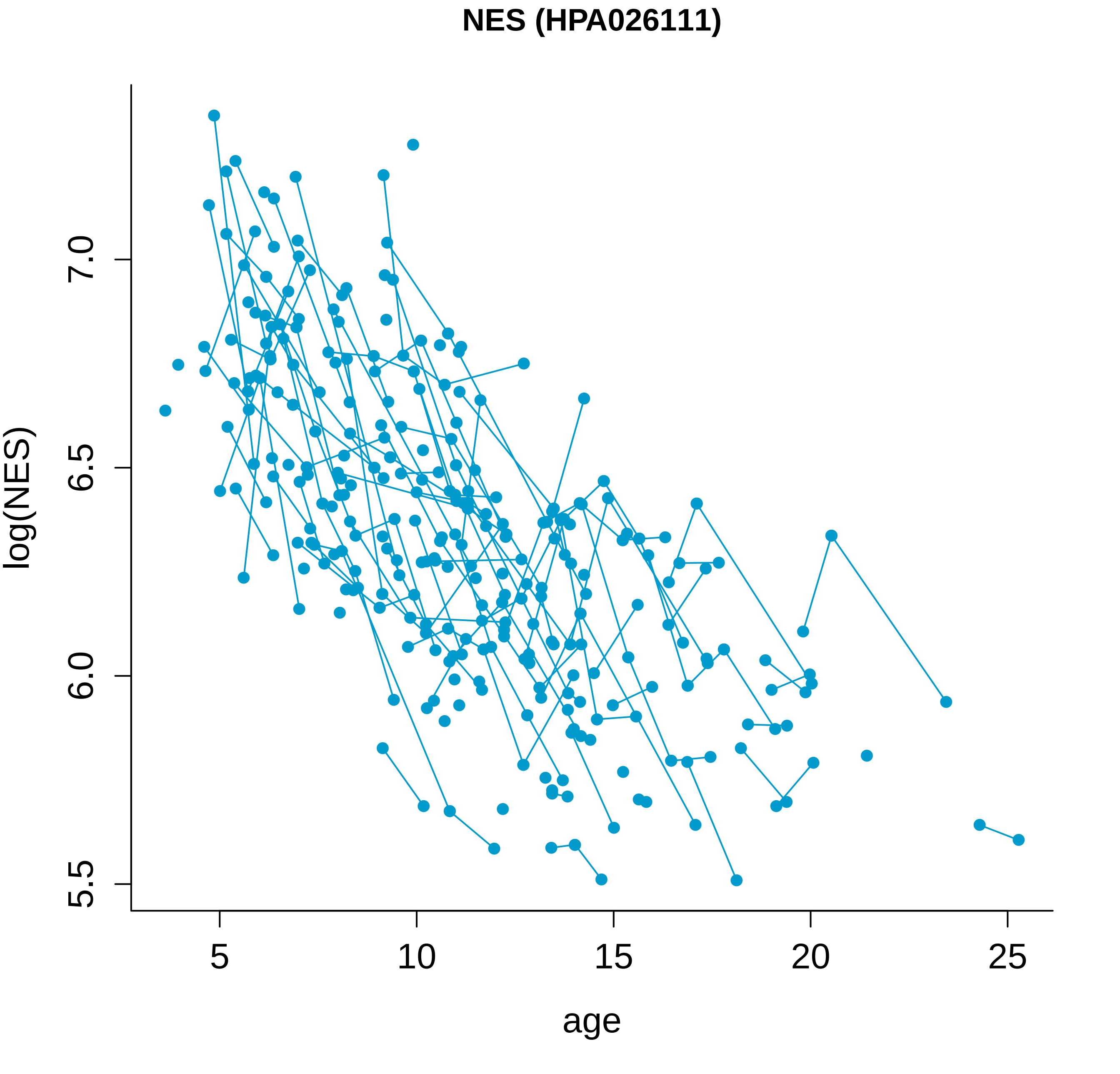
NES (HPA026111)
log(NES)
5.5
6.0
6.5
7.0
5
10
15
20
25
age

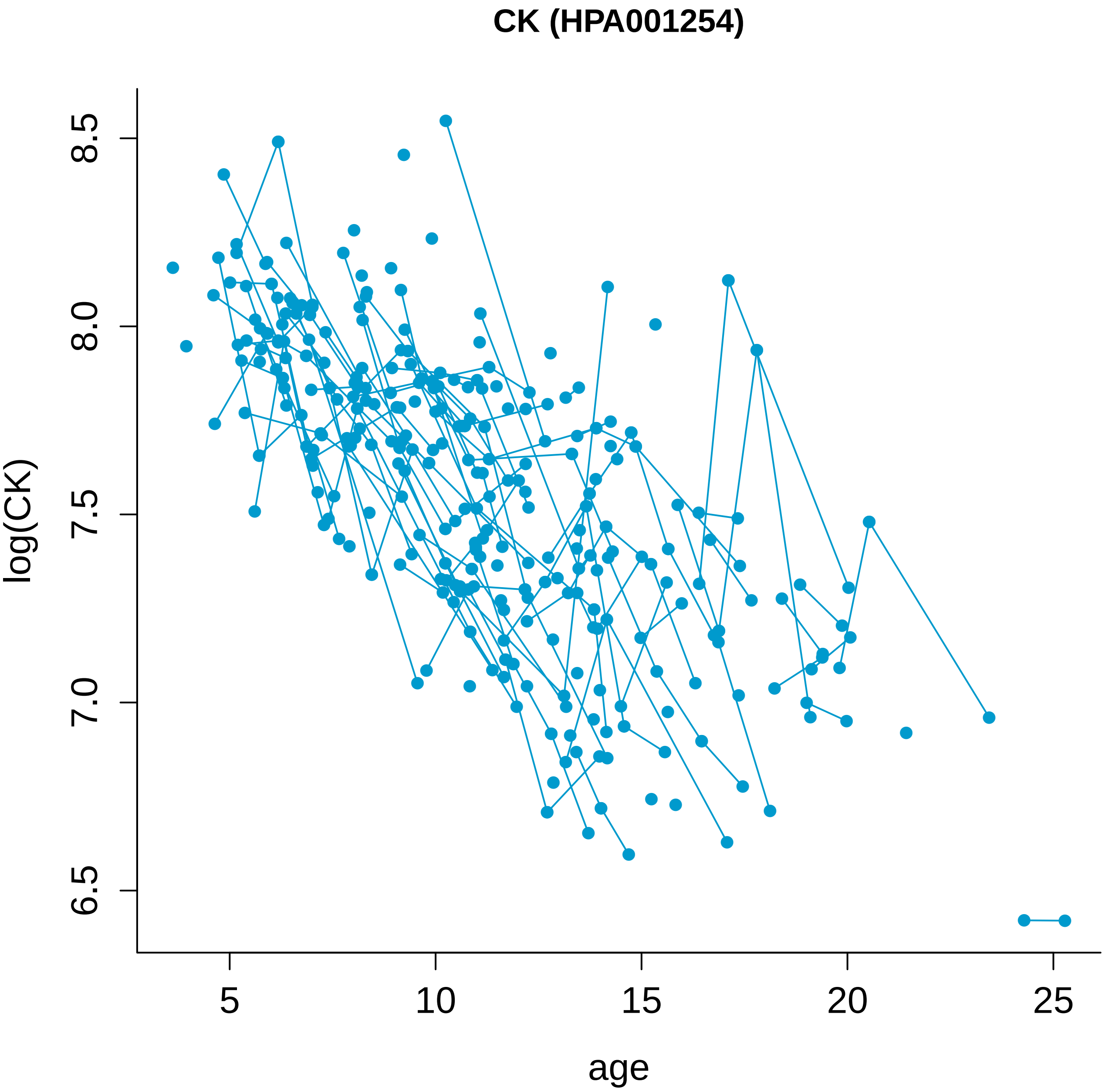
CK (HPA001254)
log(CK)
8.5
8.0
7.5
7.0
6.5
5
10
15
20
25
age

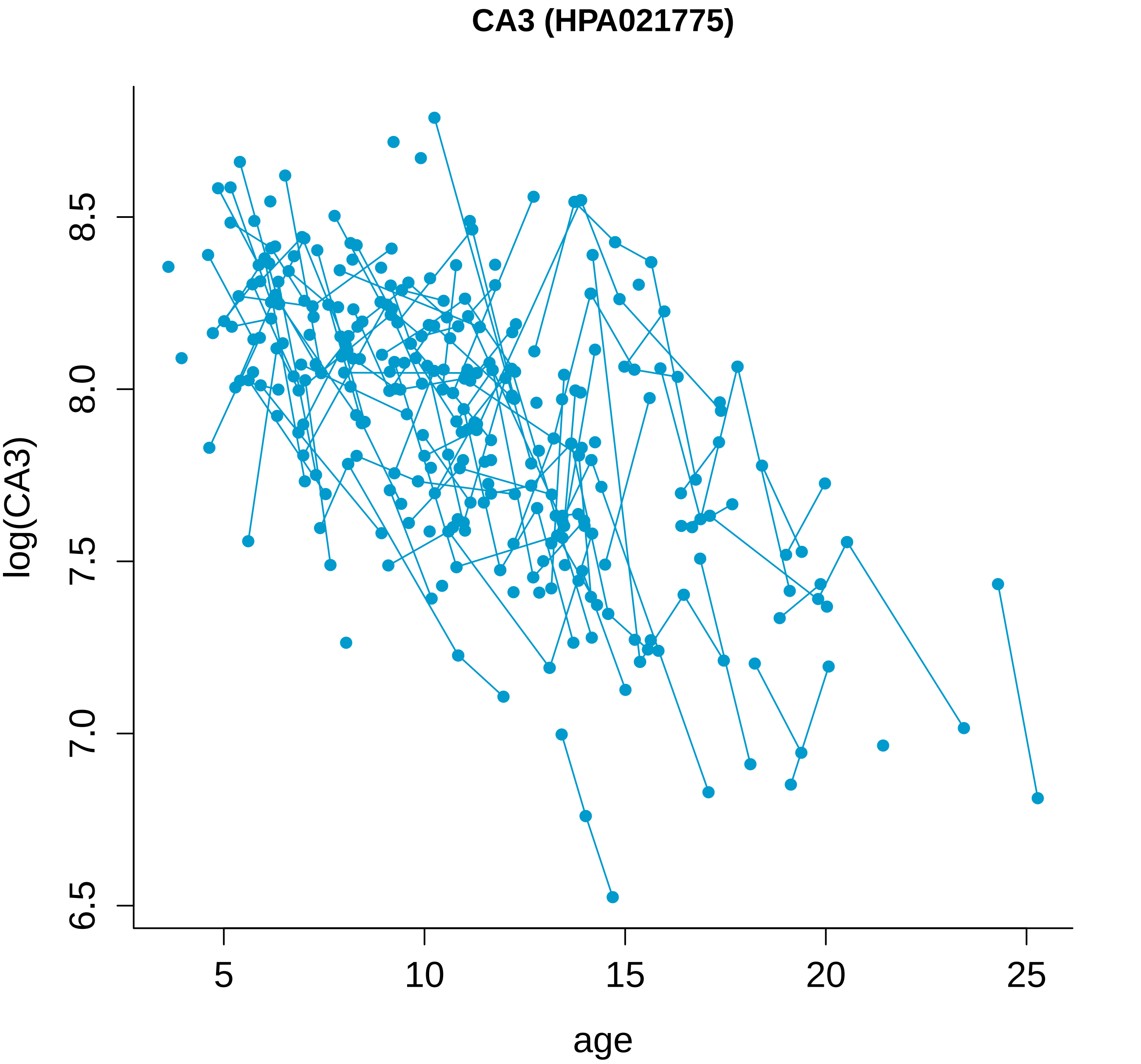

CA3 (HPA021775)
log(CA3)
6.5
7.0
7.5
8.0
8.5
age
5
10
15
20
25

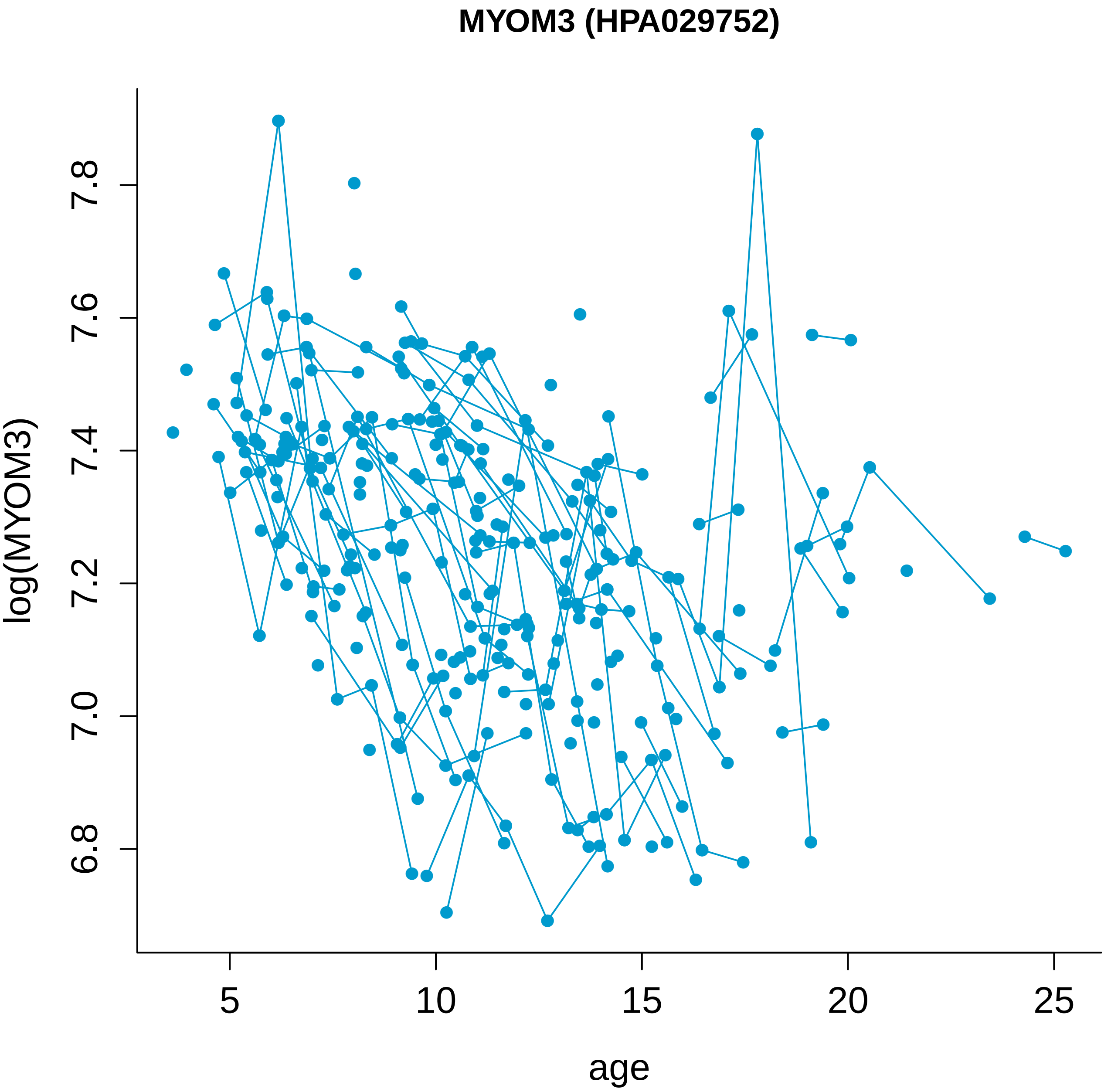

MYOM3 (HPA029752)
log(MYOM3)
7.8
7.6
7.4
7.2
7.0
6.8
5
10
15
20
25
age

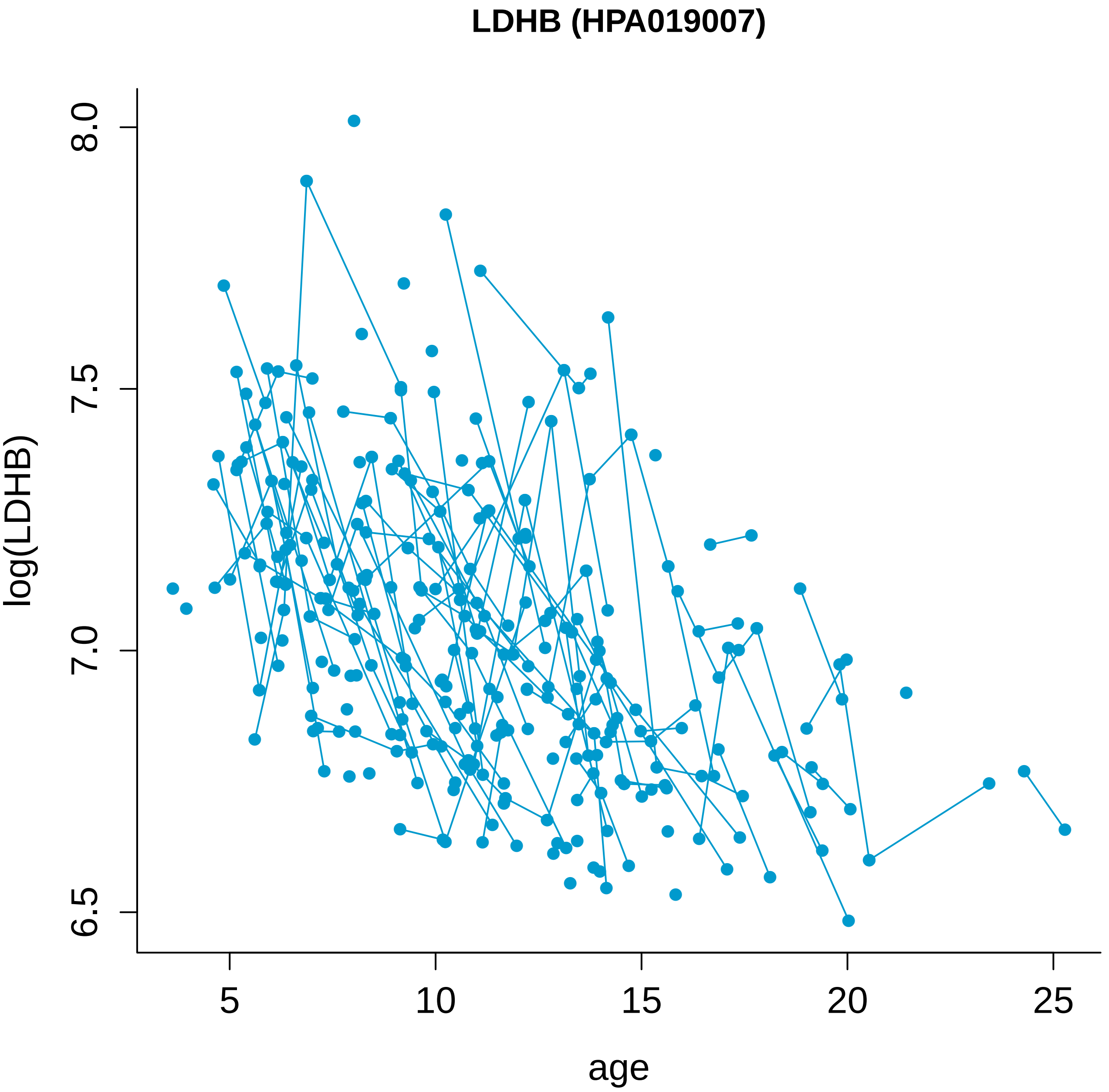
LDHB (HPA019007)
log(LDHB)
8.0
7.5
7.0
6.5
5
10
15
20
25
age

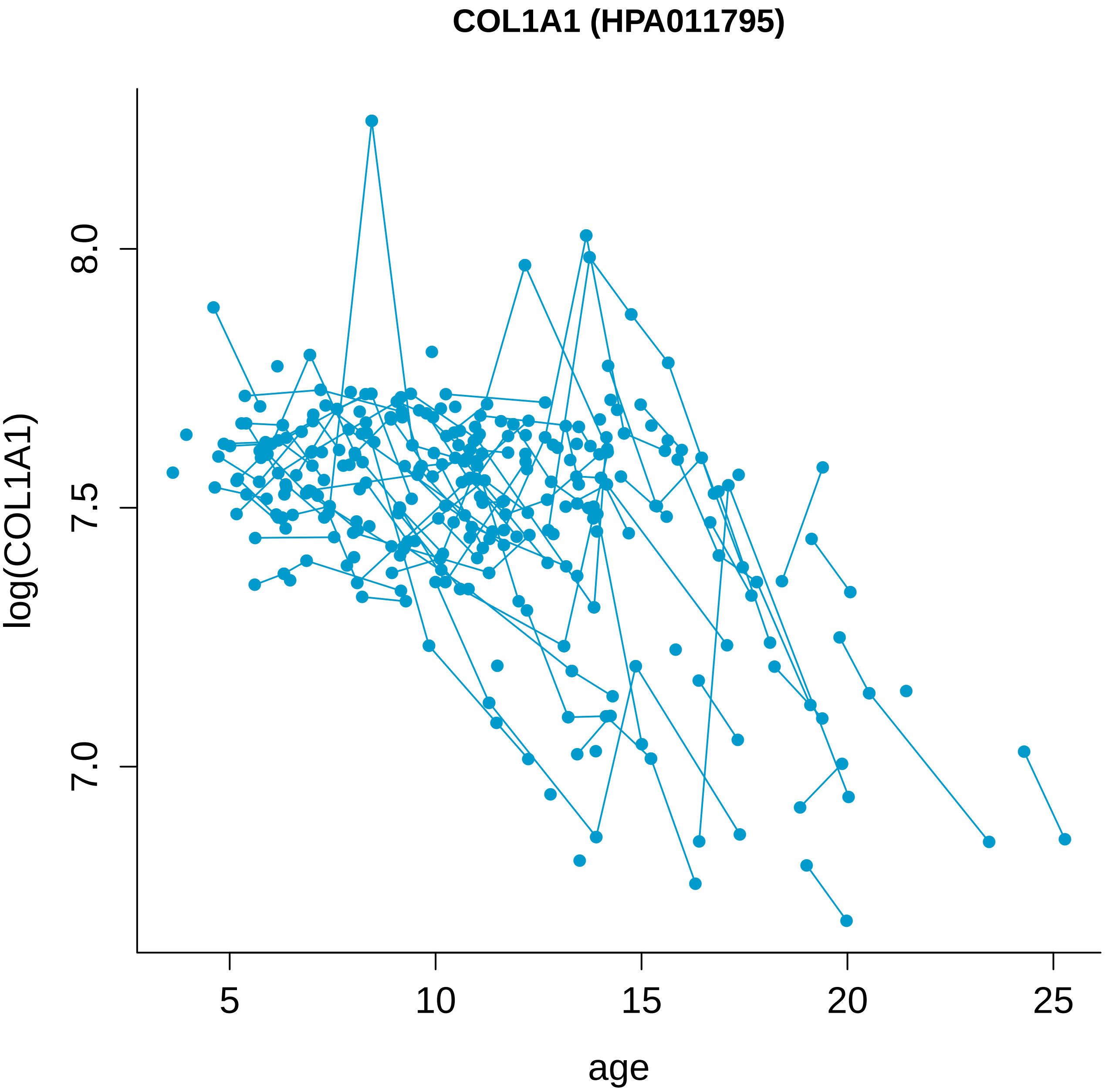
COL1A1 (HPA011795)
log(COL1A1)
8.0
7.5
7.0
5
10
15
20
25
age

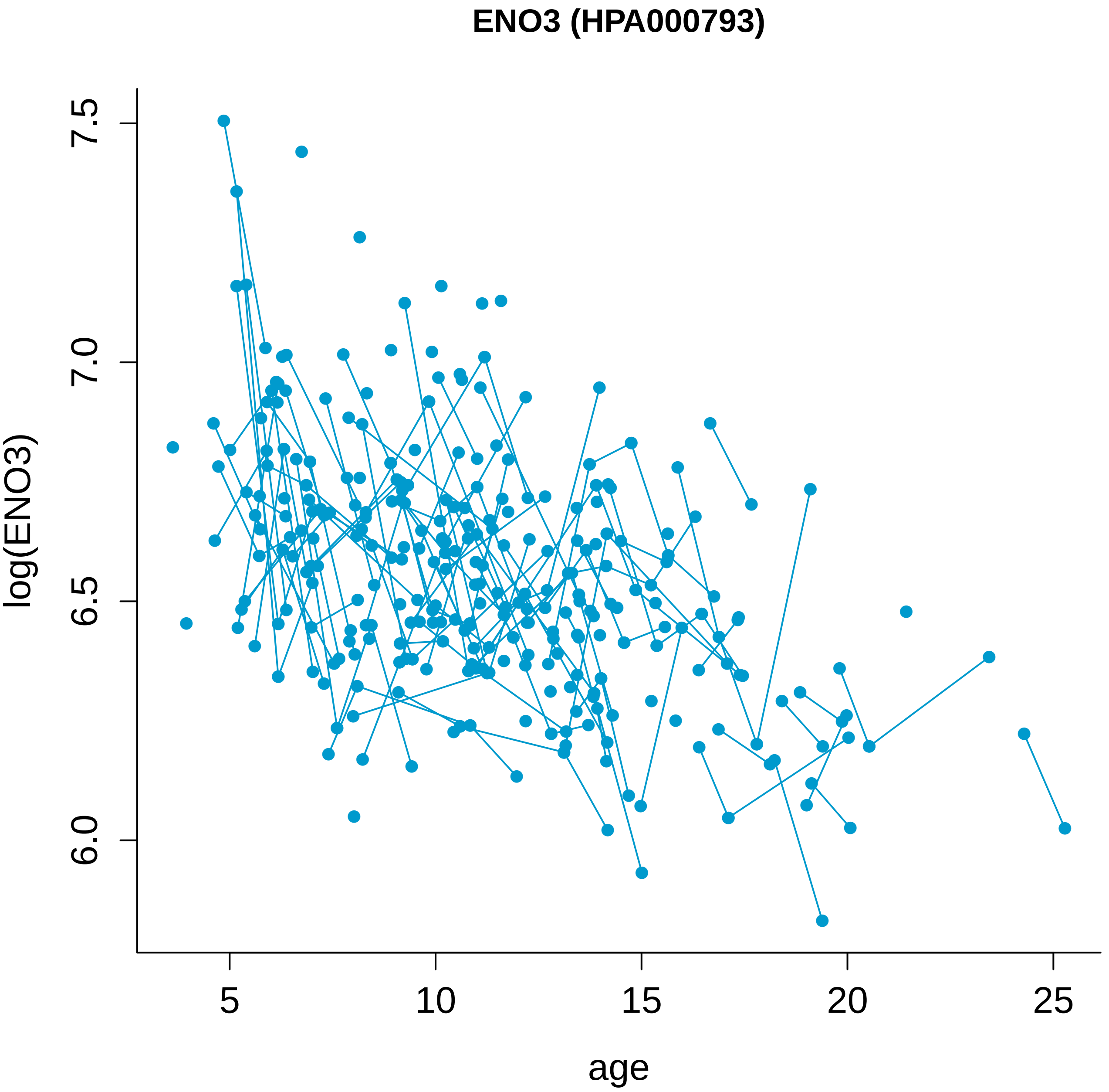
ENO3 (HPA000793)
log(ENO3)
7.5
7.0
6.5
6.0
5
10
15
20
25
age

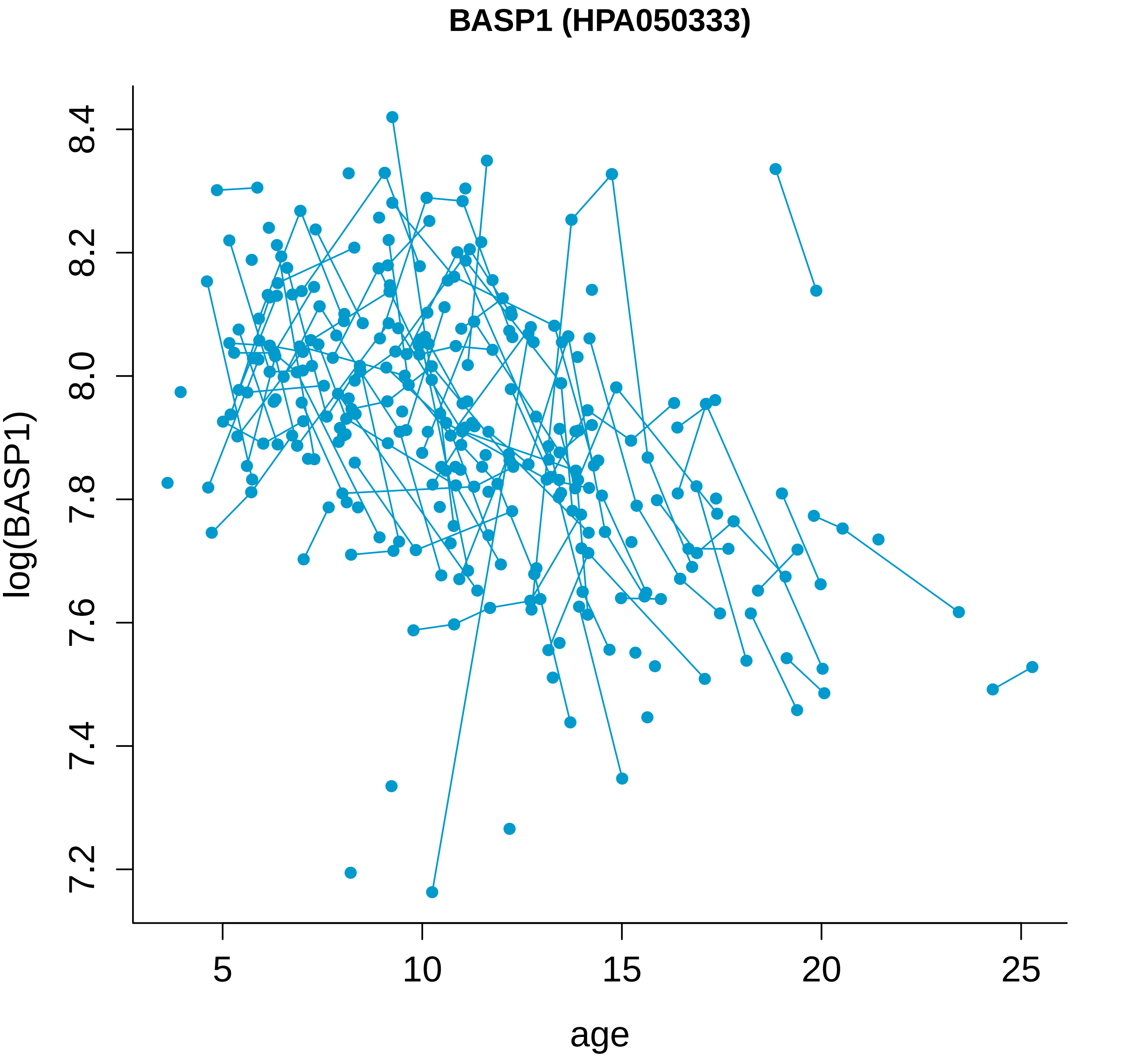

BASP1 (HPA050333)
log(BASP1)
8.4
8.2
8.0
7.8
7.6
7.4
7.2
5
10
15
20
25
age

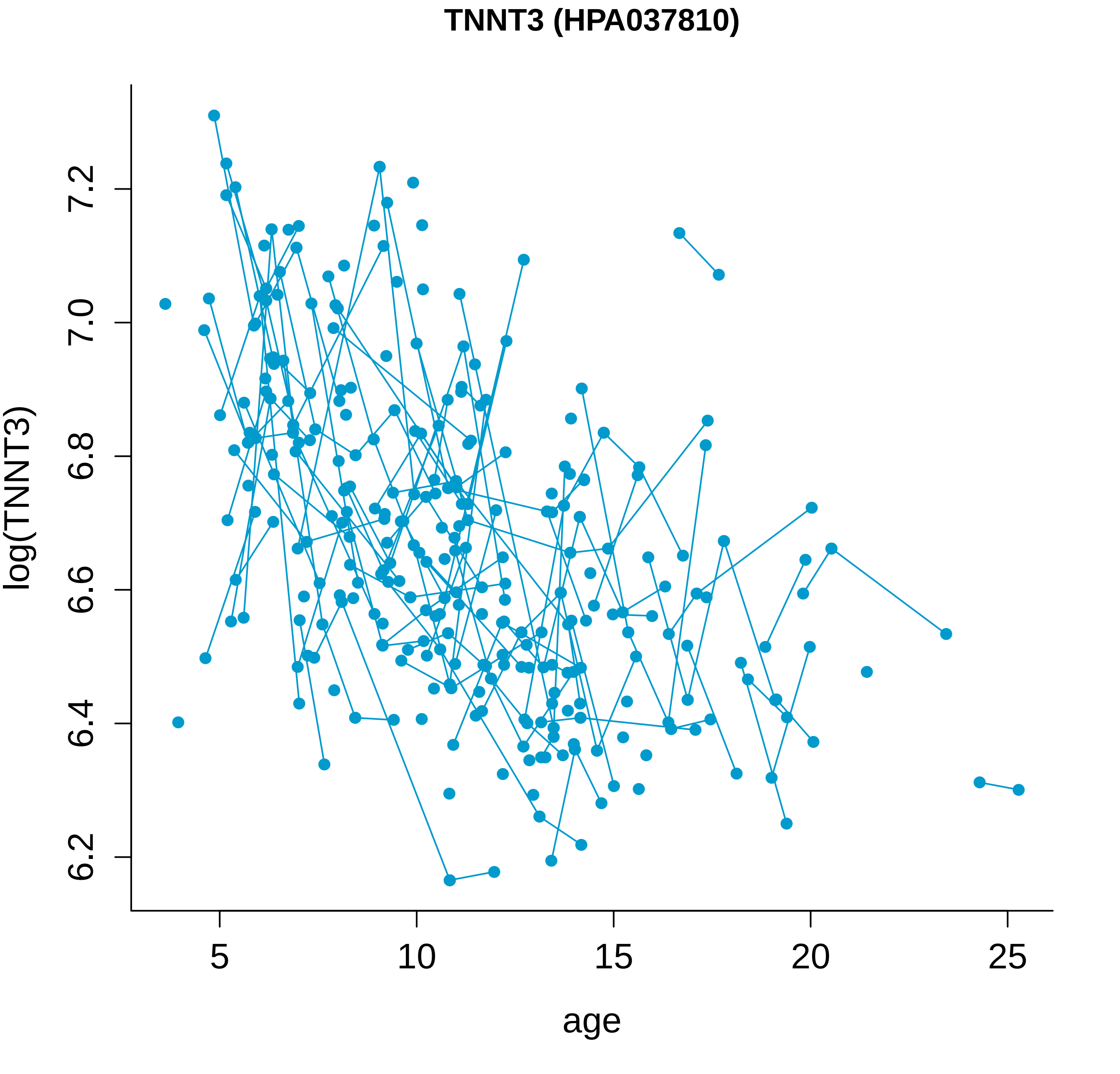
TNNT3 (HPA037810)
log(TNNT3)
7.2
7.0
6.8
6.6
6.4
6.2
5
10
15
20
25
age

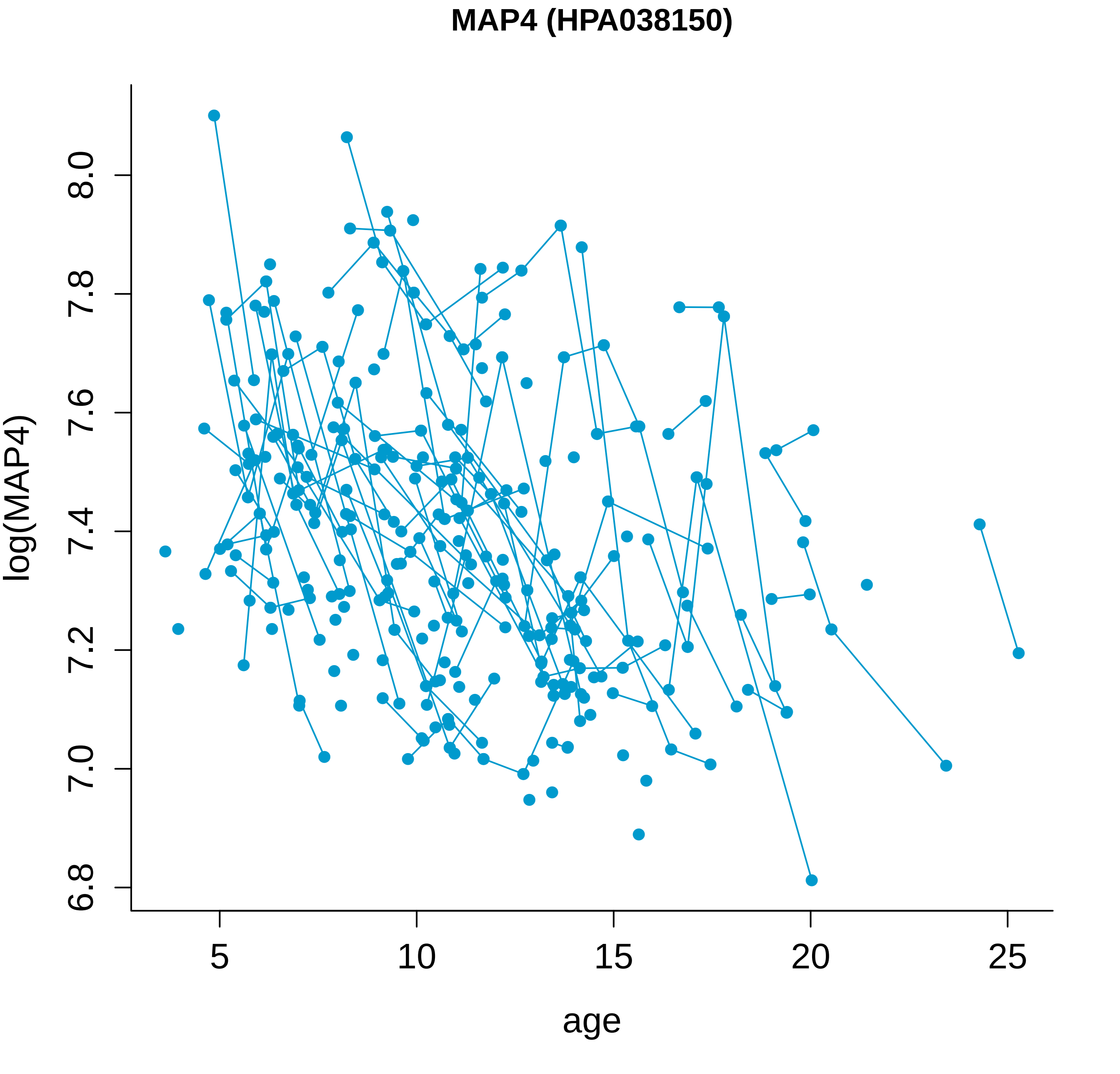
MAP4 (HPA038150)
log(MAP4)
6.8
7.0
7.2
7.4
7.6
7.8
8.0
5
10
15
20
25
age

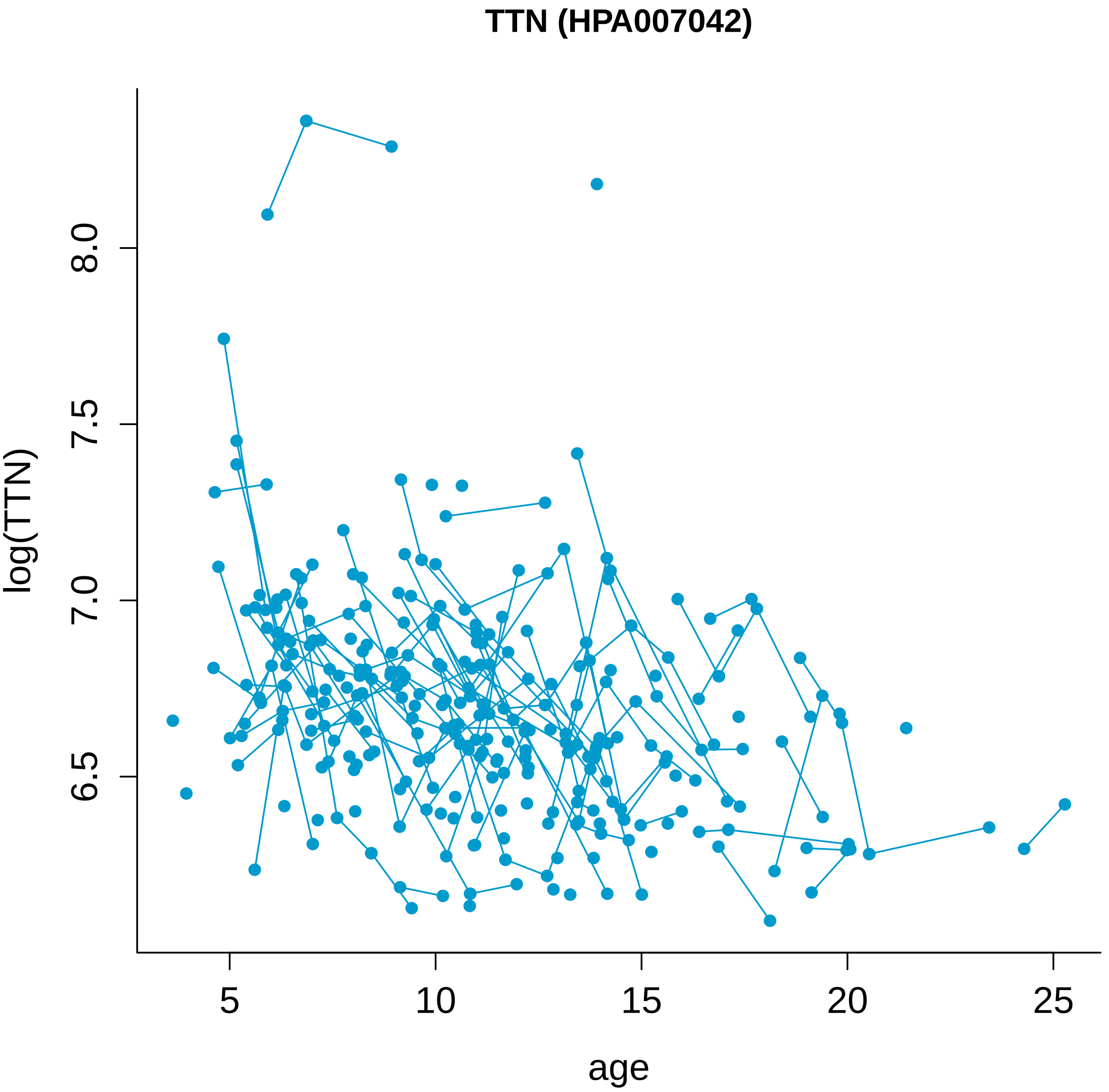

TTN (HPA007042)
log(TTN)
8.0
7.5
7.0
6.5
5
10
15
20
25
age

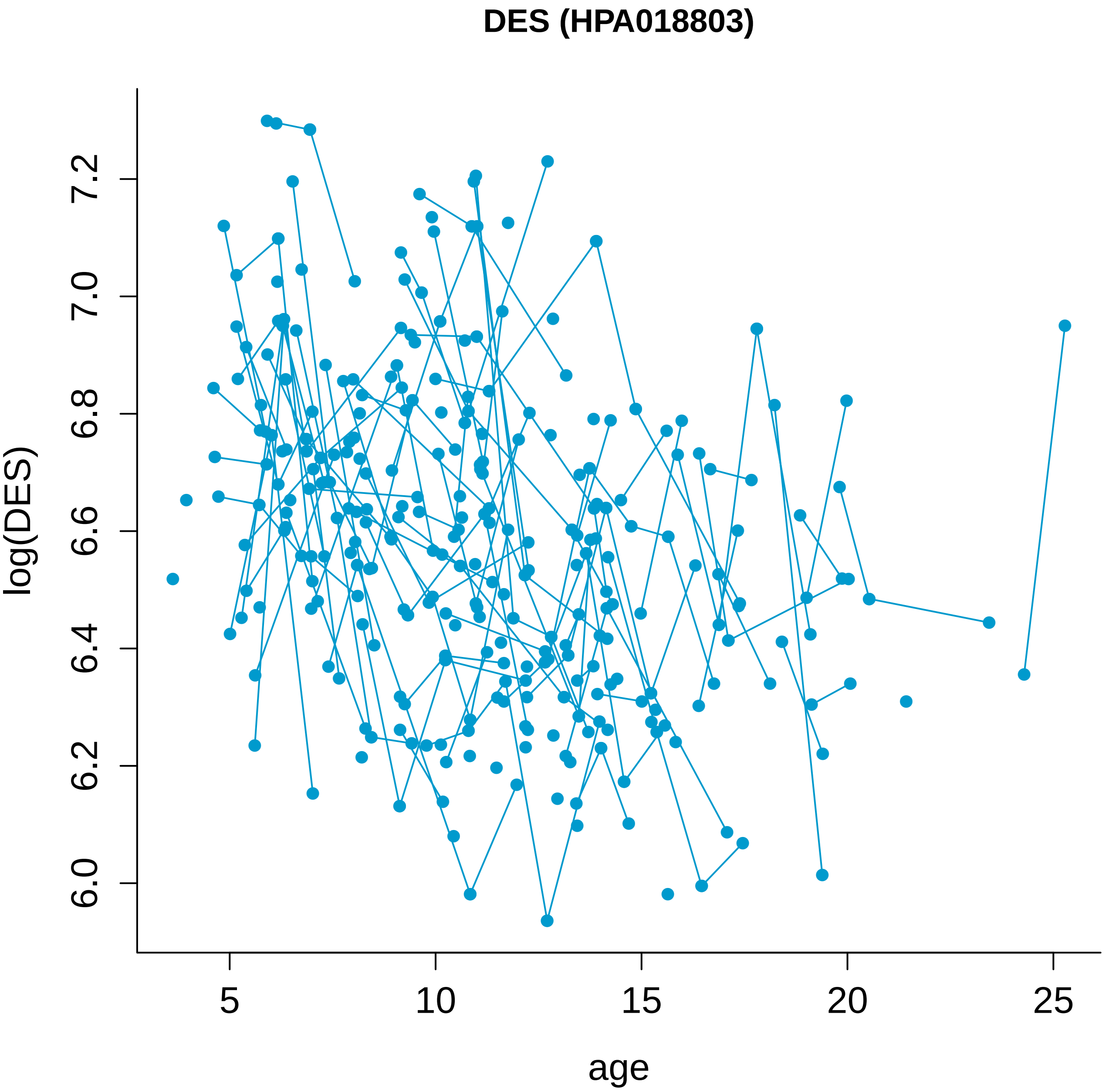
DES (HPA018803)
log(DES)
6.0
6.2
6.4
6.6
6.8
7.0
7.2
age
5
10
15
20
25

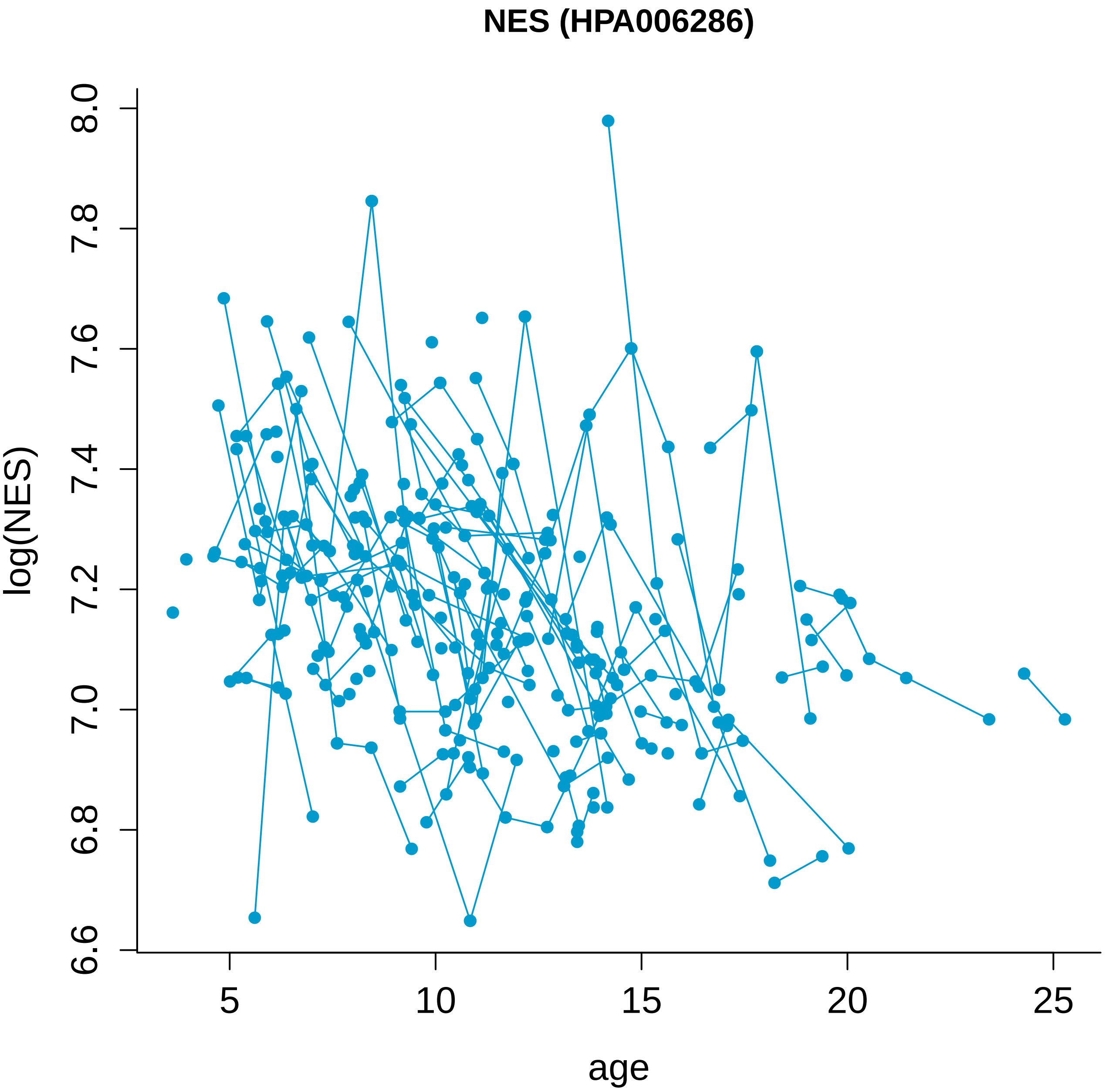
NES (HPA006286)
log(NES)
8.0
7.8
7.6
7.4
7.2
7.0
6.8
6.6
5
10
15
20
25
age

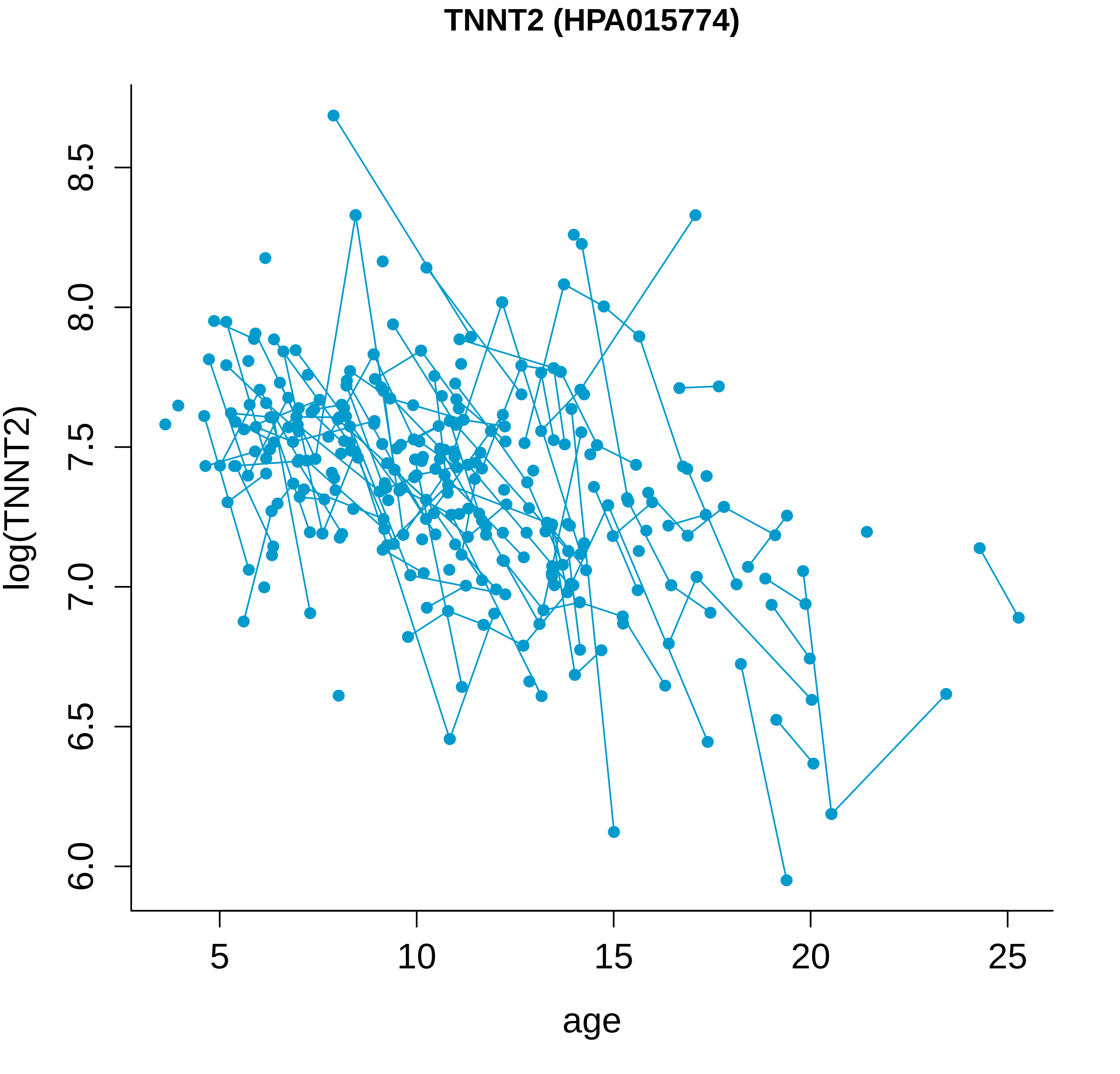
TNNT2 (HPA015774)
log(TNNT2)
6.0
6.5
7.0
7.5
8.0
8.5
5
10
15
20
25
age

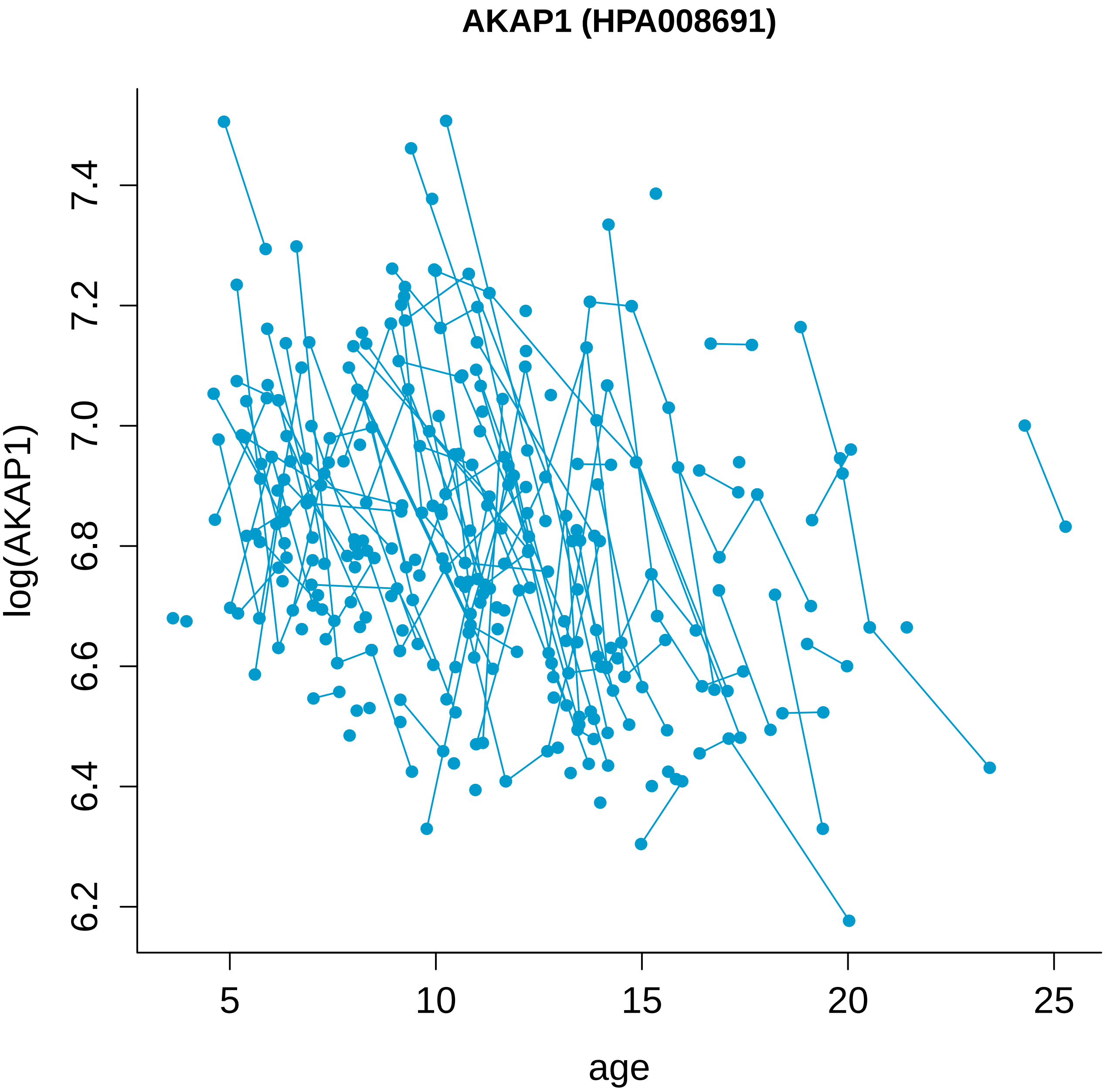

AKAP1 (HPA008691)
log(AKAP1)
age
6.2
6.4
6.6
6.8
7.0
7.2
7.4
5
10
15
20
25

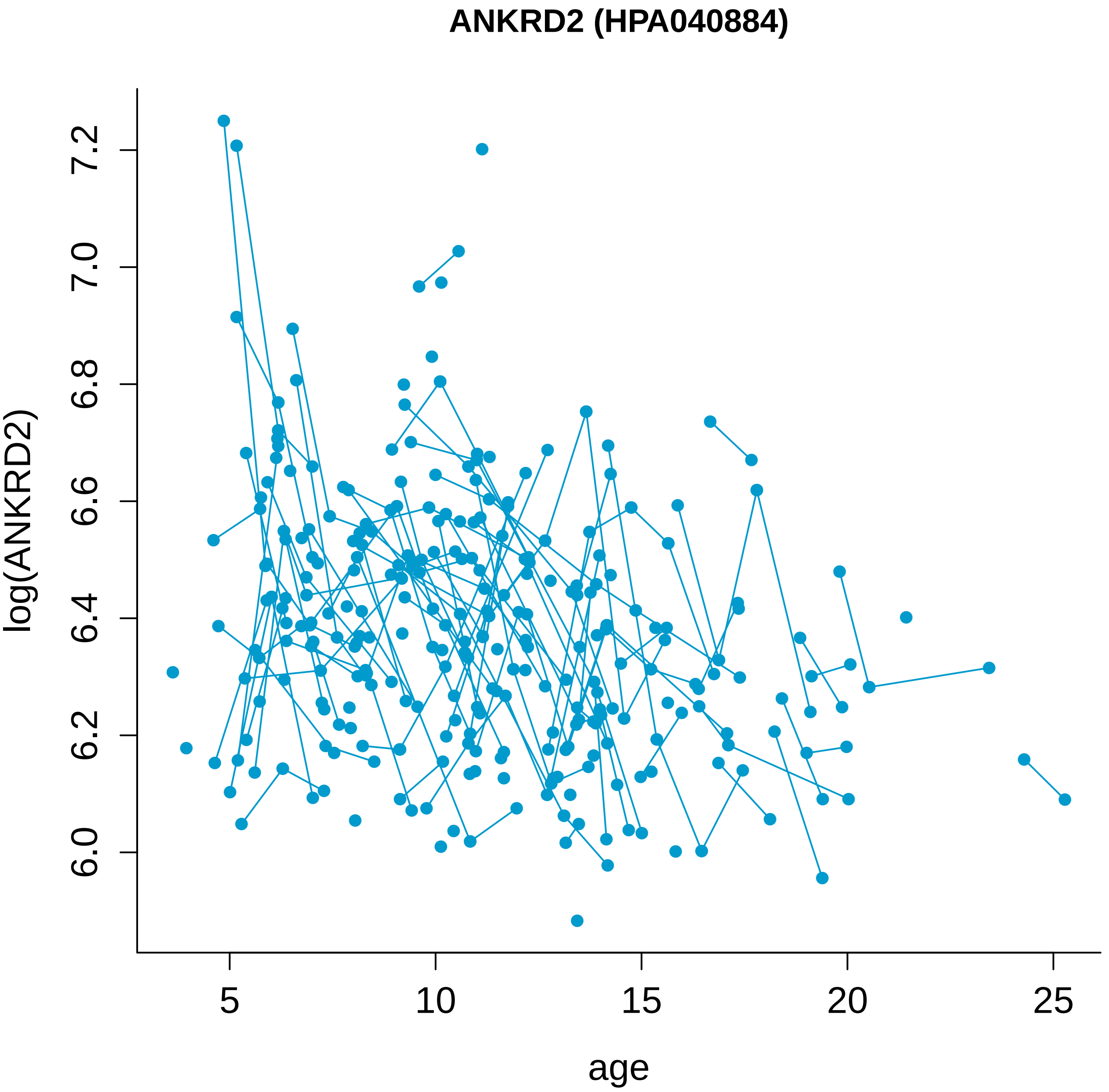

ANKRD2 (HPA040884)
log(ANKRD2)
age
6.0
6.2
6.4
6.6
6.8
7.0
7.2
5
10
15
20
25

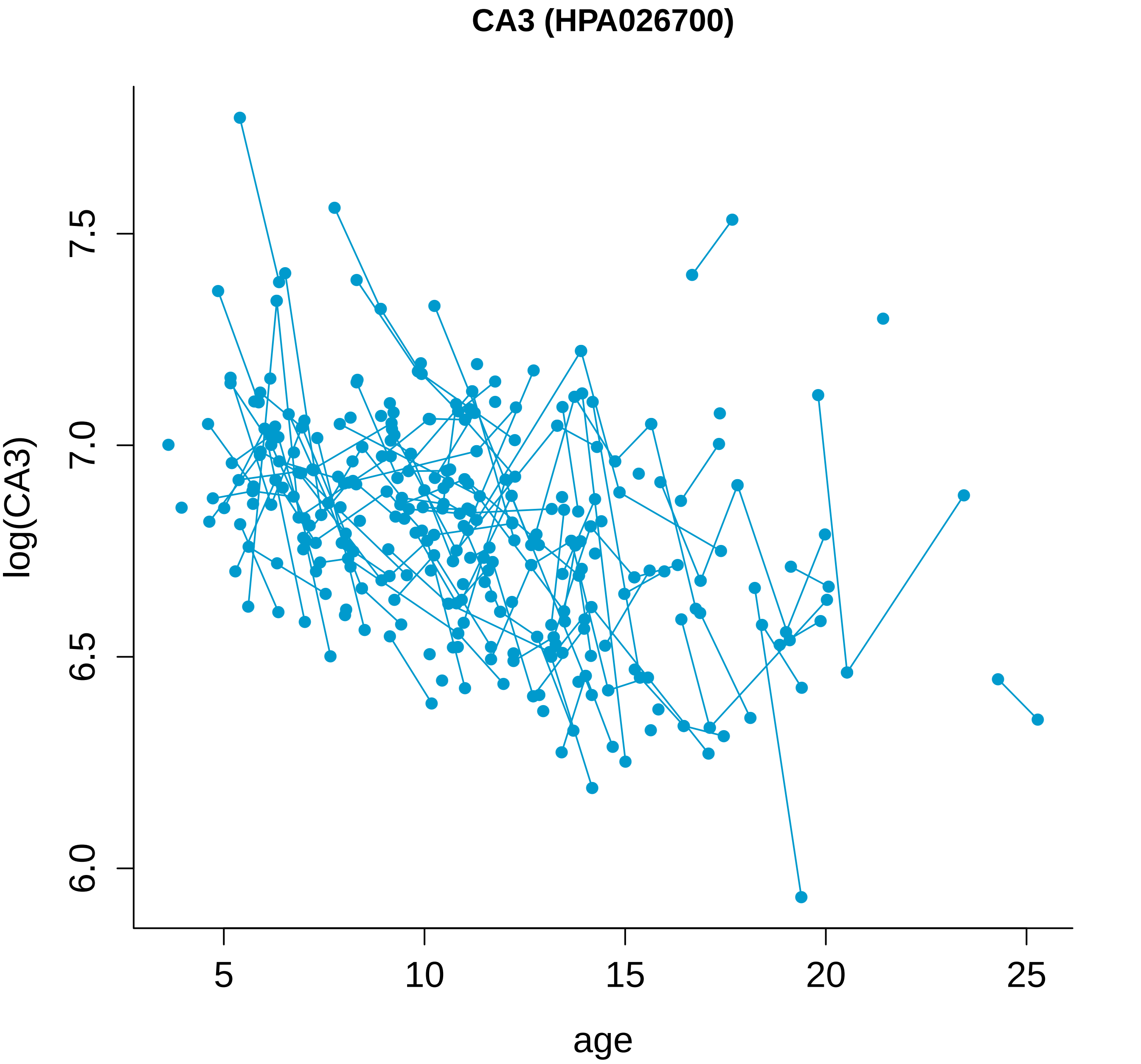
CA3 (HPA026700)
log(CA3)
age
6.0
6.5
7.0
7.5
5
10
15
20
25

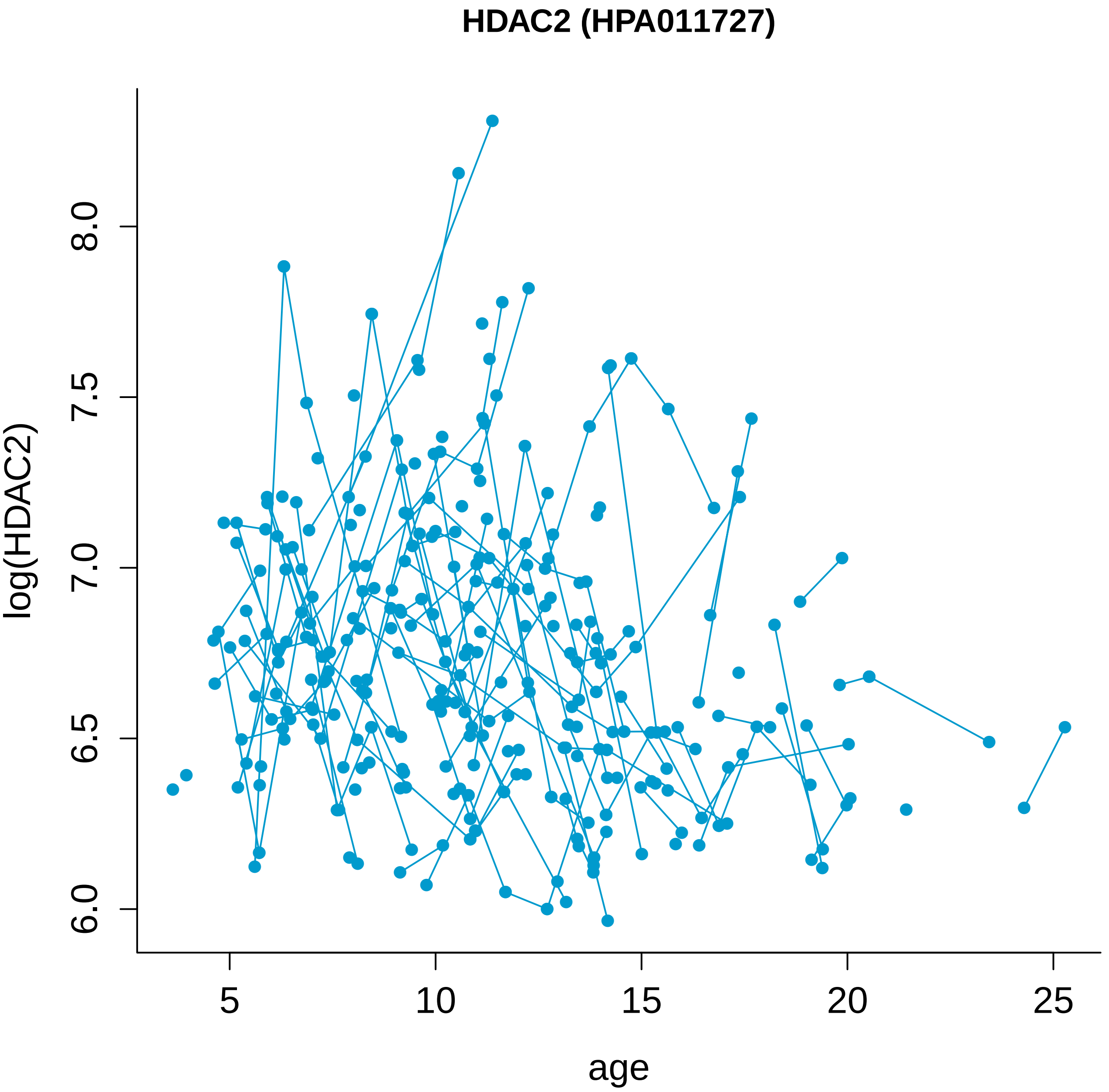
HDAC2 (HPA011727)
log(HDAC2)
8.0
7.5
7.0
6.5
6.0
5
10
15
20
25
age

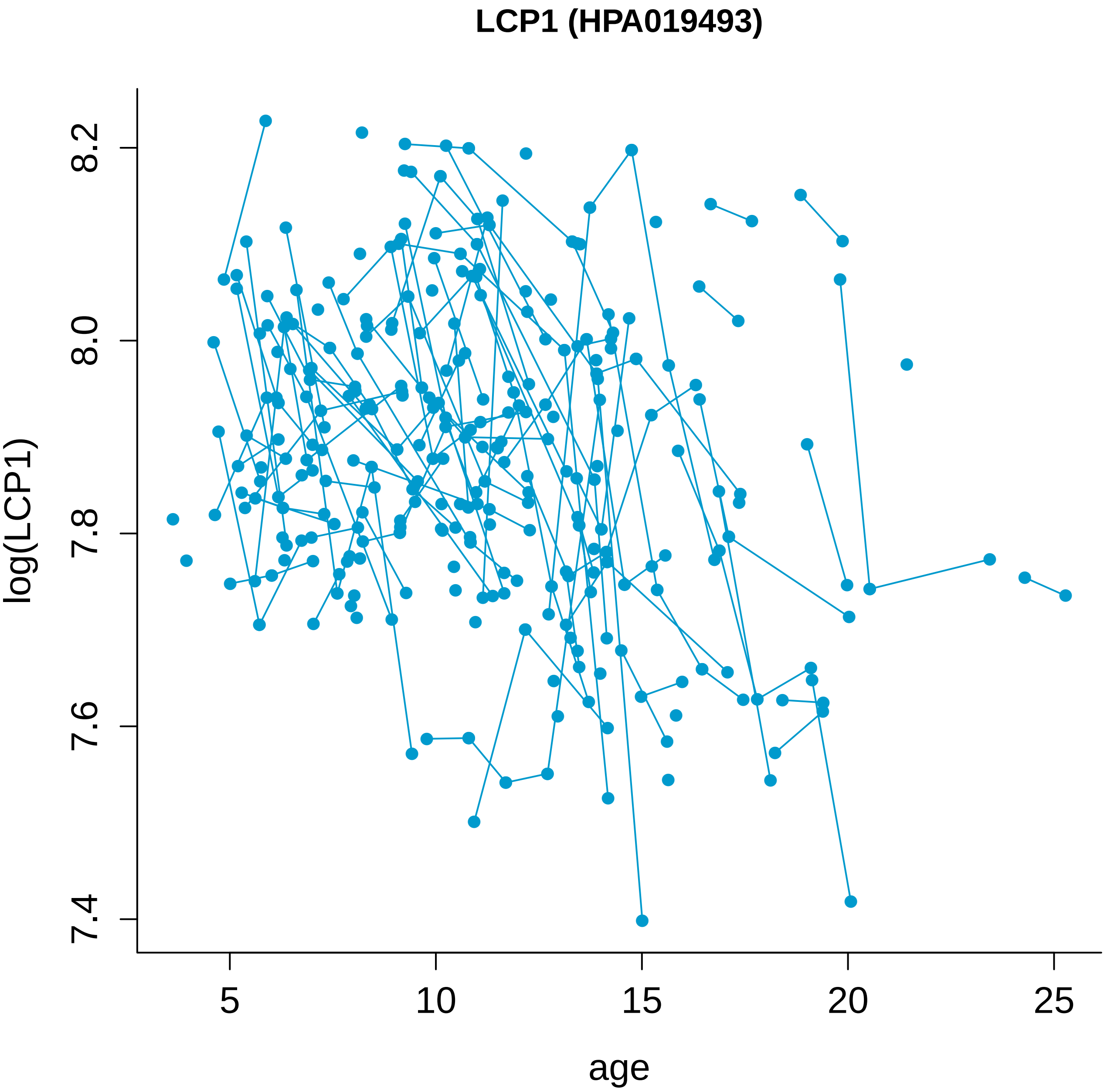

LCP1 (HPA019493)
log(LCP1)
8.2
8.0
7.8
7.6
7.4
5
10
15
20
25
age

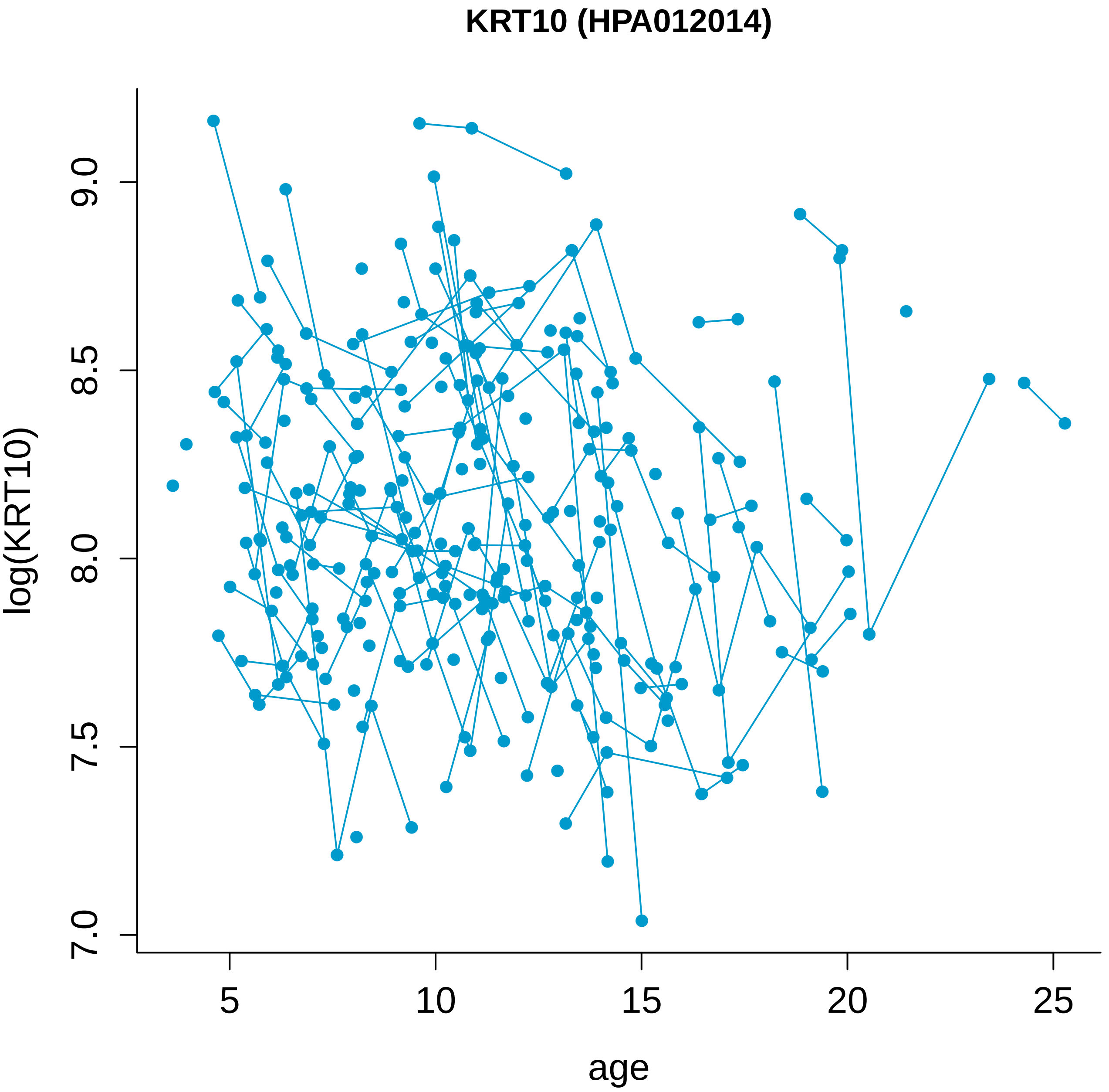
KRT10 (HPA012014)
log(KRT10)
7.0
7.5
8.0
8.5
9.0
age
5
10
15
20
25

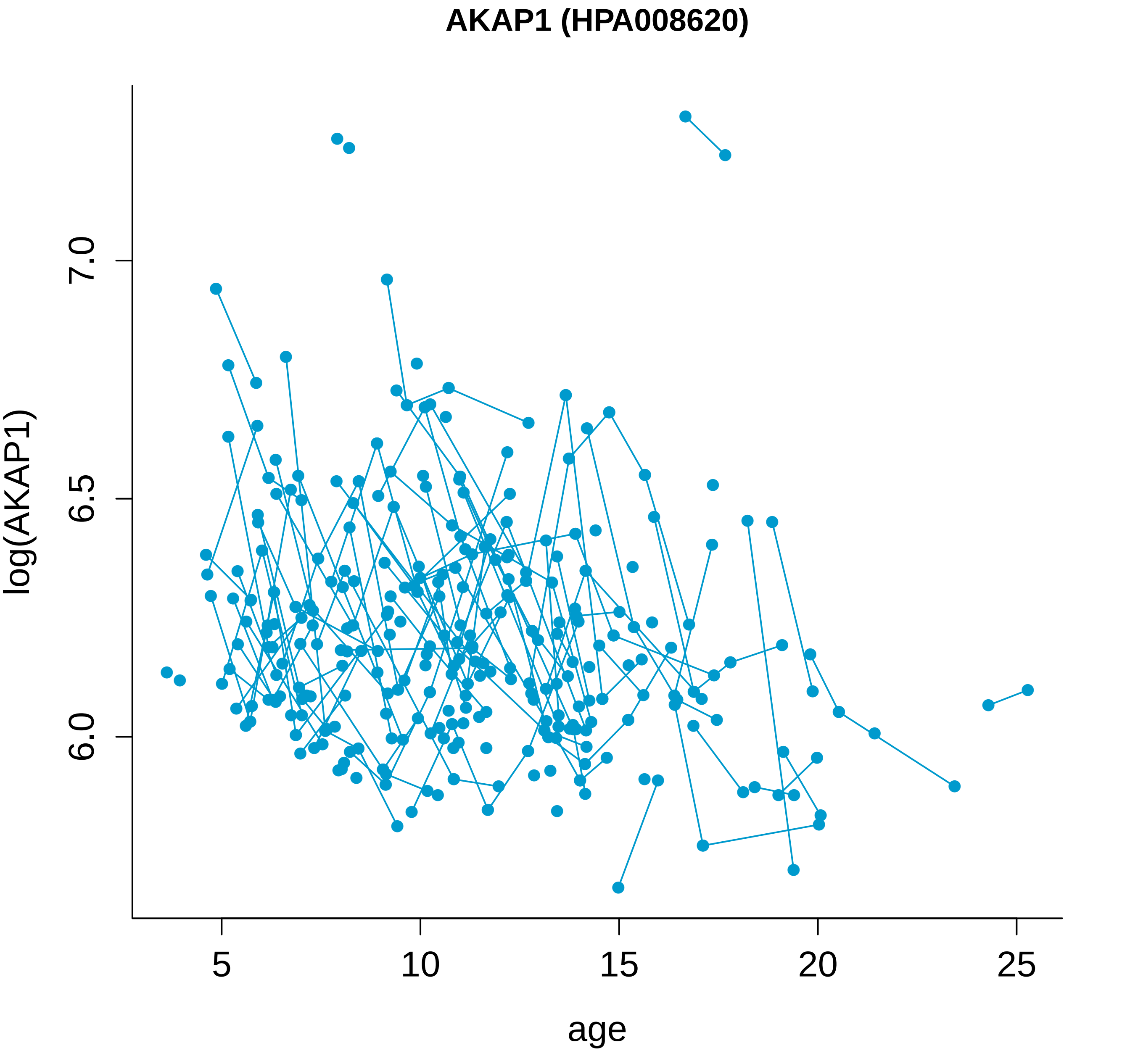
AKAP1 (HPA008620)
log(AKAP1)
6.0
6.5
7.0
5
10
15
20
25
age
