# Supplementary figures and images for: Longitudinal serum biomarker screening identifies malate dehydrogenase 2 as candidate prognostic biomarker for Duchenne muscular dystrophy
Source: J Cachexia Sarcopenia Muscle. 2019 Dec 27;11(2):505–17. doi: 10.1002/jcsm.12517 (PMC7113516; doi:10.1002/jcsm.12517)

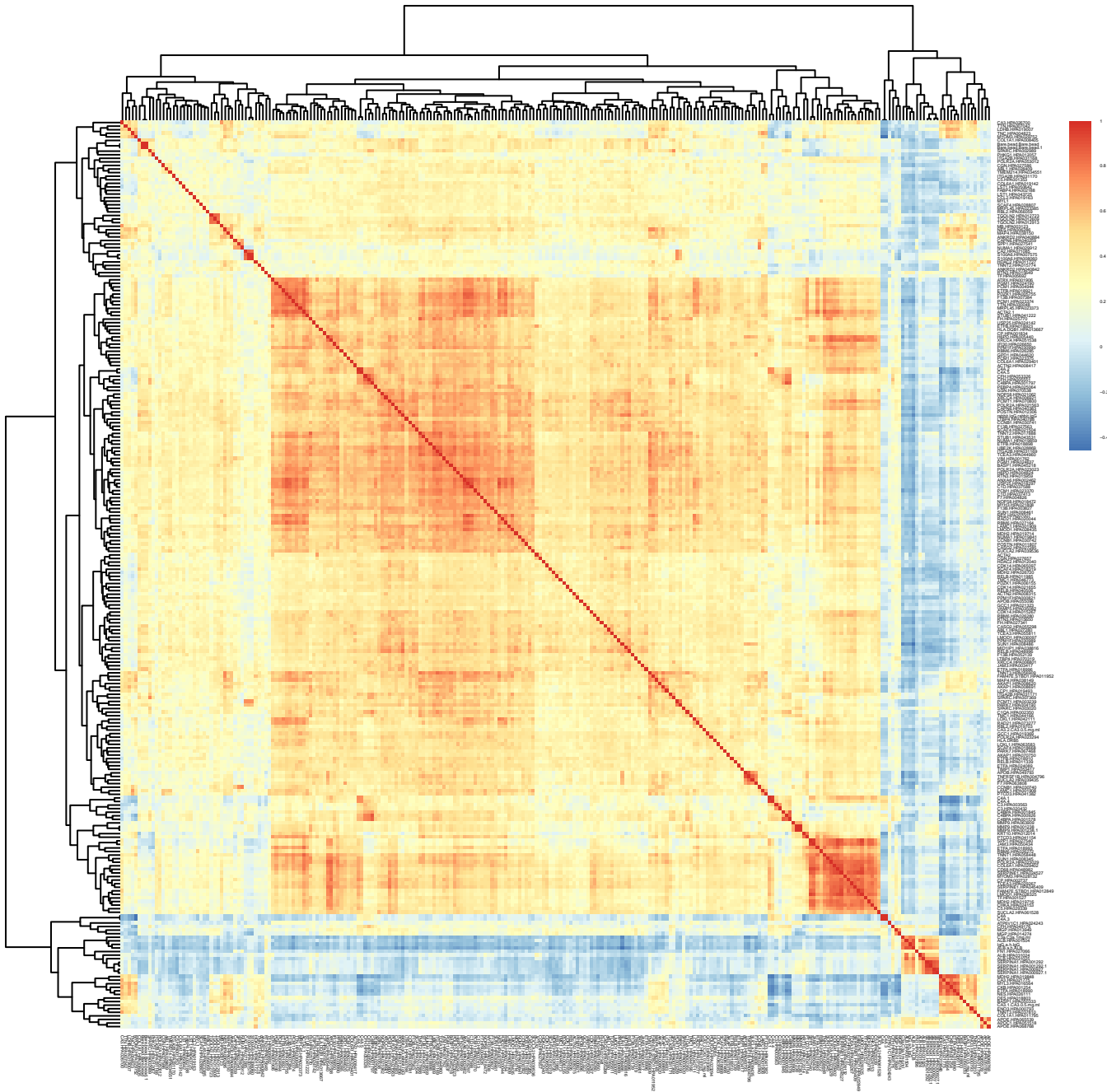

Supplement: Supplementary file 7 — File S3 Heatmap with pairwise Pearson's correlation coefficients between all antibodies throughout assay [file JCSM-11-505-s003.pdf]
